# Supplementary material for: A Multistage In Silico Study of Natural Potential Inhibitors Targeting SARS-CoV-2 Main Protease
Source: Int J Mol Sci. 2022 Jul 29;23(15):8407. doi: 10.3390/ijms23158407 (PMC9369012; doi:10.3390/ijms23158407)
Supplement: Supplementary file 1 [file ijms-23-08407-s001.zip › ijms-1811462-supplementary.pdf]

Supporting Materials

A Multistage In Silico Study of Natural Potential Inhibitors Targeting SARS-CoV-2 Main Protease

Eslam B. Elkaeed [1,*], Ibrahim H. Eissa [2], Hazem Elkady [2], Ahmed Abdelalim [3], Ahmad M. Alqaisi [4], Aisha A. Alsfouk [5], Alaa Elwan [2] and Ahmed M. Metwaly [6,7,*]

[1] Department of Pharmaceutical Sciences, College of Pharmacy, AlMaarefa University, Riyadh 13713, Saudi Arabia
[2] Pharmaceutical Medicinal Chemistry & Drug Design Department, Faculty of Pharmacy (Boys), Al-Azhar University, Cairo 11884, Egypt
[3] Faculty of Pharmacy (Boys), Al-Azhar University, Cairo 11884, Egypt
[4] Department of Chemistry, University of Jordan, Amman 11942, Jordan
[5] Department of Pharmaceutical Sciences, College of Pharmacy, Princess Nourah bint Abdulrahman University, P.O. Box 84428, Riyadh 11671, Saudi Arabia
[6] Pharmacognosy and Medicinal Plants Department, Faculty of Pharmacy (Boys), Al-Azhar University, Cairo 11884, Egypt
[7] Biopharmaceutical Products Research Department, Genetic Engineering and Biotechnology Research Institute, City of Scientific Research and Technological Applications (SRTA-City), Alexandria 21934, Egypt

Content

**Figure S1.** The crystal structure of the ligand-free M^{pro} (PDB ID: 5R84)	
**Figure S2.** Chemical structures of the examined natural antiviral compounds	
**Figure S3.** Superimposition of the re-docked conformer of the co-crystallized ligand over the original one with an RMSD value of 0.73 °A	
**Figure S4.** 3D of **GWS** in the active site of M^{pro}.	
**Figure S5.** 2D of **112** in the active site of $M^{pro.}$	
**Figure S6.** 3D of **291** in the active site of M^{pro}.	
**Figure S7.** 3D of **292** in the active of site M^{pro}.	
**Figure S8.** 3D of **293** in the active site of M^{pro}.	
**Figure S9.** 3D& 2D of **303** in the active site M^{pro}.	
**Figure S10.** 3D & 2D of **305** in the active site M^{pro}	
**Figure S11.** Hydrogen number plot that formed between caprolactin A-M^{pro} complex.	
**Figure S12.** Representative poses of caprolactin A in the M^{pro} pocket. Hydrogen bond interactions purple dashed	
**Figure S13.** Contact frequency plot of main protease residues bonded with caprolactin A.	
**Table S1 :** Molecular properties of metabolites having structural similarity with **GWS**	
Method	Molecular Similarity
	Pharmacophore
	Docking studies
	ADMET studies
	Toxicity studies
	DFT studies
	Molecular dynamic simulations
Toxicity report	

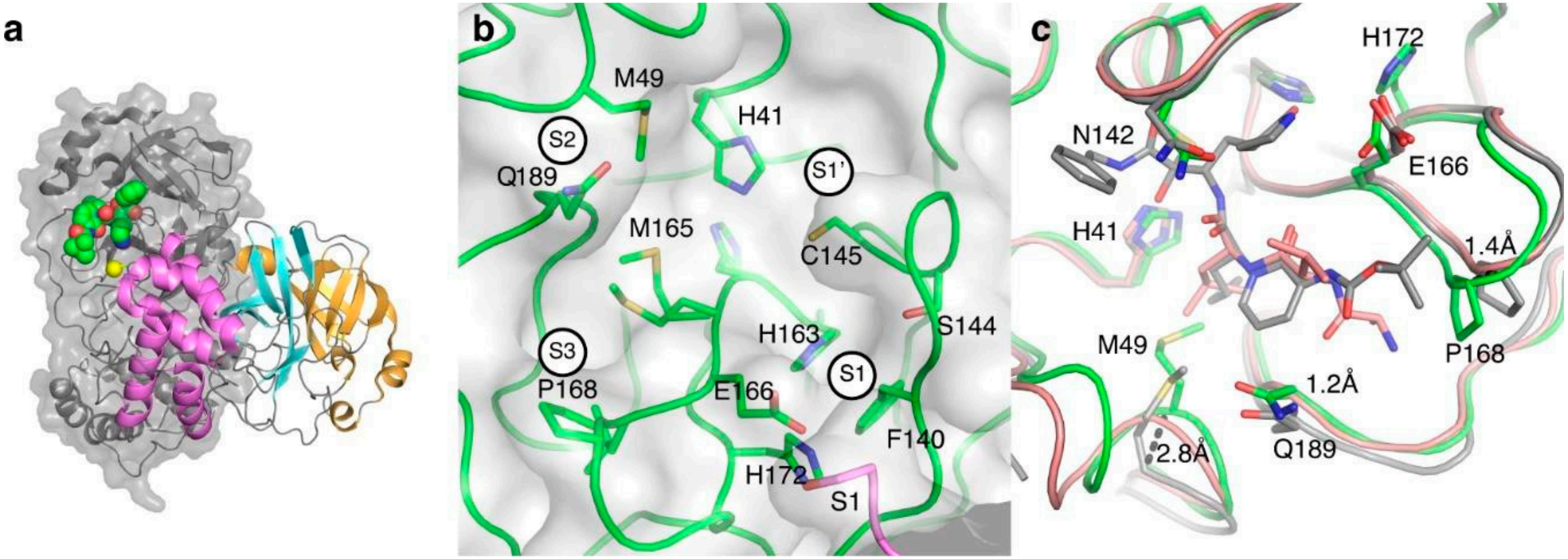

Figure S1. The crystal structure of the ligand-free M^pro (PDB ID: 5R84) a: cartoon description of the M^pro dimer, b: the active site residues, c: active site plasticity

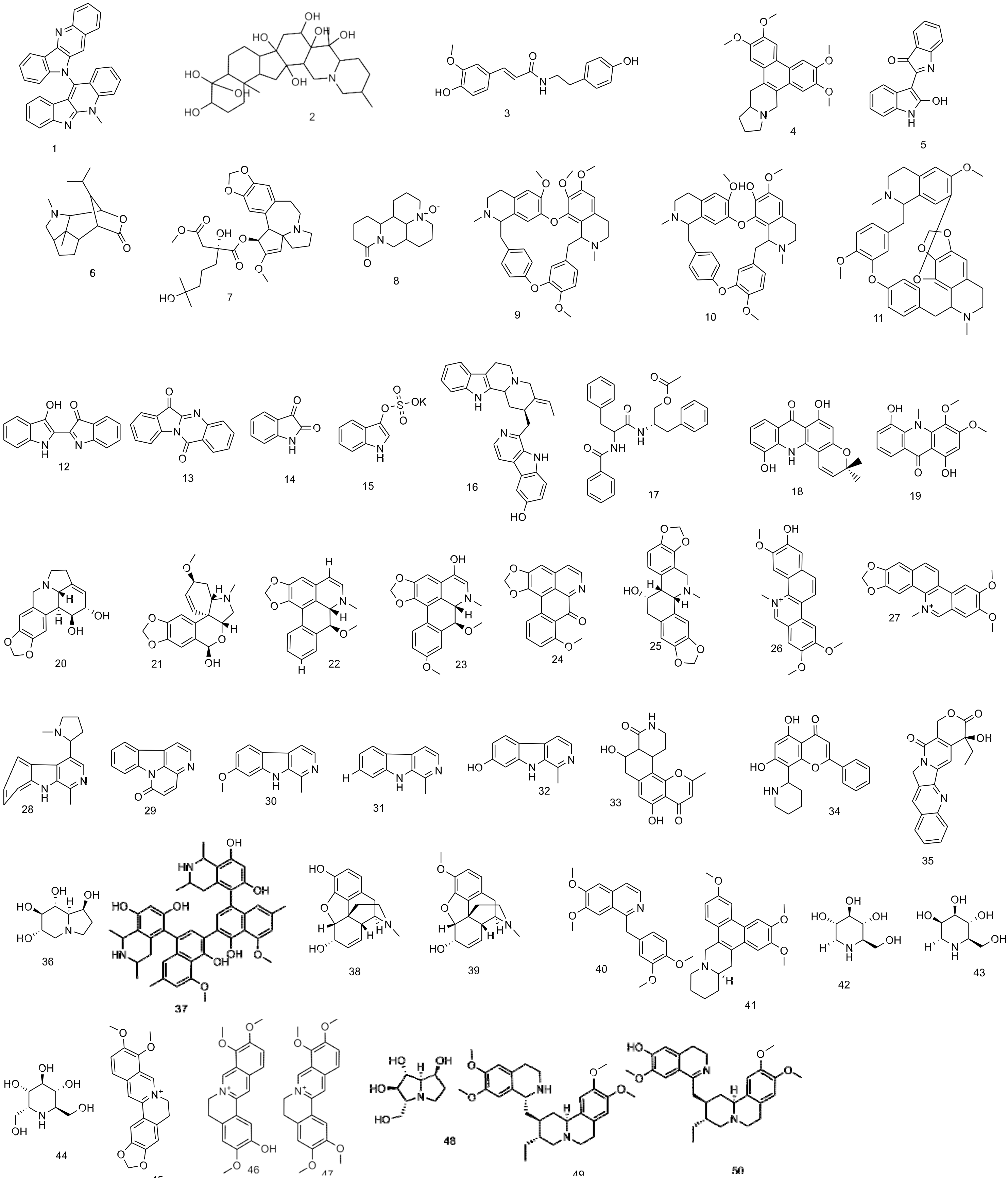
1
2
3
4
5
6
7
8
9
10
11
12
13
14
15
16
17
18
19
20
21
22
23
24
25
26
27
28
29
30
31
32
33
34
35
36
37
38
39
40
41
42
43
44
46
47
48
49
50

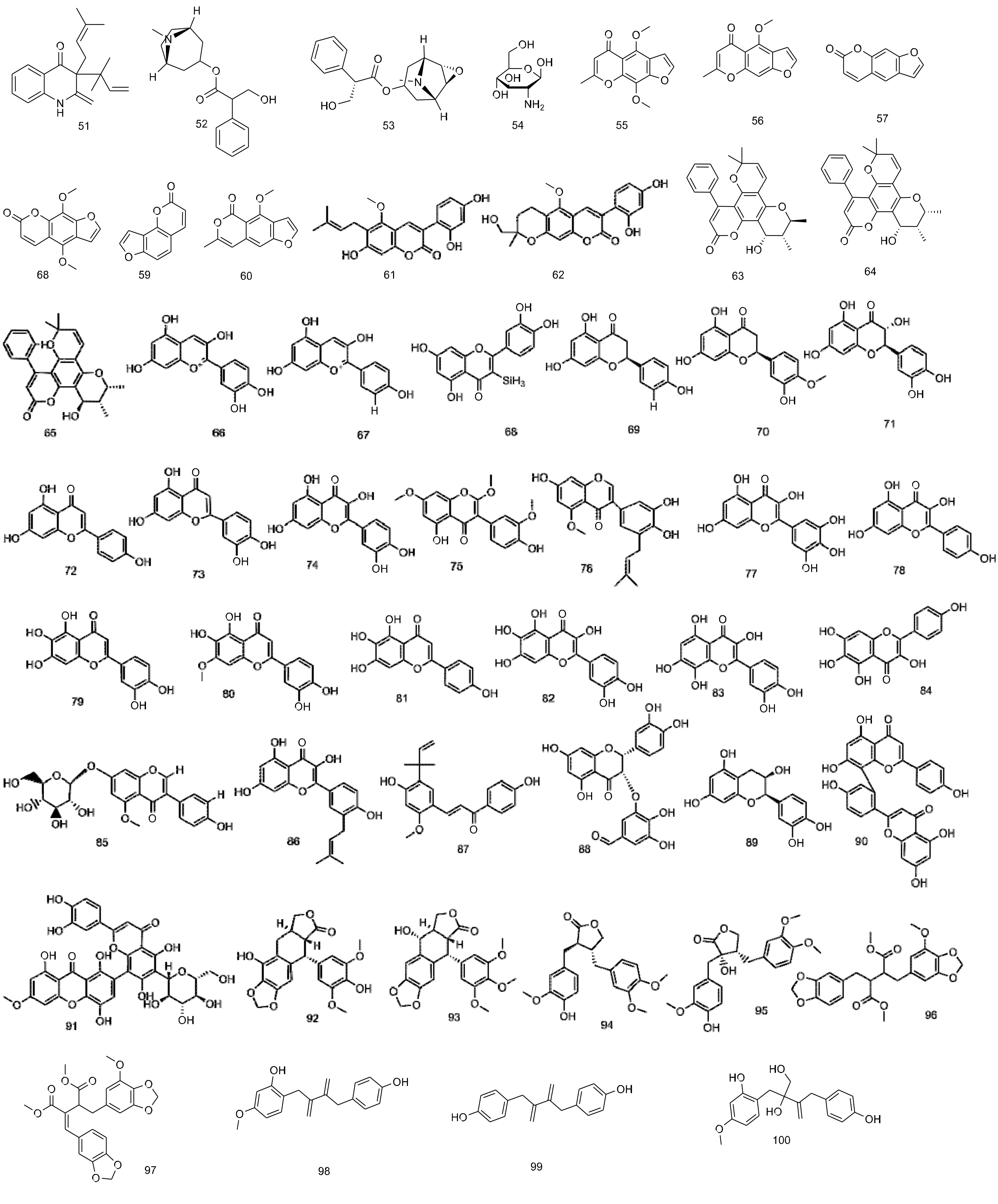
51
52
53
54
55
56
57
68
59
60
61
62
63
64
65
66
67
68
69
70
71
72
73
74
75
76
77
78
79
80
81
82
83
84
85
86
87
88
89
90
91
92
93
94
95
96
97
98
99
100

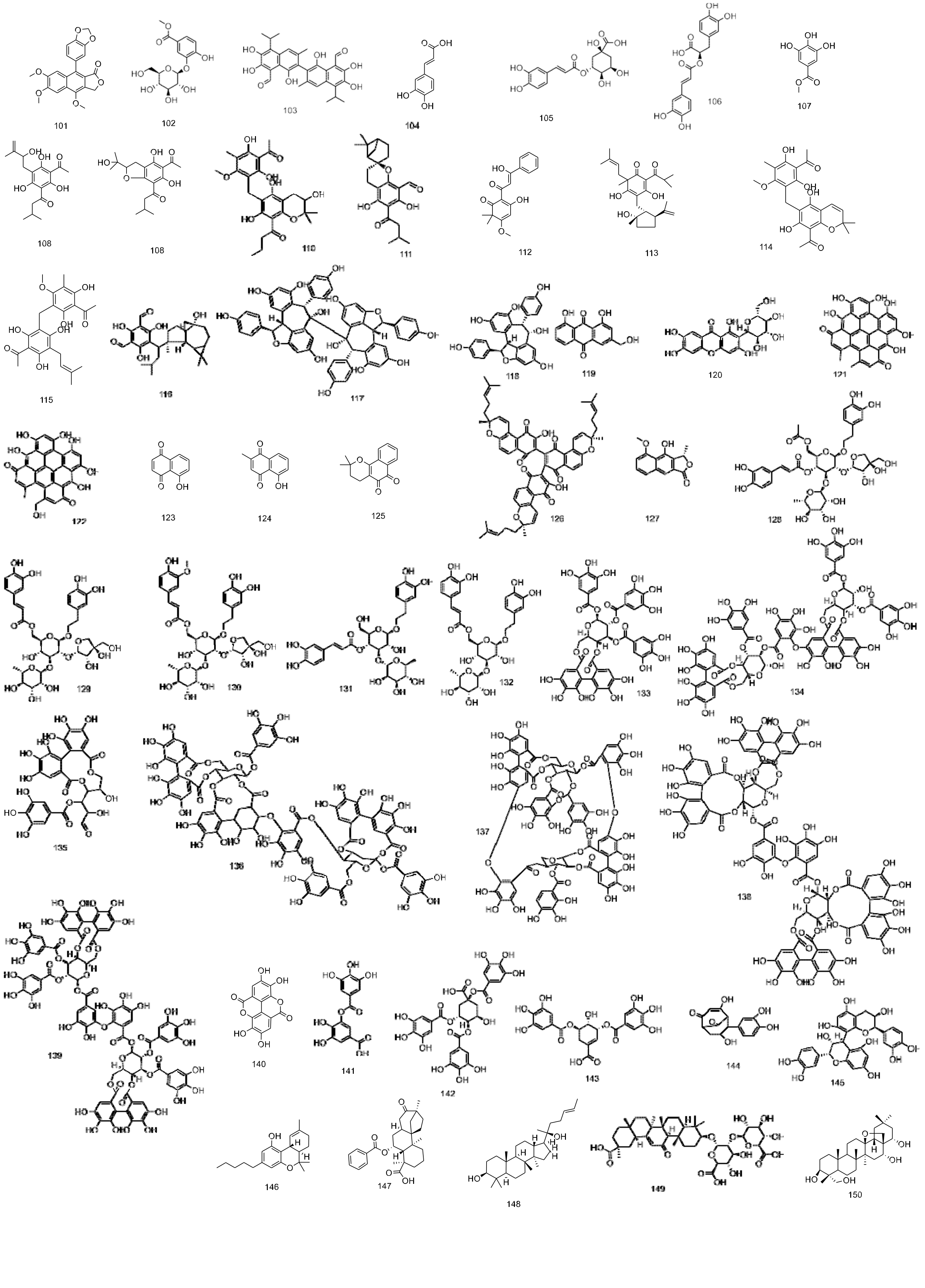
101
102
103
104
105
106
107
108
108
110
111
112
113
114
115
116
117
118
119
120
121
122
123
124
125
126
127
128
129
130
131
132
133
134
135
136
137
138
139
140
141
142
143
144
145
146
147
148
149
150

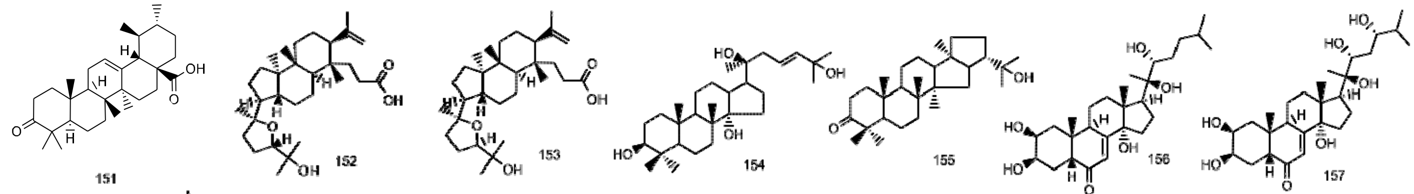
151
152
153
154
155
156
157

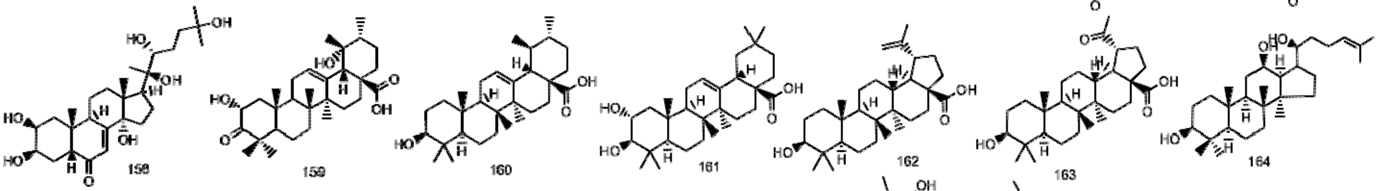
158
159
160
161
162
163
164

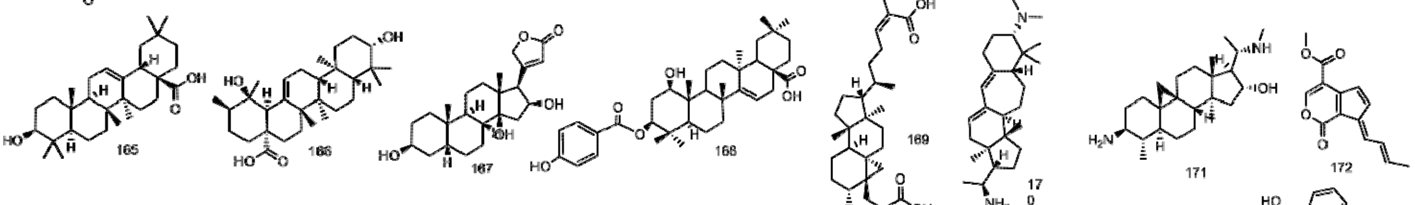
165
166
167
168
169
17
0
171
172

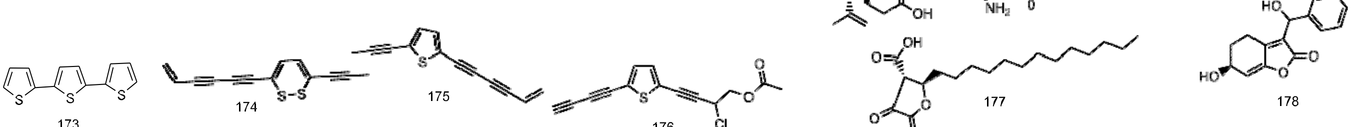
173
174
175
176
177
178

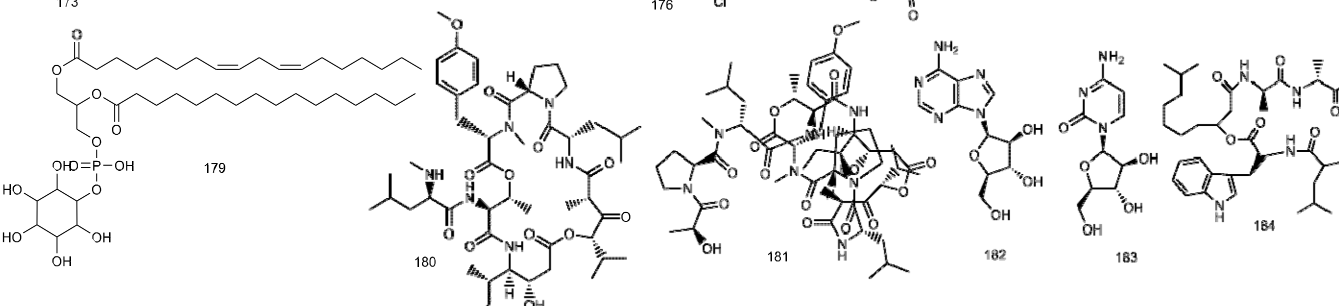
179
180
181
182
183
184

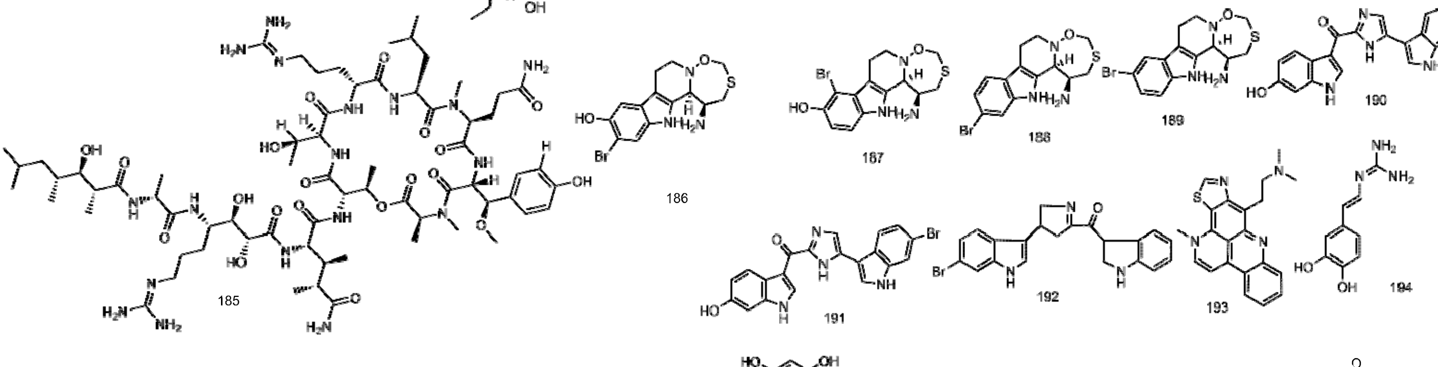
185
186
187
188
189
190
191
192
193
194

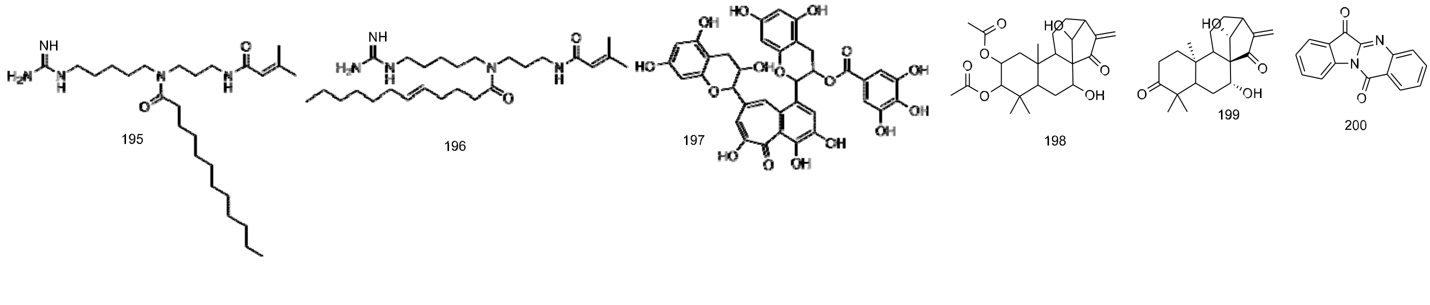
195
196
197
198
199
200

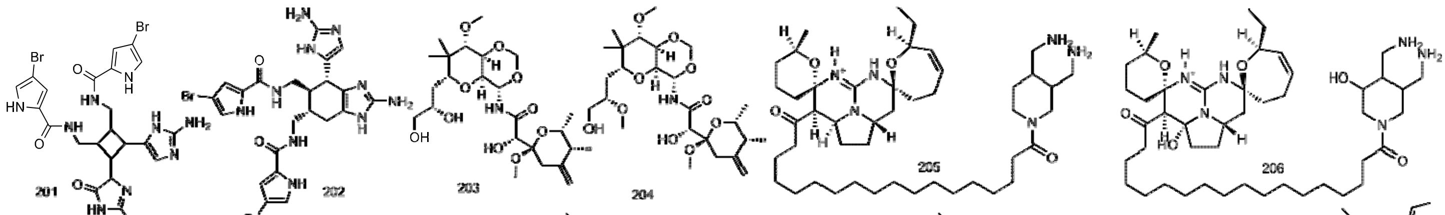

201 202 203 204 205 206

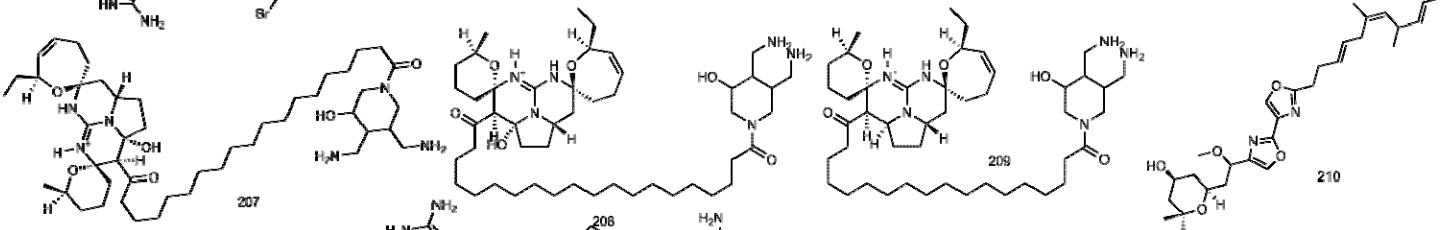

207 208 209 210

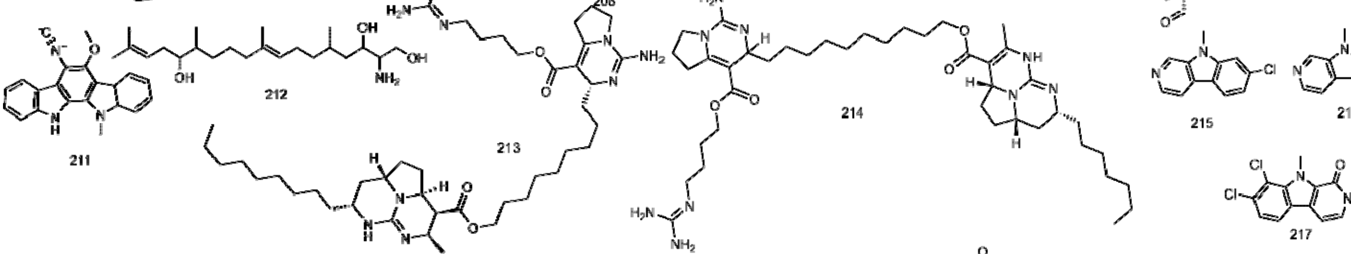

211 212 213 214 215 216 217

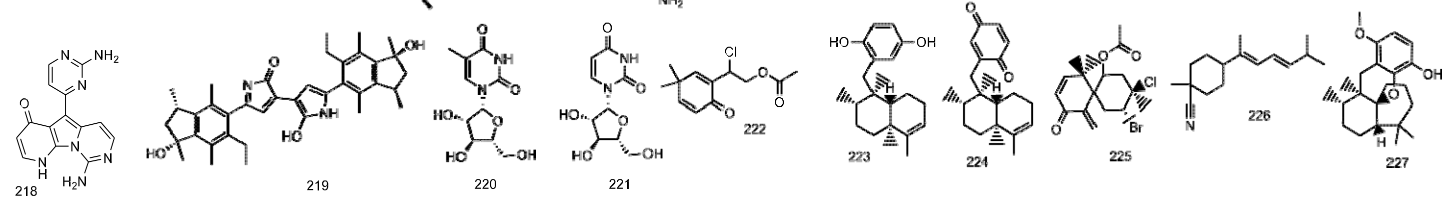

218 219 220 221 222 223 224 225 226 227

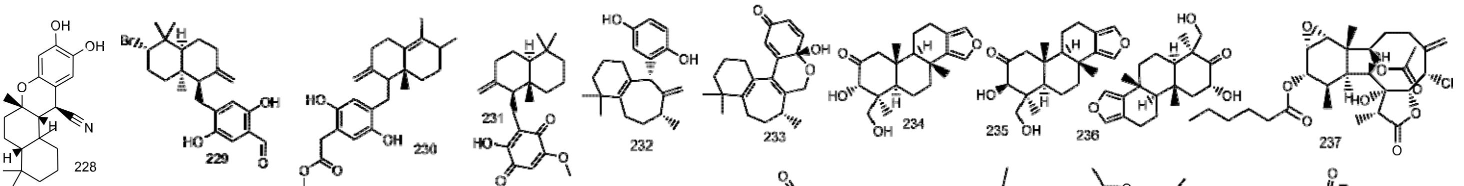

228 229 230 231 232 233 234 235 236 237

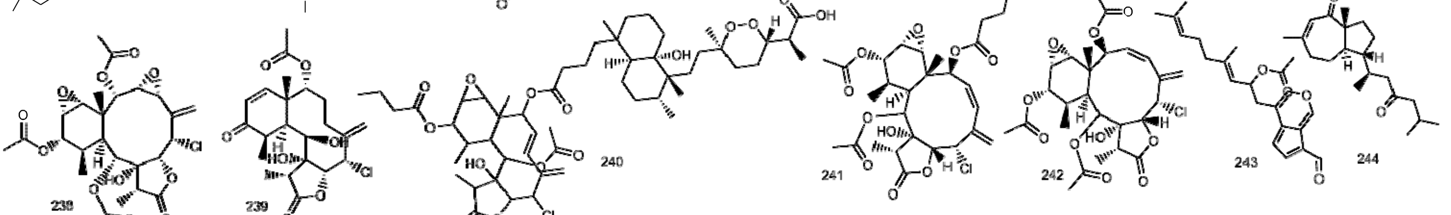

238 239 240 241 242 243 244

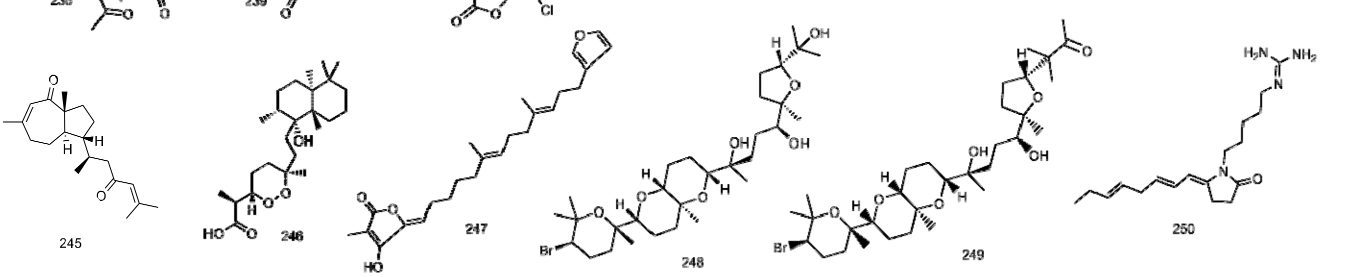

245 246 247 248 249 250

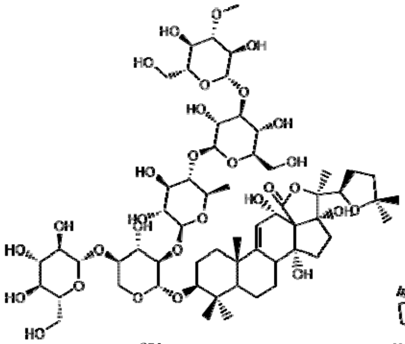

251

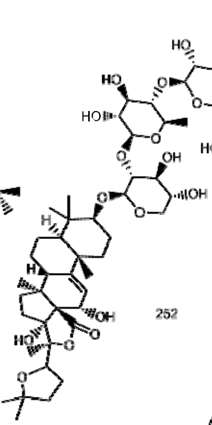

252

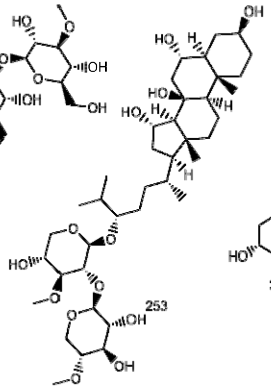

253

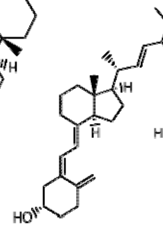

354

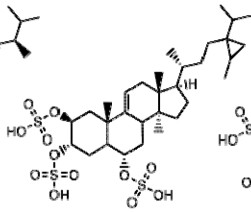

255

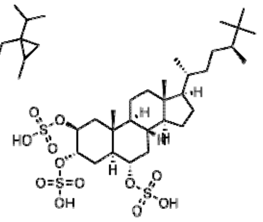

266

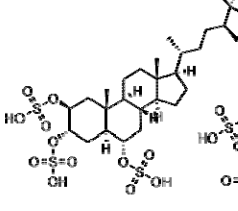

257

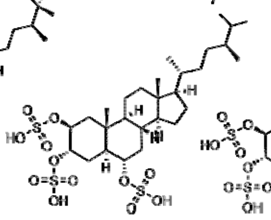

256

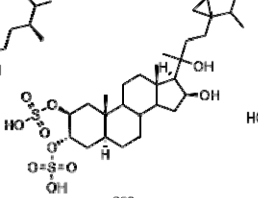

259

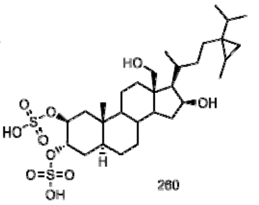

260

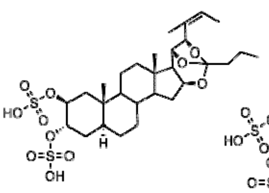

261

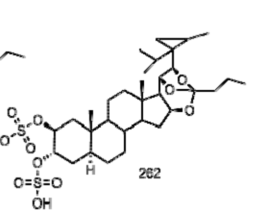

262

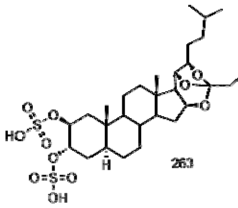

263

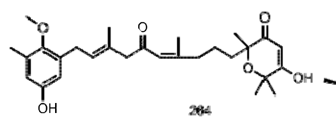

284

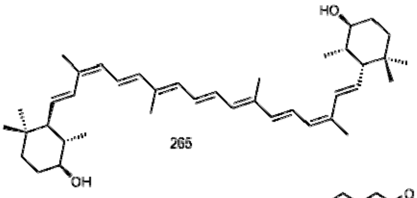

265

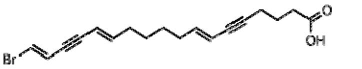

266

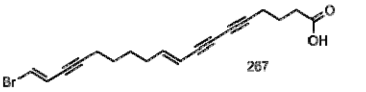

267

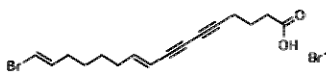

268

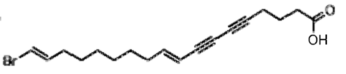

269

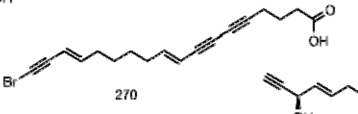

270

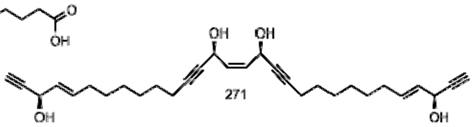

271

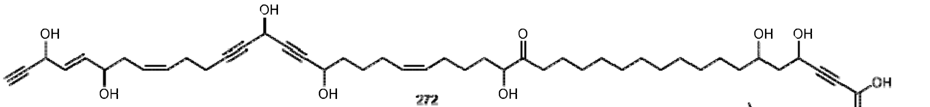

272

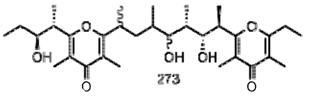

273

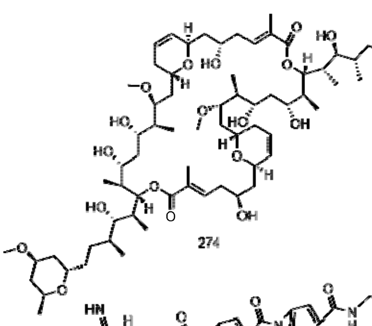

274

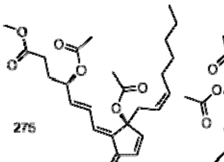

275

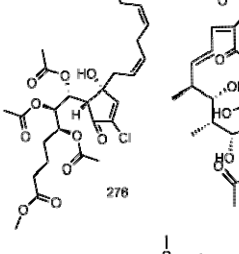

276

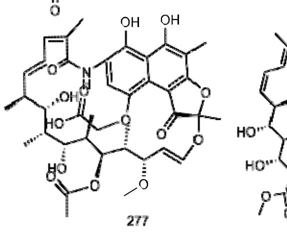

277

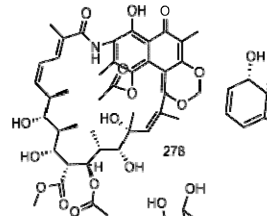

276

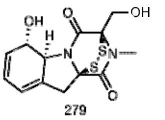

279

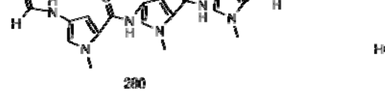

280

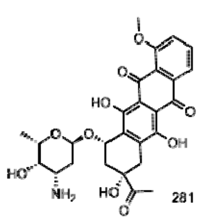

281

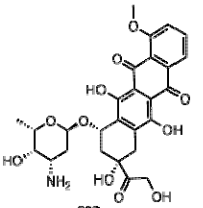

282

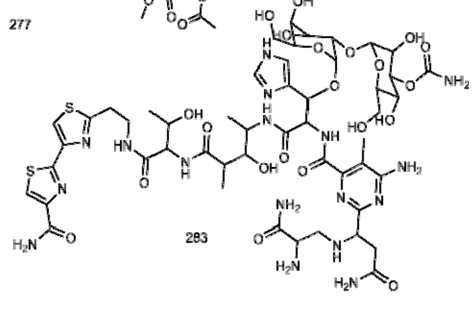

283

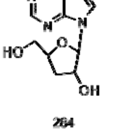

264

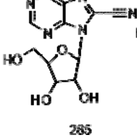

285

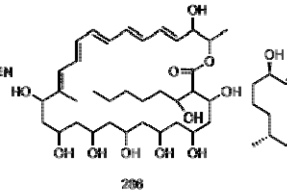

286

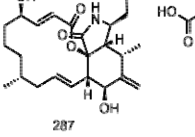

287

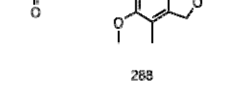

288

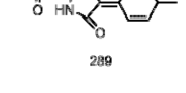

289

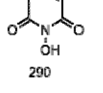

290

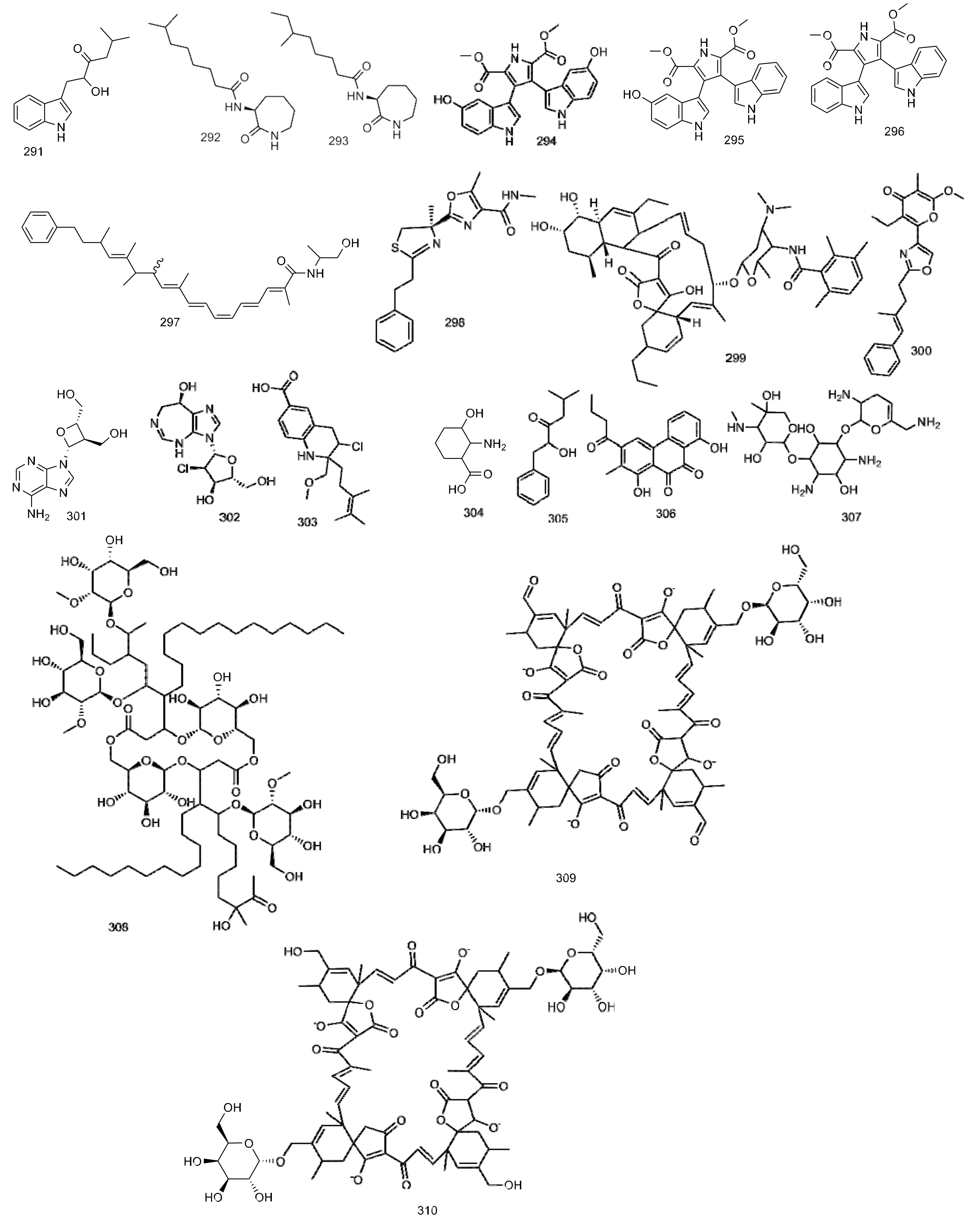

Figure S2. Chemical structures of the examined 310 natural antiviral compounds.

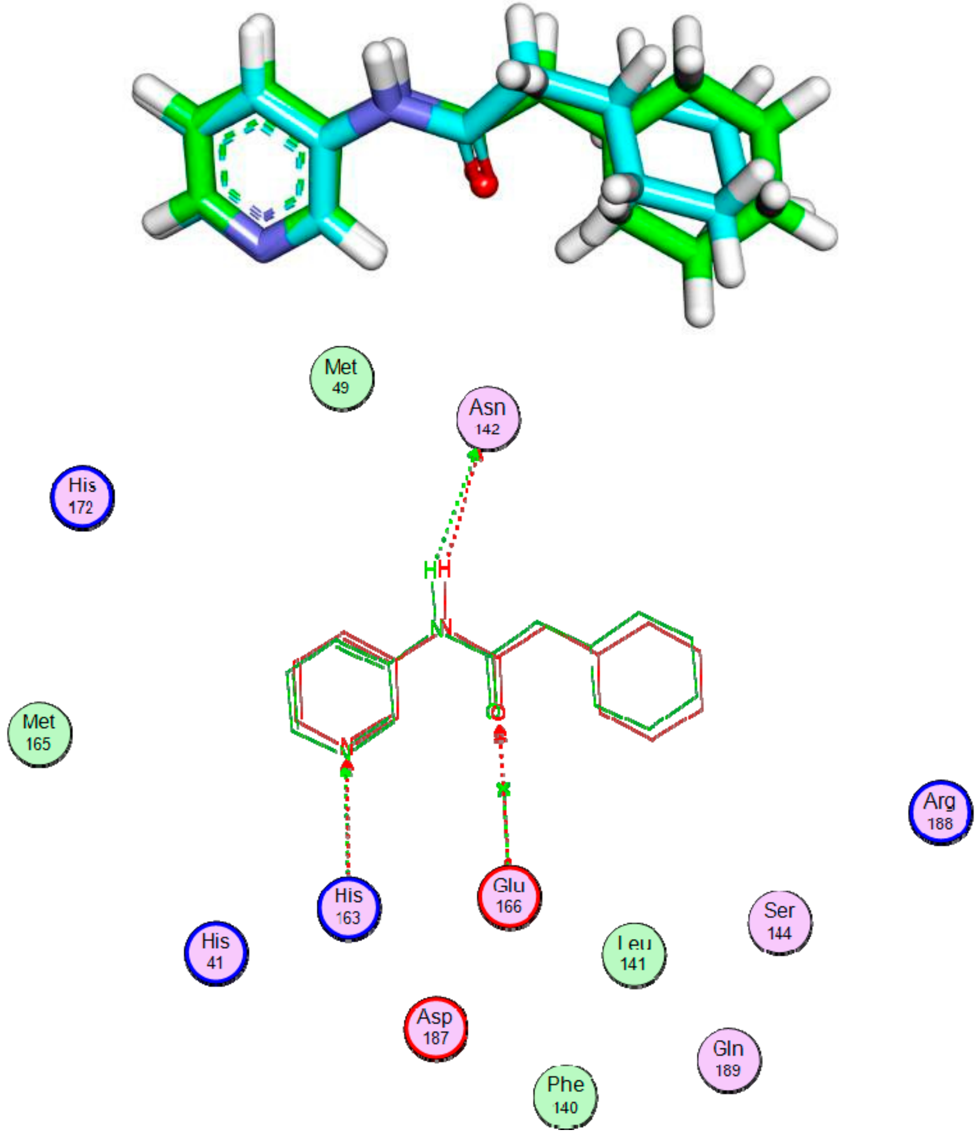

Figure S3. Superimposition of the re-docked conformer of the co-crystallized ligand over the original one with an RMSD value of 0.73 °A.

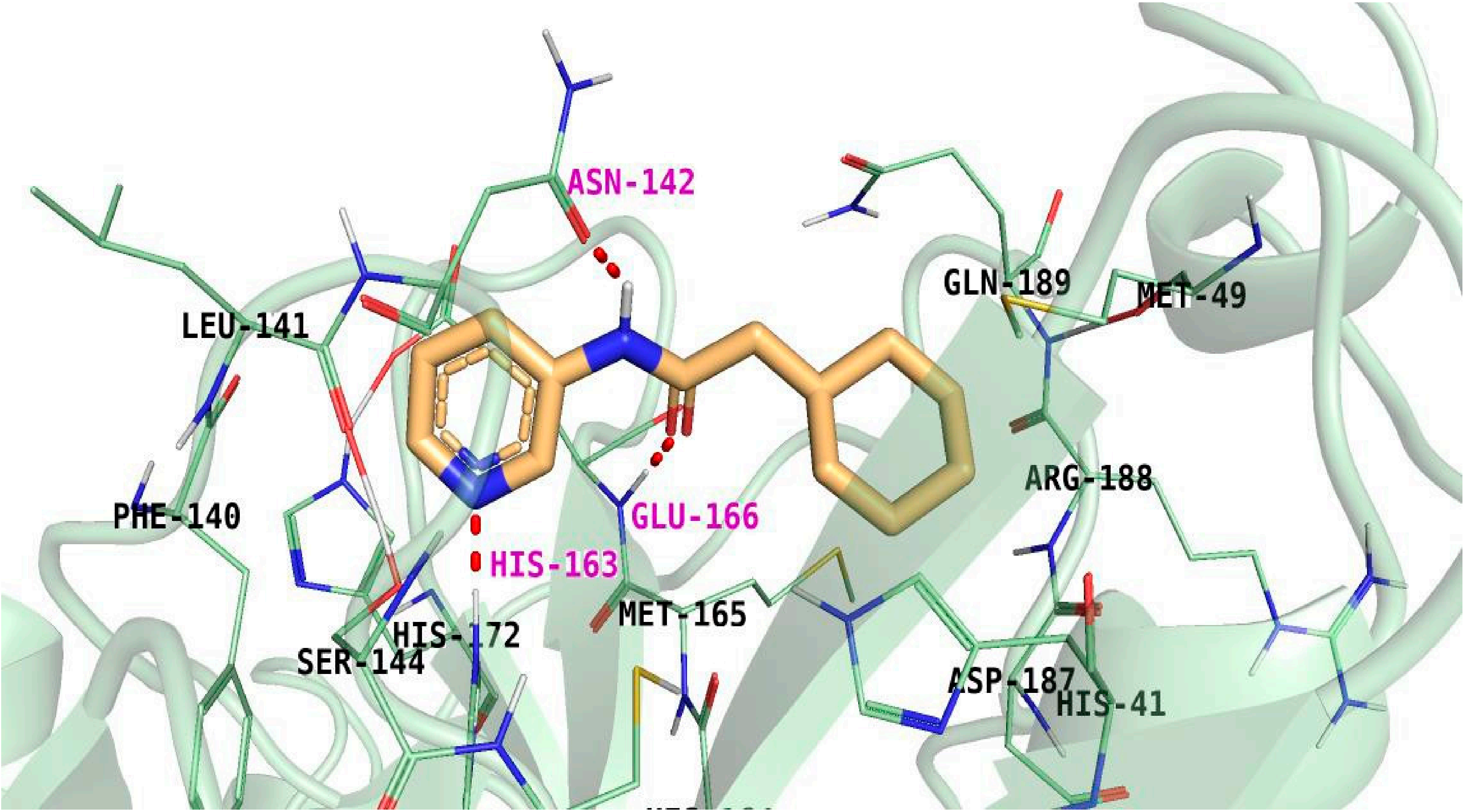

Figure S4. 3D of **GWS** in the active site of M^pro.

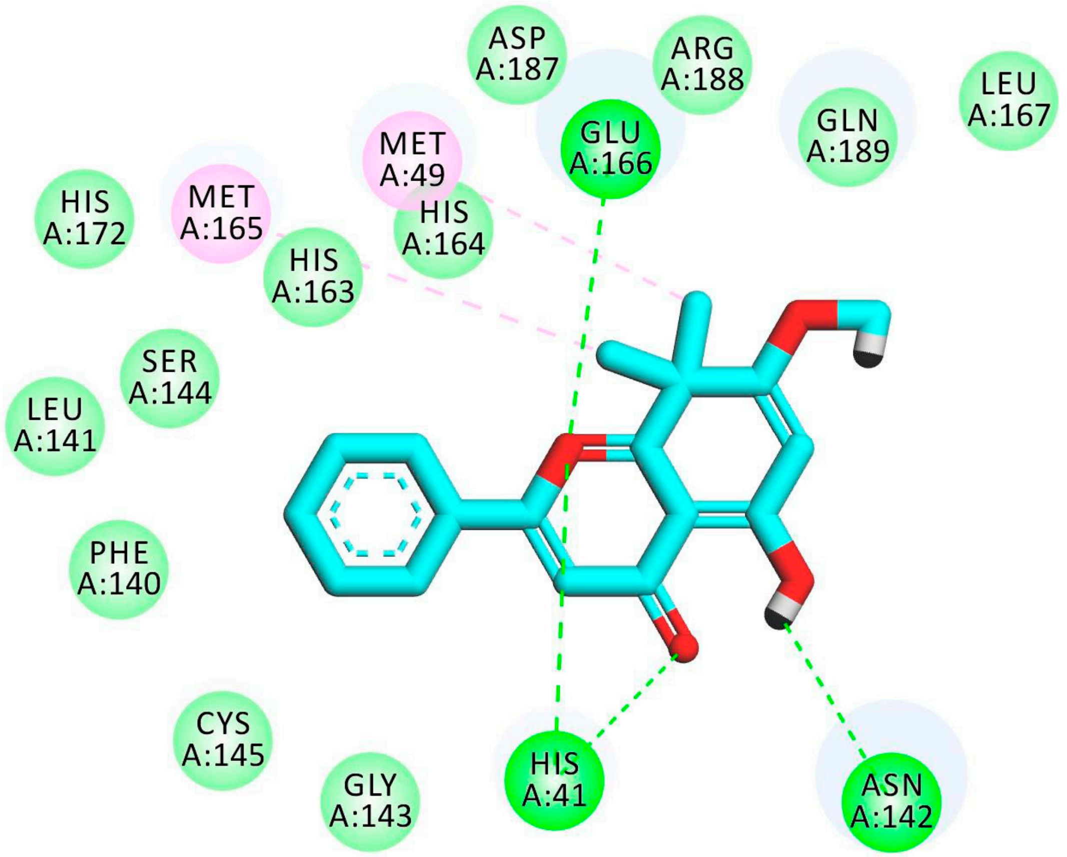

Figure S5. 2D of **112** in the active site of M$^{pro.}$

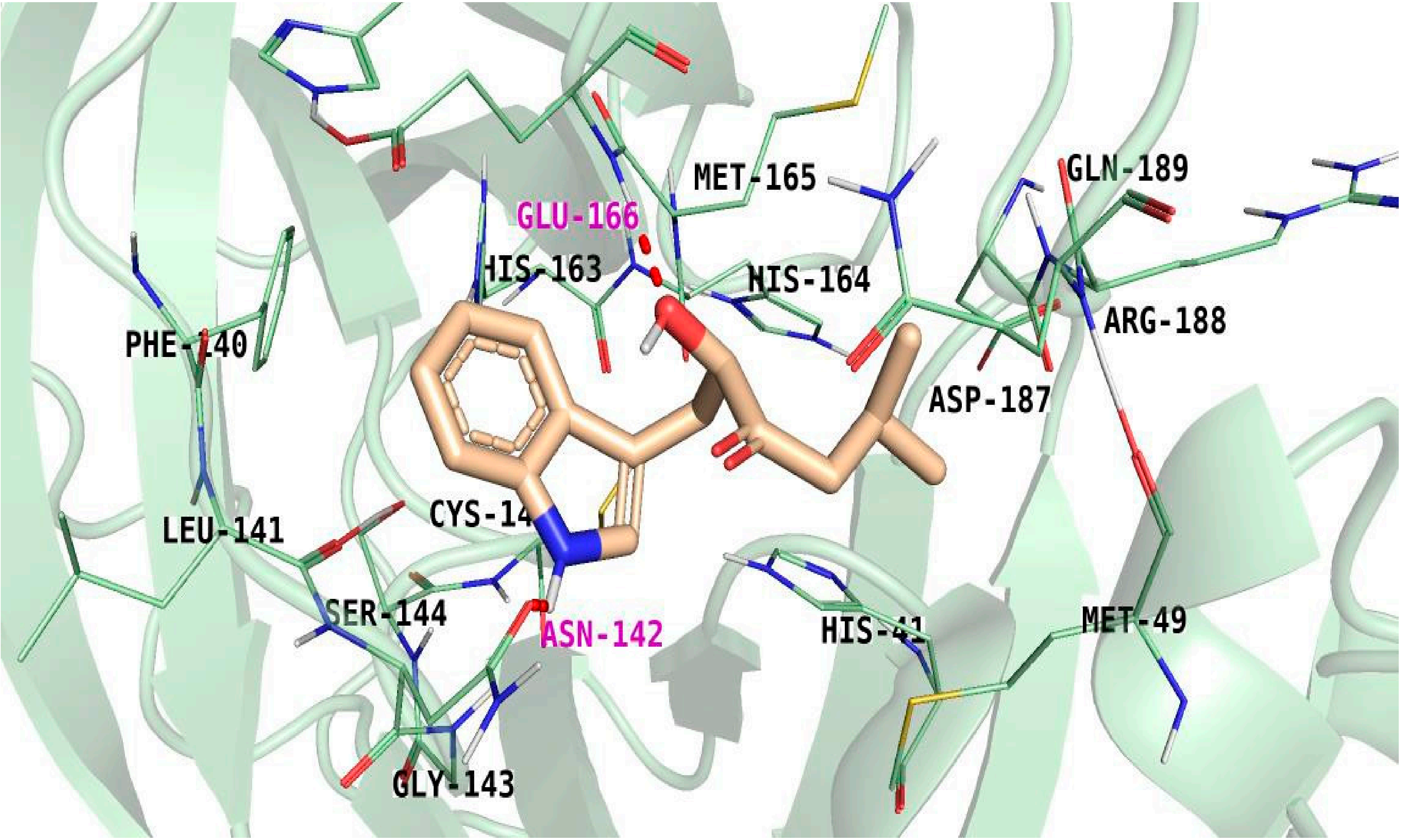

Figure S6. 3D of **291** in the active site of M^pro.

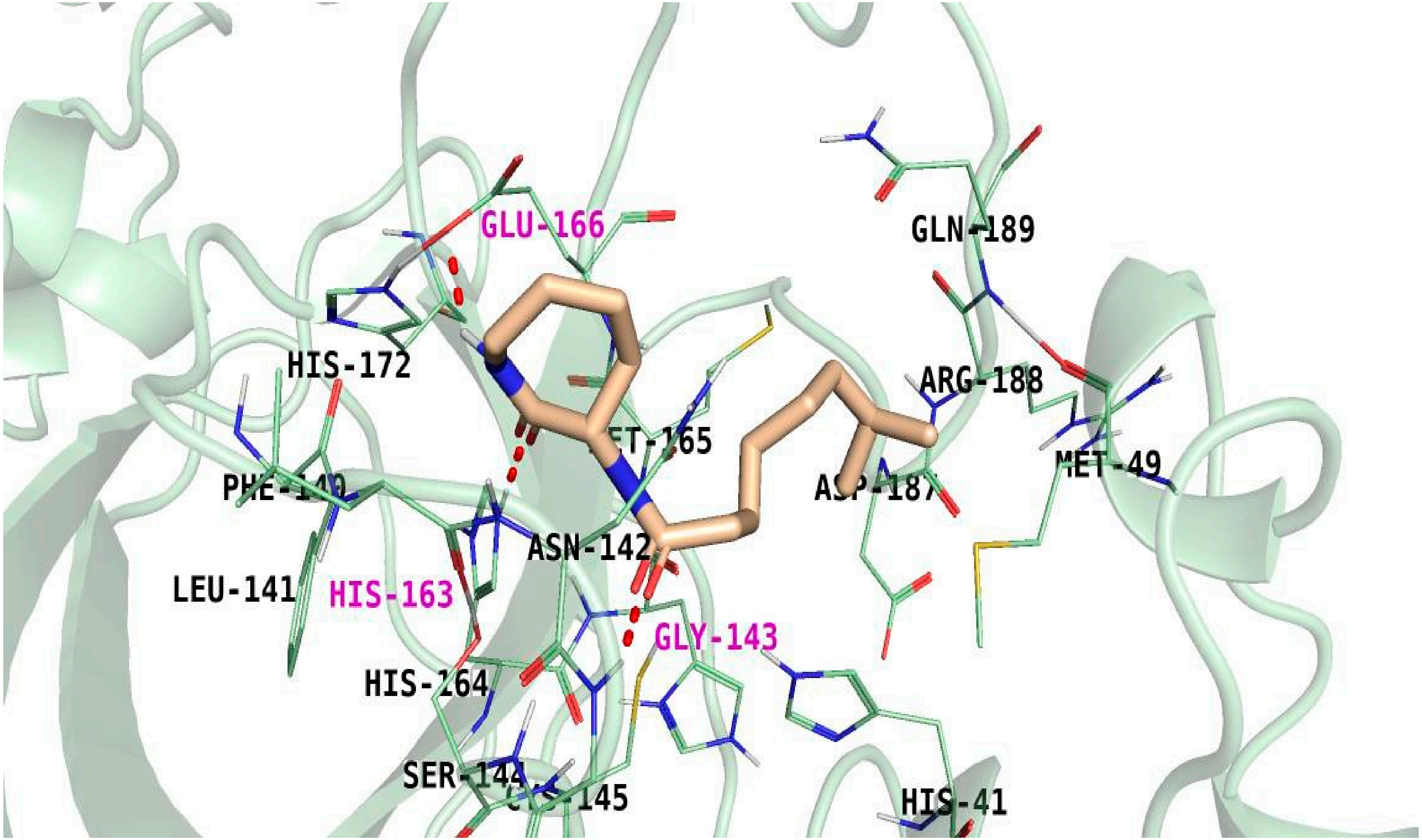

Figure S7. 3D of **292** in the active of site $M^{pro.}$

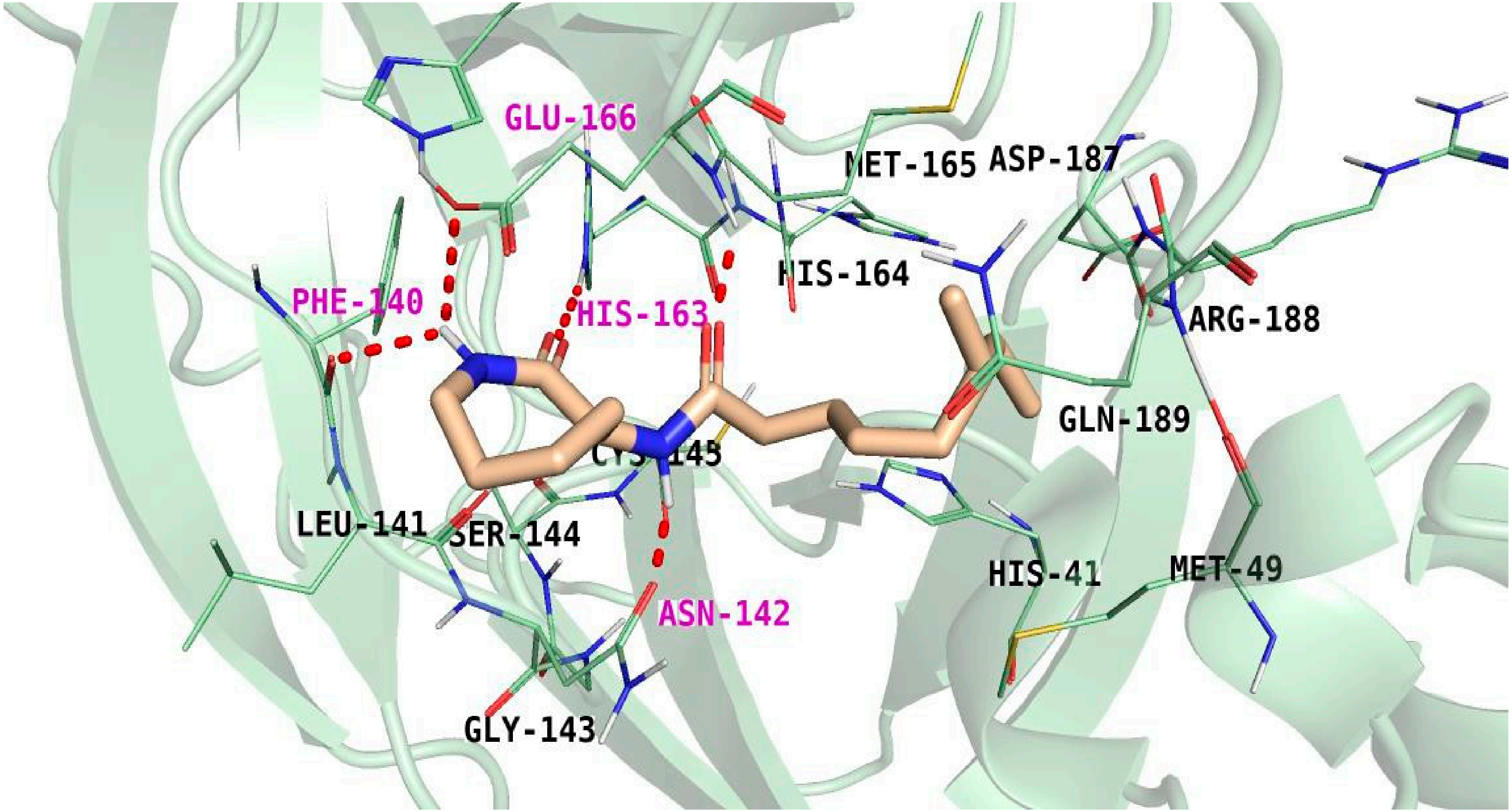

Figure S8. 3D of **293** in the active site of M[pro.]

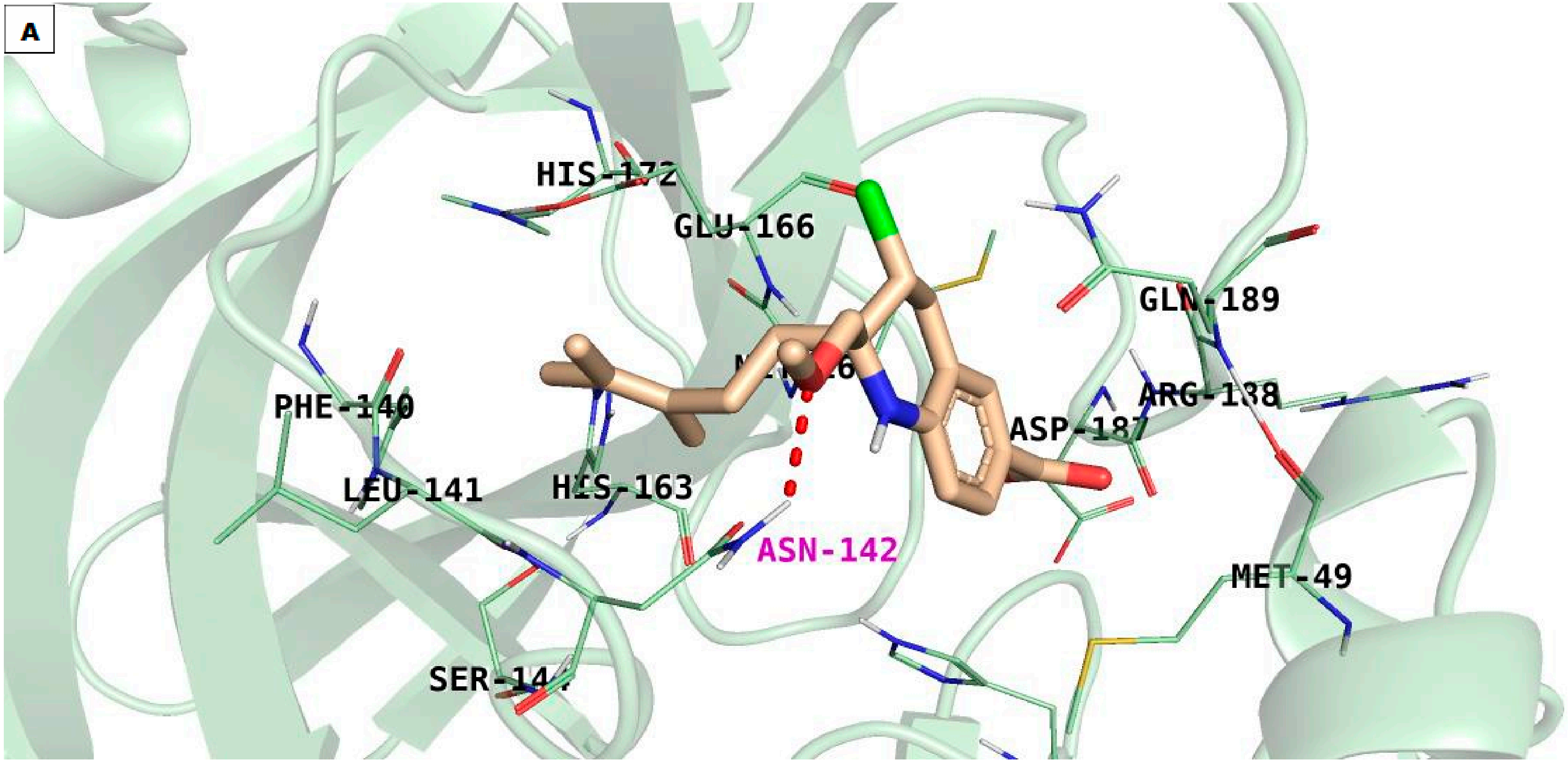

Figure S9A. 3D of **303** in the active site M$^{pro.}$

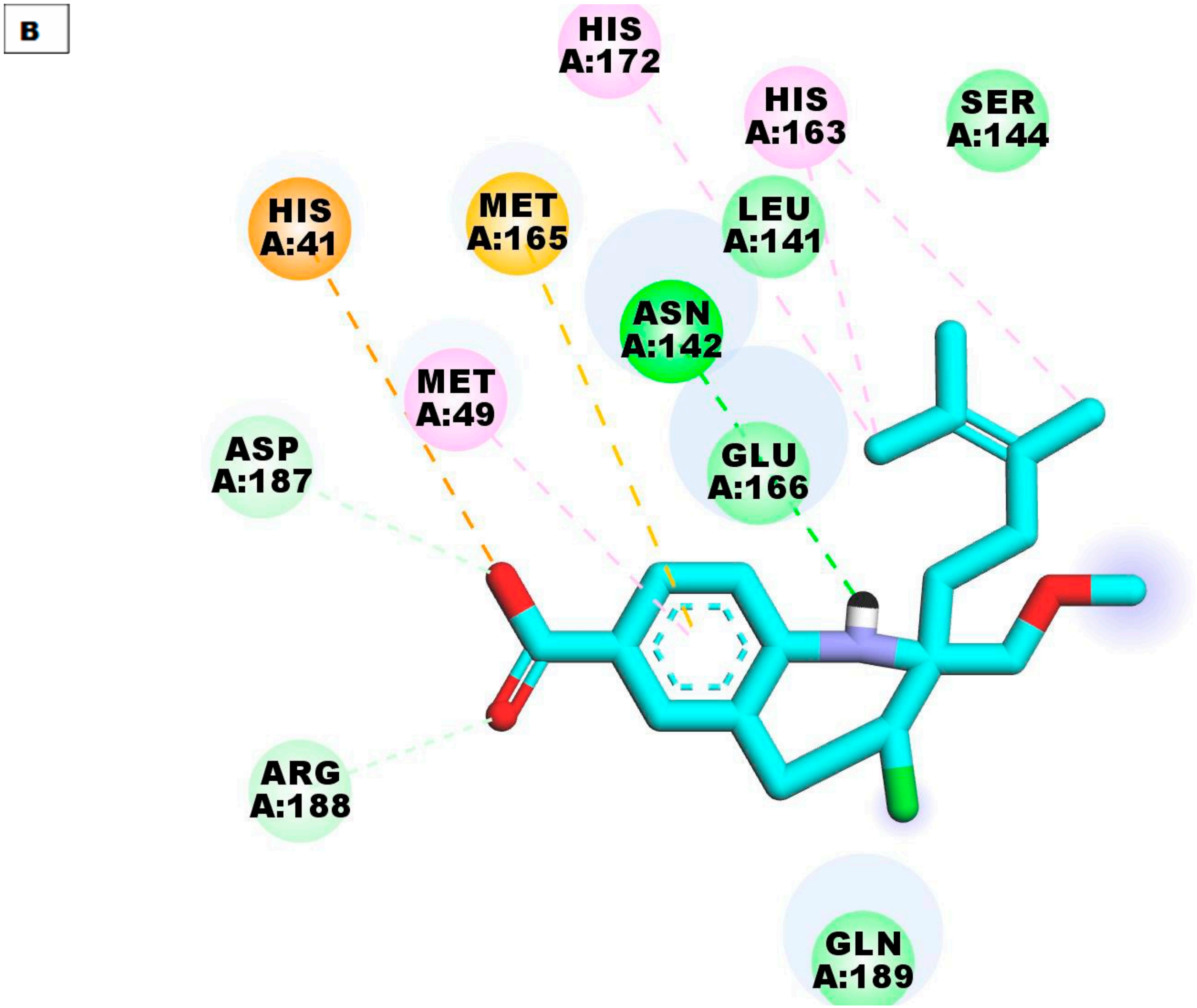

Figure S9B. 2D of **303** in the active site M$^{pro.}$

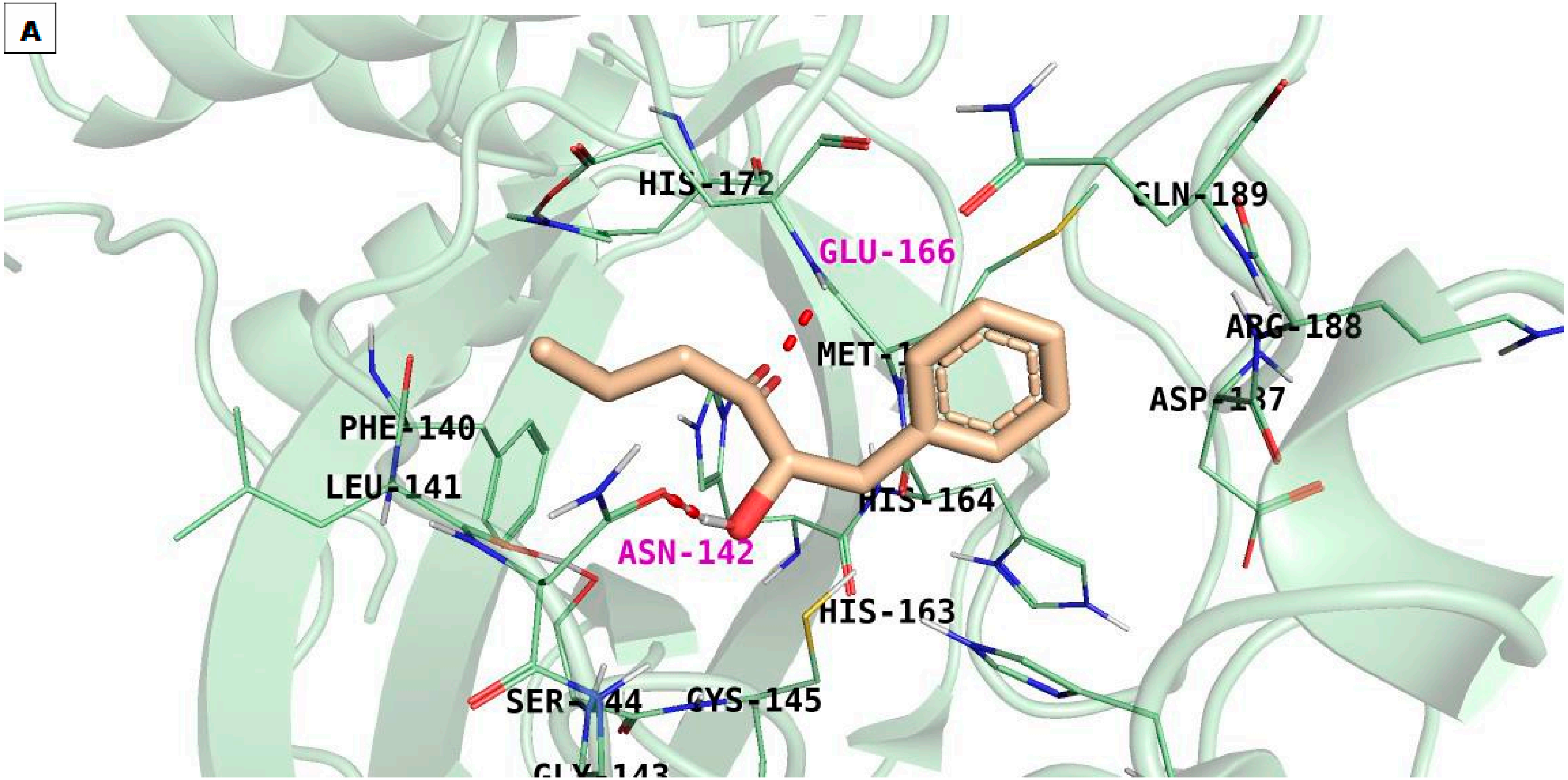

Figure S10 A. 3D of **305** in the active site M[pro.]

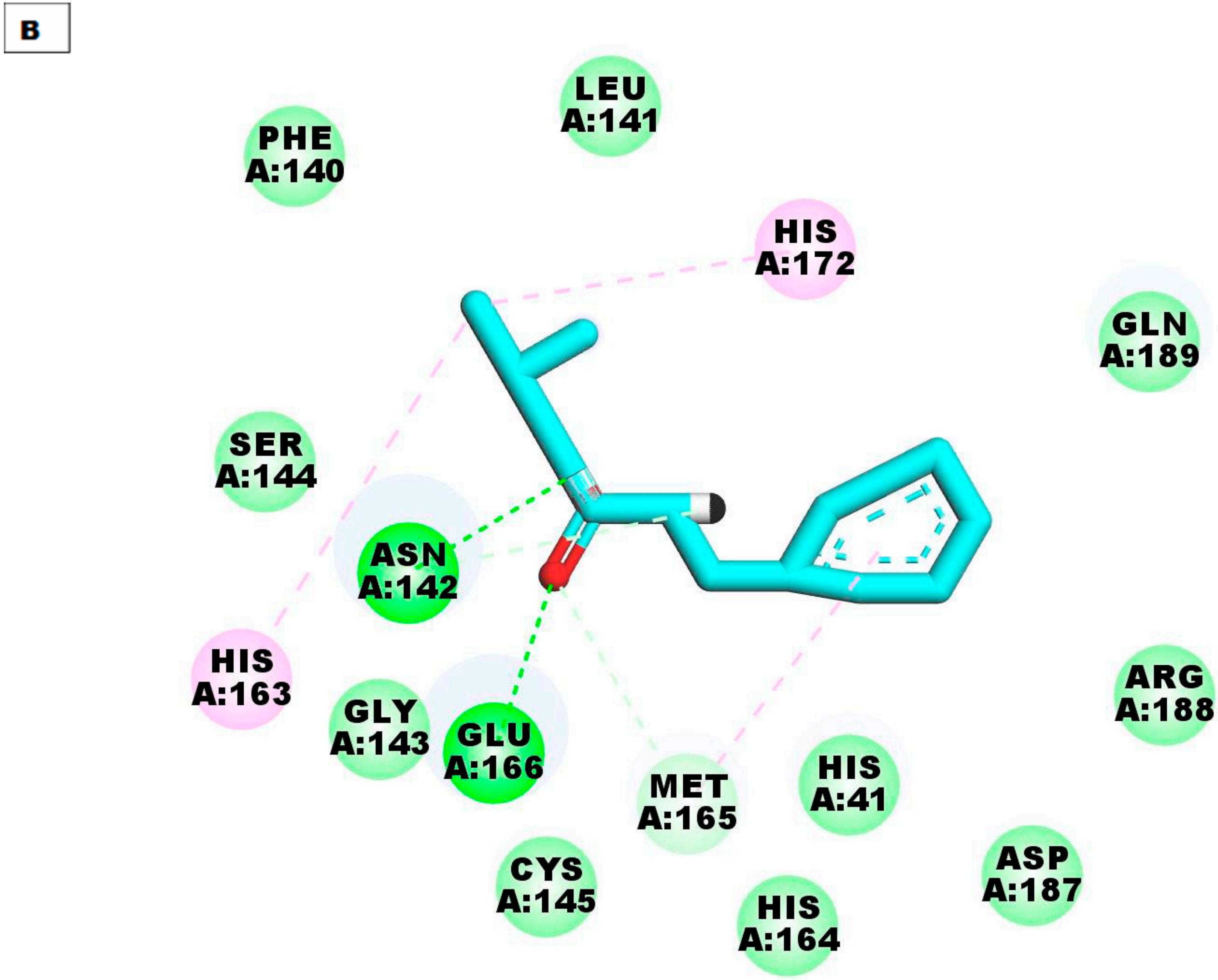

Figure S10 B. 2D of **305** in the active site M$^{pro.}$

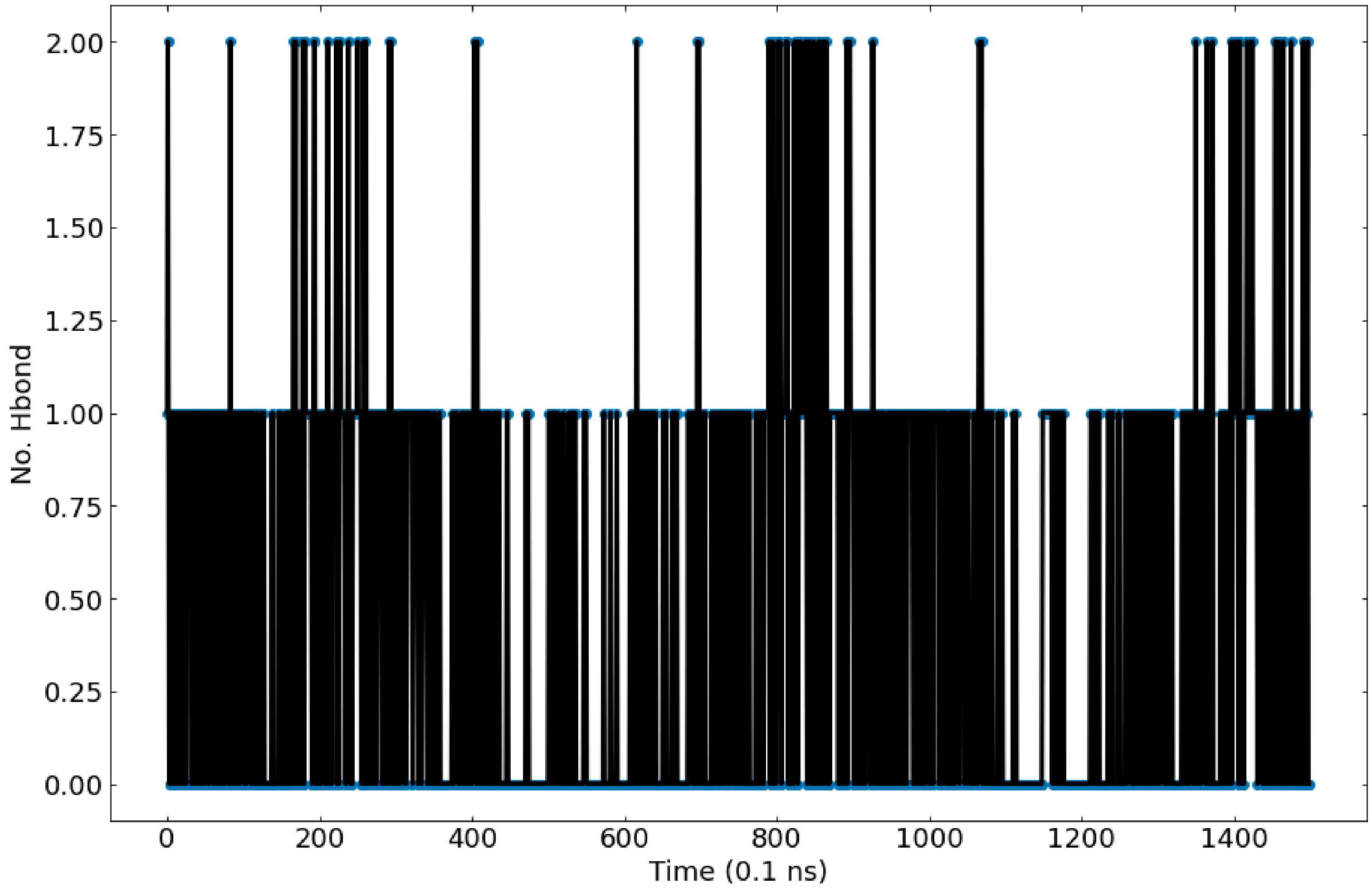

Figure S11. Hydrogen number plot that formed between caprolactin A-M^{pro} complex.

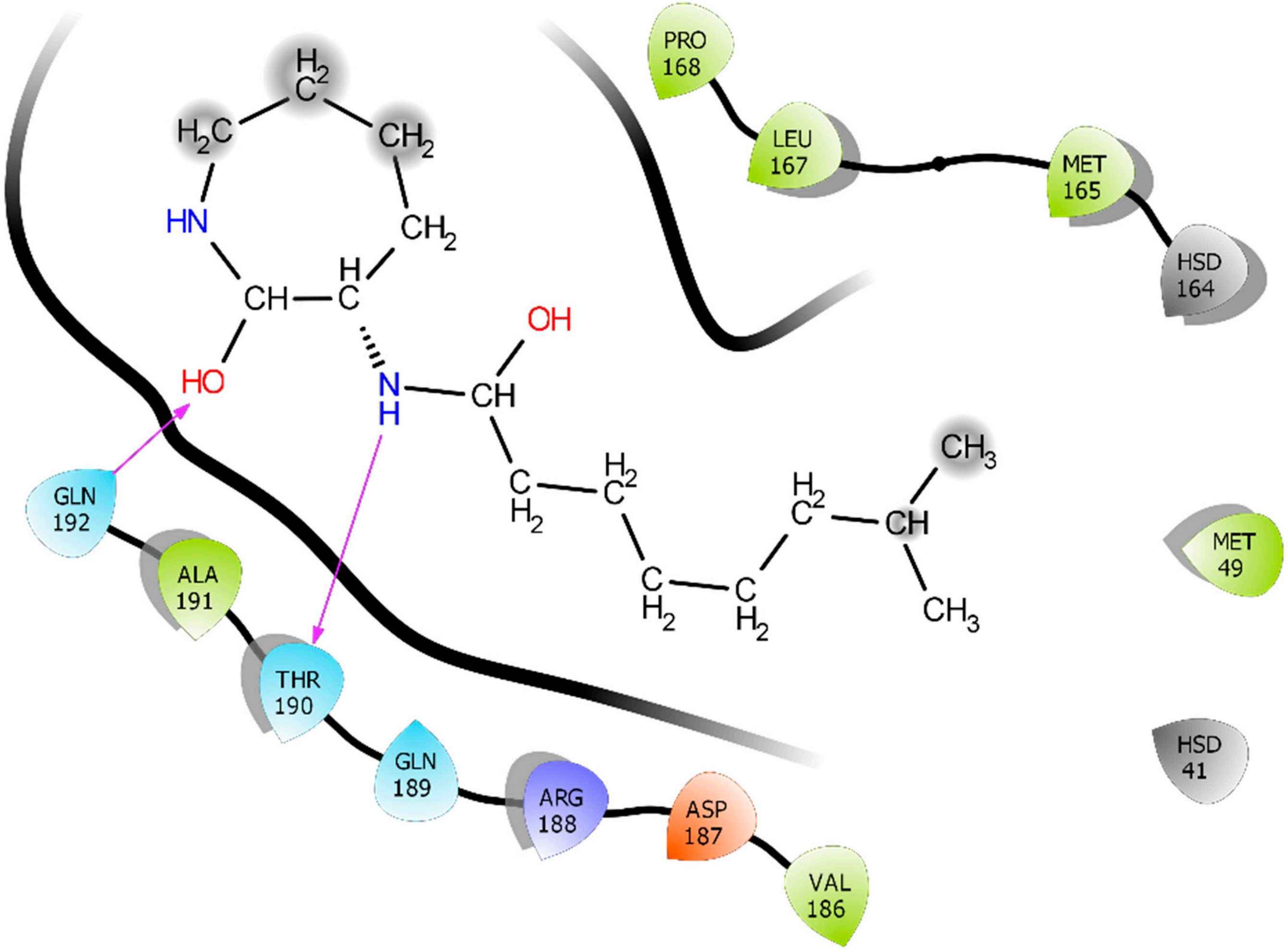
PRO
168
LEU
167
MET
165
HSD
164
GLN
192
ALA
191
THR
190
GLN
189
ARG
188
ASP
187
VAL
186
MET
49
HSD
41
HN
HO
NH
OH
CH
CH
CH3
CH3

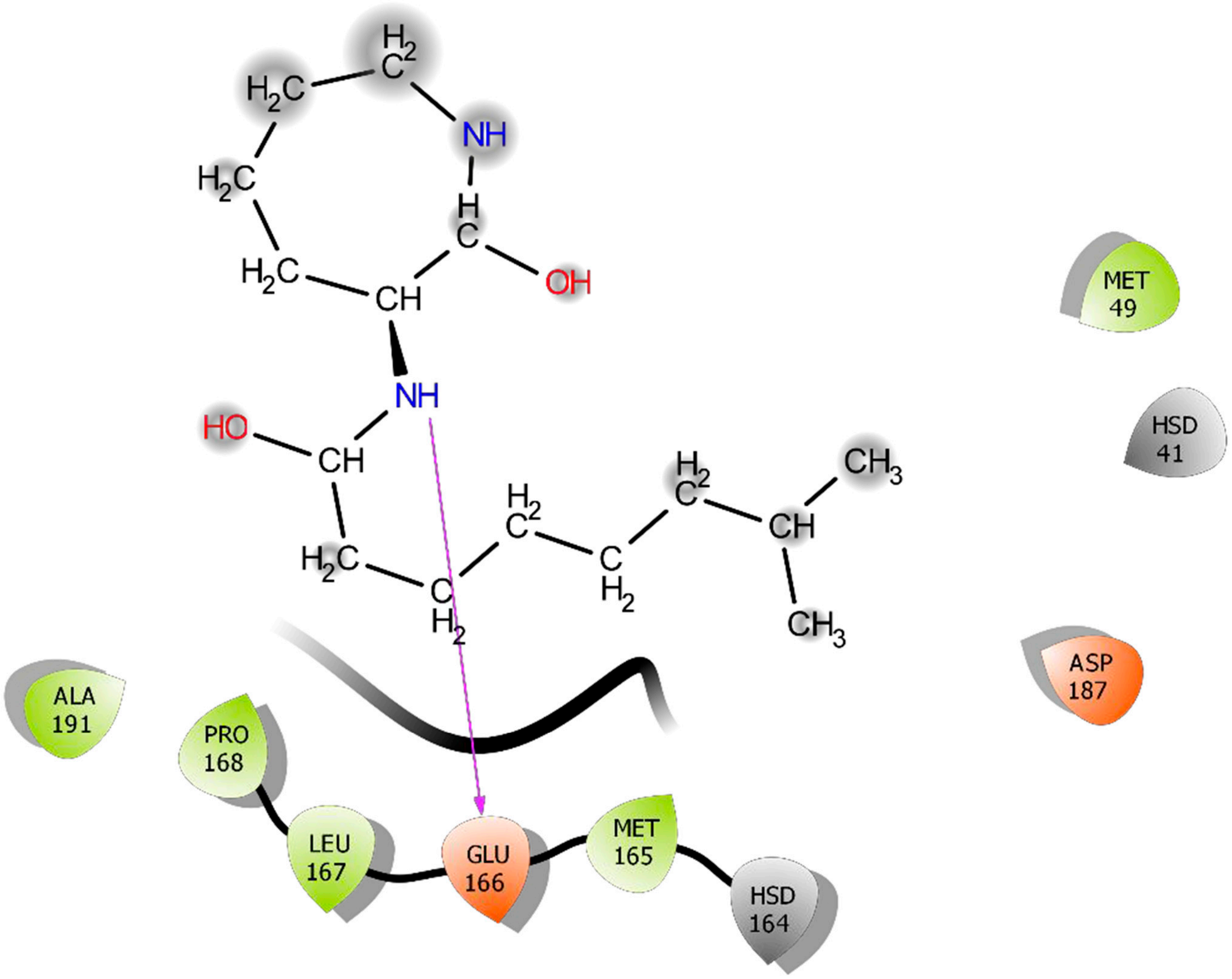

Figure S12. Representative poses of caprolactin A in the M^{pro} pocket. Hydrogen bond interactions purple dashed line

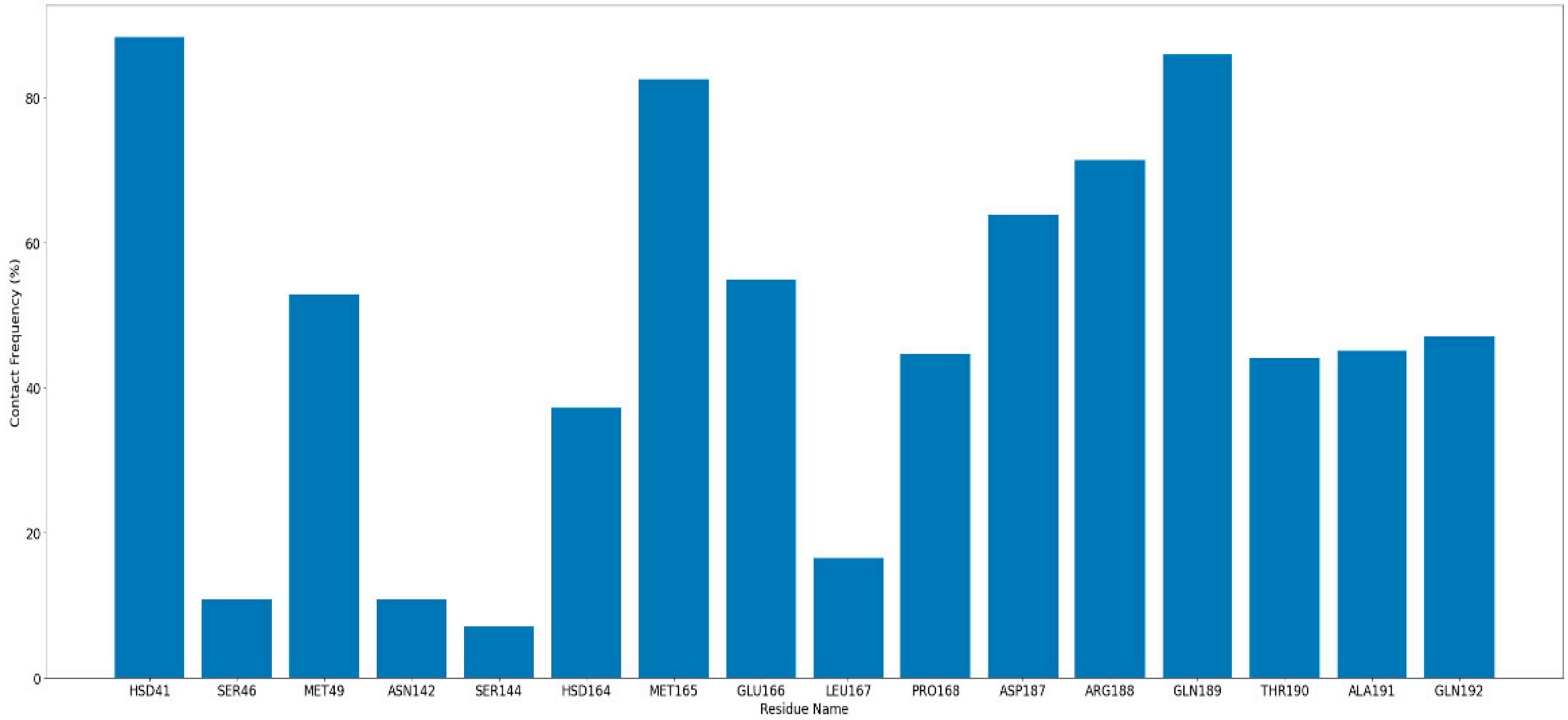

Figure S13. Contact frequency plot of main protease residues bonded with caprolactin A.

Table S1. Molecular properties of metabolites having structural similarity with **GWS.**

Comp.	ALog p[a]	M. Wt[b]	HBA[c]	HBD[d]	R- b	Ri	Ar- ri	MFPSA[e]	Minimum Distance
**6**	0.827	264.383	2	1	1	4	0	0.113	0.614
**14**	0.67	147.131	2	1	0	2	1	0.339	0.676
**15**	1.579	251.301	4	1	3	2	2	0.292	0.560
**30**	2.438	212.247	2	1	1	3	3	0.171	0.561
**31**	2.455	182.221	1	1	0	3	3	0.154	0.682
**51**	4.791	295.419	2	1	4	2	1	0.083	0.731
**52**	0.16	290.377	3	2	5	3	1	0.172	0.727
**55**	2.172	260.242	4	0	2	3	2	0.222	0.779
**56**	2.189	230.216	3	0	1	3	2	0.216	0.770
**60**	2.175	230.216	3	0	1	3	2	0.216	0.770
**109**	2.33	336.38	6	3	5	2	1	0.285	0.354
**112**	2.161	314.333	5	2	4	2	1	0.249	0.228
**124**	1.962	188.179	3	1	0	2	1	0.292	0.369
**125**	2.509	242.27	3	0	0	3	1	0.176	0.255
**127**	2.466	244.243	4	1	1	3	2	0.235	0.273
**172**	1.597	244.243	4	0	3	2	0	0.208	0.353
**174**	4.343	228.333	2	0	4	1	0	0.206	0.503
**175**	4.883	196.268	0	0	4	1	1	0.126	0.544
**176**	4.851	276.738	2	0	6	1	1	0.193	0.512
**178**	1.436	258.269	4	2	2	3	1	0.274	0.448
**217**	2.846	267.111	1	1	0	3	2	0.142	0.472
**222**	2.154	242.699	3	0	4	1	0	0.164	0.451
**234**	2.24	332.434	3	2	1	4	1	0.197	0.559
**235**	2.24	332.434	3	2	1	4	1	0.197	0.559
**236**	2.526	332.434	3	2	1	4	1	0.198	0.562
**291**	2.932	245.317	2	2	5	2	2	0.198	0.311
**292**	2.685	268.395	2	2	7	1	0	0.186	0.362
**293**	2.685	268.395	2	2	7	1	0	0.186	0.362
**303**	3.006	350.86	4	1	6	2	1	0.158	0.231
**305**	2.639	206.281	2	1	5	1	1	0.156	0.176
**GWS**	2.171	218.295	2	1	3	2	1	0.179	0.000

[a] Partition coefficient; [b] Molecular weight; [c] H- bond doners; [d] H- bond acceptors; [e] Molecular fractional polar surface area.

Method

Method

1- Molecular Similarity

Molecular Similarity of the 310 natural compounds against the co-crystallized ligand (GWS) of SARS-Cov-2 main protease (**PDB ID: 5R84)** was carried out calculated using Discovery studio 4.0. At first, the CHARMM force field was applied then the compounds were prepared using prepare ligand protocol. Then, the 310 compounds were used as a test set while the co-crystallized ligand (GWS) was used as a reference compounds. The protocol was adjusted to give 10% output. The default molecular properties were applied. The molecular properties include number of rotatable bonds, number of rings, number of aromatic rings, number of hydrogen bond donors (HBA), number of hydrogen bond acceptors (HBD), partition coefficient (ALog p), molecular weight (M. Wt), and molecular fractional polar surface area (MFPSA).

2- Pharmacophore

Pharmacophore model was carried out using Discovery Studio 4.0 software. The protocol of receptor-ligand pharmacophore generation was applied. In this protocol, the co-crystallized ligand of SARS-Cov-2 main protease (**PDB ID: 5R84**) was used as a reference molecule. The tested compounds were used as a training set. In this protocol, we used the following features in pharmacophore generation; i) hydrogen bond donor (HBD), ii) hydrogen bond acceptor (HBA), iii) hydrophobic aliphatic (HA), iii) hydrophobic aromatic (HAr), and ring aromatic (RA). Then, ligand pharmacophore mapping protocol was used in virtual screening process. The most predictive model was used as 3D queries to identify compounds with high fit value [1-3].

.

3- Docking studies

Crystal structure of SARS-Cov-2 main protease [(**PDB ID: 5R84**) , resolution: 1.83Å] was obtained from Protein Data Bank. The docking investigation was accomplished using MOE2014 software. At first, the crystal structure of SARS-Cov-2 main protease was prepared by removing water molecules. Only one chain was retained beside the co-crystallized ligand (GWS). Then, the selected chain was protonated and subjected to minimization of energy process. Next, the active site of the target protein was defined. The x-y-z coordinates of the

active site of the Mpro enzyme was 9.813136 -3.822304 23.394054. Structures of the tested compounds and the co-crystallized ligand were drawn using ChemBioDraw Ultra 14.0 and saved as MDL-SD format. Such file was opened using MOE to display the 3D structures which were protonated and subjected to energy minimization. Formerly, validation of the docking process was performed by docking the co-crystallized ligand against the isolated pocket of active site. The produced RMSD value indicated the validity of process. Finally, docking of the tested compounds was done through the dock option inserted in compute window. For each docked molecule, 30 docked poses were produced using ASE for scoring function and force field for refinement. The results of the docking process were then visualized using Discovery Studio 4.0 software.

4- ADMET studies

ADMET descriptors (absorption, distribution, metabolism, excretion and toxicity) of the compounds were determined using Discovery studio 4.0. At first, the CHARMM force field was applied then the tested compounds were prepared and minimized according to the preparation of small molecule protocol. Then ADMET descriptors protocol was applied to carry out these studies.

5- Toxicity studies

The toxicity parameters of the tested compounds were calculated using Discovery studio 4.0. Indinavir was used as a reference drug. At first, the CHARMM force field was applied then the compounds were prepared and minimized according to the preparation of small molecule protocol. Then different parameters were calculated from the toxicity prediction (extensible) protocol.

6- DFT studies:

The DFT parameters (total energy, binding energy, HOMO, LUMO, gap energy, dipole moment, and electrostatic potential) were calculated using Discovery studio software. the tested compounds were prepared using prepare ligand protocol. Then, the prepared compounds were subjected to DFT calculation protocol using the default option. The functional used in this test was PWC of local density approximation (LDA). In addi-tion, the quality was adjusted to be Coarse which uses DN basis set with SCF density

convergence of 1.0e-4 as utilized from Accelrys in the DMol3 module of Materials Studio package.

7- MD simulatons

Among the tested compounds, the most promising one 292 was advanced to MD simulations to study the relative stability of the protein–ligand interactions. All the simulations were done using the NAMD 2.13 package, and the CHARMM36 force field. The parameters for the top docking results were generated using the CHARMM general force field (CgenFF). The TIP3P explicit solvation model was used, and the periodic boundary conditions were set with a dimension of the dimensions 95.56 Å, 95.63 Å, and 95.55 Å in x, y, and z, respectively. Afterward, the system was neutralized using 4 (Na^+) ions. The MD protocols involved minimization, annealing, equilibration, and production. A 2 fs time step of integration was chosen for all MD simulations, the equilibration was carried in the canonical (NVT) ensemble, while the isothermal–isobaric (NPT) ensemble was for the production. Through the 100 ns of MD production, the pressure was set at 1 atm using the Nose´–Hoover Langevin piston barostat with a Langevin piston decay of 0.05 ps and a period of 0.1 ps. The temperature was set at 298.15 K using the Langevin thermostat. A distance cutoff of 12.0 Å was applied to short-range nonbonded interactions with a pair list distance of 16 Å, and Lennard Jones interactions were smoothly truncated at 8.0 Å. Long-range electrostatic interactions were treated using the particle-mesh Ewald (PME) method,[8, 9] where a grid spacing of 1.0 Å was used for all simulation cells. All covalent bonds involving hydrogen atoms were constrained using the SHAKE algorithm For consistency, we have applied the same protocol for all MD simulations.

Toxicity Report

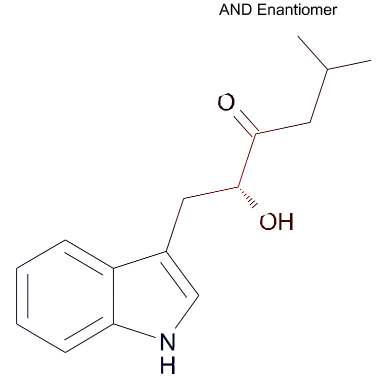

$C_{15}H_{19}NO_2$
Molecular Weight: 245.31685
ALogP: 2.932
Rotatable Bonds: 5
Acceptors: 2
Donors: 2

Model Prediction

Prediction: Toxic
Probability: 0.548
Enrichment: 1.042
Bayesian Score: 0.008
Mahalanobis Distance: 10.273
Mahalanobis Distance p-value: 0.0101

Prediction: Positive if the Bayesian score is above the estimated best cutoff value from minimizing the false positive and false negative rate.
Probability: The esimated probability that the sample is in the positive category. This assumes that the Bayesian score follows a normal distribution and is different from the prediction using a cutoff.
Enrichment: An estimate of enrichment, that is, the increased likelihood (versus random) of this sample being in the category.
Bayesian Score: The standard Laplacian-modified Bayesian score.
Mahalanobis Distance: The Mahalanobis distance (MD) is the distance to the center of the training data. The larger the MD, the less trustworthy the prediction.
Mahalanobis Distance p-value: The p-value gives the fraction of training data with an MD greater than or equal to the one for the given sample, assuming normally distributed data. The smaller the p-value, the less trustworthy the prediciton. For highly non-normal X properties (e.g., fingerprints), the MD p-value is wildly inaccurate.

Structural Similar Compounds

Name	Parbendazole	Bufexamac	Flufenamic Acid
Structure	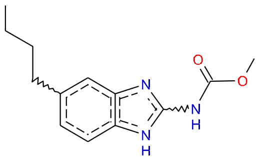	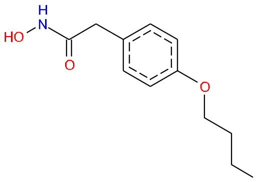	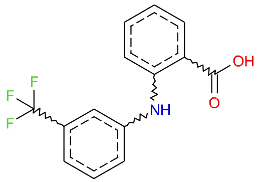
Actual Endpoint	Toxic	Toxic	Non-Toxic
Predicted Endpoint	Toxic	Toxic	Non-Toxic
Distance	0.517	0.567	0.580
Reference	Cornell Vet 64:Suppl 4:97-103; 1974	Arzneimittelforschung 20(4):565-9; 1970	Kiso to Rinsho 13:3302-3313; 1979

Model Applicability

Unknown features are fingerprint features in the query molecule, but not found in the training set.

1. All properties and OPS components are within expected ranges.

Feature Contribution

Top features for positive contribution				
**Fingerprint**	**Bit/Smiles**	**Feature Structure**	**Score**	**Toxic in training set**
SCFP_6	1702664599	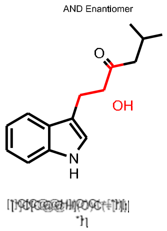	0.322	4 out of 5

SCFP_6	-424515134	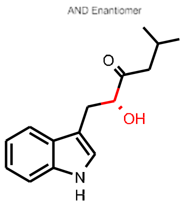 [*]C([*])O	0.275	39 out of 56
SCFP_6	2054003838	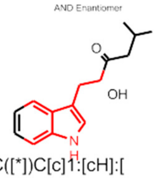 [*]C([*])C[c]1:[cH]:[nH]:[c]2:[cH]:[*]:[*]:[cH]:[c]:1:2	0.271	1 out of 1

Top Features for negative contribution

Fingerprint	Bit/Smiles	Feature Structure	Score	Toxic in training set
SCFP_6	-496201075	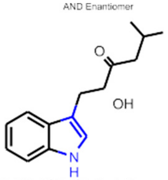 [*][c]1:[*]:[*]:[nH]: [cH]:1	-0.289	8 out of 21
SCFP_6	-1379591900	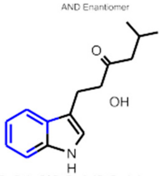 [*]:[c]1:[*]:[cH]:[cH]:[cH]:[cH]:1	-0.282	33 out of 84
SCFP_6	8	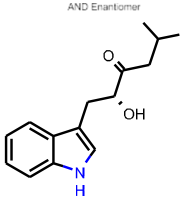 [*]:[nH]:[*]	-0.278	24 out of 61

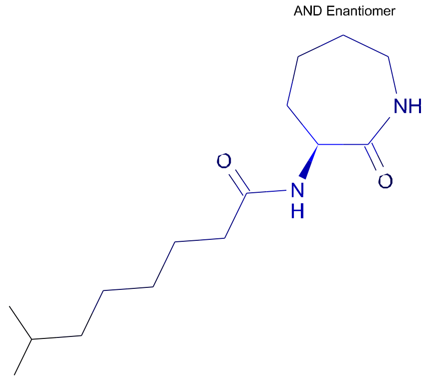

$C_{15}H_{28}N_2O_2$
Molecular Weight: 268.39501
ALogP: 2.685
Rotatable Bonds: 7
Acceptors: 2
Donors: 2

Structural Similar Compounds

Name	Bufexamac	Parbendazole	Befunolol .HCl (Free base form)
Structure	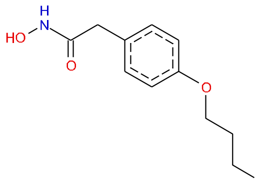	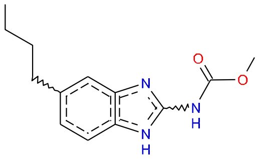	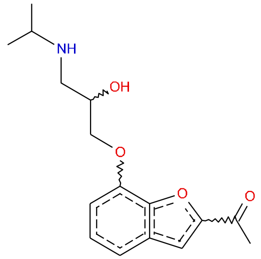
Actual Endpoint	Toxic	Toxic	Non-Toxic
Predicted Endpoint	Toxic	Toxic	Non-Toxic
Distance	0.578	0.631	0.632
Reference	Arzneimittelforschung 20(4):565-9; 1970	Cornell Vet 64:Suppl 4:97-103; 1974	Kiso to Rinsho 13:3678-3714; 1979

Model Prediction

Prediction: Non-Toxic
Probability: 0.336
Enrichment: 0.638
Bayesian Score: -6.983
Mahalanobis Distance: 9.903
Mahalanobis Distance p-value: 0.0268

Prediction: Positive if the Bayesian score is above the estimated best cutoff value from minimizing the false positive and false negative rate.
Probability: The esimated probability that the sample is in the positive category. This assumes that the Bayesian score follows a normal distribution and is different from the prediction using a cutoff.
Enrichment: An estimate of enrichment, that is, the increased likelihood (versus random) of this sample being in the category.
Bayesian Score: The standard Laplacian-modified Bayesian score.
Mahalanobis Distance: The Mahalanobis distance (MD) is the distance to the center of the training data. The larger the MD, the less trustworthy the prediction.
Mahalanobis Distance p-value: The p-value gives the fraction of training data with an MD greater than or equal to the one for the given sample, assuming normally distributed data. The smaller the p-value, the less trustworthy the prediciton. For highly non-normal X properties (e.g., fingerprints), the MD p-value is wildly inaccurate.

Model Applicability

Unknown features are fingerprint features in the query molecule, but not found in the training set.

1. All properties and OPS components are within expected ranges.

Feature Contribution

Top features for positive contribution

Fingerprint	Bit/Smiles	Feature Structure	Score	Toxic in training set
SCFP_6	-1529004787	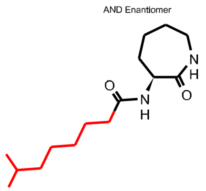 [*]CCCCCC(C)C	0.271	1 out of 1

SCFP_6	9	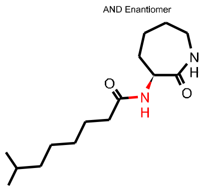 [*]N[*]	0.093	45 out of 78
SCFP_6	-1272798659	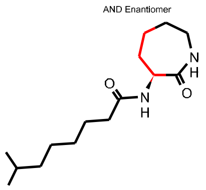 [*]CCC([*])[*]	0.071	44 out of 78

Top Features for negative contribution

Fingerprint	Bit/Smiles	Feature Structure	Score	Toxic in training set
SCFP_6	-1047009138	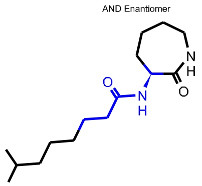 [*]CCC(=O)NC([*])[*]	-0.718	0 out of 2
SCFP_6	-98332825	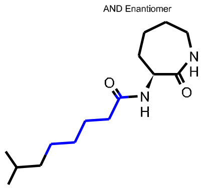 [*]CCCCC(=[*])[*]	-0.718	0 out of 2
SCFP_6	2005026407	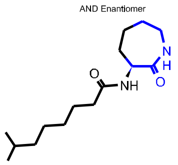 [*][C@H]1[*][*]CCNC1=O	-0.718	0 out of 2

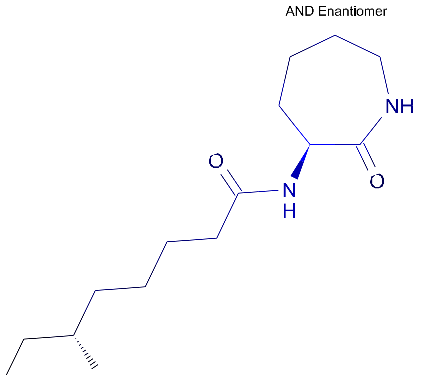

$C_{15}H_{28}N_2O_2$
Molecular Weight: 268.39501
ALogP: 2.685
Rotatable Bonds: 7
Acceptors: 2
Donors: 2

Model Prediction

Prediction: Non-Toxic
Probability: 0.329
Enrichment: 0.626
Bayesian Score: -7.270
Mahalanobis Distance: 9.903
Mahalanobis Distance p-value: 0.0268

Prediction: Positive if the Bayesian score is above the estimated best cutoff value from minimizing the false positive and false negative rate.
Probability: The esimated probability that the sample is in the positive category. This assumes that the Bayesian score follows a normal distribution and is different from the prediction using a cutoff.
Enrichment: An estimate of enrichment, that is, the increased likelihood (versus random) of this sample being in the category.
Bayesian Score: The standard Laplacian-modified Bayesian score.
Mahalanobis Distance: The Mahalanobis distance (MD) is the distance to the center of the training data. The larger the MD, the less trustworthy the prediction.
Mahalanobis Distance p-value: The p-value gives the fraction of training data with an MD greater than or equal to the one for the given sample, assuming normally distributed data. The smaller the p-value, the less trustworthy the prediciton. For highly non-normal X properties (e.g., fingerprints), the MD p-value is wildly inaccurate.

Structural Similar Compounds

Name	Bufexamac	Parbendazole	Befunolol .HCl (Free base form)
Structure	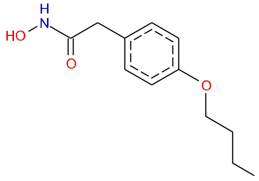	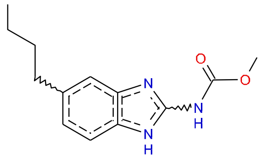	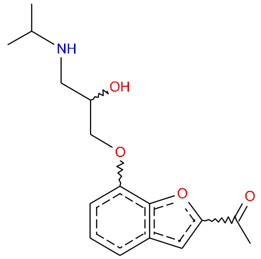
Actual Endpoint	Toxic	Toxic	Non-Toxic
Predicted Endpoint	Toxic	Toxic	Non-Toxic
Distance	0.579	0.632	0.632
Reference	Arzneimittelforschung 20(4):565-9; 1970	Cornell Vet 64:Suppl 4:97-103; 1974	Kiso to Rinsho 13:3678-3714; 1979

Model Applicability

Unknown features are fingerprint features in the query molecule, but not found in the training set.

1. All properties and OPS components are within expected ranges.

Feature Contribution

Top features for positive contribution

Fingerprint	Bit/Smiles	Feature Structure	Score	Toxic in training set
SCFP_6	9	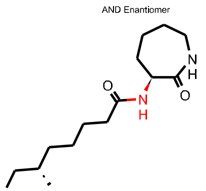 [*]N[*]	0.093	45 out of 78

SCFP_6	-1272798659	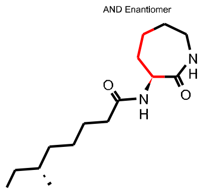 [*]CCC([*])[*]	0.071	44 out of 78
SCFP_6	-1272768868	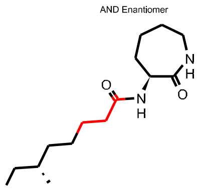 [*]C([*])CC(=[*])[*]	0.061	14 out of 25

Top Features for negative contribution

Fingerprint	Bit/Smiles	Feature Structure	Score	Toxic in training set
SCFP_6	-1047009138	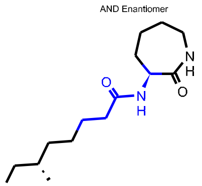 [*]CCC(=O)NC([*])[*]	-0.718	0 out of 2
SCFP_6	2005026407	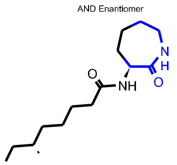 [*][C@H]1[*][*]CCNC1=<br>O	-0.718	0 out of 2
SCFP_6	-98332825	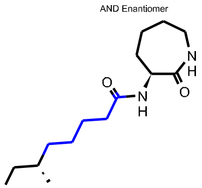 [*]CCCCC(=[*])[*]	-0.718	0 out of 2

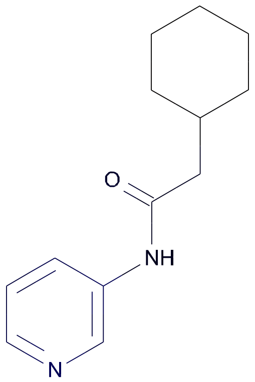

$C_{13}H_{18}N_2O$

Molecular Weight: 218.29481

ALogP: 2.171

Rotatable Bonds: 3

Acceptors: 2

Donors: 1

Model Prediction

Prediction: Non-Toxic

Probability: 0.465

Enrichment: 0.884

Bayesian Score: -2.307

Mahalanobis Distance: 9.253

Mahalanobis Distance p-value: 0.112

Prediction: Positive if the Bayesian score is above the estimated best cutoff value from minimizing the false positive and false negative rate.
Probability: The esimated probability that the sample is in the positive category. This assumes that the Bayesian score follows a normal distribution and is different from the prediction using a cutoff.
Enrichment: An estimate of enrichment, that is, the increased likelihood (versus random) of this sample being in the category.
Bayesian Score: The standard Laplacian-modified Bayesian score.
Mahalanobis Distance: The Mahalanobis distance (MD) is the distance to the center of the training data. The larger the MD, the less trustworthy the prediction.
Mahalanobis Distance p-value: The p-value gives the fraction of training data with an MD greater than or equal to the one for the given sample, assuming normally distributed data. The smaller the p-value, the less trustworthy the prediciton. For highly non-normal X properties (e.g., fingerprints), the MD p-value is wildly inaccurate.

Structural Similar Compounds

Name	Carbaryl	Mexiletine .HCl (Free base form)	Miroprofen
Structure	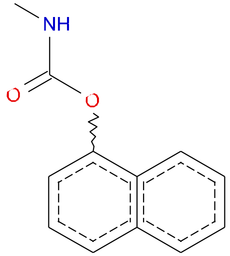	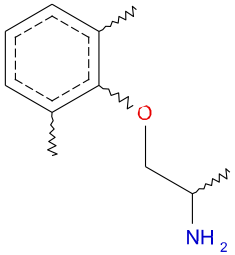	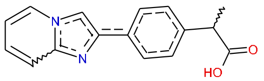
Actual Endpoint	Non-Toxic	Non-Toxic	Non-Toxic
Predicted Endpoint	Non-Toxic	Non-Toxic	Non-Toxic
Distance	0.516	0.534	0.540
Reference	Toxicol Appl Pharmacol 26(4):621-38; 1973	Iyakuhin Kenkyu 14(4):550-570; 1983	Iyakuhin Kenkyu 12:808-826; 1981

Model Applicability

Unknown features are fingerprint features in the query molecule, but not found in the training set.

1. All properties and OPS components are within expected ranges.

Feature Contribution

Top features for positive contribution

Fingerprint	Bit/Smiles	Feature Structure	Score	Toxic in training set
SCFP_6	-1529004787	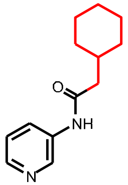 [*]CCCCCC(C)C	0.271	1 out of 1

SCFP_6	1631845520	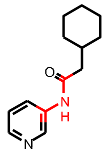 [*]C(=[*])N[c](:[*]): [*]	0.210	8 out of 12
SCFP_6	1205586762	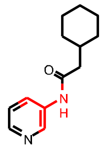 [*]N[c](:[cH]:[*]):[c H]:[*]	0.139	11 out of 18
**Top Features for negative contribution**				
**Fingerprint**	**Bit/Smiles**	**Feature Structure**	**Score**	**Toxic in training set**
SCFP_6	-758850909	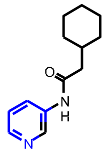 [*][c]1:[*]:n:[cH]:[c H]:[cH]:1	-0.646	2 out of 9
SCFP_6	2097618059	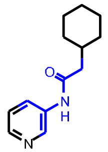 [*]CC(=O)N[c](:[cH]:[*]):[cH]:[*]	-0.422	0 out of 1
SCFP_6	-216207339	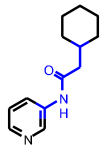 [*]C([*])CC(=O)N[c](: [*]):[*]	-0.422	0 out of 1

Indinavir

TOPKAT_Developmental_Toxicity_Potential

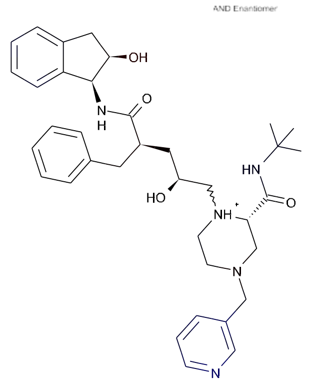

$C_{36}H_{48}N_5O_4$
Molecular Weight: 614.79741
ALogP: 1.521
Rotatable Bonds: 12
Acceptors: 6
Donors: 5

Structural Similar Compounds

Name	Ametantrone Acetate	Prostaglandin e2	Enalapril
Structure	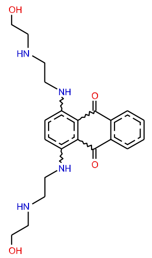	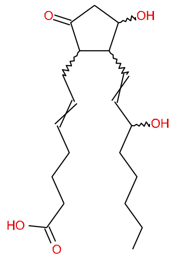	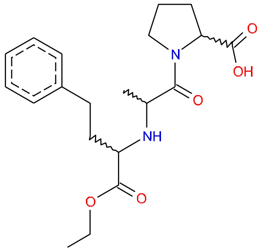
Actual Endpoint	Non-Toxic	Non-Toxic	Non-Toxic
Predicted Endpoint	Non-Toxic	Non-Toxic	Non-Toxic
Distance	0.818	0.928	0.970
Reference	Teratology 34:271-278; 1986	Yakuri to Chiryo 9:1369-1394; 1981	Yakuri to Chiryo 13:519-528; 1985

Model Prediction

Prediction: Non-Toxic
Probability: 0.480
Enrichment: 0.912
Bayesian Score: -1.870
Mahalanobis Distance: 11.327
Mahalanobis Distance p-value: 0.000358

Prediction: Positive if the Bayesian score is above the estimated best cutoff value from minimizing the false positive and false negative rate.
Probability: The esimated probability that the sample is in the positive category. This assumes that the Bayesian score follows a normal distribution and is different from the prediction using a cutoff.
Enrichment: An estimate of enrichment, that is, the increased likelihood (versus random) of this sample being in the category.
Bayesian Score: The standard Laplacian-modified Bayesian score.
Mahalanobis Distance: The Mahalanobis distance (MD) is the distance to the center of the training data. The larger the MD, the less trustworthy the prediction.
Mahalanobis Distance p-value: The p-value gives the fraction of training data with an MD greater than or equal to the one for the given sample, assuming normally distributed data. The smaller the p-value, the less trustworthy the prediciton. For highly non-normal X properties (e.g., fingerprints), the MD p-value is wildly inaccurate.

Model Applicability

Unknown features are fingerprint features in the query molecule, but not found in the training set.

1. All properties and OPS components are within expected ranges.

Feature Contribution

Top features for positive contribution				
Fingerprint	Bit/Smiles	Feature Structure	Score	Toxic in training set
SCFP_6	-1849894309	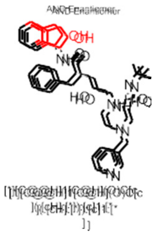	0.381	2 out of 2

SCFP_6	2088704928	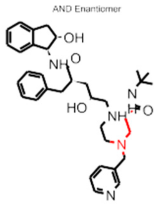<br>[*]C([*])CN([*])[*]	0.303	38 out of 53
SCFP_6	-424515134	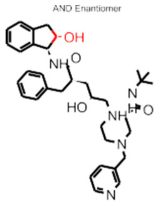<br>[*]C([*])O	0.275	39 out of 56

Top Features for negative contribution

Fingerprint	Bit/Smiles	Feature Structure	Score	Toxic in training set
SCFP_6	-758850909	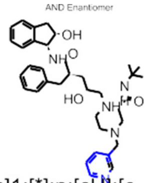<br>[*][c]1:[*]:n:[cH]:[cH]:[cH]:1	-0.646	2 out of 9
SCFP_6	562969918	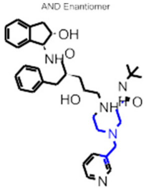<br>[*][C@H]1[*]CCN(C[c](:[*]):[*])C1	-0.422	0 out of 1
SCFP_6	-1136473982	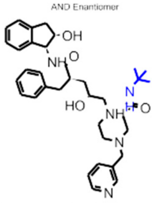<br>[*]NC(C)(C)C	-0.422	0 out of 1

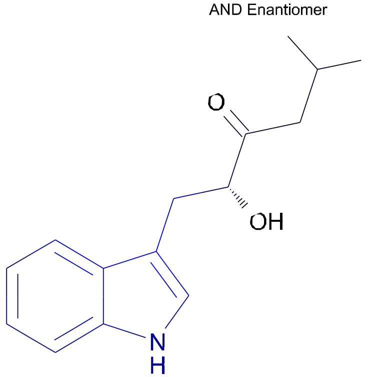

$C_{15}H_{19}NO_2$
Molecular Weight: 245.31685
ALogP: 2.932
Rotatable Bonds: 5
Acceptors: 2
Donors: 2

Model Prediction

Prediction: Non-Carcinogen
Probability: 0.205
Enrichment: 0.640
Bayesian Score: -5.708
Mahalanobis Distance: 9.813
Mahalanobis Distance p-value: 0.532

Prediction: Positive if the Bayesian score is above the estimated best cutoff value from minimizing the false positive and false negative rate.
Probability: The esimated probability that the sample is in the positive category. This assumes that the Bayesian score follows a normal distribution and is different from the prediction using a cutoff.
Enrichment: An estimate of enrichment, that is, the increased likelihood (versus random) of this sample being in the category.
Bayesian Score: The standard Laplacian-modified Bayesian score.
Mahalanobis Distance: The Mahalanobis distance (MD) is the distance to the center of the training data. The larger the MD, the less trustworthy the prediction.
Mahalanobis Distance p-value: The p-value gives the fraction of training data with an MD greater than or equal to the one for the given sample, assuming normally distributed data. The smaller the p-value, the less trustworthy the prediciton. For highly non-normal X properties (e.g., fingerprints), the MD p-value is wildly inaccurate.

Structural Similar Compounds

Name	Prilocaine	Propranolol	Pronetalol
Structure	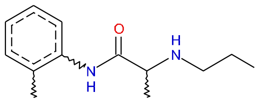	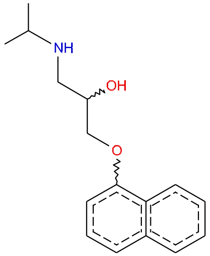	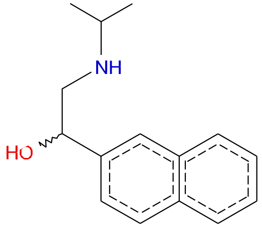
Actual Endpoint	Carcinogen	Non-Carcinogen	Carcinogen
Predicted Endpoint	Carcinogen	Non-Carcinogen	Carcinogen
Distance	0.518	0.550	0.553
Reference	US FDA (Centre for Drug Eval.& Res./Off. Testing & Res.) Sept. 1997	US FDA (Centre for Drug Eval.& Res./Off. Testing & Res.) Sept. 1997	US FDA (Centre for Drug Eval.& Res./Off. Testing & Res.) Sept. 1997

Model Applicability

Unknown features are fingerprint features in the query molecule, but not found in the training set.

1. All properties and OPS components are within expected ranges.
2. Unknown ECFP_2 feature: 80433051: [*]C[C@@H](O)C(=[*])[*]
3. Unknown ECFP_2 feature: 1732075620: [*]CC(=O)C([*])[*]

Feature Contribution

Top features for positive contribution				
Fingerprint	Bit/Smiles	Feature Structure	Score	Carcinogen in training set
ECFP_6	-953984246	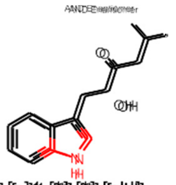 [*]:[c]1:[*]:[*]:[cH]:[nH]:1	0.364	4 out of 8

ECFP_6	-152683720	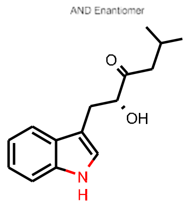<br>[*]:[nH]:[*]	0.245	7 out of 17
ECFP_6	1099224616	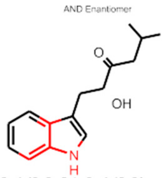<br>[*]:[cH]:[c]1:[nH]:[*<br>]:[*]:[c]:1:[*]	0.127	4 out of 11
**Top Features for negative contribution**				
**Fingerprint**	**Bit/Smiles**	**Feature Structure**	**Score**	**Carcinogen in training set**
ECFP_6	-1659633832	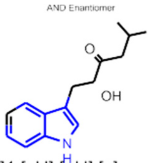<br>[*][c]1:[cH]:[nH]:[c]<br>2:[cH]:[cH]:[*]:[cH]<br>:[c]:1:2	-0.657	0 out of 3
ECFP_6	770157610	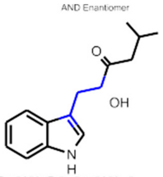<br>[*]C([*])C[c](:[*]):[<br>*]	-0.482	0 out of 2
ECFP_6	-93267474	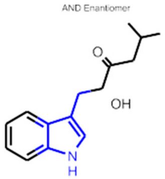<br>[*]C[c]1:[cH]:[nH]:[c<br>](:[*]):[c]:1:[*]	-0.482	0 out of 2

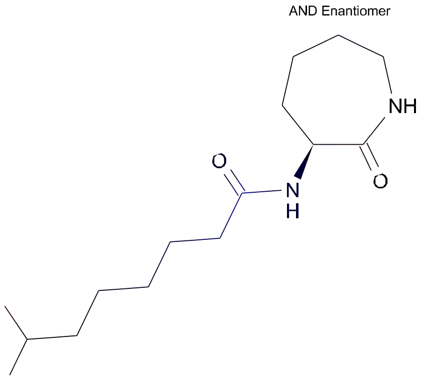

$C_{15}H_{28}N_2O_2$
Molecular Weight: 268.39501
ALogP: 2.685
Rotatable Bonds: 7
Acceptors: 2
Donors: 2

Structural Similar Compounds

Name	Propranolol	Penbutalol	Prilocaine
Structure	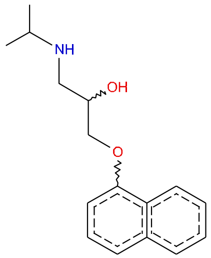	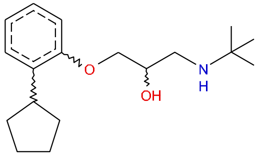	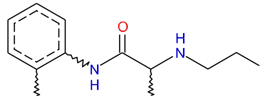
Actual Endpoint	Non-Carcinogen	Non-Carcinogen	Carcinogen
Predicted Endpoint	Non-Carcinogen	Non-Carcinogen	Carcinogen
Distance	0.587	0.597	0.606
Reference	US FDA (Centre for Drug Eval.& Res./Off. Testing & Res.) Sept. 1997	US FDA (Centre for Drug Eval.& Res./Off. Testing & Res.) Sept. 1997	US FDA (Centre for Drug Eval.& Res./Off. Testing & Res.) Sept. 1997

Model Prediction

Prediction: Non-Carcinogen
Probability: 0.225
Enrichment: 0.702
Bayesian Score: -2.492
Mahalanobis Distance: 14.506
Mahalanobis Distance p-value: 1.97e-006

Prediction: Positive if the Bayesian score is above the estimated best cutoff value from minimizing the false positive and false negative rate.
Probability: The esimated probability that the sample is in the positive category. This assumes that the Bayesian score follows a normal distribution and is different from the prediction using a cutoff.
Enrichment: An estimate of enrichment, that is, the increased likelihood (versus random) of this sample being in the category.
Bayesian Score: The standard Laplacian-modified Bayesian score.
Mahalanobis Distance: The Mahalanobis distance (MD) is the distance to the center of the training data. The larger the MD, the less trustworthy the prediction.
Mahalanobis Distance p-value: The p-value gives the fraction of training data with an MD greater than or equal to the one for the given sample, assuming normally distributed data. The smaller the p-value, the less trustworthy the prediciton. For highly non-normal X properties (e.g., fingerprints), the MD p-value is wildly inaccurate.

Model Applicability

Unknown features are fingerprint features in the query molecule, but not found in the training set.

1. All properties and OPS components are within expected ranges.

Feature Contribution

Top features for positive contribution

Fingerprint	Bit/Smiles	Feature Structure	Score	Carcinogen in training set
ECFP_6	-1291950249	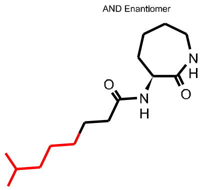 [*]CCCC(C)C	0.424	1 out of 1

ECFP_6	-745187652	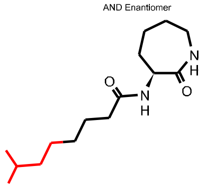 [*]CCC(C)C	0.424	1 out of 1
ECFP_6	2106656448	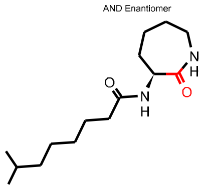 [*]C(=O)[*]	0.254	31 out of 77
**Top Features for negative contribution**				
**Fingerprint**	**Bit/Smiles**	**Feature Structure**	**Score**	**Carcinogen in training set**
ECFP_6	1731843802	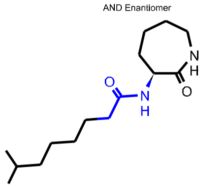 [*]CC(=O)N[*]	-0.657	0 out of 3
ECFP_6	-2091181441	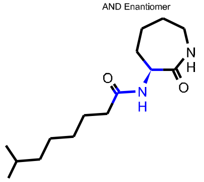 [*]C([*])NC(=[*])[*]	-0.638	1 out of 9
ECFP_6	-2123658813	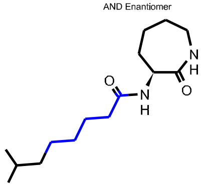 [*]CCCCC(=[*])[*]	-0.482	0 out of 2

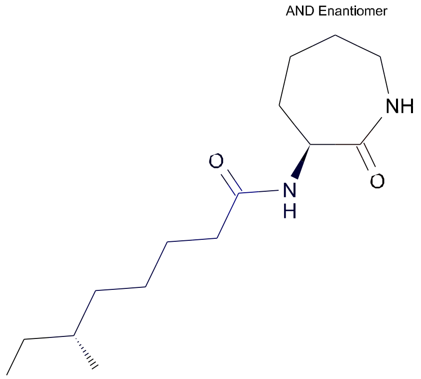

$C_{15}H_{28}N_2O_2$
Molecular Weight: 268.39501
ALogP: 2.685
Rotatable Bonds: 7
Acceptors: 2
Donors: 2

Structural Similar Compounds

Name	Propranolol	Penbutalol	Prilocaine
Structure	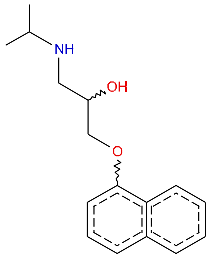	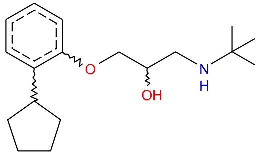	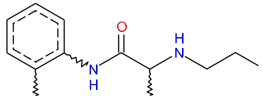
Actual Endpoint	Non-Carcinogen	Non-Carcinogen	Carcinogen
Predicted Endpoint	Non-Carcinogen	Non-Carcinogen	Carcinogen
Distance	0.588	0.598	0.601
Reference	US FDA (Centre for Drug Eval.& Res./Off. Testing & Res.) Sept. 1997	US FDA (Centre for Drug Eval.& Res./Off. Testing & Res.) Sept. 1997	US FDA (Centre for Drug Eval.& Res./Off. Testing & Res.) Sept. 1997

Model Prediction

Prediction: Non-Carcinogen
Probability: 0.217
Enrichment: 0.676
Bayesian Score: -3.339
Mahalanobis Distance: 14.795
Mahalanobis Distance p-value: 5.95e-007

Prediction: Positive if the Bayesian score is above the estimated best cutoff value from minimizing the false positive and false negative rate.
Probability: The esimated probability that the sample is in the positive category. This assumes that the Bayesian score follows a normal distribution and is different from the prediction using a cutoff.
Enrichment: An estimate of enrichment, that is, the increased likelihood (versus random) of this sample being in the category.
Bayesian Score: The standard Laplacian-modified Bayesian score.
Mahalanobis Distance: The Mahalanobis distance (MD) is the distance to the center of the training data. The larger the MD, the less trustworthy the prediction.
Mahalanobis Distance p-value: The p-value gives the fraction of training data with an MD greater than or equal to the one for the given sample, assuming normally distributed data. The smaller the p-value, the less trustworthy the prediciton. For highly non-normal X properties (e.g., fingerprints), the MD p-value is wildly inaccurate.

Model Applicability

Unknown features are fingerprint features in the query molecule, but not found in the training set.

1. All properties and OPS components are within expected ranges.
2. Unknown ECFP_2 feature: 194354829: [*]CC(C)C[*]

Feature Contribution

Top features for positive contribution

Fingerprint	Bit/Smiles	Feature Structure	Score	Carcinogen in training set
ECFP_6	2106656448	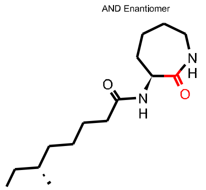 [*]C(=O)[*]	0.254	31 out of 77

ECFP_6	-1331450522	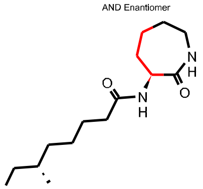 [*]CCC([*])[*]	0.238	25 out of 63
ECFP_6	-2097159651	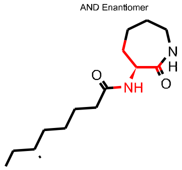 [*]C[C@H](N[*])C(=[*])[*]	0.212	1 out of 2

Top Features for negative contribution

Fingerprint	Bit/Smiles	Feature Structure	Score	Carcinogen in training set
ECFP_6	1731843802	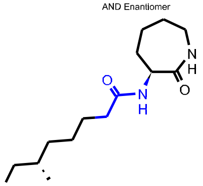 [*]CC(=O)N[*]	-0.657	0 out of 3
ECFP_6	-2091181441	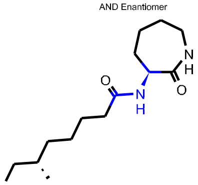 [*]C([*])NC(=[*])[*]	-0.638	1 out of 9
ECFP_6	-2123658813	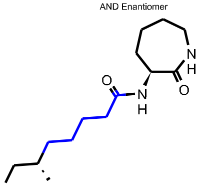 [*]CCCCC(=[*])[*]	-0.482	0 out of 2

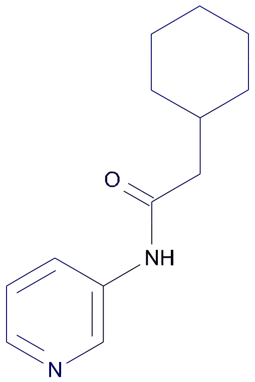

$C_{13}H_{18}N_2O$
Molecular Weight: 218.29481
ALogP: 2.171
Rotatable Bonds: 3
Acceptors: 2
Donors: 1

Model Prediction

Prediction: Non-Carcinogen
Probability: 0.209
Enrichment: 0.651
Bayesian Score: -4.614
Mahalanobis Distance: 9.595
Mahalanobis Distance p-value: 0.633

Prediction: Positive if the Bayesian score is above the estimated best cutoff value from minimizing the false positive and false negative rate.
Probability: The esimated probability that the sample is in the positive category. This assumes that the Bayesian score follows a normal distribution and is different from the prediction using a cutoff.
Enrichment: An estimate of enrichment, that is, the increased likelihood (versus random) of this sample being in the category.
Bayesian Score: The standard Laplacian-modified Bayesian score.
Mahalanobis Distance: The Mahalanobis distance (MD) is the distance to the center of the training data. The larger the MD, the less trustworthy the prediction.
Mahalanobis Distance p-value: The p-value gives the fraction of training data with an MD greater than or equal to the one for the given sample, assuming normally distributed data. The smaller the p-value, the less trustworthy the prediciton. For highly non-normal X properties (e.g., fingerprints), the MD p-value is wildly inaccurate.

Structural Similar Compounds

Name	Phenacetin	Methylphenidate	Mexiletine
Structure	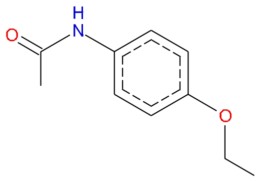	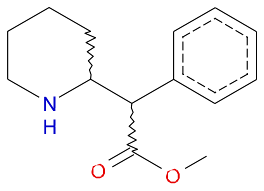	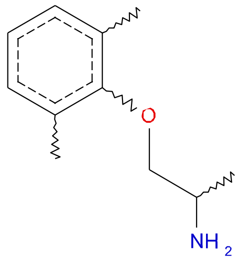
Actual Endpoint	Carcinogen	Carcinogen	Non-Carcinogen
Predicted Endpoint	Carcinogen	Carcinogen	Non-Carcinogen
Distance	0.533	0.537	0.538
Reference	US FDA (Centre for Drug Eval.& Res./Off. Testing & Res.) Sept. 1997	US FDA (Centre for Drug Eval.& Res./Off. Testing & Res.) Sept. 1997	US FDA (Centre for Drug Eval.& Res./Off. Testing & Res.) Sept. 1997

Model Applicability

Unknown features are fingerprint features in the query molecule, but not found in the training set.

1. All properties and OPS components are within expected ranges.
2. Unknown ECFP_2 feature: -82840383: [*]C([*])CC(=[*])[*]

Feature Contribution

Top features for positive contribution				
**Fingerprint**	**Bit/Smiles**	**Feature Structure**	**Score**	**Carcinogen in training set**
ECFP_6	-177077903	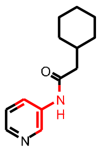 [*]N[c](:[cH]:[*]):[cH]:[*]	0.279	4 out of 9

ECFP_6	-1331450522	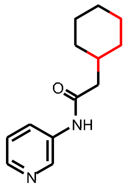 [*]CCC([*])[*]	0.238	25 out of 63
ECFP_6	894876384	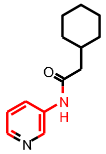 [*]N[c]1:[cH]:[*]:[cH]:[cH]:[cH]:1	0.212	1 out of 2
**Top Features for negative contribution**				
**Fingerprint**	**Bit/Smiles**	**Feature Structure**	**Score**	**Carcinogen in training set**
ECFP_6	2013347047	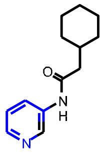 [*][c]1:[*]:n:[cH]:[c H]:[cH]:1	-0.805	0 out of 4
ECFP_6	-1607899848	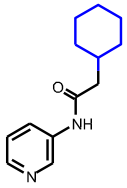 [*]C1CCCCC1	-0.805	0 out of 4
ECFP_6	662850656	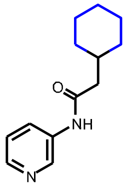 [*]1CCCCC1	-0.716	1 out of 10

Indinavir

TOPKAT_Mouse_Female_FDA_None_vs_Carcinogen

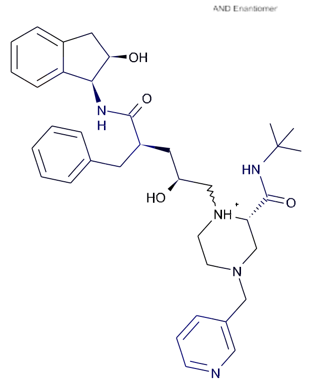

$C_{36}H_{48}N_5O_4$
Molecular Weight: 614.79741
ALogP: 1.521
Rotatable Bonds: 12
Acceptors: 6
Donors: 5

Structural Similar Compounds

Name	Pravastatin	Quinapril	Glyburide
Structure	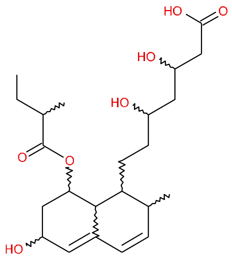	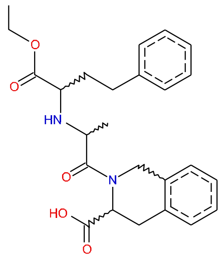	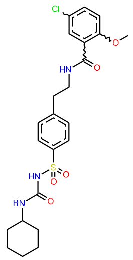
Actual Endpoint	Non-Carcinogen	Non-Carcinogen	Non-Carcinogen
Predicted Endpoint	Non-Carcinogen	Non-Carcinogen	Non-Carcinogen
Distance	0.852	0.964	0.969
Reference	US FDA (Centre for Drug Eval.& Res./Off. Testing & Res.) Sept. 1997	US FDA (Centre for Drug Eval.& Res./Off. Testing & Res.) Sept. 1997	US FDA (Centre for Drug Eval.& Res./Off. Testing & Res.) Sept. 1997

Model Prediction

Prediction: Non-Carcinogen
Probability: 0.225
Enrichment: 0.702
Bayesian Score: -11.796
Mahalanobis Distance: 16.880
Mahalanobis Distance p-value: 4.32e-011

Prediction: Positive if the Bayesian score is above the estimated best cutoff value from minimizing the false positive and false negative rate.
Probability: The esimated probability that the sample is in the positive category. This assumes that the Bayesian score follows a normal distribution and is different from the prediction using a cutoff.
Enrichment: An estimate of enrichment, that is, the increased likelihood (versus random) of this sample being in the category.
Bayesian Score: The standard Laplacian-modified Bayesian score.
Mahalanobis Distance: The Mahalanobis distance (MD) is the distance to the center of the training data. The larger the MD, the less trustworthy the prediction.
Mahalanobis Distance p-value: The p-value gives the fraction of training data with an MD greater than or equal to the one for the given sample, assuming normally distributed data. The smaller the p-value, the less trustworthy the prediciton. For highly non-normal X properties (e.g., fingerprints), the MD p-value is wildly inaccurate.

Model Applicability

Unknown features are fingerprint features in the query molecule, but not found in the training set.

1. All properties and OPS components are within expected ranges.
2. Unknown ECFP_2 feature: 1976330679: [*][NH+]([*])[*]
3. Unknown ECFP_2 feature: 1134829831: [*]C[NH+](C[*])C([*])[*]
4. Unknown ECFP_2 feature: -1924540582: [*]C[C@H]([NH+]([*])[*])C(=[*])[*]
5. Unknown ECFP_2 feature: -244159614: [*]CC[NH+]([*])[*]
6. Unknown ECFP_2 feature: 474121058: [*]C([*])C[NH+]([*])[*]

Feature Contribution

Top features for positive contribution

Fingerprint	Bit/Smiles	Feature Structure	Score	Carcinogen in training set
ECFP_6	1336304100	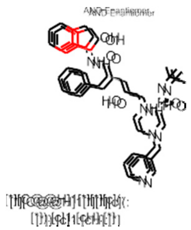	0.296	7 out of 16

ECFP_6	-709633021	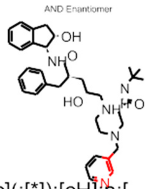 [*][c](:[*]):[cH]:n:[*]	0.178	5 out of 13
ECFP_6	769323258	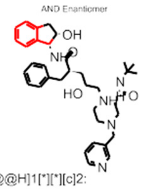 [*][C@@H]1[*][*][c]2: [cH]:[cH]:[cH]:[cH]: [c]1:2	0.164	2 out of 5
**Top Features for negative contribution**				
**Fingerprint**	**Bit/Smiles**	**Feature Structure**	**Score**	**Carcinogen in training set**
ECFP_6	2013347047	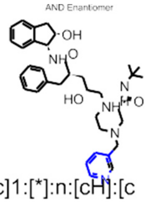 [*][c]1:[*]:n:[cH]:[c H]:[cH]:1	-0.805	0 out of 4
ECFP_6	-1567199489	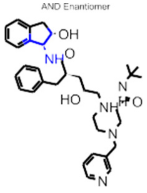 [*]N[C@@H]1[C@H]([*]) [*][*][c]1:[*]	-0.657	0 out of 3
ECFP_6	-2091181441	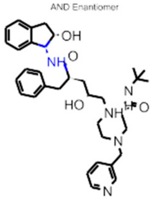 [*]C([*])NC(=[*])[*]	-0.638	1 out of 9

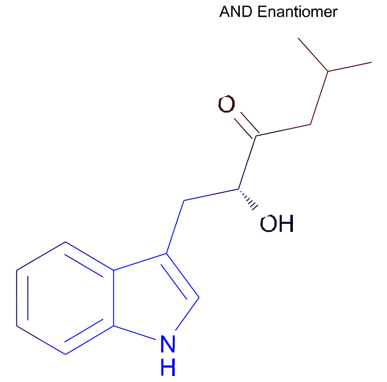

$C_{15}H_{19}NO_2$
Molecular Weight: 245.31685
ALogP: 2.932
Rotatable Bonds: 5
Acceptors: 2
Donors: 2

Model Prediction

Prediction: Non-Carcinogen
Probability: 0.189
Enrichment: 0.643
Bayesian Score: -5.686
Mahalanobis Distance: 10.772
Mahalanobis Distance p-value: 0.117

Prediction: Positive if the Bayesian score is above the estimated best cutoff value from minimizing the false positive and false negative rate.
Probability: The esimated probability that the sample is in the positive category. This assumes that the Bayesian score follows a normal distribution and is different from the prediction using a cutoff.
Enrichment: An estimate of enrichment, that is, the increased likelihood (versus random) of this sample being in the category.
Bayesian Score: The standard Laplacian-modified Bayesian score.
Mahalanobis Distance: The Mahalanobis distance (MD) is the distance to the center of the training data. The larger the MD, the less trustworthy the prediction.
Mahalanobis Distance p-value: The p-value gives the fraction of training data with an MD greater than or equal to the one for the given sample, assuming normally distributed data. The smaller the p-value, the less trustworthy the prediciton. For highly non-normal X properties (e.g., fingerprints), the MD p-value is wildly inaccurate.

Structural Similar Compounds

Name	Prilocaine	Pronetalol	Etodolac
Structure	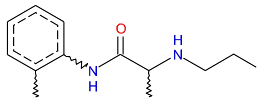	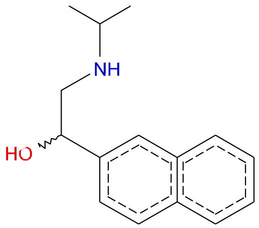	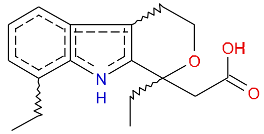
Actual Endpoint	Carcinogen	Carcinogen	Non-Carcinogen
Predicted Endpoint	Carcinogen	Carcinogen	Non-Carcinogen
Distance	0.514	0.530	0.539
Reference	US FDA (Centre for Drug Eval.& Res./Off. Testing & Res.) Sept. 1997	US FDA (Centre for Drug Eval.& Res./Off. Testing & Res.) Sept. 1997	US FDA (Centre for Drug Eval.& Res./Off. Testing & Res.) Sept. 1997

Model Applicability

Unknown features are fingerprint features in the query molecule, but not found in the training set.

1. All properties and OPS components are within expected ranges.

Feature Contribution

Top features for positive contribution

Fingerprint	Bit/Smiles	Feature Structure	Score	Carcinogen in training set
FCFP_6	-387072142	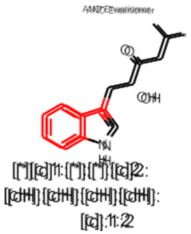	0.477	4 out of 8

FCFP_6	1306984497	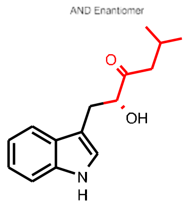 [*]C([*])C(=O)CC(C)C	0.460	1 out of 1
FCFP_6	-1043339860	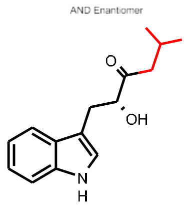 [*]CC(C)C	0.383	24 out of 61
**Top Features for negative contribution**				
**Fingerprint**	**Bit/Smiles**	**Feature Structure**	**Score**	**Carcinogen in training set**
FCFP_6	1618184456	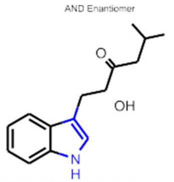 [*][c]1:[*]:[*]:[nH]:[cH]:1	-0.719	0 out of 4
FCFP_6	-1828565903	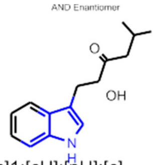 [*][c]1:[cH]:[nH]:[c](:[cH]:[*]):[c]:1:[*]	-0.719	0 out of 4
FCFP_6	155061250	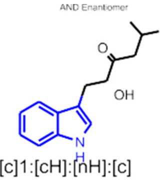 [*][c]1:[cH]:[nH]:[c]2:[cH]:[cH]:[cH]:[cH]:[c]:1:2	-0.719	0 out of 4

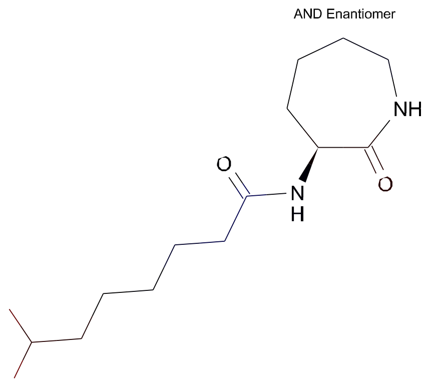

$C_{15}H_{28}N_2O_2$
Molecular Weight: 268.39501
ALogP: 2.685
Rotatable Bonds: 7
Acceptors: 2
Donors: 2

Structural Similar Compounds

Name	Propranolol	Prilocaine	Penbutalol
Structure	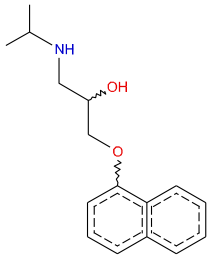	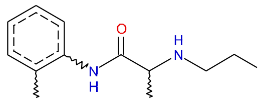	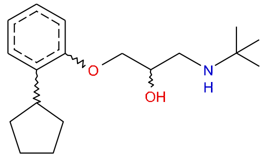
Actual Endpoint	Non-Carcinogen	Carcinogen	Non-Carcinogen
Predicted Endpoint	Non-Carcinogen	Carcinogen	Non-Carcinogen
Distance	0.567	0.570	0.587
Reference	US FDA (Centre for Drug Eval.& Res./Off. Testing & Res.) Sept. 1997	US FDA (Centre for Drug Eval.& Res./Off. Testing & Res.) Sept. 1997	US FDA (Centre for Drug Eval.& Res./Off. Testing & Res.) Sept. 1997

Model Prediction

Prediction: Carcinogen
Probability: 0.322
Enrichment: 1.095
Bayesian Score: 0.590
Mahalanobis Distance: 9.460
Mahalanobis Distance p-value: 0.625

Prediction: Positive if the Bayesian score is above the estimated best cutoff value from minimizing the false positive and false negative rate.
Probability: The esimated probability that the sample is in the positive category. This assumes that the Bayesian score follows a normal distribution and is different from the prediction using a cutoff.
Enrichment: An estimate of enrichment, that is, the increased likelihood (versus random) of this sample being in the category.
Bayesian Score: The standard Laplacian-modified Bayesian score.
Mahalanobis Distance: The Mahalanobis distance (MD) is the distance to the center of the training data. The larger the MD, the less trustworthy the prediction.
Mahalanobis Distance p-value: The p-value gives the fraction of training data with an MD greater than or equal to the one for the given sample, assuming normally distributed data. The smaller the p-value, the less trustworthy the prediciton. For highly non-normal X properties (e.g., fingerprints), the MD p-value is wildly inaccurate.

Model Applicability

Unknown features are fingerprint features in the query molecule, but not found in the training set.

1. All properties and OPS components are within expected ranges.

Feature Contribution

Top features for positive contribution				
**Fingerprint**	**Bit/Smiles**	**Feature Structure**	**Score**	**Carcinogen in training set**
FCFP_6	-55265897	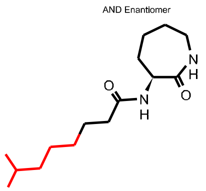 [*]CCCC(C)C	0.594	17 out of 34

FCFP_6	-154166589	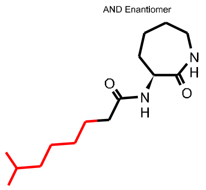 [*]CCCCC(C)C	0.460	1 out of 1
FCFP_6	566058135	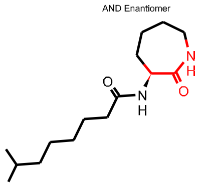 [*]NC(=O)C([*])[*]	0.447	17 out of 40
**Top Features for negative contribution**				
**Fingerprint**	**Bit/Smiles**	**Feature Structure**	**Score**	**Carcinogen in training set**
FCFP_6	-1525101452	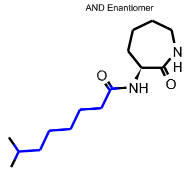 [*]C([*])CCCCCC(=[*])[*]	-1.133	0 out of 8
FCFP_6	494226440	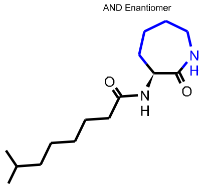 [*]1[*]NCCCC1	-0.254	1 out of 6
FCFP_6	-228300541	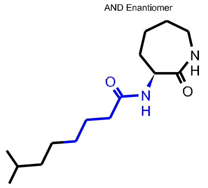 [*]CCCC(=O)N[*]	-0.233	0 out of 1

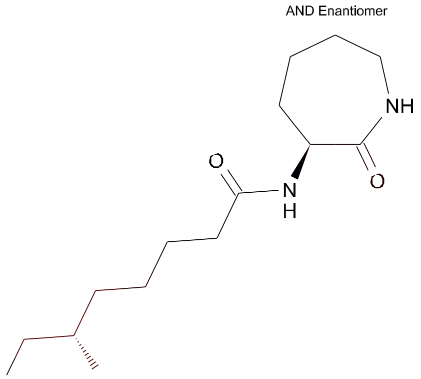

$C_{15}H_{28}N_2O_2$
Molecular Weight: 268.39501
ALogP: 2.685
Rotatable Bonds: 7
Acceptors: 2
Donors: 2

Model Prediction

Prediction: Carcinogen
Probability: 0.346
Enrichment: 1.175
Bayesian Score: 1.375
Mahalanobis Distance: 9.460
Mahalanobis Distance p-value: 0.625

Prediction: Positive if the Bayesian score is above the estimated best cutoff value from minimizing the false positive and false negative rate.
Probability: The esimated probability that the sample is in the positive category. This assumes that the Bayesian score follows a normal distribution and is different from the prediction using a cutoff.
Enrichment: An estimate of enrichment, that is, the increased likelihood (versus random) of this sample being in the category.
Bayesian Score: The standard Laplacian-modified Bayesian score.
Mahalanobis Distance: The Mahalanobis distance (MD) is the distance to the center of the training data. The larger the MD, the less trustworthy the prediction.
Mahalanobis Distance p-value: The p-value gives the fraction of training data with an MD greater than or equal to the one for the given sample, assuming normally distributed data. The smaller the p-value, the less trustworthy the prediciton. For highly non-normal X properties (e.g., fingerprints), the MD p-value is wildly inaccurate.

Structural Similar Compounds

Name	Propranolol	Prilocaine	Penbutalol
Structure	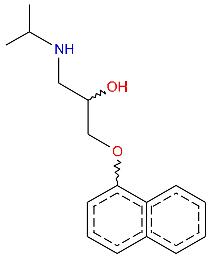	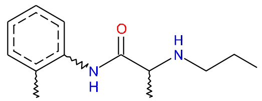	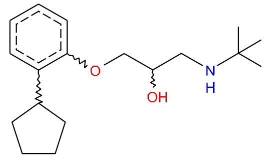
Actual Endpoint	Non-Carcinogen	Carcinogen	Non-Carcinogen
Predicted Endpoint	Non-Carcinogen	Carcinogen	Non-Carcinogen
Distance	0.568	0.571	0.588
Reference	US FDA (Centre for Drug Eval.& Res./Off. Testing & Res.) Sept. 1997	US FDA (Centre for Drug Eval.& Res./Off. Testing & Res.) Sept. 1997	US FDA (Centre for Drug Eval.& Res./Off. Testing & Res.) Sept. 1997

Model Applicability

Unknown features are fingerprint features in the query molecule, but not found in the training set.

1. All properties and OPS components are within expected ranges.

Feature Contribution

Top features for positive contribution				
**Fingerprint**	**Bit/Smiles**	**Feature Structure**	**Score**	**Carcinogen in training set**
FCFP_6	-55265897	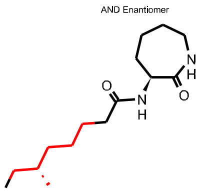 [*]CCCC(C)C	0.594	17 out of 34

FCFP_6	566058135	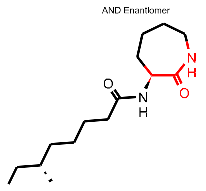 [*]NC(=O)C([*])[*]	0.447	17 out of 40
FCFP_6	-1043339860	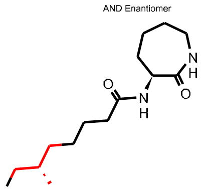 [*]CC(C)C	0.383	24 out of 61

Top Features for negative contribution

Fingerprint	Bit/Smiles	Feature Structure	Score	Carcinogen in training set
FCFP_6	494226440	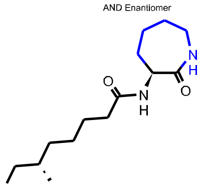 [*]1[*]NCCCC1	-0.254	1 out of 6
FCFP_6	-228300541	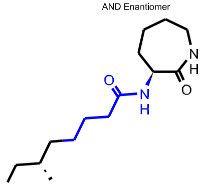 [*]CCCC(=O)N[*]	-0.233	0 out of 1
FCFP_6	-885550502	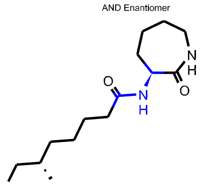 [*]C([*])NC(=[*])[*]	-0.186	21 out of 97

GWS **TOPKAT_Mouse_Male_FDA_None_vs_Carcinogen**

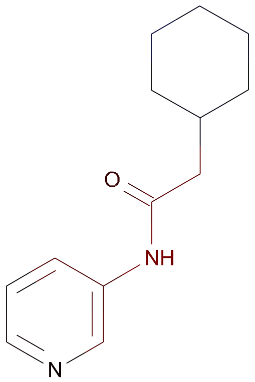

$C_{13}H_{18}N_2O$

Molecular Weight: 218.29481

ALogP: 2.171

Rotatable Bonds: 3

Acceptors: 2

Donors: 1

Structural Similar Compounds

Name	Phenacetin	Mexiletine	Methylphenidate
Structure	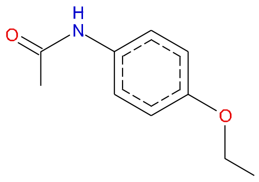	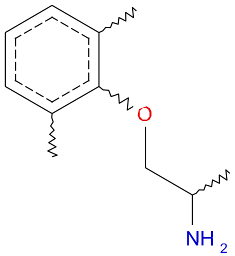	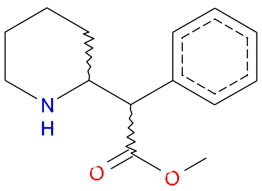
Actual Endpoint	Carcinogen	Non-Carcinogen	Carcinogen
Predicted Endpoint	Carcinogen	Non-Carcinogen	Carcinogen
Distance	0.503	0.530	0.551
Reference	US FDA (Centre for Drug Eval.& Res./Off. Testing & Res.) Sept. 1997	US FDA (Centre for Drug Eval.& Res./Off. Testing & Res.) Sept. 1997	US FDA (Centre for Drug Eval.& Res./Off. Testing & Res.) Sept. 1997

Model Prediction

Prediction: Carcinogen

Probability: 0.393

Enrichment: 1.334

Bayesian Score: 2.802

Mahalanobis Distance: 10.758

Mahalanobis Distance p-value: 0.12

Prediction: Positive if the Bayesian score is above the estimated best cutoff value from minimizing the false positive and false negative rate.
Probability: The esimated probability that the sample is in the positive category. This assumes that the Bayesian score follows a normal distribution and is different from the prediction using a cutoff.
Enrichment: An estimate of enrichment, that is, the increased likelihood (versus random) of this sample being in the category.
Bayesian Score: The standard Laplacian-modified Bayesian score.
Mahalanobis Distance: The Mahalanobis distance (MD) is the distance to the center of the training data. The larger the MD, the less trustworthy the prediction.
Mahalanobis Distance p-value: The p-value gives the fraction of training data with an MD greater than or equal to the one for the given sample, assuming normally distributed data. The smaller the p-value, the less trustworthy the prediciton. For highly non-normal X properties (e.g., fingerprints), the MD p-value is wildly inaccurate.

Model Applicability

Unknown features are fingerprint features in the query molecule, but not found in the training set.

1. All properties and OPS components are within expected ranges.

Feature Contribution

Top features for positive contribution

Fingerprint	Bit/Smiles	Feature Structure	Score	Carcinogen in training set
FCFP_6	1175665944	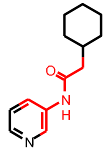	0.655	7 out of 12

FCFP_6	-55265897	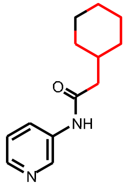<br>[*]CCCC(C)C	0.594	17 out of 34
FCFP_6	566058135	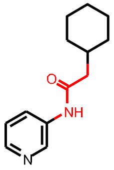<br>[*]NC(=O)C([*])[*]	0.447	17 out of 40

Top Features for negative contribution

**Fingerprint**	**Bit/Smiles**	**Feature Structure**	**Score**	**Carcinogen in training set**
FCFP_6	-1525101452	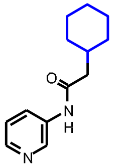<br>[*]C([*])CCCCCC(=[*])[*]	-1.133	0 out of 8
FCFP_6	1888947587	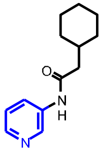<br>[*][c]1:[cH]:[cH]:[cH]:n:[cH]:1	-0.423	0 out of 2
FCFP_6	-1944142687	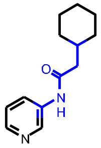<br>[*]C([*])CC(=O)N[c](:[*]):[*]	-0.423	0 out of 2

Indinavir

TOPKAT_Mouse_Male_FDA_None_vs_Carcinogen

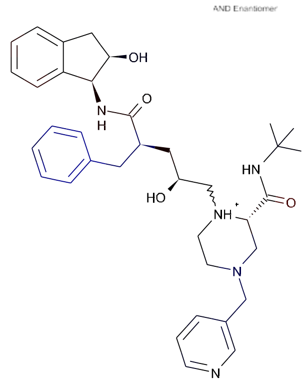

$C_{36}H_{48}N_5O_4$

Molecular Weight: 614.79741

ALogP: 1.521

Rotatable Bonds: 12

Acceptors: 6

Donors: 5

Model Prediction

Prediction: Non-Carcinogen

Probability: 0.175

Enrichment: 0.594

Bayesian Score: -6.747

Mahalanobis Distance: 16.618

Mahalanobis Distance p-value: 4.41e-011

Prediction: Positive if the Bayesian score is above the estimated best cutoff value from minimizing the false positive and false negative rate.
Probability: The esimated probability that the sample is in the positive category. This assumes that the Bayesian score follows a normal distribution and is different from the prediction using a cutoff.
Enrichment: An estimate of enrichment, that is, the increased likelihood (versus random) of this sample being in the category.
Bayesian Score: The standard Laplacian-modified Bayesian score.
Mahalanobis Distance: The Mahalanobis distance (MD) is the distance to the center of the training data. The larger the MD, the less trustworthy the prediction.
Mahalanobis Distance p-value: The p-value gives the fraction of training data with an MD greater than or equal to the one for the given sample, assuming normally distributed data. The smaller the p-value, the less trustworthy the prediciton. For highly non-normal X properties (e.g., fingerprints), the MD p-value is wildly inaccurate.

Structural Similar Compounds

Name	Pravastatin	Salmeterol	Glyburide
Structure	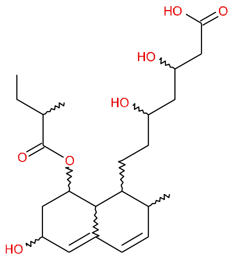	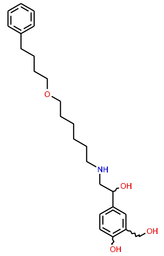	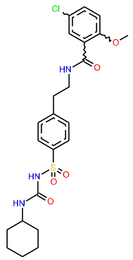
Actual Endpoint	Non-Carcinogen	Non-Carcinogen	Non-Carcinogen
Predicted Endpoint	Carcinogen	Non-Carcinogen	Non-Carcinogen
Distance	0.862	0.954	0.961
Reference	US FDA (Centre for Drug Eval.& Res./Off. Testing & Res.) Sept. 1997	US FDA (Centre for Drug Eval.& Res./Off. Testing & Res.) Sept. 1997	US FDA (Centre for Drug Eval.& Res./Off. Testing & Res.) Sept. 1997

Model Applicability

Unknown features are fingerprint features in the query molecule, but not found in the training set.

1. OPS PC10 out of range. Value: 5.4508. Training min, max, SD, explained variance: -4.2405, 5.172, 1.631, 0.0279.
2. Unknown FCFP_2 feature: 10: [*][NH+]([*])[*]
3. Unknown FCFP_2 feature: -1853714334: [*]C[NH+](C[*])C([*])[*]
4. Unknown FCFP_2 feature: -1817836174: [*]C[C@H]([NH+]([*])[*])C(=[*])[*]
5. Unknown FCFP_2 feature: 1155241219: [*]CC[NH+]([*])[*]

Feature Contribution

Top features for positive contribution

Fingerprint	Bit/Smiles	Feature Structure	Score	Carcinogen in training set
FCFP_6	-1943140669	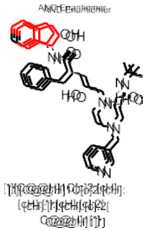	0.503	5 out of 10

FCFP_6	251901275	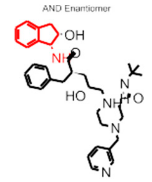 [*]N[C@@H]1[C@H]([*])C[c]2:[cH]:[cH]:[cH]:[cH]:[c]1:2	0.460	1 out of 1
FCFP_6	-550841003	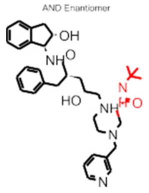 [*]C([*])C(=O)NC(C)(C)C	0.460	1 out of 1

Top Features for negative contribution

Fingerprint	Bit/Smiles	Feature Structure	Score	Carcinogen in training set
FCFP_6	1981711554	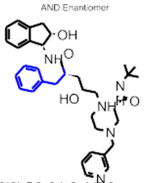 [*]C([*])C[c]1:[cH]:[cH]:[cH]:[cH]:[cH]:1	-1.424	0 out of 12
FCFP_6	-497728148	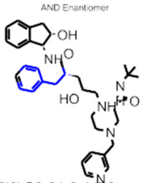 [*]C([*])C[c]1:[cH]:[cH]:[*]:[cH]:[cH]:1	-0.960	2 out of 26
FCFP_6	-1692123269	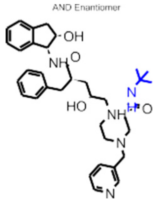 [*]C(=[*])NC(C)(C)C	-0.666	1 out of 11

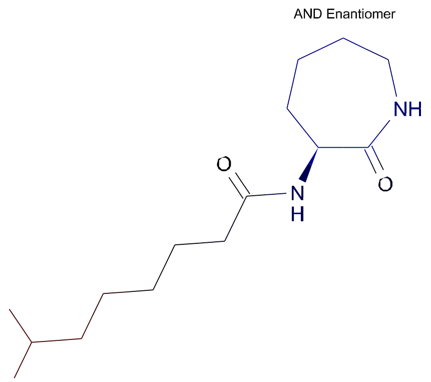

$C_{15}H_{28}N_2O_2$
Molecular Weight: 268.39501
ALogP: 2.685
Rotatable Bonds: 7
Acceptors: 2
Donors: 2

Model Prediction

Prediction: Multiple-Carcinogen
Probability: 0.181
Enrichment: 0.601
Bayesian Score: -3.783
Mahalanobis Distance: 10.212
Mahalanobis Distance p-value: 0.0131

Prediction: Positive if the Bayesian score is above the estimated best cutoff value from minimizing the false positive and false negative rate.
Probability: The esimated probability that the sample is in the positive category. This assumes that the Bayesian score follows a normal distribution and is different from the prediction using a cutoff.
Enrichment: An estimate of enrichment, that is, the increased likelihood (versus random) of this sample being in the category.
Bayesian Score: The standard Laplacian-modified Bayesian score.
Mahalanobis Distance: The Mahalanobis distance (MD) is the distance to the center of the training data. The larger the MD, the less trustworthy the prediction.
Mahalanobis Distance p-value: The p-value gives the fraction of training data with an MD greater than or equal to the one for the given sample, assuming normally distributed data. The smaller the p-value, the less trustworthy the prediciton. For highly non-normal X properties (e.g., fingerprints), the MD p-value is wildly inaccurate.

Structural Similar Compounds

Name	Prilocaine	Pronetalol	Cyclosporine
Structure	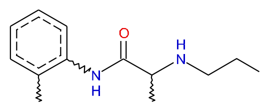	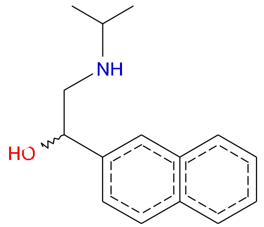	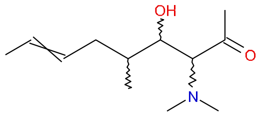
Actual Endpoint	Single-Carcinogen	Single-Carcinogen	Multiple-Carcinogen
Predicted Endpoint	Single-Carcinogen	Single-Carcinogen	Multiple-Carcinogen
Distance	0.605	0.691	0.714
Reference	US FDA (Centre for Drug Eval.& Res./Off. Testing & Res.) Sept. 1997	US FDA (Centre for Drug Eval.& Res./Off. Testing & Res.) Sept. 1997	US FDA (Centre for Drug Eval.& Res./Off. Testing & Res.) Sept. 1997

Model Applicability

Unknown features are fingerprint features in the query molecule, but not found in the training set.

1. All properties and OPS components are within expected ranges.

Feature Contribution

Top features for positive contribution

Fingerprint	Bit/Smiles	Feature Structure	Score	Multiple-Carcinogen in training set
FCFP_12	-1870530637	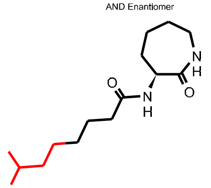 [*]CCC(C)C	0.400	1 out of 1

FCFP_12	-154166589	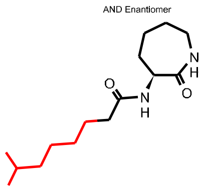<br>[*]CCCCC(C)C	0.400	1 out of 1
FCFP_12	-1043339860	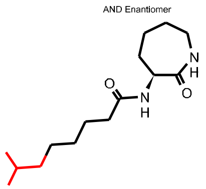<br>[*]CC(C)C	0.349	12 out of 24
**Top Features for negative contribution**				
**Fingerprint**	**Bit/Smiles**	**Feature Structure**	**Score**	**Multiple-Carcinogen in training set**
FCFP_12	354117335	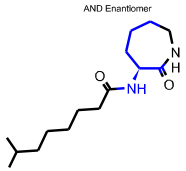<br>[*]N[C@H]1CCC[*][*]C1 =[*]	-0.859	0 out of 4
FCFP_12	566058135	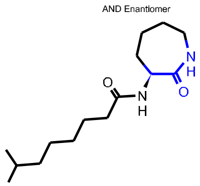<br>[*]NC(=O)C([*])[*]	-0.528	3 out of 17
FCFP_12	159404153	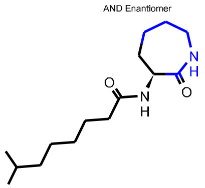<br>[*]=C1[*][*]CCCN1	-0.519	0 out of 2

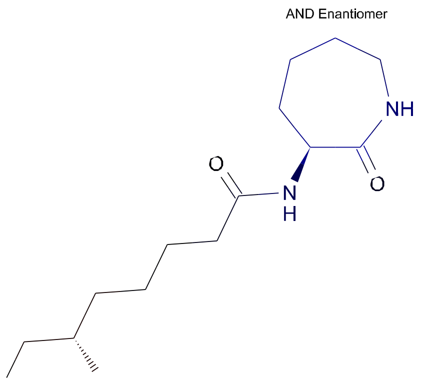

$C_{15}H_{28}N_2O_2$
Molecular Weight: 268.39501
ALogP: 2.685
Rotatable Bonds: 7
Acceptors: 2
Donors: 2

Structural Similar Compounds

Name	Prilocaine	Pronetalol	Cyclosporine
Structure	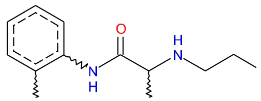	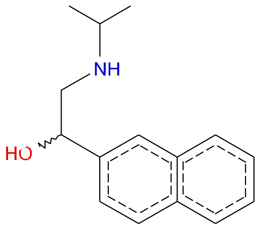	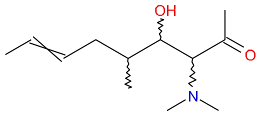
Actual Endpoint	Single-Carcinogen	Single-Carcinogen	Multiple-Carcinogen
Predicted Endpoint	Single-Carcinogen	Single-Carcinogen	Multiple-Carcinogen
Distance	0.606	0.692	0.715
Reference	US FDA (Centre for Drug Eval.& Res./Off. Testing & Res.) Sept. 1997	US FDA (Centre for Drug Eval.& Res./Off. Testing & Res.) Sept. 1997	US FDA (Centre for Drug Eval.& Res./Off. Testing & Res.) Sept. 1997

Model Prediction

Prediction: Multiple-Carcinogen
Probability: 0.172
Enrichment: 0.572
Bayesian Score: -4.477
Mahalanobis Distance: 10.212
Mahalanobis Distance p-value: 0.0131

Prediction: Positive if the Bayesian score is above the estimated best cutoff value from minimizing the false positive and false negative rate.
Probability: The esimated probability that the sample is in the positive category. This assumes that the Bayesian score follows a normal distribution and is different from the prediction using a cutoff.
Enrichment: An estimate of enrichment, that is, the increased likelihood (versus random) of this sample being in the category.
Bayesian Score: The standard Laplacian-modified Bayesian score.
Mahalanobis Distance: The Mahalanobis distance (MD) is the distance to the center of the training data. The larger the MD, the less trustworthy the prediction.
Mahalanobis Distance p-value: The p-value gives the fraction of training data with an MD greater than or equal to the one for the given sample, assuming normally distributed data. The smaller the p-value, the less trustworthy the prediciton. For highly non-normal X properties (e.g., fingerprints), the MD p-value is wildly inaccurate.

Model Applicability

Unknown features are fingerprint features in the query molecule, but not found in the training set.

1. All properties and OPS components are within expected ranges.

Feature Contribution

Top features for positive contribution

Fingerprint	Bit/Smiles	Feature Structure	Score	Multiple-Carcinogen in training set
FCFP_12	-1580903393	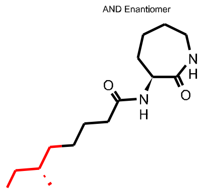 [*]C[C@H](C)CC	0.400	1 out of 1

FCFP_12	-1043339860	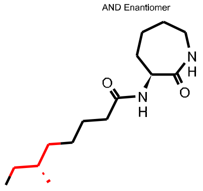 [*]CC(C)C	0.349	12 out of 24
FCFP_12	-55265897	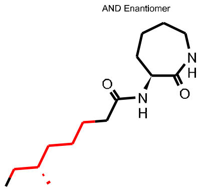 [*]CCCC(C)C	0.282	8 out of 17
**Top Features for negative contribution**				
**Fingerprint**	**Bit/Smiles**	**Feature Structure**	**Score**	**Multiple-Carcinogen in training set**
FCFP_12	354117335	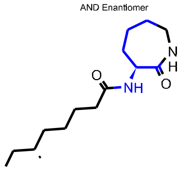 [*]N[C@H]1CCC[*][*]C1 =[*]	-0.859	0 out of 4
FCFP_12	566058135	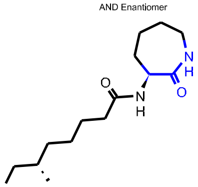 [*]NC(=O)C([*])[*]	-0.528	3 out of 17
FCFP_12	159404153	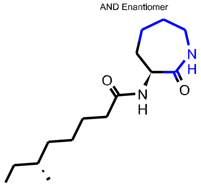 [*]=C1[*][*]CCCN1	-0.519	0 out of 2

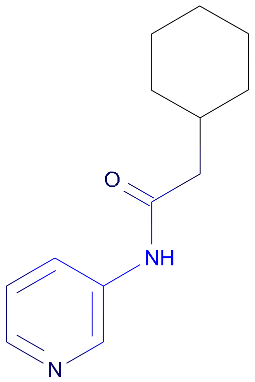

$C_{13}H_{18}N_2O$

Molecular Weight: 218.29481

ALogP: 2.171

Rotatable Bonds: 3

Acceptors: 2

Donors: 1

Structural Similar Compounds

Name	Phenacetin	Methylphenidate	Ripazepam
Structure	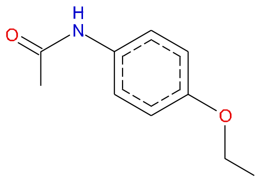	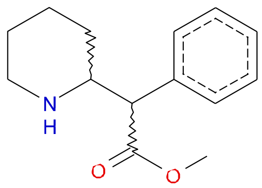	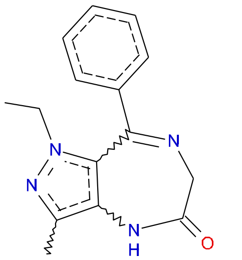
Actual Endpoint	Single-Carcinogen	Single-Carcinogen	Single-Carcinogen
Predicted Endpoint	Single-Carcinogen	Single-Carcinogen	Single-Carcinogen
Distance	0.520	0.576	0.599
Reference	US FDA (Centre for Drug Eval.& Res./Off. Testing & Res.) Sept. 1997	US FDA (Centre for Drug Eval.& Res./Off. Testing & Res.) Sept. 1997	US FDA (Centre for Drug Eval.& Res./Off. Testing & Res.) Sept. 1997

Model Prediction

Prediction: Single-Carcinogen

Probability: 0.146

Enrichment: 0.485

Bayesian Score: -8.763

Mahalanobis Distance: 12.383

Mahalanobis Distance p-value: 0.000303

Prediction: Positive if the Bayesian score is above the estimated best cutoff value from minimizing the false positive and false negative rate.
Probability: The esimated probability that the sample is in the positive category. This assumes that the Bayesian score follows a normal distribution and is different from the prediction using a cutoff.
Enrichment: An estimate of enrichment, that is, the increased likelihood (versus random) of this sample being in the category.
Bayesian Score: The standard Laplacian-modified Bayesian score.
Mahalanobis Distance: The Mahalanobis distance (MD) is the distance to the center of the training data. The larger the MD, the less trustworthy the prediction.
Mahalanobis Distance p-value: The p-value gives the fraction of training data with an MD greater than or equal to the one for the given sample, assuming normally distributed data. The smaller the p-value, the less trustworthy the prediciton. For highly non-normal X properties (e.g., fingerprints), the MD p-value is wildly inaccurate.

Model Applicability

Unknown features are fingerprint features in the query molecule, but not found in the training set.

1. All properties and OPS components are within expected ranges.

Feature Contribution

Top features for positive contribution

Fingerprint	Bit/Smiles	Feature Structure	Score	Multiple-Carcinogen in training set
FCFP_12	547884906	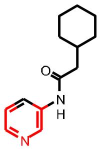 [*][c]1:[*]:[cH]:[cH]:n:[cH]:1	0.400	1 out of 1

FCFP_12	-1043339860	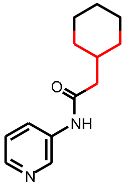 [*]CC(C)C	0.349	12 out of 24
FCFP_12	-55265897	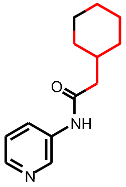 [*]CCCC(C)C	0.282	8 out of 17
**Top Features for negative contribution**				
**Fingerprint**	**Bit/Smiles**	**Feature Structure**	**Score**	**Multiple-Carcinogen in training set**
FCFP_12	1294255210	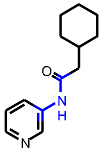 [*]C(=[*])N[c](:[*]):[*]	-1.626	0 out of 12
FCFP_12	1175665944	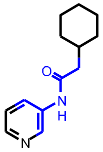 [*]CC(=O)N[c](:[cH]:[*]):[cH]:[*]	-1.219	0 out of 7
FCFP_12	590925877	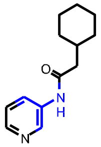 [*]N[c](:[cH]:[*]):[cH]:[*]	-0.998	1 out of 13

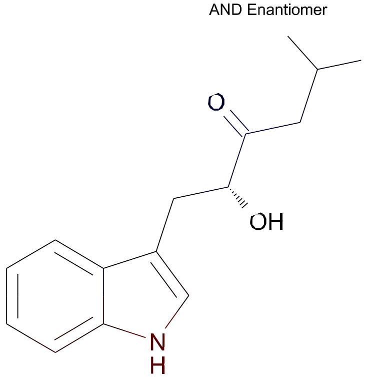

$C_{15}H_{19}NO_2$
Molecular Weight: 245.31685
ALogP: 2.932
Rotatable Bonds: 5
Acceptors: 2
Donors: 2

Structural Similar Compounds

Name	FLUORENE-9;9-(BIS)PROPYLAMINE	ANILINE;4;4'-METHYLENEBIS(2-METHYL-	BENZOIN; OXIME
Structure	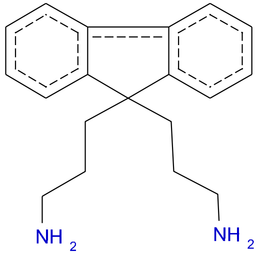	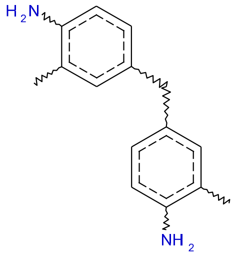	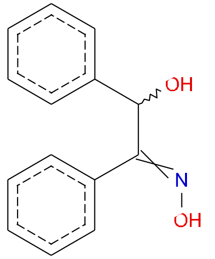
Actual Endpoint	Moderate_Severe	Moderate_Severe	Mild
Predicted Endpoint	Moderate_Severe	Moderate_Severe	Mild
Distance	0.530	0.566	0.571
Reference	IHFCAY 6;1;67	28ZPAK-;72;72	28ZPAK-;111;72

Model Prediction

Prediction: Moderate_Severe
Probability: 0.817
Enrichment: 1.186
Bayesian Score: -0.256
Mahalanobis Distance: 9.311
Mahalanobis Distance p-value: 0.347

Prediction: Positive if the Bayesian score is above the estimated best cutoff value from minimizing the false positive and false negative rate.
Probability: The esimated probability that the sample is in the positive category. This assumes that the Bayesian score follows a normal distribution and is different from the prediction using a cutoff.
Enrichment: An estimate of enrichment, that is, the increased likelihood (versus random) of this sample being in the category.
Bayesian Score: The standard Laplacian-modified Bayesian score.
Mahalanobis Distance: The Mahalanobis distance (MD) is the distance to the center of the training data. The larger the MD, the less trustworthy the prediction.
Mahalanobis Distance p-value: The p-value gives the fraction of training data with an MD greater than or equal to the one for the given sample, assuming normally distributed data. The smaller the p-value, the less trustworthy the prediciton. For highly non-normal X properties (e.g., fingerprints), the MD p-value is wildly inaccurate.

Model Applicability

Unknown features are fingerprint features in the query molecule, but not found in the training set.

1. All properties and OPS components are within expected ranges.

Feature Contribution

Top features for positive contribution

Fingerprint	Bit/Smiles	Feature Structure	Score	Moderate_Severe in training set
FCFP_10	1673997923	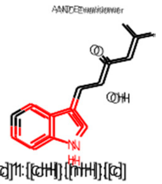 [*][c]1:[cH]:[nH]:[c]2:[cH]:[cH]:[*]:[cH]:[c]:1:2	0.256	2 out of 2

FCFP_10	155061250	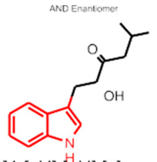 [*][c]1:[cH]:[nH]:[c]2:[cH]:[cH]:[cH]:[cH]:[c]:1:2	0.256	2 out of 2
FCFP_10	1070061035	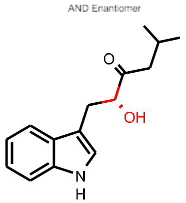 [*]C([*])O	0.239	284 out of 338
**Top Features for negative contribution**				
**Fingerprint**	**Bit/Smiles**	**Feature Structure**	**Score**	**Moderate_Severe in training set**
FCFP_10	1306984497	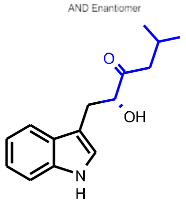 [*]C([*])C(=O)CC(C)C	-0.507	0 out of 1
FCFP_10	-2006448698	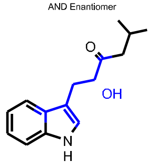 [*]C(=[*])[C@H](O)C[c]1:[cH]:[*]:[*]:[c]:1:[*]	-0.507	0 out of 1
FCFP_10	565968762	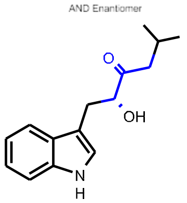 [*]CC(=O)C([*])[*]	-0.372	17 out of 38

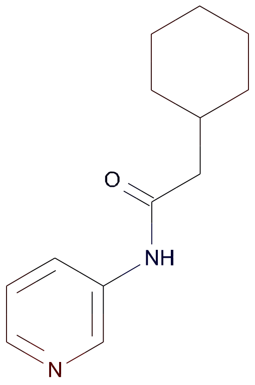

$C_{13}H_{18}N_2O$

Molecular Weight: 218.29481

ALogP: 2.171

Rotatable Bonds: 3

Acceptors: 2

Donors: 1

Structural Similar Compounds

Name	CARBAMIC ACID; METHYL-; 1-NAPHTHYL ESTER	Ethanol; 2-(2;4-dichlorophenoxy)-	ANILINE;P-PHENOXY-
Structure	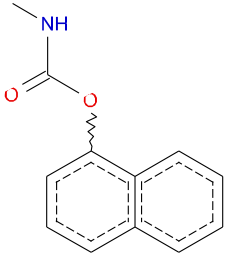	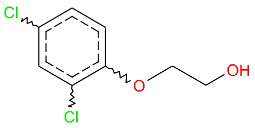	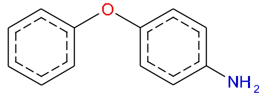
Actual Endpoint	Mild	Moderate_Severe	Moderate_Severe
Predicted Endpoint	Mild	Moderate_Severe	Mild
Distance	0.513	0.528	0.535
Reference	28ZPAK-;164;72	Prehled Prumyslove Toxikologie; Organicke Latky; Marhold; J. -;530;86	28ZPAK-;119;72

Model Prediction

Prediction: Moderate_Severe

Probability: 0.837

Enrichment: 1.214

Bayesian Score: 1.044

Mahalanobis Distance: 9.086

Mahalanobis Distance p-value: 0.465

Prediction: Positive if the Bayesian score is above the estimated best cutoff value from minimizing the false positive and false negative rate.
Probability: The esimated probability that the sample is in the positive category. This assumes that the Bayesian score follows a normal distribution and is different from the prediction using a cutoff.
Enrichment: An estimate of enrichment, that is, the increased likelihood (versus random) of this sample being in the category.
Bayesian Score: The standard Laplacian-modified Bayesian score.
Mahalanobis Distance: The Mahalanobis distance (MD) is the distance to the center of the training data. The larger the MD, the less trustworthy the prediction.
Mahalanobis Distance p-value: The p-value gives the fraction of training data with an MD greater than or equal to the one for the given sample, assuming normally distributed data. The smaller the p-value, the less trustworthy the prediciton. For highly non-normal X properties (e.g., fingerprints), the MD p-value is wildly inaccurate.

Model Applicability

Unknown features are fingerprint features in the query molecule, but not found in the training set.

1. All properties and OPS components are within expected ranges.

Feature Contribution

Top features for positive contribution

Fingerprint	Bit/Smiles	Feature Structure	Score	Moderate_Severe in training set
FCFP_10	547884906	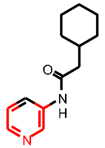 [*][c]1:[*]:[cH]:[cH]:n:[cH]:1	0.317	4 out of 4

FCFP_10	-1695756380	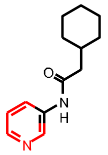 [*]1:[cH]:[cH]:[cH]:n :[cH]:1	0.285	10 out of 11
FCFP_10	1940464803	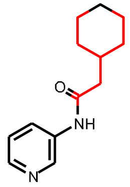 [*]C(=[*])CC1CC[*]CC1	0.285	10 out of 11
**Top Features for negative contribution**				
**Fingerprint**	**Bit/Smiles**	**Feature Structure**	**Score**	**Moderate_Severe in training set**
FCFP_10	-1944142687	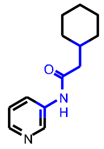 [*]C([*])CC(=O)N[c](: [*]):[*]	-0.507	0 out of 1
FCFP_10	-773983804	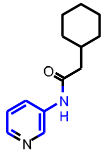 [*]N[c]1:[cH]:[*]:[cH]:[cH]:[cH]:1	-0.294	50 out of 102
FCFP_10	1294255210	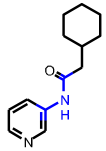 [*]C(=[*])N[c](:[*]): [*]	-0.218	20 out of 38

Indinavir **TOPKAT_Ocular_Irritancy_Mild_vs_Moderate_Severe**

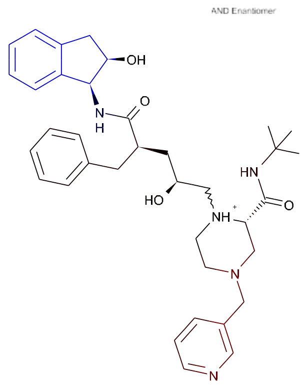

$C_{36}H_{48}N_5O_4$
Molecular Weight: 614.79741
ALogP: 1.521
Rotatable Bonds: 12
Acceptors: 6
Donors: 5

Structural Similar Compounds

Name	2-Naphthalenesulfonic acid; 5;6'-iminobis(1-hydroxy-	ANTHRAQUINONE; 1;5-DIAMINO-4;8-DIHYDROXY-3-(p-METHOXYPHENYL)-	4;4'-DIAMINO-1;1'-DIANTHRIMIDE
Structure	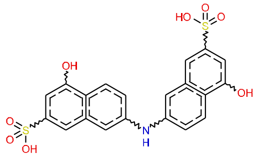	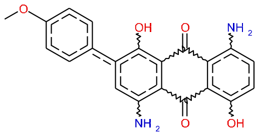	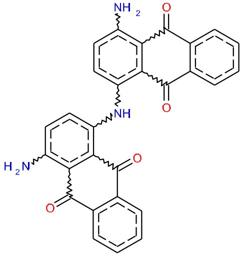
Actual Endpoint	Mild	Mild	Mild
Predicted Endpoint	Mild	Mild	Mild
Distance	1.091	1.108	1.149
Reference	Prehled Prumyslove Toxikologie; Organicke Latky; Marhold; J. pp 1065;86	28ZPAK 245;72	28ZPAK-;125;72

Model Prediction

Prediction: Moderate_Severe
Probability: 0.833
Enrichment: 1.209
Bayesian Score: 0.740
Mahalanobis Distance: 17.365
Mahalanobis Distance p-value: 5.42e-023

Prediction: Positive if the Bayesian score is above the estimated best cutoff value from minimizing the false positive and false negative rate.
Probability: The esimated probability that the sample is in the positive category. This assumes that the Bayesian score follows a normal distribution and is different from the prediction using a cutoff.
Enrichment: An estimate of enrichment, that is, the increased likelihood (versus random) of this sample being in the category.
Bayesian Score: The standard Laplacian-modified Bayesian score.
Mahalanobis Distance: The Mahalanobis distance (MD) is the distance to the center of the training data. The larger the MD, the less trustworthy the prediction.
Mahalanobis Distance p-value: The p-value gives the fraction of training data with an MD greater than or equal to the one for the given sample, assuming normally distributed data. The smaller the p-value, the less trustworthy the prediciton. For highly non-normal X properties (e.g., fingerprints), the MD p-value is wildly inaccurate.

Model Applicability

Unknown features are fingerprint features in the query molecule, but not found in the training set.

1. All properties and OPS components are within expected ranges.
2. Unknown FCFP_2 feature: 10: [*][NH+]([*])[*]
3. Unknown FCFP_2 feature: -1853714334: [*]C[NH+](C[*])C([*])[*]
4. Unknown FCFP_2 feature: -1817836174: [*]C[C@H]([NH+]([*])[*])C(=[*])[*]
5. Unknown FCFP_2 feature: 1155241219: [*]CC[NH+]([*])[*]

Feature Contribution

Top features for positive contribution

Fingerprint	Bit/Smiles	Feature Structure	Score	Moderate_Severe in training set

FCFP_10	-497728148	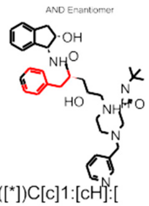 [*]C([*])C[c]1:[cH]:[<br>cH]:[*]:[cH]:[cH]:1	0.356	24 out of 25
FCFP_10	906798516	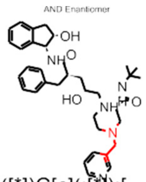 [*]N([*])C[c](:[*]):[<br>*]	0.344	6 out of 6
FCFP_10	-587569116	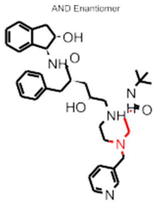 [*]C([*])CN([*])[*]	0.335	66 out of 71
**Top Features for negative contribution**				
**Fingerprint**	**Bit/Smiles**	**Feature Structure**	**Score**	**Moderate_Severe in training set**
FCFP_10	-1947828591	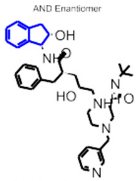 [*][C@@H]1C[c]2:[cH]:<br>[cH]:[cH]:[cH]:[c]:2<br>[C@@H]1[*]	-1.460	0 out of 5
FCFP_10	-1943140669	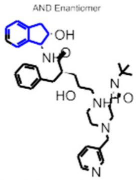 [*][C@@H]1C[c]2:[cH]:<br>[cH]:[*]:[cH]:[c]:2[<br>C@@H]1[*]	-0.629	2 out of 7

FCFP_10	251901275	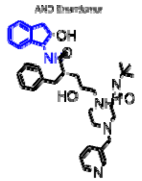 [*]N[C@@H]1[C@H]([*])C[c]2:[cH]:[cH]:[cH]:[cH]:[c]1 2	-	0 out of 1

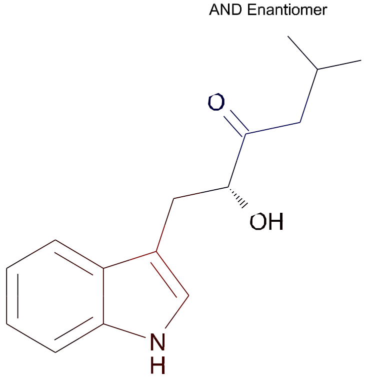

$C_{15}H_{19}NO_2$
Molecular Weight: 245.31685
ALogP: 2.932
Rotatable Bonds: 5
Acceptors: 2
Donors: 2

Model Prediction

Prediction: Severe
Probability: 0.685
Enrichment: 1.106
Bayesian Score: -0.174
Mahalanobis Distance: 9.288
Mahalanobis Distance p-value: 0.211

Prediction: Positive if the Bayesian score is above the estimated best cutoff value from minimizing the false positive and false negative rate.
Probability: The esimated probability that the sample is in the positive category. This assumes that the Bayesian score follows a normal distribution and is different from the prediction using a cutoff.
Enrichment: An estimate of enrichment, that is, the increased likelihood (versus random) of this sample being in the category.
Bayesian Score: The standard Laplacian-modified Bayesian score.
Mahalanobis Distance: The Mahalanobis distance (MD) is the distance to the center of the training data. The larger the MD, the less trustworthy the prediction.
Mahalanobis Distance p-value: The p-value gives the fraction of training data with an MD greater than or equal to the one for the given sample, assuming normally distributed data. The smaller the p-value, the less trustworthy the prediciton. For highly non-normal X properties (e.g., fingerprints), the MD p-value is wildly inaccurate.

Structural Similar Compounds

Name	FLUORENE-9;9-(BIS)PROPYLAMINE	ANILINE;4;4'-METHYLENEBIS(2-METHYL-	2-BIPHENYLCARBOXYLIC ACID; 2'-HYDROXYMETHYL-
Structure	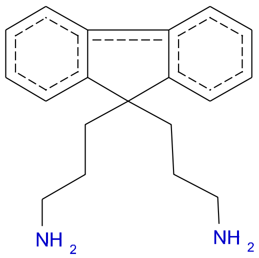	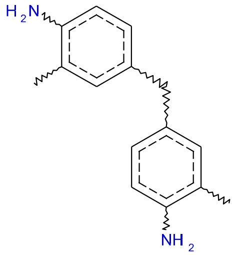	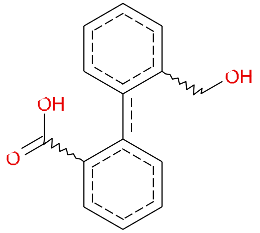
Actual Endpoint	Severe	Moderate	Moderate
Predicted Endpoint	Severe	Moderate	Moderate
Distance	0.550	0.568	0.582
Reference	IHFCAY 6;1;67	28ZPAK-;72;72	IHFCAY 6;1;67

Model Applicability

Unknown features are fingerprint features in the query molecule, but not found in the training set.

1. All properties and OPS components are within expected ranges.

Feature Contribution

Top features for positive contribution				
**Fingerprint**	**Bit/Smiles**	**Feature Structure**	**Score**	**Severe in training set**
SCFP_12	-496201075	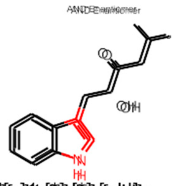 [*][c]1:[*]:[*]:[nH]:[cH]:1	0.378	12 out of 13

SCFP_12	-673674794	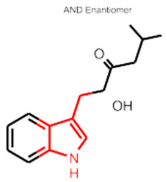<br>[*]C[c]1:[cH]:[nH]:[c<br>](:[*]):[c]:1:[*]	0.376	4 out of 4
SCFP_12	1188101983	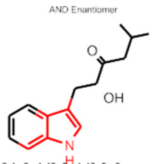<br>[*][c]1:[cH]:[nH]:[c]<br>(:[cH]:[*]):[c]:1:[*<br>]	0.303	2 out of 2
**Top Features for negative contribution**				
**Fingerprint**	**Bit/Smiles**	**Feature Structure**	**Score**	**Severe in training set**
SCFP_12	571765461	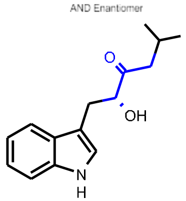<br>[*]CC(=O)C([*])[*]	-1.017	2 out of 12
SCFP_12	-1873554935	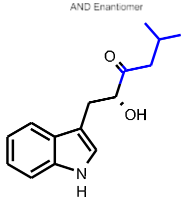<br>[*]C(=[*])CC(C)C	-0.475	0 out of 1
SCFP_12	112554633	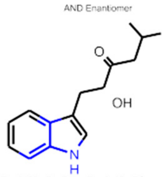<br>[*]:[cH]:[c]1:[nH]:[*<br>]:[*]:[c]:1:[*]	-0.324	5 out of 12

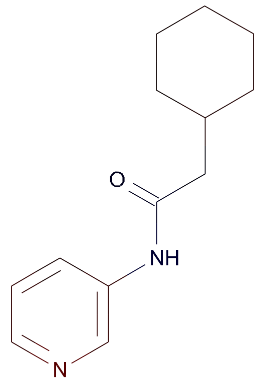

$C_{13}H_{18}N_2O$
Molecular Weight: 218.29481
ALogP: 2.171
Rotatable Bonds: 3
Acceptors: 2
Donors: 1

Structural Similar Compounds

Name	1-NAPHTHALENEACETIC ACID	Ethanol; 2-(2;4-dichlorophenoxy)-	ETHANOL;2-(P-CHLOROPHENOXY)-
Structure	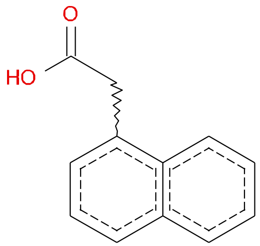	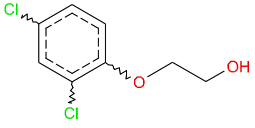	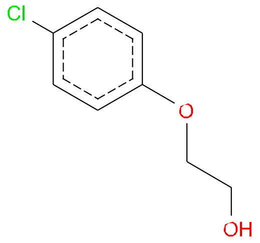
Actual Endpoint	Severe	Severe	Severe
Predicted Endpoint	Severe	Severe	Severe
Distance	0.530	0.545	0.563
Reference	PESTC* 9;10;80	Prehled Prumyslove Toxikologie; Organicke Latky; Marhold; J. -;530;86	28ZPAK-;81;72

Model Prediction

Prediction: Severe
Probability: 0.727
Enrichment: 1.173
Bayesian Score: 1.213
Mahalanobis Distance: 12.097
Mahalanobis Distance p-value: 1.02e-005

Prediction: Positive if the Bayesian score is above the estimated best cutoff value from minimizing the false positive and false negative rate.
Probability: The esimated probability that the sample is in the positive category. This assumes that the Bayesian score follows a normal distribution and is different from the prediction using a cutoff.
Enrichment: An estimate of enrichment, that is, the increased likelihood (versus random) of this sample being in the category.
Bayesian Score: The standard Laplacian-modified Bayesian score.
Mahalanobis Distance: The Mahalanobis distance (MD) is the distance to the center of the training data. The larger the MD, the less trustworthy the prediction.
Mahalanobis Distance p-value: The p-value gives the fraction of training data with an MD greater than or equal to the one for the given sample, assuming normally distributed data. The smaller the p-value, the less trustworthy the prediciton. For highly non-normal X properties (e.g., fingerprints), the MD p-value is wildly inaccurate.

Model Applicability

Unknown features are fingerprint features in the query molecule, but not found in the training set.

1. All properties and OPS components are within expected ranges.

Feature Contribution

Top features for positive contribution

Fingerprint	Bit/Smiles	Feature Structure	Score	Severe in training set
SCFP_12	-937094999	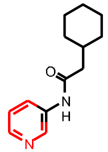 [*]1:[cH]:[cH]:[cH]:n:[cH]:1	0.420	7 out of 7

SCFP_12	-496201075	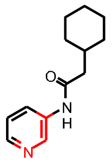 [*][c]1:[*]:[*]:[nH]:[cH]:1	0.378	12 out of 13
SCFP_12	1188429584	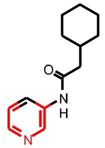 [*][c]1:[*]:[cH]:[cH]:n:[cH]:1	0.376	4 out of 4
**Top Features for negative contribution**				
**Fingerprint**	**Bit/Smiles**	**Feature Structure**	**Score**	**Severe in training set**
SCFP_12	1256995004	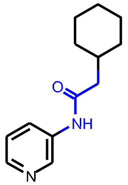 [*]CC(=O)N[*]	-0.483	12 out of 33
SCFP_12	9	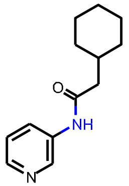 [*]N[*]	-0.315	29 out of 66
SCFP_12	-1375926917	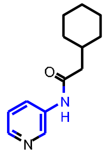 [*]N[c]1:[cH]:[*]:[cH]:[cH]:[cH]:1	-0.248	5 out of 11

Indinavir

TOPKAT_Ocular_Irritancy_Moderate_vs_Severe

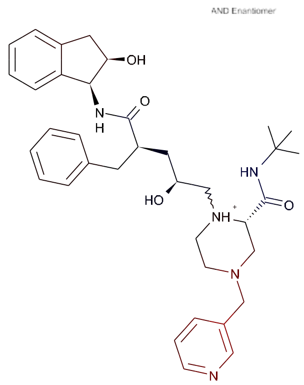

$C_{36}H_{48}N_5O_4$
Molecular Weight: 614.79741
ALogP: 1.521
Rotatable Bonds: 12
Acceptors: 6
Donors: 5

Model Prediction

Prediction: Severe
Probability: 0.794
Enrichment: 1.281
Bayesian Score: 3.262
Mahalanobis Distance: 16.330
Mahalanobis Distance p-value: 7.05e-019

Prediction: Positive if the Bayesian score is above the estimated best cutoff value from minimizing the false positive and false negative rate.
Probability: The esimated probability that the sample is in the positive category. This assumes that the Bayesian score follows a normal distribution and is different from the prediction using a cutoff.
Enrichment: An estimate of enrichment, that is, the increased likelihood (versus random) of this sample being in the category.
Bayesian Score: The standard Laplacian-modified Bayesian score.
Mahalanobis Distance: The Mahalanobis distance (MD) is the distance to the center of the training data. The larger the MD, the less trustworthy the prediction.
Mahalanobis Distance p-value: The p-value gives the fraction of training data with an MD greater than or equal to the one for the given sample, assuming normally distributed data. The smaller the p-value, the less trustworthy the prediciton. For highly non-normal X properties (e.g., fingerprints), the MD p-value is wildly inaccurate.

Structural Similar Compounds

Name	p-Acetophenetidide; 3'-(bis(2-hydroxyethyl)amino)-	2;7-NAPHTHALENE DISULFONIC ACID;4-AMINO-5-HYDROXY-;P-TOLUENE SULFONATE (ESTER)	METHANE;TRIS(4-AMINOPHENYL)-
Structure	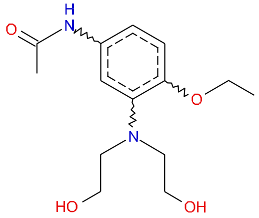	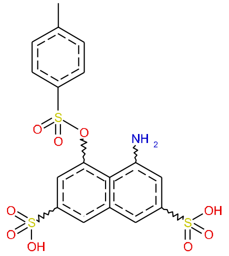	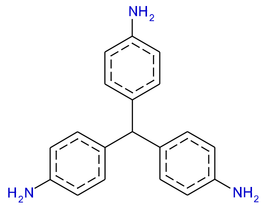
Actual Endpoint	Moderate	Moderate	Moderate
Predicted Endpoint	Moderate	Moderate	Moderate
Distance	1.241	1.295	1.320
Reference	Prehled Prumyslove Toxikologie; Organicke Latky; Marhold; J. -;645;86	28ZPAK-;194;72	28ZPAK-;73;72

Model Applicability

Unknown features are fingerprint features in the query molecule, but not found in the training set.

1. All properties and OPS components are within expected ranges.

Feature Contribution

Top features for positive contribution

Fingerprint	Bit/Smiles	Feature Structure	Score	Severe in training set
SCFP_12	2088794301	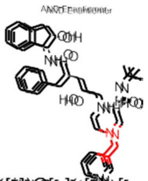 [*]N([*])C[c](:[*]):[*]	0.420	7 out of 7

SCFP_12	-937094999	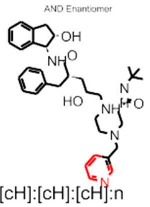 [*]1:[cH]:[cH]:[cH]:n:[cH]:1	0.420	7 out of 7
SCFP_12	1453622480	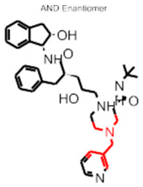 [*]CN(C[*])C[c](:[cH]:[*]):[cH]:[*]	0.396	5 out of 5
**Top Features for negative contribution**				
**Fingerprint**	**Bit/Smiles**	**Feature Structure**	**Score**	**Severe in training set**
SCFP_12	1256995004	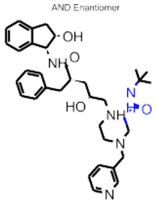 [*]CC(=O)N[*]	-0.483	12 out of 33
SCFP_12	-1903488337	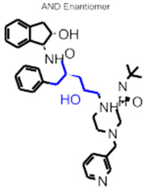 [*]C[C@@H](O)C[C@@H](C[*])C(=[*])[*]	-0.475	0 out of 1
SCFP_12	-111024397	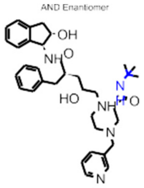 [*]C(=[*])NC([*])([*])[*]	-0.345	1 out of 3

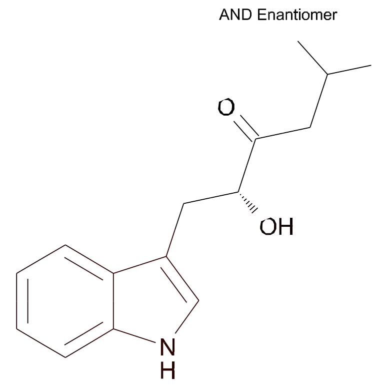

$C_{15}H_{19}NO_2$
Molecular Weight: 245.31685
ALogP: 2.932
Rotatable Bonds: 5
Acceptors: 2
Donors: 2

Structural Similar Compounds

Name	FLUORENE-9;9-(BIS)PROPYLAMINE	ANILINE;4;4'-METHYLENEBIS(2-METHYL-	BENZOIN; OXIME
Structure	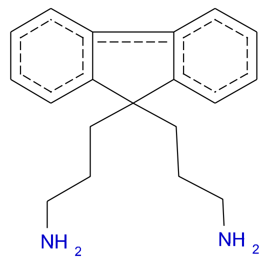	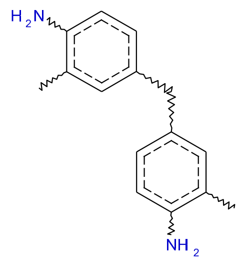	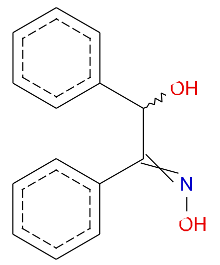
Actual Endpoint	Irritant	Irritant	Irritant
Predicted Endpoint	Irritant	Irritant	Irritant
Distance	0.526	0.557	0.565
Reference	IHFCAY 6;1;67	28ZPAK-;72;72	28ZPAK-;111;72

Model Applicability

Unknown features are fingerprint features in the query molecule, but not found in the training set.

1. All properties and OPS components are within expected ranges.

Model Prediction

Prediction: Irritant
Probability: 1.000
Enrichment: 1.175
Bayesian Score: 1.191
Mahalanobis Distance: 7.466
Mahalanobis Distance p-value: 0.987

Prediction: Positive if the Bayesian score is above the estimated best cutoff value from minimizing the false positive and false negative rate.
Probability: The esimated probability that the sample is in the positive category. This assumes that the Bayesian score follows a normal distribution and is different from the prediction using a cutoff.
Enrichment: An estimate of enrichment, that is, the increased likelihood (versus random) of this sample being in the category.
Bayesian Score: The standard Laplacian-modified Bayesian score.
Mahalanobis Distance: The Mahalanobis distance (MD) is the distance to the center of the training data. The larger the MD, the less trustworthy the prediction.
Mahalanobis Distance p-value: The p-value gives the fraction of training data with an MD greater than or equal to the one for the given sample, assuming normally distributed data. The smaller the p-value, the less trustworthy the prediciton. For highly non-normal X properties (e.g., fingerprints), the MD p-value is wildly inaccurate.

Feature Contribution

Top features for positive contribution				
Fingerprint	Bit/Smiles	Feature Structure	Score	Irritant in training set
FCFP_12	2005402822	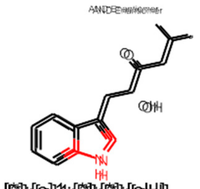 [*]:[c]1:[*]:[*]:[cH]:[nH]:1	0.175	5 out of 5

FCFP_12	-1192617147	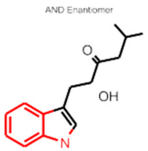 [*]1:[*]:[c]2:[cH]:[cH]:[cH]:[cH]:[c]:2:[nH]:1	0.156	3 out of 3
FCFP_12	155061250	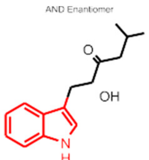 [*][c]1:[cH]:[nH]:[c]2:[cH]:[cH]:[cH]:[cH]:[c]:1:2	0.137	2 out of 2
**Top Features for negative contribution**				
**Fingerprint**	**Bit/Smiles**	**Feature Structure**	**Score**	**Irritant in training set**
FCFP_12	1306984497	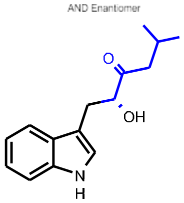 [*]C([*])C(=O)CC(C)C	-0.268	1 out of 2
FCFP_12	-1870530637	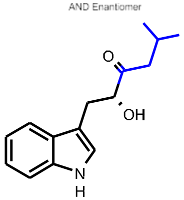 [*]C(=[*])CC(C)C	-0.070	18 out of 24
FCFP_12	0	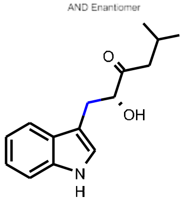 [*]C[*]	0.000	1184 out of 1397

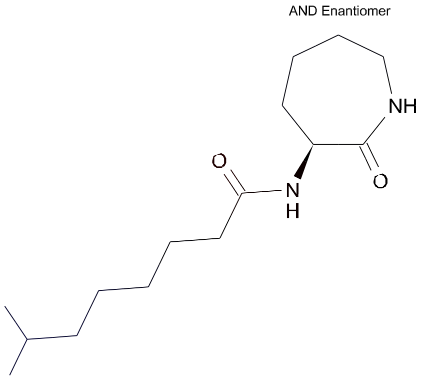

$C_{15}H_{28}N_2O_2$
Molecular Weight: 268.39501
ALogP: 2.685
Rotatable Bonds: 7
Acceptors: 2
Donors: 2

Model Prediction

Prediction: Non-Irritant
Probability: 0.974
Enrichment: 1.145
Bayesian Score: -0.419
Mahalanobis Distance: 11.124
Mahalanobis Distance p-value: 0.00282

Prediction: Positive if the Bayesian score is above the estimated best cutoff value from minimizing the false positive and false negative rate.
Probability: The esimated probability that the sample is in the positive category. This assumes that the Bayesian score follows a normal distribution and is different from the prediction using a cutoff.
Enrichment: An estimate of enrichment, that is, the increased likelihood (versus random) of this sample being in the category.
Bayesian Score: The standard Laplacian-modified Bayesian score.
Mahalanobis Distance: The Mahalanobis distance (MD) is the distance to the center of the training data. The larger the MD, the less trustworthy the prediction.
Mahalanobis Distance p-value: The p-value gives the fraction of training data with an MD greater than or equal to the one for the given sample, assuming normally distributed data. The smaller the p-value, the less trustworthy the prediciton. For highly non-normal X properties (e.g., fingerprints), the MD p-value is wildly inaccurate.

Structural Similar Compounds

Name	FLUORENE-9;9-(BIS)PROPYLAMINE	ETHYLENEDIAMINE;N;N' BIS(ALPHA-METHYLBENZYL)-	BENZOIN; OXIME
Structure	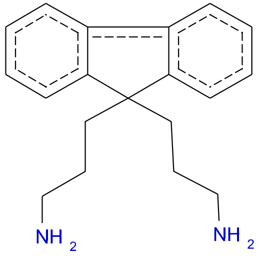	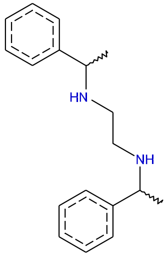	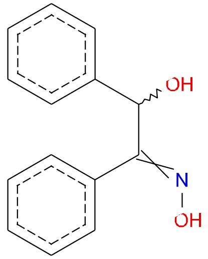
Actual Endpoint	Irritant	Irritant	Irritant
Predicted Endpoint	Irritant	Irritant	Irritant
Distance	0.511	0.630	0.645
Reference	IHFCAY 6;1;67	AMIHBC 10;61;54	28ZPAK-;111;72

Model Applicability

Unknown features are fingerprint features in the query molecule, but not found in the training set.

1. All properties and OPS components are within expected ranges.

Feature Contribution

Top features for positive contribution				
**Fingerprint**	**Bit/Smiles**	**Feature Structure**	**Score**	**Irritant in training set**
FCFP_12	-885550502	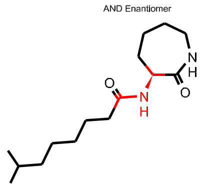 [*]C([*])NC(=[*])[*]	0.180	64 out of 66

FCFP_12	566058135	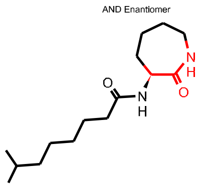 [*]NC(=O)C([*])[*]	0.163	23 out of 24
FCFP_12	494226440	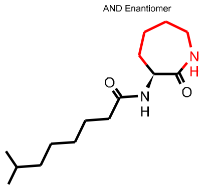 [*]1[*]NCCCC1	0.150	33 out of 35
**Top Features for negative contribution**				
**Fingerprint**	**Bit/Smiles**	**Feature Structure**	**Score**	**Irritant in training set**
FCFP_12	618627883	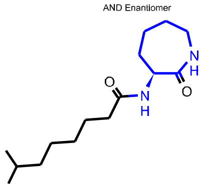 [*]N[C@H]1CCCCNC1=[*]	-0.592	0 out of 1
FCFP_12	-154166589	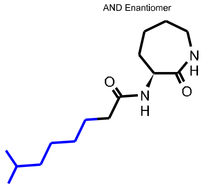 [*]CCCCC(C)C	-0.344	2 out of 4
FCFP_12	-278115048	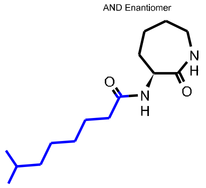 [*]C(=[*])CCCCCC(C)C	-0.268	1 out of 2

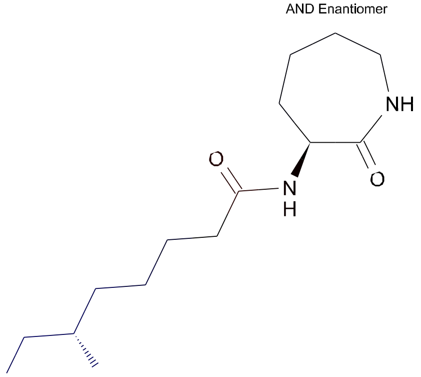

$C_{15}H_{28}N_2O_2$
Molecular Weight: 268.39501
ALogP: 2.685
Rotatable Bonds: 7
Acceptors: 2
Donors: 2

Structural Similar Compounds

Name	FLUORENE-9;9-(BIS)PROPYLAMINE	ETHYLENEDIAMINE;N;N' BIS(ALPHA-METHYLBENZYL)-	2-HYDROXY-3-ETHYL HEPTANOIC ACID
Structure	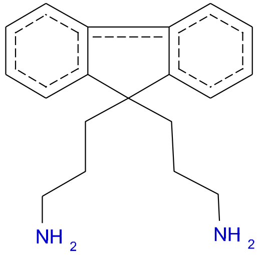	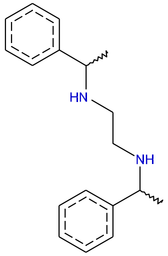	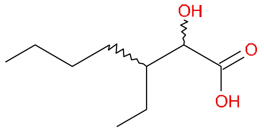
Actual Endpoint	Irritant	Irritant	Irritant
Predicted Endpoint	Irritant	Irritant	Irritant
Distance	0.512	0.630	0.642
Reference	IHFCAY 6;1;67	AMIHBC 10;61;54	AIHAAP 23;95;62

Model Prediction

Prediction: Non-Irritant
Probability: 0.970
Enrichment: 1.140
Bayesian Score: -1.195
Mahalanobis Distance: 12.927
Mahalanobis Distance p-value: 3.23e-007

Prediction: Positive if the Bayesian score is above the estimated best cutoff value from minimizing the false positive and false negative rate.
Probability: The esimated probability that the sample is in the positive category. This assumes that the Bayesian score follows a normal distribution and is different from the prediction using a cutoff.
Enrichment: An estimate of enrichment, that is, the increased likelihood (versus random) of this sample being in the category.
Bayesian Score: The standard Laplacian-modified Bayesian score.
Mahalanobis Distance: The Mahalanobis distance (MD) is the distance to the center of the training data. The larger the MD, the less trustworthy the prediction.
Mahalanobis Distance p-value: The p-value gives the fraction of training data with an MD greater than or equal to the one for the given sample, assuming normally distributed data. The smaller the p-value, the less trustworthy the prediciton. For highly non-normal X properties (e.g., fingerprints), the MD p-value is wildly inaccurate.

Model Applicability

Unknown features are fingerprint features in the query molecule, but not found in the training set.

1. All properties and OPS components are within expected ranges.

Feature Contribution

Top features for positive contribution

Fingerprint	Bit/Smiles	Feature Structure	Score	Irritant in training set
FCFP_12	-885550502	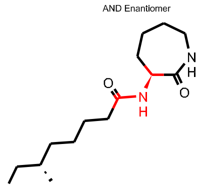 [*]C([*])NC(=[*])[*]	0.180	64 out of 66

FCFP_12	566058135	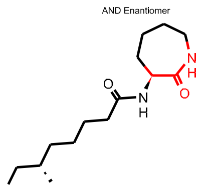 [*]NC(=O)C([*])[*]	0.163	23 out of 24
FCFP_12	494226440	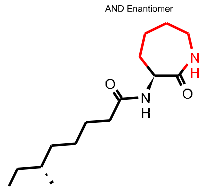 [*]1[*]NCCCC1	0.150	33 out of 35
**Top Features for negative contribution**				
**Fingerprint**	**Bit/Smiles**	**Feature Structure**	**Score**	**Irritant in training set**
FCFP_12	-1245176701	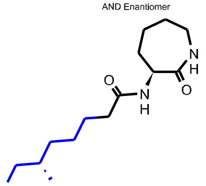 [*]CCC[C@H](C)CC	-0.592	0 out of 1
FCFP_12	618627883	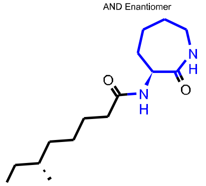 [*]N[C@H]1CCCCNC1=[*]	-0.592	0 out of 1
FCFP_12	-150334802	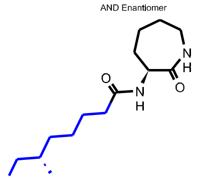 [*]CCCC[C@H](C)CC	-0.537	1 out of 3

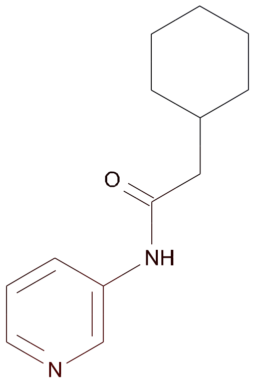

$C_{13}H_{18}N_2O$
Molecular Weight: 218.29481
ALogP: 2.171
Rotatable Bonds: 3
Acceptors: 2
Donors: 1

Structural Similar Compounds

Name	CARBAMIC ACID; METHYL-; 1-NAPHTHYL ESTER	Ethanol; 2-(2;4-dichlorophenoxy)-	ANILINE;P-PHENOXY-
Structure	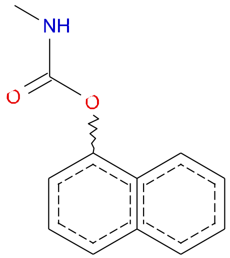	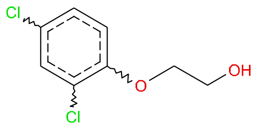	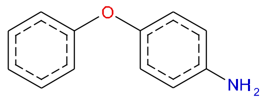
Actual Endpoint	Irritant	Irritant	Irritant
Predicted Endpoint	Irritant	Irritant	Non-Irritant
Distance	0.508	0.524	0.528
Reference	28ZPAK-;164;72	Prehled Prumyslove Toxikologie; Organicke Latky; Marhold; J. -;530;86	28ZPAK-;119;72

Model Prediction

Prediction: Irritant
Probability: 1.000
Enrichment: 1.176
Bayesian Score: 1.945
Mahalanobis Distance: 8.983
Mahalanobis Distance p-value: 0.52

Prediction: Positive if the Bayesian score is above the estimated best cutoff value from minimizing the false positive and false negative rate.
Probability: The esimated probability that the sample is in the positive category. This assumes that the Bayesian score follows a normal distribution and is different from the prediction using a cutoff.
Enrichment: An estimate of enrichment, that is, the increased likelihood (versus random) of this sample being in the category.
Bayesian Score: The standard Laplacian-modified Bayesian score.
Mahalanobis Distance: The Mahalanobis distance (MD) is the distance to the center of the training data. The larger the MD, the less trustworthy the prediction.
Mahalanobis Distance p-value: The p-value gives the fraction of training data with an MD greater than or equal to the one for the given sample, assuming normally distributed data. The smaller the p-value, the less trustworthy the prediciton. For highly non-normal X properties (e.g., fingerprints), the MD p-value is wildly inaccurate.

Model Applicability

Unknown features are fingerprint features in the query molecule, but not found in the training set.

1. All properties and OPS components are within expected ranges.

Feature Contribution

Top features for positive contribution

Fingerprint	Bit/Smiles	Feature Structure	Score	Irritant in training set
FCFP_12	1747237384	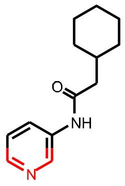 [*]:[cH]:n:[cH]:[*]	0.208	44 out of 44

FCFP_12	-124655670	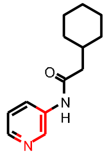<br>[*][c](:[*]):[cH]:n:[<br>*]	0.200	16 out of 16
FCFP_12	1175665944	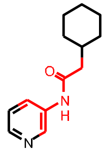<br>[*]CC(=O)N[c](:[cH]:[<br>*]):[cH]:[*]	0.198	14 out of 14
**Top Features for negative contribution**				
**Fingerprint**	**Bit/Smiles**	**Feature Structure**	**Score**	**Irritant in training set**
FCFP_12	1175638033	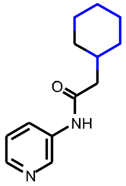<br>[*][C@@H]1[*][*]CCCC1	-0.133	207 out of 293
FCFP_12	-1525101452	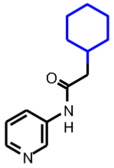<br>[*]C([*])CCCCCC(=[*])<br>[*]	-0.127	108 out of 152
FCFP_12	-55265897	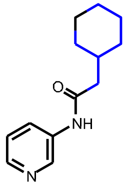<br>[*]CCCC(C)C	-0.114	77 out of 107

Indinavir

TOPKAT_Ocular_Irritancy_None_vs_Irritant

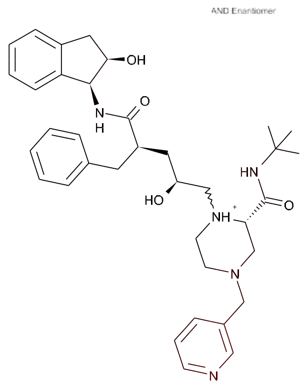

$C_{36}H_{48}N_5O_4$

Molecular Weight: 614.79741

ALogP: 1.521

Rotatable Bonds: 12

Acceptors: 6

Donors: 5

Model Prediction

Prediction: Irritant

Probability: 1.000

Enrichment: 1.176

Bayesian Score: 3.083

Mahalanobis Distance: 13.779

Mahalanobis Distance p-value: 1.14e-009

Prediction: Positive if the Bayesian score is above the estimated best cutoff value from minimizing the false positive and false negative rate.
Probability: The esimated probability that the sample is in the positive category. This assumes that the Bayesian score follows a normal distribution and is different from the prediction using a cutoff.
Enrichment: An estimate of enrichment, that is, the increased likelihood (versus random) of this sample being in the category.
Bayesian Score: The standard Laplacian-modified Bayesian score.
Mahalanobis Distance: The Mahalanobis distance (MD) is the distance to the center of the training data. The larger the MD, the less trustworthy the prediction.
Mahalanobis Distance p-value: The p-value gives the fraction of training data with an MD greater than or equal to the one for the given sample, assuming normally distributed data. The smaller the p-value, the less trustworthy the prediciton. For highly non-normal X properties (e.g., fingerprints), the MD p-value is wildly inaccurate.

Structural Similar Compounds

Name	2-Naphthalenesulfonic acid; 5;6'-iminobis(1-hydroxy-	ANTHRAQUINONE; 1;5-DIAMINO-4;8-DIHYDROXY-3-(p-METHOXYPHENYL)-	4;4'-DIAMINO-1;1'-DIANTHRIMIDE
Structure	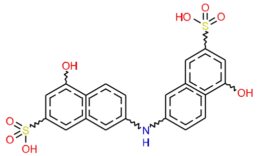	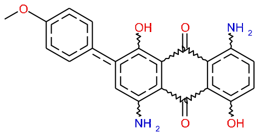	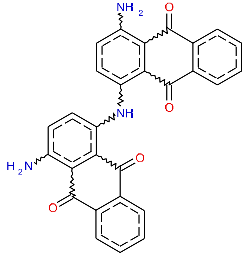
Actual Endpoint	Irritant	Irritant	Irritant
Predicted Endpoint	Irritant	Irritant	Irritant
Distance	1.069	1.082	1.125
Reference	Prehled Prumyslove Toxikologie; Organicke Latky; Marhold; J. pp 1065;86	28ZPAK 245;72	28ZPAK-;125;72

Model Applicability

Unknown features are fingerprint features in the query molecule, but not found in the training set.

1. All properties and OPS components are within expected ranges.
2. Unknown FCFP_2 feature: 10: [*][NH+]([*])[*]
3. Unknown FCFP_2 feature: -1853714334: [*]C[NH+](C[*])C([*])[*]
4. Unknown FCFP_2 feature: -1817836174: [*]C[C@H]([NH+]([*])[*])C(=[*])[*]
5. Unknown FCFP_2 feature: 1155241219: [*]CC[NH+]([*])[*]

Feature Contribution

Top features for positive contribution				
**Fingerprint**	**Bit/Smiles**	**Feature Structure**	**Score**	**Irritant in training set**

FCFP_12	1747237384	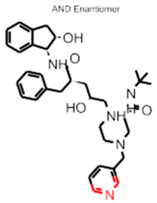<br>[*]:[cH]:n:[cH]:[*]	0.208	44 out of 44
FCFP_12	-124655670	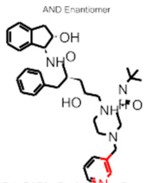<br>[*][c](:[*]):[cH]:n:[<br>*]	0.200	16 out of 16
FCFP_12	1186393305	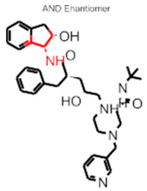<br>[*]N[C@@H]1[C@H]([*])<br>[*][*]:[c]1:[*]	0.197	13 out of 13

Top Features for negative contribution

**Fingerprint**	**Bit/Smiles**	**Feature Structure**	**Score**	**Irritant in training set**
FCFP_12	-59531427	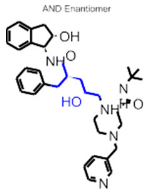<br>[*]C[C@@H](O)C[C@@H](<br>C[*])C(=[*])[*]	-0.156	4 out of 6
FCFP_12	-1947828591	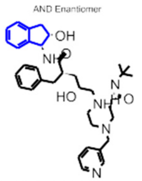<br>[*][C@@H]1C[c]2:[cH]:<br>[cH]:[cH]:[cH]:[c]:2<br>[C@@H]1[*]	-0.103	5 out of 7

FCFP_12	1981711554	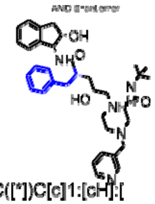 [*]C([*])C[c]1:[cH]:[cH]:[cH]:[cH]:[cH]:1	-	5 out of 7

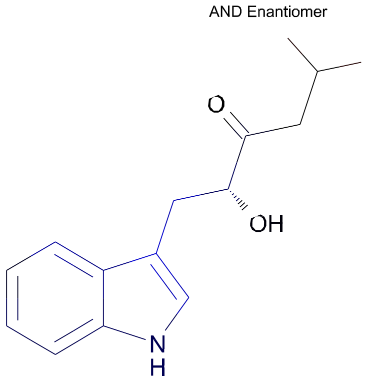

$C_{15}H_{19}NO_2$
Molecular Weight: 245.31685
ALogP: 2.932
Rotatable Bonds: 5
Acceptors: 2
Donors: 2

Model Prediction

Prediction: Non-Carcinogen
Probability: 0.254
Enrichment: 0.789
Bayesian Score: -2.508
Mahalanobis Distance: 9.727
Mahalanobis Distance p-value: 0.499

Prediction: Positive if the Bayesian score is above the estimated best cutoff value from minimizing the false positive and false negative rate.
Probability: The esimated probability that the sample is in the positive category. This assumes that the Bayesian score follows a normal distribution and is different from the prediction using a cutoff.
Enrichment: An estimate of enrichment, that is, the increased likelihood (versus random) of this sample being in the category.
Bayesian Score: The standard Laplacian-modified Bayesian score.
Mahalanobis Distance: The Mahalanobis distance (MD) is the distance to the center of the training data. The larger the MD, the less trustworthy the prediction.
Mahalanobis Distance p-value: The p-value gives the fraction of training data with an MD greater than or equal to the one for the given sample, assuming normally distributed data. The smaller the p-value, the less trustworthy the prediciton. For highly non-normal X properties (e.g., fingerprints), the MD p-value is wildly inaccurate.

Structural Similar Compounds

Name	Prilocaine	Pronetalol	Propranolol
Structure	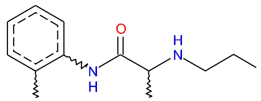	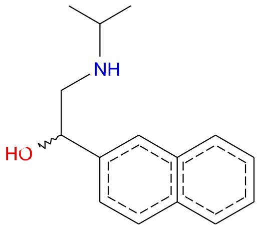	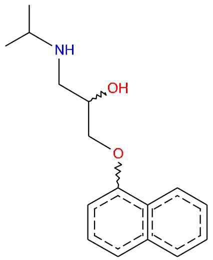
Actual Endpoint	Carcinogen	Carcinogen	Non-Carcinogen
Predicted Endpoint	Carcinogen	Carcinogen	Non-Carcinogen
Distance	0.532	0.566	0.569
Reference	US FDA (Centre for Drug Eval.& Res./Off. Testing & Res.) Sept. 1997	US FDA (Centre for Drug Eval.& Res./Off. Testing & Res.) Sept. 1997	US FDA (Centre for Drug Eval.& Res./Off. Testing & Res.) Sept. 1997

Model Applicability

Unknown features are fingerprint features in the query molecule, but not found in the training set.

1. All properties and OPS components are within expected ranges.
2. Unknown ECFP_2 feature: 80433051: [*]C[C@@H](O)C(=[*])[*]
3. Unknown ECFP_2 feature: 1732075620: [*]CC(=O)C([*])[*]

Feature Contribution

Top features for positive contribution				
**Fingerprint**	**Bit/Smiles**	**Feature Structure**	**Score**	**Carcinogen in training set**
ECFP_12	1035165602	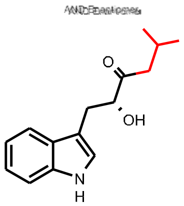 [*]CC(C)C	0.575	3 out of 4

ECFP_12	1099224616	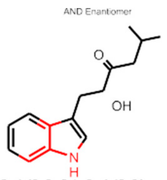<br>[*]:[cH]:[c]1:[nH]:[*<br>]:[*]:[c]:1:[*]	0.456	6 out of 11
ECFP_12	1639827160	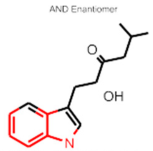<br>[*]1:[*]:[c]2:[*]:[cH<br>]:[cH]:[cH]:[c]:2:[n<br>H]:1	0.450	4 out of 7

Top Features for negative contribution

Fingerprint	Bit/Smiles	Feature Structure	Score	Carcinogen in training set
ECFP_12	-1659633832	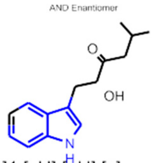<br>[*][c]1:[cH]:[nH]:[c]<br>2:[cH]:[cH]:[*]:[cH]<br>:[c]:1:2	-0.661	0 out of 3
ECFP_12	-1310859884	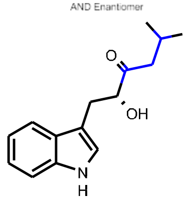<br>[*]C([*])CC(=[*])[*]	-0.661	0 out of 3
ECFP_12	-93267474	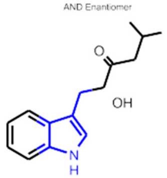<br>[*]C[c]1:[cH]:[nH]:[c<br>](:[*]):[c]:1:[*]	-0.485	0 out of 2

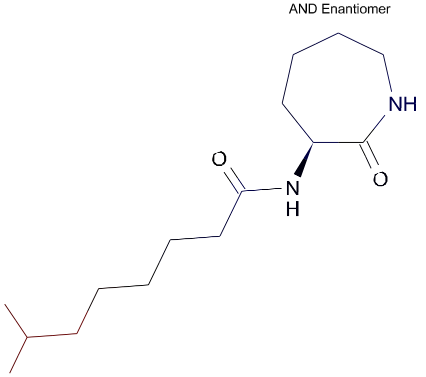

$C_{15}H_{28}N_2O_2$
Molecular Weight: 268.39501
ALogP: 2.685
Rotatable Bonds: 7
Acceptors: 2
Donors: 2

Model Prediction

Prediction: Non-Carcinogen
Probability: 0.261
Enrichment: 0.811
Bayesian Score: -2.061
Mahalanobis Distance: 12.434
Mahalanobis Distance p-value: 0.00116

Prediction: Positive if the Bayesian score is above the estimated best cutoff value from minimizing the false positive and false negative rate.
Probability: The esimated probability that the sample is in the positive category. This assumes that the Bayesian score follows a normal distribution and is different from the prediction using a cutoff.
Enrichment: An estimate of enrichment, that is, the increased likelihood (versus random) of this sample being in the category.
Bayesian Score: The standard Laplacian-modified Bayesian score.
Mahalanobis Distance: The Mahalanobis distance (MD) is the distance to the center of the training data. The larger the MD, the less trustworthy the prediction.
Mahalanobis Distance p-value: The p-value gives the fraction of training data with an MD greater than or equal to the one for the given sample, assuming normally distributed data. The smaller the p-value, the less trustworthy the prediciton. For highly non-normal X properties (e.g., fingerprints), the MD p-value is wildly inaccurate.

Structural Similar Compounds

Name	Propranolol	Penbutalol	Prilocaine
Structure	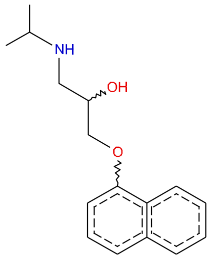	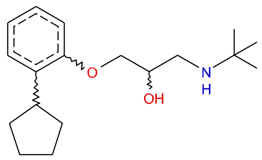	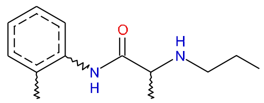
Actual Endpoint	Non-Carcinogen	Non-Carcinogen	Carcinogen
Predicted Endpoint	Non-Carcinogen	Non-Carcinogen	Carcinogen
Distance	0.598	0.614	0.619
Reference	US FDA (Centre for Drug Eval.& Res./Off. Testing & Res.) Sept. 1997	US FDA (Centre for Drug Eval.& Res./Off. Testing & Res.) Sept. 1997	US FDA (Centre for Drug Eval.& Res./Off. Testing & Res.) Sept. 1997

Model Applicability

Unknown features are fingerprint features in the query molecule, but not found in the training set.

1. All properties and OPS components are within expected ranges.

Feature Contribution

Top features for positive contribution

Fingerprint	Bit/Smiles	Feature Structure	Score	Carcinogen in training set
ECFP_12	1035165602	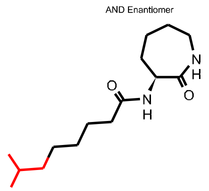 [*]CC(C)C	0.575	3 out of 4

ECFP_12	-2119919996	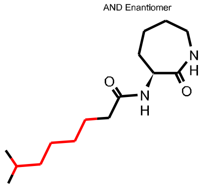 [*]CCCCC([*])[*]	0.421	1 out of 1
ECFP_12	-1291950249	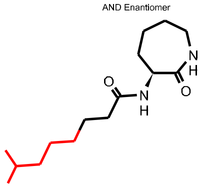 [*]CCCC(C)C	0.421	1 out of 1
**Top Features for negative contribution**				
**Fingerprint**	**Bit/Smiles**	**Feature Structure**	**Score**	**Carcinogen in training set**
ECFP_12	660766836	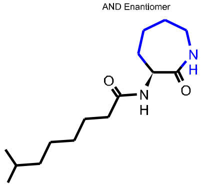 [*]1[*]NCCCC1	-0.661	0 out of 3
ECFP_12	-867777309	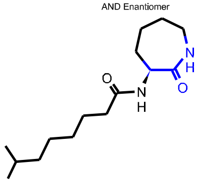 [*]NC(=O)C([*])[*]	-0.661	0 out of 3
ECFP_12	-2123658813	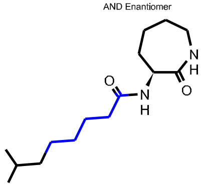 [*]CCCCC(=[*])[*]	-0.661	0 out of 3

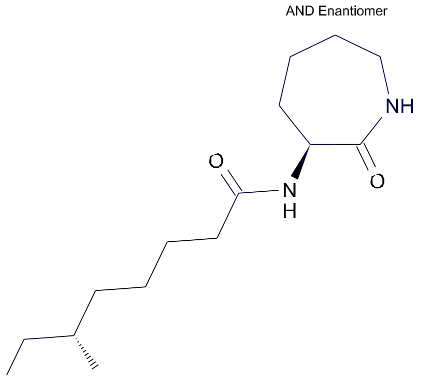

$C_{15}H_{28}N_2O_2$
Molecular Weight: 268.39501
ALogP: 2.685
Rotatable Bonds: 7
Acceptors: 2
Donors: 2

Structural Similar Compounds

Name	Propranolol	Penbutalol	Prilocaine
Structure	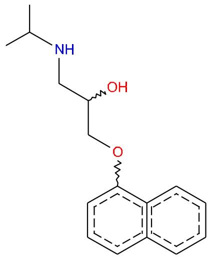	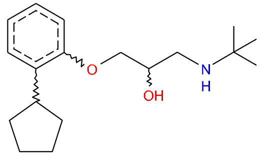	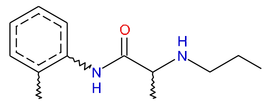
Actual Endpoint	Non-Carcinogen	Non-Carcinogen	Carcinogen
Predicted Endpoint	Non-Carcinogen	Non-Carcinogen	Carcinogen
Distance	0.598	0.615	0.615
Reference	US FDA (Centre for Drug Eval.& Res./Off. Testing & Res.) Sept. 1997	US FDA (Centre for Drug Eval.& Res./Off. Testing & Res.) Sept. 1997	US FDA (Centre for Drug Eval.& Res./Off. Testing & Res.) Sept. 1997

Model Prediction

Prediction: Non-Carcinogen
Probability: 0.233
Enrichment: 0.722
Bayesian Score: -4.042
Mahalanobis Distance: 12.614
Mahalanobis Distance p-value: 0.000618

Prediction: Positive if the Bayesian score is above the estimated best cutoff value from minimizing the false positive and false negative rate.
Probability: The esimated probability that the sample is in the positive category. This assumes that the Bayesian score follows a normal distribution and is different from the prediction using a cutoff.
Enrichment: An estimate of enrichment, that is, the increased likelihood (versus random) of this sample being in the category.
Bayesian Score: The standard Laplacian-modified Bayesian score.
Mahalanobis Distance: The Mahalanobis distance (MD) is the distance to the center of the training data. The larger the MD, the less trustworthy the prediction.
Mahalanobis Distance p-value: The p-value gives the fraction of training data with an MD greater than or equal to the one for the given sample, assuming normally distributed data. The smaller the p-value, the less trustworthy the prediciton. For highly non-normal X properties (e.g., fingerprints), the MD p-value is wildly inaccurate.

Model Applicability

Unknown features are fingerprint features in the query molecule, but not found in the training set.

1. All properties and OPS components are within expected ranges.
2. Unknown ECFP_2 feature: 194354829: [*]CC(C)C[*]

Feature Contribution

Top features for positive contribution

Fingerprint	Bit/Smiles	Feature Structure	Score	Carcinogen in training set
ECFP_12	-2119919996	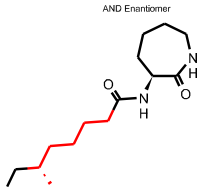 [*]CCCCC([*])[*]	0.421	1 out of 1

ECFP_12	-2097159651	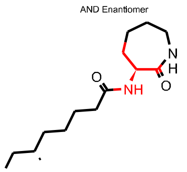 [*]C[C@H](N[*])C(=[*])[*]	0.208	1 out of 2
ECFP_12	-1694930393	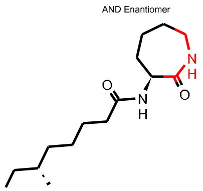 [*]CNC(=[*])[*]	0.158	2 out of 5
**Top Features for negative contribution**				
**Fingerprint**	**Bit/Smiles**	**Feature Structure**	**Score**	**Carcinogen in training set**
ECFP_12	-949992060	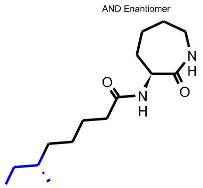 [*]C([*])CC	-0.811	0 out of 4
ECFP_12	660766836	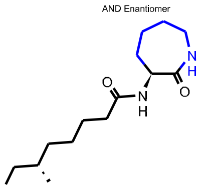 [*]1[*]NCCCC1	-0.661	0 out of 3
ECFP_12	-867777309	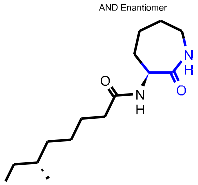 [*]NC(=O)C([*])[*]	-0.661	0 out of 3

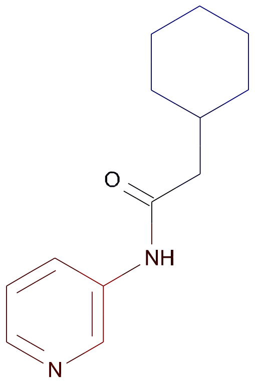

$C_{13}H_{18}N_2O$

Molecular Weight: 218.29481

ALogP: 2.171

Rotatable Bonds: 3

Acceptors: 2

Donors: 1

Model Prediction

Prediction: Carcinogen

Probability: 0.300

Enrichment: 0.932

Bayesian Score: 0.077

Mahalanobis Distance: 10.375

Mahalanobis Distance p-value: 0.217

Prediction: Positive if the Bayesian score is above the estimated best cutoff value from minimizing the false positive and false negative rate.
Probability: The esimated probability that the sample is in the positive category. This assumes that the Bayesian score follows a normal distribution and is different from the prediction using a cutoff.
Enrichment: An estimate of enrichment, that is, the increased likelihood (versus random) of this sample being in the category.
Bayesian Score: The standard Laplacian-modified Bayesian score.
Mahalanobis Distance: The Mahalanobis distance (MD) is the distance to the center of the training data. The larger the MD, the less trustworthy the prediction.
Mahalanobis Distance p-value: The p-value gives the fraction of training data with an MD greater than or equal to the one for the given sample, assuming normally distributed data. The smaller the p-value, the less trustworthy the prediciton. For highly non-normal X properties (e.g., fingerprints), the MD p-value is wildly inaccurate.

Structural Similar Compounds

Name	Mexiletine	Phenacetin	Methylphenidate
Structure	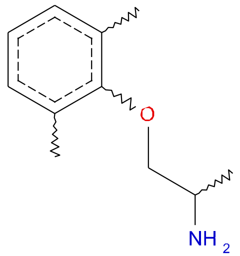	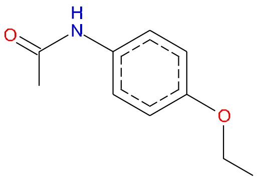	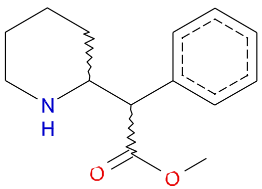
Actual Endpoint	Non-Carcinogen	Carcinogen	Non-Carcinogen
Predicted Endpoint	Non-Carcinogen	Carcinogen	Non-Carcinogen
Distance	0.543	0.547	0.549
Reference	US FDA (Centre for Drug Eval.& Res./Off. Testing & Res.) Sept. 1997	US FDA (Centre for Drug Eval.& Res./Off. Testing & Res.) Sept. 1997	US FDA (Centre for Drug Eval.& Res./Off. Testing & Res.) Sept. 1997

Model Applicability

Unknown features are fingerprint features in the query molecule, but not found in the training set.

1. All properties and OPS components are within expected ranges.
2. Unknown ECFP_2 feature: -82840383: [*]C([*])CC(=[*])[*]

Feature Contribution

Top features for positive contribution

Fingerprint	Bit/Smiles	Feature Structure	Score	Carcinogen in training set
ECFP_12	-177077903	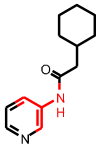 [*]N[c](:[cH]:[*]):[cH]:[*]	0.529	6 out of 10

ECFP_12	-1236483485	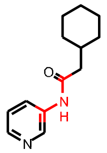 [*]C(=[*])N[c](:[*]):[*]	0.460	9 out of 17
ECFP_12	894876384	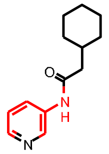 [*]N[c]1:[cH]:[*]:[cH]:[cH]:[cH]:1	0.421	1 out of 1
**Top Features for negative contribution**				
**Fingerprint**	**Bit/Smiles**	**Feature Structure**	**Score**	**Carcinogen in training set**
ECFP_12	662850656	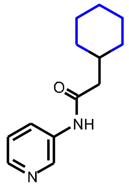 [*]1CCCCC1	-0.929	1 out of 13
ECFP_12	663943468	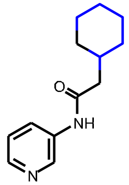 [*][C@@H]1[*][*]CCCC1	-0.523	2 out of 13
ECFP_12	-1607899848	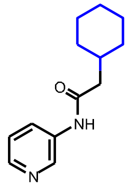 [*]C1CCCCC1	-0.466	1 out of 7

Indinavir

TOPKAT_Rat_Female_FDA_None_vs_Carcinogen

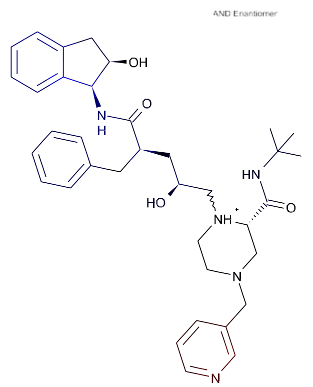

$C_{36}H_{48}N_5O_4$
Molecular Weight: 614.79741
ALogP: 1.521
Rotatable Bonds: 12
Acceptors: 6
Donors: 5

Structural Similar Compounds

Name	Pravastatin	Mesuprine	Glyburide
Structure	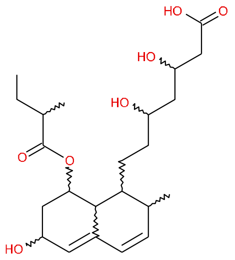	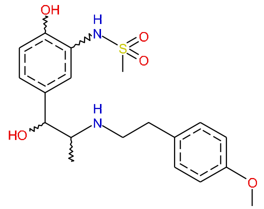	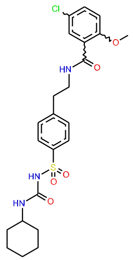
Actual Endpoint	Non-Carcinogen	Carcinogen	Non-Carcinogen
Predicted Endpoint	Non-Carcinogen	Carcinogen	Non-Carcinogen
Distance	0.872	0.947	0.988
Reference	US FDA (Centre for Drug Eval.& Res./Off. Testing & Res.) Sept. 1997	US FDA (Centre for Drug Eval.& Res./Off. Testing & Res.) Sept. 1997	US FDA (Centre for Drug Eval.& Res./Off. Testing & Res.) Sept. 1997

Model Prediction

Prediction: Non-Carcinogen
Probability: 0.188
Enrichment: 0.583
Bayesian Score: -8.907
Mahalanobis Distance: 17.376
Mahalanobis Distance p-value: 3.93e-014

Prediction: Positive if the Bayesian score is above the estimated best cutoff value from minimizing the false positive and false negative rate.
Probability: The esimated probability that the sample is in the positive category. This assumes that the Bayesian score follows a normal distribution and is different from the prediction using a cutoff.
Enrichment: An estimate of enrichment, that is, the increased likelihood (versus random) of this sample being in the category.
Bayesian Score: The standard Laplacian-modified Bayesian score.
Mahalanobis Distance: The Mahalanobis distance (MD) is the distance to the center of the training data. The larger the MD, the less trustworthy the prediction.
Mahalanobis Distance p-value: The p-value gives the fraction of training data with an MD greater than or equal to the one for the given sample, assuming normally distributed data. The smaller the p-value, the less trustworthy the prediciton. For highly non-normal X properties (e.g., fingerprints), the MD p-value is wildly inaccurate.

Model Applicability

Unknown features are fingerprint features in the query molecule, but not found in the training set.

1. All properties and OPS components are within expected ranges.
2. Unknown ECFP_2 feature: 1976330679: [*][NH+]([*])[*]
3. Unknown ECFP_2 feature: 1134829831: [*]C[NH+](C[*])C([*])[*]
4. Unknown ECFP_2 feature: -1924540582: [*]C[C@H]([NH+]([*])[*])C(=[*])[*]
5. Unknown ECFP_2 feature: -244159614: [*]CC[NH+]([*])[*]
6. Unknown ECFP_2 feature: 474121058: [*]C([*])C[NH+]([*])[*]

Feature Contribution

Top features for positive contribution				
**Fingerprint**	**Bit/Smiles**	**Feature Structure**	**Score**	**Carcinogen in training set**
ECFP_12	53207596	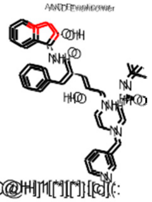 [*][C@H]([*])[c](:[*])C1	0.459	8 out of 15

ECFP_12	-1818873508	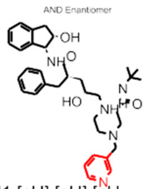<br>[*][c]1:[cH]:[cH]:[cH<br>]:n:[cH]:1	0.421	1 out of 1
ECFP_12	198723869	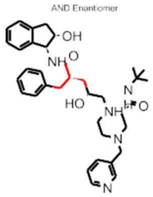<br>[*]CC(C[*])C(=[*])[*]	0.421	1 out of 1

Top Features for negative contribution

Fingerprint	Bit/Smiles	Feature Structure	Score	Carcinogen in training set
ECFP_12	-1567199489	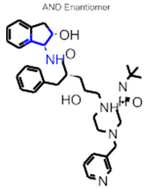<br>[*]N[C@@H]1[C@H]([*])<br>[*][*]:[c]1:[*]	-0.811	0 out of 4
ECFP_12	1601903945	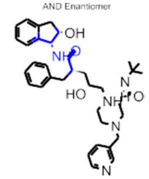<br>[*]C([*])C(=O)N[C@@H]<br>1[C@H]([*])[*][*]:[c<br>]1:[*]	-0.661	0 out of 3
ECFP_12	1638636092	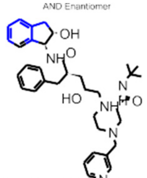<br>[*]1[*][c]2:[*]:[cH]:<br>[cH]:[cH]:[c]:2C1	-0.645	1 out of 9

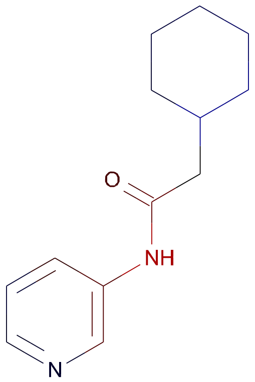

$C_{13}H_{18}N_2O$
Molecular Weight: 218.29481
ALogP: 2.171
Rotatable Bonds: 3
Acceptors: 2
Donors: 1

Model Prediction

Prediction: Multiple-Carcinogen
Probability: 0.532
Enrichment: 1.423
Bayesian Score: 1.490
Mahalanobis Distance: 12.005
Mahalanobis Distance p-value: 0.00174

Prediction: Positive if the Bayesian score is above the estimated best cutoff value from minimizing the false positive and false negative rate.
Probability: The esimated probability that the sample is in the positive category. This assumes that the Bayesian score follows a normal distribution and is different from the prediction using a cutoff.
Enrichment: An estimate of enrichment, that is, the increased likelihood (versus random) of this sample being in the category.
Bayesian Score: The standard Laplacian-modified Bayesian score.
Mahalanobis Distance: The Mahalanobis distance (MD) is the distance to the center of the training data. The larger the MD, the less trustworthy the prediction.
Mahalanobis Distance p-value: The p-value gives the fraction of training data with an MD greater than or equal to the one for the given sample, assuming normally distributed data. The smaller the p-value, the less trustworthy the prediciton. For highly non-normal X properties (e.g., fingerprints), the MD p-value is wildly inaccurate.

Structural Similar Compounds

Name	Phenacetin	Lidocaine	Prilocaine
Structure	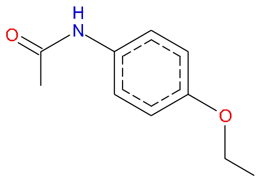	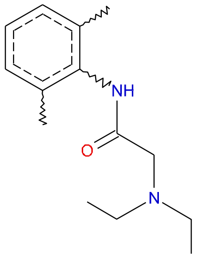	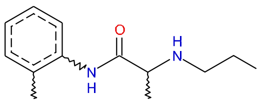
Actual Endpoint	Multiple-Carcinogen	Multiple-Carcinogen	Multiple-Carcinogen
Predicted Endpoint	Multiple-Carcinogen	Multiple-Carcinogen	Multiple-Carcinogen
Distance	0.456	0.525	0.569
Reference	US FDA (Centre for Drug Eval.& Res./Off. Testing & Res.) Sept. 1997	US FDA (Centre for Drug Eval.& Res./Off. Testing & Res.) Sept. 1997	US FDA (Centre for Drug Eval.& Res./Off. Testing & Res.) Sept. 1997

Model Applicability

Unknown features are fingerprint features in the query molecule, but not found in the training set.

1. OPS PC8 out of range. Value: 4.2747. Training min, max, SD, explained variance: -3.3768, 4.2738, 1.554, 0.0358.

Feature Contribution

Top features for positive contribution

Fingerprint	Bit/Smiles	Feature Structure	Score	Multiple-Carcinogen in training set
SCFP_4	2097618059	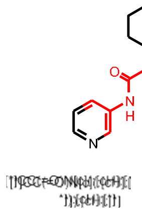	0.730	5 out of 6

SCFP_4	1631845520	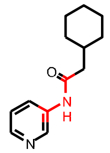 [*]C(=[*])N[c](:[*]):[*]	0.601	6 out of 9
SCFP_4	-1375926917	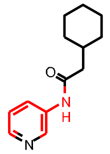 [*]N[c]1:[cH]:[*]:[cH]:[cH]:[cH]:1	0.522	6 out of 10

Top Features for negative contribution

**Fingerprint**	**Bit/Smiles**	**Feature Structure**	**Score**	**Multiple-Carcinogen in training set**
SCFP_4	1175638033	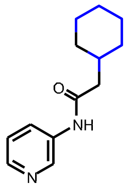 [*]C1[*]CCCC1	-1.165	0 out of 7
SCFP_4	-1043339860	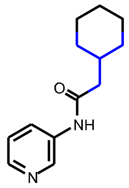 [*]CC(C[*])C[*]	-0.675	4 out of 28
SCFP_4	1188429584	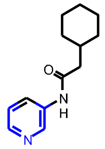 [*][c]1:[*]:[cH]:[cH]:n:[cH]:1	-0.666	0 out of 3

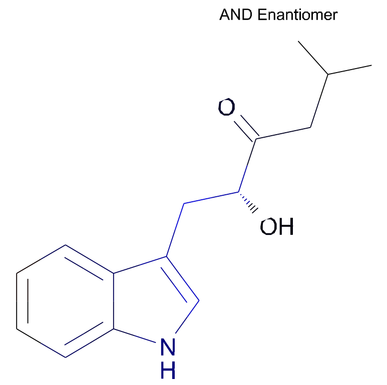

$C_{15}H_{19}NO_2$
Molecular Weight: 245.31685
ALogP: 2.932
Rotatable Bonds: 5
Acceptors: 2
Donors: 2

Model Prediction

Prediction: Non-Carcinogen
Probability: 0.313
Enrichment: 0.937
Bayesian Score: -1.592
Mahalanobis Distance: 11.803
Mahalanobis Distance p-value: 0.0454

Prediction: Positive if the Bayesian score is above the estimated best cutoff value from minimizing the false positive and false negative rate.
Probability: The esimated probability that the sample is in the positive category. This assumes that the Bayesian score follows a normal distribution and is different from the prediction using a cutoff.
Enrichment: An estimate of enrichment, that is, the increased likelihood (versus random) of this sample being in the category.
Bayesian Score: The standard Laplacian-modified Bayesian score.
Mahalanobis Distance: The Mahalanobis distance (MD) is the distance to the center of the training data. The larger the MD, the less trustworthy the prediction.
Mahalanobis Distance p-value: The p-value gives the fraction of training data with an MD greater than or equal to the one for the given sample, assuming normally distributed data. The smaller the p-value, the less trustworthy the prediciton. For highly non-normal X properties (e.g., fingerprints), the MD p-value is wildly inaccurate.

Structural Similar Compounds

Name	Etodolac	Prilocaine	Pronetalol
Structure	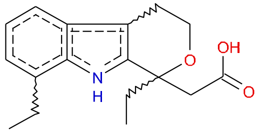	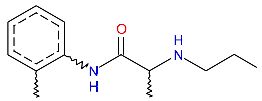	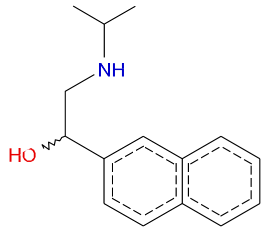
Actual Endpoint	Non-Carcinogen	Carcinogen	Carcinogen
Predicted Endpoint	Non-Carcinogen	Carcinogen	Non-Carcinogen
Distance	0.531	0.534	0.541
Reference	US FDA (Centre for Drug Eval.& Res./Off. Testing & Res.) Sept. 1997	US FDA (Centre for Drug Eval.& Res./Off. Testing & Res.) Sept. 1997	US FDA (Centre for Drug Eval.& Res./Off. Testing & Res.) Sept. 1997

Model Applicability

Unknown features are fingerprint features in the query molecule, but not found in the training set.

1. All properties and OPS components are within expected ranges.

Feature Contribution

Top features for positive contribution

Fingerprint	Bit/Smiles	Feature Structure	Score	Carcinogen in training set
SCFP_6	1651620003	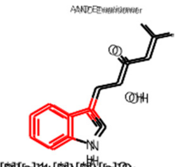 [*][c]1:[*]:[*][c]2:[cH]:[cH]:[cH]:[cH]:[c]:1:2	0.643	7 out of 10

SCFP_6	-1379673609	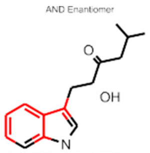 [*][c]1:[*]:[*]:[c]2:[*]:[cH]:[cH]:[cH]:[c]:1:2	0.526	11 out of 19
SCFP_6	1655199790	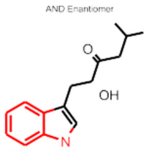 [*]1:[*]:[c]2:[cH]:[cH]:[cH]:[cH]:[c]:2:[nH]:1	0.520	5 out of 8

Top Features for negative contribution

**Fingerprint**	**Bit/Smiles**	**Feature Structure**	**Score**	**Carcinogen in training set**
SCFP_6	571765461	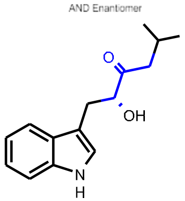 [*]CC(=O)C([*])[*]	-0.715	2 out of 16
SCFP_6	-1770674960	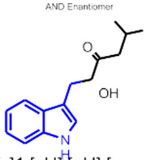 [*]C[c]1:[cH]:[nH]:[c]2:[cH]:[cH]:[cH]:[cH]:[c]:1:2	-0.674	0 out of 3
SCFP_6	2054003838	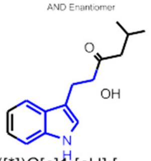 [*]C([*])C[c]1:[cH]:[nH]:[c]2:[cH]:[*]:[*]:[cH]:[c]:1:2	-0.674	0 out of 3

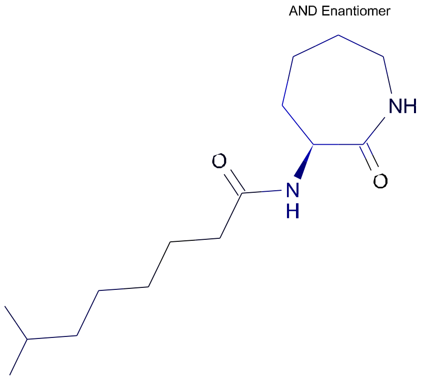

$C_{15}H_{28}N_2O_2$
Molecular Weight: 268.39501
ALogP: 2.685
Rotatable Bonds: 7
Acceptors: 2
Donors: 2

Model Prediction

Prediction: Non-Carcinogen
Probability: 0.225
Enrichment: 0.672
Bayesian Score: -5.785
Mahalanobis Distance: 11.192
Mahalanobis Distance p-value: 0.156

Prediction: Positive if the Bayesian score is above the estimated best cutoff value from minimizing the false positive and false negative rate.
Probability: The esimated probability that the sample is in the positive category. This assumes that the Bayesian score follows a normal distribution and is different from the prediction using a cutoff.
Enrichment: An estimate of enrichment, that is, the increased likelihood (versus random) of this sample being in the category.
Bayesian Score: The standard Laplacian-modified Bayesian score.
Mahalanobis Distance: The Mahalanobis distance (MD) is the distance to the center of the training data. The larger the MD, the less trustworthy the prediction.
Mahalanobis Distance p-value: The p-value gives the fraction of training data with an MD greater than or equal to the one for the given sample, assuming normally distributed data. The smaller the p-value, the less trustworthy the prediciton. For highly non-normal X properties (e.g., fingerprints), the MD p-value is wildly inaccurate.

Structural Similar Compounds

Name	Prilocaine	Propranolol	Penbutalol
Structure	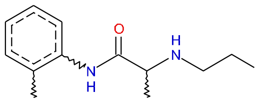	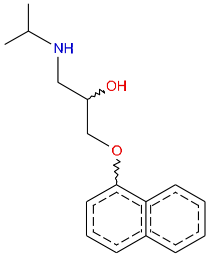	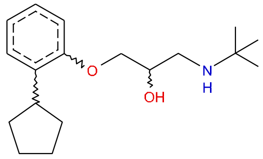
Actual Endpoint	Carcinogen	Non-Carcinogen	Non-Carcinogen
Predicted Endpoint	Carcinogen	Non-Carcinogen	Non-Carcinogen
Distance	0.602	0.607	0.626
Reference	US FDA (Centre for Drug Eval.& Res./Off. Testing & Res.) Sept. 1997	US FDA (Centre for Drug Eval.& Res./Off. Testing & Res.) Sept. 1997	US FDA (Centre for Drug Eval.& Res./Off. Testing & Res.) Sept. 1997

Model Applicability

Unknown features are fingerprint features in the query molecule, but not found in the training set.

1. All properties and OPS components are within expected ranges.

Feature Contribution

Top features for positive contribution

Fingerprint	Bit/Smiles	Feature Structure	Score	Carcinogen in training set
SCFP_6	-1272768868	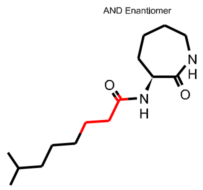 [*]C([*])CC(=[*])[*]	0.242	26 out of 63

Fingerprint	Bit/Smiles	Feature Structure	Score	Carcinogen in training set
SCFP_6	-1072897324	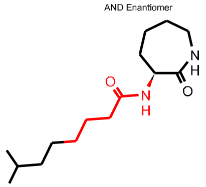 [*]CCCC(=O)N[*]	0.198	1 out of 2
SCFP_6	13	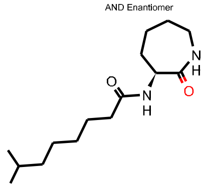 [*]=O	0.072	90 out of 261
**Top Features for negative contribution**				
**Fingerprint**	**Bit/Smiles**	**Feature Structure**	**Score**	**Carcinogen in training set**
SCFP_6	-1946889102	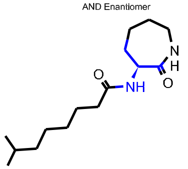 [*]C[C@H](N[*])C(=[*]<br>)[*]	-0.885	1 out of 12
SCFP_6	2002926168	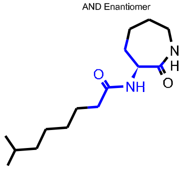 [*]C[C@H](NC(=O)C[*])<br>C(=[*])[*]	-0.825	0 out of 4
SCFP_6	1175638033	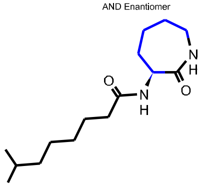 [*][C@@H]1[*][*]CCCC1	-0.812	4 out of 32

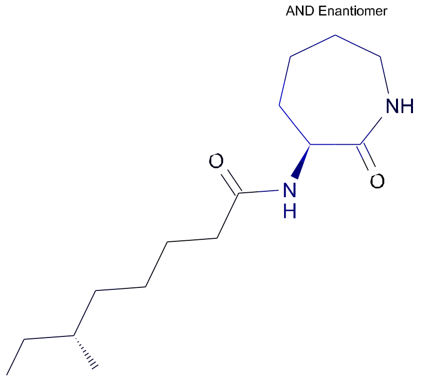

$C_{15}H_{28}N_2O_2$
Molecular Weight: 268.39501
ALogP: 2.685
Rotatable Bonds: 7
Acceptors: 2
Donors: 2

Structural Similar Compounds

Name	Prilocaine	Propranolol	Penbutalol
Structure	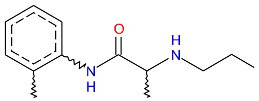	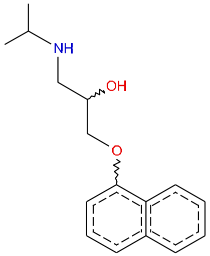	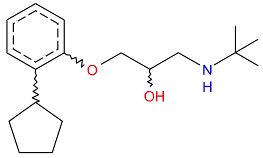
Actual Endpoint	Carcinogen	Non-Carcinogen	Non-Carcinogen
Predicted Endpoint	Carcinogen	Non-Carcinogen	Non-Carcinogen
Distance	0.602	0.607	0.626
Reference	US FDA (Centre for Drug Eval.& Res./Off. Testing & Res.) Sept. 1997	US FDA (Centre for Drug Eval.& Res./Off. Testing & Res.) Sept. 1997	US FDA (Centre for Drug Eval.& Res./Off. Testing & Res.) Sept. 1997

Model Prediction

Prediction: Non-Carcinogen
Probability: 0.235
Enrichment: 0.703
Bayesian Score: -5.196
Mahalanobis Distance: 11.192
Mahalanobis Distance p-value: 0.156

Prediction: Positive if the Bayesian score is above the estimated best cutoff value from minimizing the false positive and false negative rate.
Probability: The esimated probability that the sample is in the positive category. This assumes that the Bayesian score follows a normal distribution and is different from the prediction using a cutoff.
Enrichment: An estimate of enrichment, that is, the increased likelihood (versus random) of this sample being in the category.
Bayesian Score: The standard Laplacian-modified Bayesian score.
Mahalanobis Distance: The Mahalanobis distance (MD) is the distance to the center of the training data. The larger the MD, the less trustworthy the prediction.
Mahalanobis Distance p-value: The p-value gives the fraction of training data with an MD greater than or equal to the one for the given sample, assuming normally distributed data. The smaller the p-value, the less trustworthy the prediciton. For highly non-normal X properties (e.g., fingerprints), the MD p-value is wildly inaccurate.

Model Applicability

Unknown features are fingerprint features in the query molecule, but not found in the training set.

1. All properties and OPS components are within expected ranges.

Feature Contribution

Top features for positive contribution

Fingerprint	Bit/Smiles	Feature Structure	Score	Carcinogen in training set
SCFP_6	-1272768868	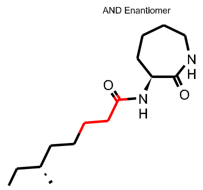 [*]C([*])CC(=[*])[*]	0.242	26 out of 63

SCFP_6	-1072897324	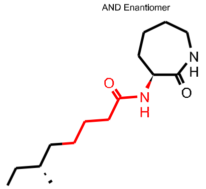 [*]CCCC(=O)N[*]	0.198	1 out of 2
SCFP_6	13	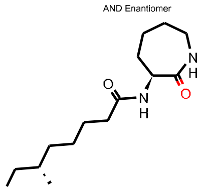 [*]=O	0.072	90 out of 261
**Top Features for negative contribution**				
**Fingerprint**	**Bit/Smiles**	**Feature Structure**	**Score**	**Carcinogen in training set**
SCFP_6	-1946889102	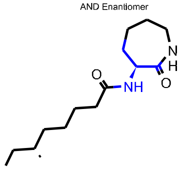 [*]C[C@H](N[*])C(=[*])[*]	-0.885	1 out of 12
SCFP_6	2002926168	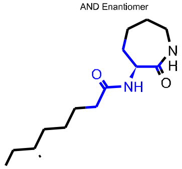 [*]C[C@H](NC(=O)C[*]) C(=[*])[*]	-0.825	0 out of 4
SCFP_6	1175638033	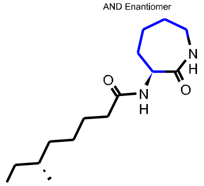 [*][C@@H]1[*][*]CCCC1	-0.812	4 out of 32

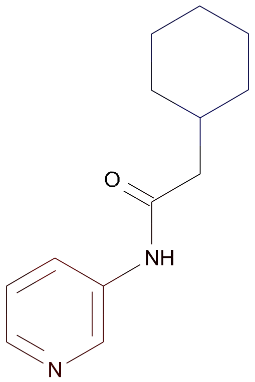

$C_{13}H_{18}N_2O$
Molecular Weight: 218.29481
ALogP: 2.171
Rotatable Bonds: 3
Acceptors: 2
Donors: 1

Model Prediction

Prediction: Carcinogen
Probability: 0.393
Enrichment: 1.175
Bayesian Score: 1.206
Mahalanobis Distance: 11.681
Mahalanobis Distance p-value: 0.0597

Prediction: Positive if the Bayesian score is above the estimated best cutoff value from minimizing the false positive and false negative rate.
Probability: The esimated probability that the sample is in the positive category. This assumes that the Bayesian score follows a normal distribution and is different from the prediction using a cutoff.
Enrichment: An estimate of enrichment, that is, the increased likelihood (versus random) of this sample being in the category.
Bayesian Score: The standard Laplacian-modified Bayesian score.
Mahalanobis Distance: The Mahalanobis distance (MD) is the distance to the center of the training data. The larger the MD, the less trustworthy the prediction.
Mahalanobis Distance p-value: The p-value gives the fraction of training data with an MD greater than or equal to the one for the given sample, assuming normally distributed data. The smaller the p-value, the less trustworthy the prediciton. For highly non-normal X properties (e.g., fingerprints), the MD p-value is wildly inaccurate.

Structural Similar Compounds

Name	Phenacetin	Mexiletine	Methylphenidate
Structure	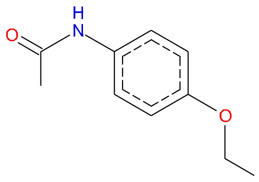	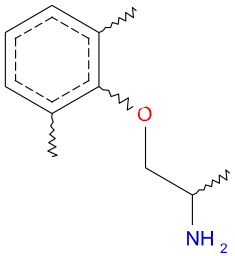	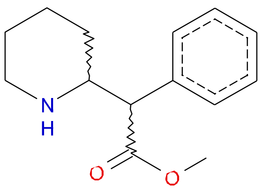
Actual Endpoint	Carcinogen	Non-Carcinogen	Non-Carcinogen
Predicted Endpoint	Carcinogen	Non-Carcinogen	Non-Carcinogen
Distance	0.504	0.551	0.557
Reference	US FDA (Centre for Drug Eval.& Res./Off. Testing & Res.) Sept. 1997	US FDA (Centre for Drug Eval.& Res./Off. Testing & Res.) Sept. 1997	US FDA (Centre for Drug Eval.& Res./Off. Testing & Res.) Sept. 1997

Model Applicability

Unknown features are fingerprint features in the query molecule, but not found in the training set.

1. All properties and OPS components are within expected ranges.

Feature Contribution

Top features for positive contribution

Fingerprint	Bit/Smiles	Feature Structure	Score	Carcinogen in training set
SCFP_6	2097618059	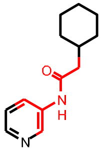	0.437	7 out of 13

SCFP_6	-105808146	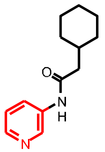 [*][c]1:[cH]:[cH]:[cH]:n:[cH]:1	0.415	1 out of 1
SCFP_6	-758850909	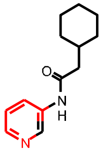 [*][c]1:[*]:n:[cH]:[cH]:[cH]:1	0.355	5 out of 10
**Top Features for negative contribution**				
**Fingerprint**	**Bit/Smiles**	**Feature Structure**	**Score**	**Carcinogen in training set**
SCFP_6	1175638033	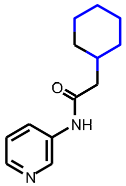 [*][C@@H]1[*][*]CCCC1	-0.812	4 out of 32
SCFP_6	-216207339	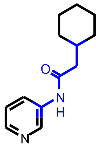 [*]C([*])CC(=O)N[c](:[*]):[*]	-0.496	0 out of 2
SCFP_6	1188429584	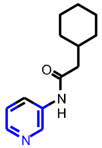 [*][c]1:[*]:[cH]:[cH]:n:[cH]:1	-0.264	1 out of 5

Indinavir

TOPKAT_Rat_Male_FDA_None_vs_Carcinogen

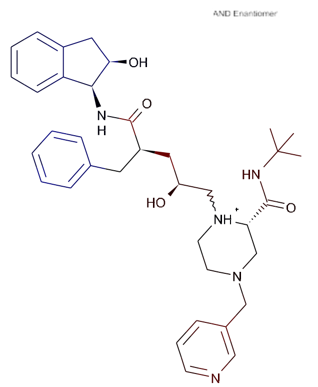

$C_{36}H_{48}N_5O_4$
Molecular Weight: 614.79741
ALogP: 1.521
Rotatable Bonds: 12
Acceptors: 6
Donors: 5

Model Prediction

Prediction: Non-Carcinogen
Probability: 0.293
Enrichment: 0.878
Bayesian Score: -2.396
Mahalanobis Distance: 15.838
Mahalanobis Distance p-value: 1.17e-008

Prediction: Positive if the Bayesian score is above the estimated best cutoff value from minimizing the false positive and false negative rate.
Probability: The esimated probability that the sample is in the positive category. This assumes that the Bayesian score follows a normal distribution and is different from the prediction using a cutoff.
Enrichment: An estimate of enrichment, that is, the increased likelihood (versus random) of this sample being in the category.
Bayesian Score: The standard Laplacian-modified Bayesian score.
Mahalanobis Distance: The Mahalanobis distance (MD) is the distance to the center of the training data. The larger the MD, the less trustworthy the prediction.
Mahalanobis Distance p-value: The p-value gives the fraction of training data with an MD greater than or equal to the one for the given sample, assuming normally distributed data. The smaller the p-value, the less trustworthy the prediciton. For highly non-normal X properties (e.g., fingerprints), the MD p-value is wildly inaccurate.

Structural Similar Compounds

Name	Pravastatin	Mesuprine	Quinapril
Structure	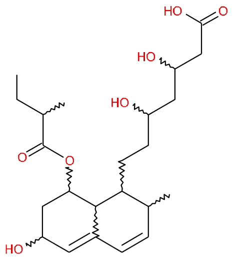	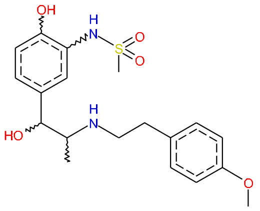	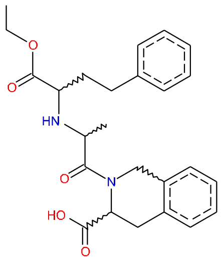
Actual Endpoint	Carcinogen	Non-Carcinogen	Non-Carcinogen
Predicted Endpoint	Carcinogen	Non-Carcinogen	Non-Carcinogen
Distance	0.867	0.920	0.959
Reference	US FDA (Centre for Drug Eval.& Res./Off. Testing & Res.) Sept. 1997	US FDA (Centre for Drug Eval.& Res./Off. Testing & Res.) Sept. 1997	US FDA (Centre for Drug Eval.& Res./Off. Testing & Res.) Sept. 1997

Model Applicability

Unknown features are fingerprint features in the query molecule, but not found in the training set.

1. All properties and OPS components are within expected ranges.

Feature Contribution

Top features for positive contribution

Fingerprint	Bit/Smiles	Feature Structure	Score	Carcinogen in training set
SCFP_6	-1903488337	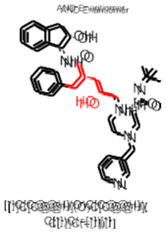	0.603	2 out of 2

SCFP_6	1453622480	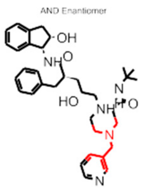 [*]CN(C[*])C[c](:[cH]:[*]):[cH]:[*]	0.561	3 out of 4
SCFP_6	2088794301	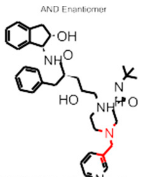 [*]N([*])C[c](:[*]):[*]	0.432	4 out of 7
**Top Features for negative contribution**				
**Fingerprint**	**Bit/Smiles**	**Feature Structure**	**Score**	**Carcinogen in training set**
SCFP_6	-1211866396	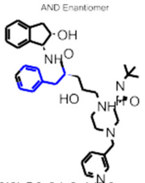 [*]C([*])C[c]1:[cH]:[cH]:[*]:[cH]:[cH]:1	-1.101	2 out of 25
SCFP_6	-1640858361	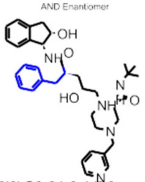 [*]C([*])C[c]1:[cH]:[cH]:[cH]:[cH]:[cH]:1	-0.817	1 out of 11
SCFP_6	1653911926	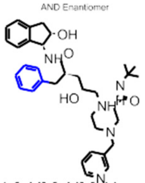 [*][c]1:[cH]:[cH]:[cH]:[cH]:[cH]:1	-0.504	12 out of 64

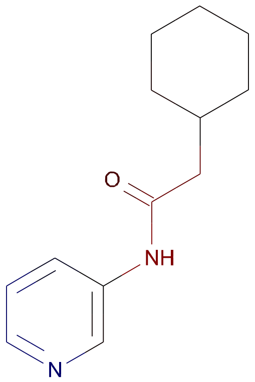

$C_{13}H_{18}N_2O$
Molecular Weight: 218.29481
ALogP: 2.171
Rotatable Bonds: 3
Acceptors: 2
Donors: 1

Model Prediction

Prediction: Multiple-Carcinogen
Probability: 0.569
Enrichment: 1.374
Bayesian Score: 1.127
Mahalanobis Distance: 11.090
Mahalanobis Distance p-value: 0.0215

Prediction: Positive if the Bayesian score is above the estimated best cutoff value from minimizing the false positive and false negative rate.
Probability: The esimated probability that the sample is in the positive category. This assumes that the Bayesian score follows a normal distribution and is different from the prediction using a cutoff.
Enrichment: An estimate of enrichment, that is, the increased likelihood (versus random) of this sample being in the category.
Bayesian Score: The standard Laplacian-modified Bayesian score.
Mahalanobis Distance: The Mahalanobis distance (MD) is the distance to the center of the training data. The larger the MD, the less trustworthy the prediction.
Mahalanobis Distance p-value: The p-value gives the fraction of training data with an MD greater than or equal to the one for the given sample, assuming normally distributed data. The smaller the p-value, the less trustworthy the prediciton. For highly non-normal X properties (e.g., fingerprints), the MD p-value is wildly inaccurate.

Structural Similar Compounds

Name	Phenacetin	Lidocaine	Flutamide
Structure	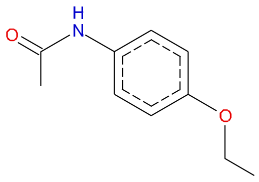	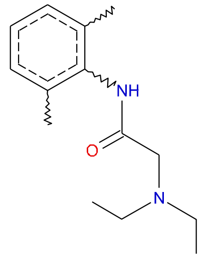	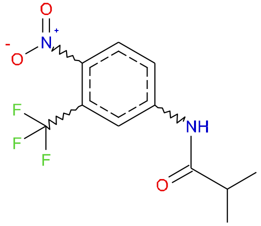
Actual Endpoint	Multiple-Carcinogen	Multiple-Carcinogen	Multiple-Carcinogen
Predicted Endpoint	Multiple-Carcinogen	Multiple-Carcinogen	Multiple-Carcinogen
Distance	0.525	0.588	0.628
Reference	US FDA (Centre for Drug Eval.& Res./Off. Testing & Res.) Sept. 1997	US FDA (Centre for Drug Eval.& Res./Off. Testing & Res.) Sept. 1997	US FDA (Centre for Drug Eval.& Res./Off. Testing & Res.) Sept. 1997

Model Applicability

Unknown features are fingerprint features in the query molecule, but not found in the training set.

1. All properties and OPS components are within expected ranges.

Feature Contribution

Top features for positive contribution

Fingerprint	Bit/Smiles	Feature Structure	Score	Multiple-Carcinogen in training set
SCFP_8	2097618059	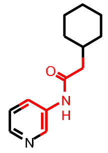	0.681	6 out of 7

SCFP_8	1631845520	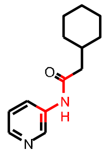 [*]C(=[*])N[c](:[*]):[*]	0.495	6 out of 9
SCFP_8	-1529004787	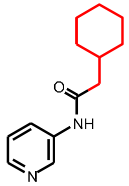 [*]CC1CCCCC1	0.383	1 out of 1

Top Features for negative contribution

**Fingerprint**	**Bit/Smiles**	**Feature Structure**	**Score**	**Multiple-Carcinogen in training set**
SCFP_8	-758850909	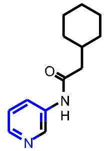 [*][c]1:[*]:n:[cH]:[cH]:[cH]:1	-1.035	0 out of 5
SCFP_8	-937094999	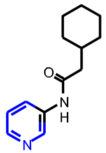 [*]1:[cH]:[cH]:[cH]:n:[cH]:1	-0.463	1 out of 6
SCFP_8	-105808146	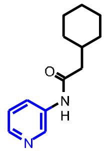 [*][c]1:[cH]:[cH]:[cH]:n:[cH]:1	-0.310	0 out of 1

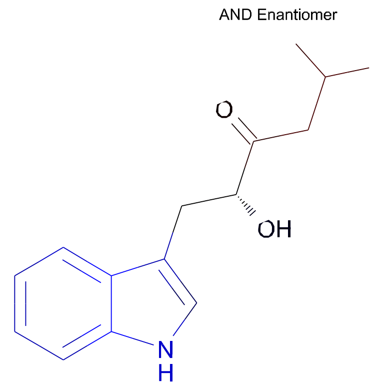

$C_{15}H_{19}NO_2$
Molecular Weight: 245.31685
ALogP: 2.932
Rotatable Bonds: 5
Acceptors: 2
Donors: 2

Structural Similar Compounds

Name	Phenol, 4,4'-isopropylidenedi-	1,7-Octanediol, 3,7-dimethyl-	o-Toluidine, N,N-bis(2-hydroxyethyl)-
Structure	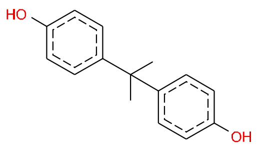	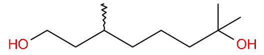	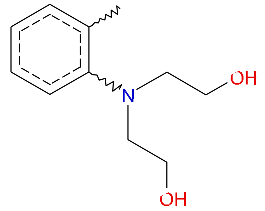
Actual Endpoint	Mild	Mild	Mild
Predicted Endpoint	Mild	Mild	Mild
Distance	0.640	0.646	0.647
Reference	85JCAE "Prehled Prumyslove Toxikologie; Organicke Latky," Marhold, J., Prague , Czechoslovakia, Avicenum, 1986 Volume(issue)/page/year: -,238,1986	FCTXAV Food and Cosmetics Toxicology. (London, UK) V.1-19, 1963-81. For pu blisher information, see FCTOD7. Volume(issue)/page/year: 12,923,1974	85JCAE "Prehled Prumyslove Toxikologie; Organicke Latky," Marhold, J., Prague , Czechoslovakia, Avicenum, 1986 Volume(issue)/page/year: -,697,1986

Model Prediction

Prediction: Mild
Probability: 0.137
Enrichment: 0.371
Bayesian Score: -7.024
Mahalanobis Distance: 8.853
Mahalanobis Distance p-value: 0.35

Prediction: Positive if the Bayesian score is above the estimated best cutoff value from minimizing the false positive and false negative rate.
Probability: The esimated probability that the sample is in the positive category. This assumes that the Bayesian score follows a normal distribution and is different from the prediction using a cutoff.
Enrichment: An estimate of enrichment, that is, the increased likelihood (versus random) of this sample being in the category.
Bayesian Score: The standard Laplacian-modified Bayesian score.
Mahalanobis Distance: The Mahalanobis distance (MD) is the distance to the center of the training data. The larger the MD, the less trustworthy the prediction.
Mahalanobis Distance p-value: The p-value gives the fraction of training data with an MD greater than or equal to the one for the given sample, assuming normally distributed data. The smaller the p-value, the less trustworthy the prediciton. For highly non-normal X properties (e.g., fingerprints), the MD p-value is wildly inaccurate.

Model Applicability

Unknown features are fingerprint features in the query molecule, but not found in the training set.

1. All properties and OPS components are within expected ranges.
2. Unknown FCFP_2 feature: 1618184456: [*][c]1:[*]:[*]:[nH]:c:1

Feature Contribution

Top features for positive contribution

Fingerprint	Bit/Smiles	Feature Structure	Score	Moderate_Severe in training set
FCFP_12	-1870530637	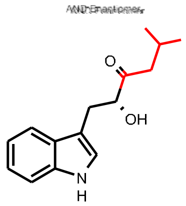 [*]C(=[*])CC(C)C	0.448	22 out of 38

FCFP_12	-1043339860	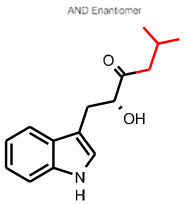 [*]CC(C)C	0.299	101 out of 207
FCFP_12	907007053	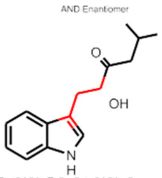 [*]C([*])C[c](:[*]):[*]	0.245	27 out of 58
**Top Features for negative contribution**				
**Fingerprint**	**Bit/Smiles**	**Feature Structure**	**Score**	**Moderate_Severe in training set**
FCFP_12	307419094	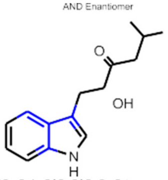 [*][c]1:[*]:[*]:[c](:[*]):[c]:1:[cH]:[*]	-0.915	2 out of 18
FCFP_12	-1320007763	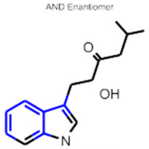 [*][c]1:[*]:[*]:[c]2:[*]:[cH]:[cH]:[cH]:[c]:1:2	-0.759	2 out of 15
FCFP_12	-387072142	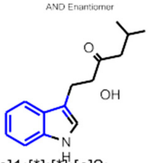 [*][c]1:[*]:[*]:[c]2:[cH]:[cH]:[cH]:[cH]:[c]:1:2	-0.753	1 out of 9

Indinavir

TOPKAT_Skin_Irritancy_Mild_vs_Moderate_Severe

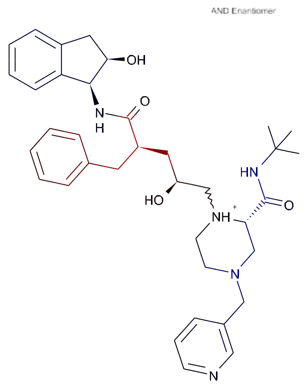

$C_{36}H_{48}N_5O_4$

Molecular Weight: 614.79741

ALogP: 1.521

Rotatable Bonds: 12

Acceptors: 6

Donors: 5

Model Prediction

Prediction: Mild

Probability: 0.127

Enrichment: 0.344

Bayesian Score: -7.357

Mahalanobis Distance: 13.759

Mahalanobis Distance p-value: 1.57e-010

Prediction: Positive if the Bayesian score is above the estimated best cutoff value from minimizing the false positive and false negative rate.
Probability: The esimated probability that the sample is in the positive category. This assumes that the Bayesian score follows a normal distribution and is different from the prediction using a cutoff.
Enrichment: An estimate of enrichment, that is, the increased likelihood (versus random) of this sample being in the category.
Bayesian Score: The standard Laplacian-modified Bayesian score.
Mahalanobis Distance: The Mahalanobis distance (MD) is the distance to the center of the training data. The larger the MD, the less trustworthy the prediction.
Mahalanobis Distance p-value: The p-value gives the fraction of training data with an MD greater than or equal to the one for the given sample, assuming normally distributed data. The smaller the p-value, the less trustworthy the prediciton. For highly non-normal X properties (e.g., fingerprints), the MD p-value is wildly inaccurate.

Structural Similar Compounds

Name	Anthraquinone, 3-methoxy-5,4'-iminobis(1-benzamido-	Benzenesulfonic acid, 2,2'-(4,4'-biphenylylenedivinylene)di-, disod ium salt	2-Anthracenesulfonic acid, 1-amino-9,10-dihydro-9,10-dioxo-4-(2,4,6 -trimethylanilino)-, monosodium salt
Structure	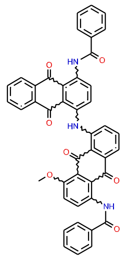	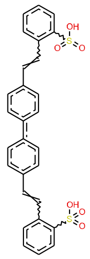	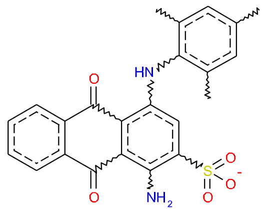
Actual Endpoint	Moderate_Severe	Mild	Mild
Predicted Endpoint	Moderate_Severe	Mild	Mild
Distance	1.219	1.283	1.337
Reference	28ZPAK "Sbornik Vysledku Toxixologickeho Vysetreni Latek A Pripravku," Marhol d, J.V., Institut Pro Vychovu Vedoucicn Pracovniku Chemickeho Prumyclu Praha, Cz echoslovakia, 1972 Volume(issue)/page/year: -,114,1	MVCRB3 MVC-Report. (Stockholm, Sweden) No.1-2, 1972-73. Discontinued. Volu me(issue)/page/year: 2,193,1973	85JCAE "Prehled Prumyslove Toxikologie; Organicke Latky," Marhold, J., Prague , Czechoslovakia, Avicenum, 1986 Volume(issue)/page/year: -,1327,1986

Model Applicability

Unknown features are fingerprint features in the query molecule, but not found in the training set.

1. All properties and OPS components are within expected ranges.
2. Unknown FCFP_2 feature: 10: [*][NH+]([*])[*]
3. Unknown FCFP_2 feature: -1853714334: [*]C[NH+](C[*])C([*])[*]
4. Unknown FCFP_2 feature: -1817836174: [*]C[C@H]([NH+]([*])[*])C(=[*])[*]
5. Unknown FCFP_2 feature: 1155241219: [*]CC[NH+]([*])[*]

Feature Contribution

Top features for positive contribution				
Fingerprint	Bit/Smiles	Feature Structure	Score	Moderate_Severe in training set

FCFP_12	-2005486458	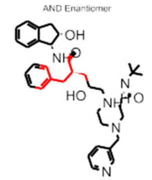 [*]C[C@@H](C[c](:[cH]:[*]):[cH]:[*])C(=[*])[*]	0.717	4 out of 4
FCFP_12	1384456758	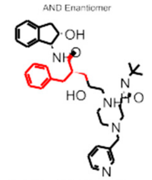 [*]C[C@@H](C[c]1:[cH]:[cH]:[cH]:[cH]:[cH]:1)C(=[*])[*]	0.653	3 out of 3
FCFP_12	-59531427	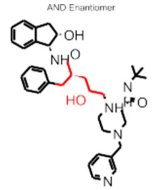 [*]C[C@@H](O)C[C@@H](C[*])C(=[*])[*]	0.350	4 out of 7
**Top Features for negative contribution**				
**Fingerprint**	**Bit/Smiles**	**Feature Structure**	**Score**	**Moderate_Severe in training set**
FCFP_12	-885550502	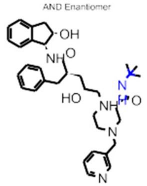 [*]C(=[*])NC([*])([*])[*]	-1.050	2 out of 21
FCFP_12	566058135	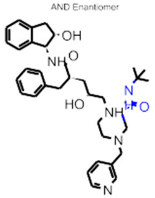 [*]NC(=O)C([*])[*]	-0.909	1 out of 11

FCFP_12	-587569116	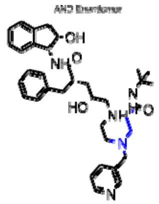 [*]C([*])CN([*])[*]	-	4 out of 27

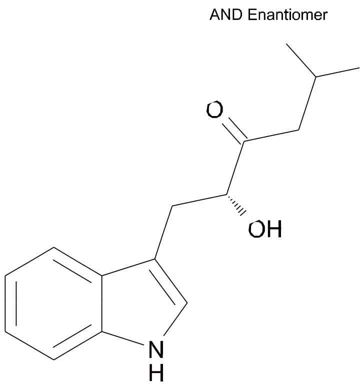

$C_{15}H_{19}NO_2$
Molecular Weight: 245.31685
ALogP: 2.932
Rotatable Bonds: 5
Acceptors: 2
Donors: 2

Model Prediction

Prediction: Irritant
Probability: 0.976
Enrichment: 1.060
Bayesian Score: -0.426
Mahalanobis Distance: 9.157
Mahalanobis Distance p-value: 0.34

Prediction: Positive if the Bayesian score is above the estimated best cutoff value from minimizing the false positive and false negative rate.
Probability: The esimated probability that the sample is in the positive category. This assumes that the Bayesian score follows a normal distribution and is different from the prediction using a cutoff.
Enrichment: An estimate of enrichment, that is, the increased likelihood (versus random) of this sample being in the category.
Bayesian Score: The standard Laplacian-modified Bayesian score.
Mahalanobis Distance: The Mahalanobis distance (MD) is the distance to the center of the training data. The larger the MD, the less trustworthy the prediction.
Mahalanobis Distance p-value: The p-value gives the fraction of training data with an MD greater than or equal to the one for the given sample, assuming normally distributed data. The smaller the p-value, the less trustworthy the prediciton. For highly non-normal X properties (e.g., fingerprints), the MD p-value is wildly inaccurate.

Structural Similar Compounds

Name	Benzoin, oxime	Aniline, 4,4'-methylenebis(2-methyl-	Disiloxane, 1,3-bis(3-aminopropyl)-1,1,3,3-tetramethyl-
Structure	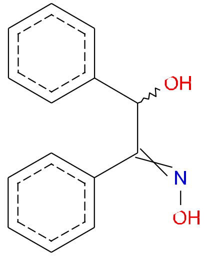	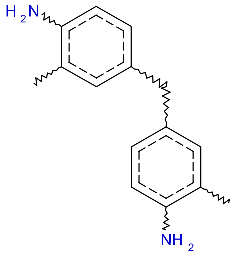	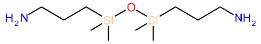
Actual Endpoint	Non-Irritant	Non-Irritant	Irritant
Predicted Endpoint	Non-Irritant	Non-Irritant	Irritant
Distance	0.593	0.610	0.631
Reference	28ZPAK -,111,72	28ZPAK -,72,72	NTIS** National Technical Information Service. (Springfield, VA 22161) Forme rly U.S. Clearinghouse for Scientific & Technical Information. Volume(issue)/pag e/year: OTS0535667

Model Applicability

Unknown features are fingerprint features in the query molecule, but not found in the training set.

1. All properties and OPS components are within expected ranges.
2. Unknown FCFP_2 feature: 1618184456: [*][c]1:[*]:[*]:[nH]:c:1

Feature Contribution

Top features for positive contribution				
Fingerprint	Bit/Smiles	Feature Structure	Score	Irritant in training set

FCFP_12	-1870530637	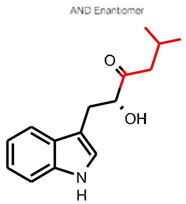 [*]C(=[*])CC(C)C	0.087	44 out of 44
FCFP_12	1306984497	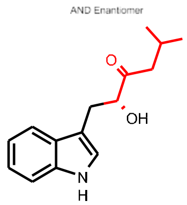 [*]C([*])C(=O)CC(C)C	0.081	11 out of 11
FCFP_12	565968762	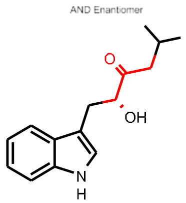 [*]CC(=O)C([*])[*]	0.075	78 out of 79
**Top Features for negative contribution**				
**Fingerprint**	**Bit/Smiles**	**Feature Structure**	**Score**	**Irritant in training set**
FCFP_12	-1320007763	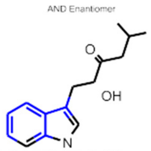 [*][c]1:[*]:[*]:[c]2:[*]:[cH]:[cH]:[cH]:[c]:1:2	-0.089	20 out of 24
FCFP_12	1618154665	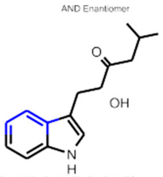 [*]:[cH]:[cH]:[c](:[*]):[*]	-0.084	412 out of 490

FCFP_12	16	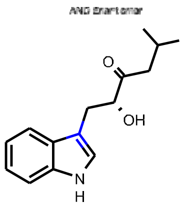 [*][c](:[*]):[*]	-	423 out of 503

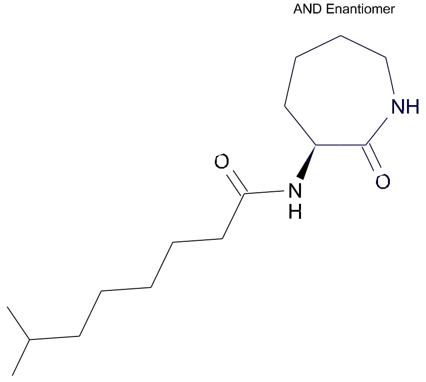

$C_{15}H_{28}N_2O_2$
Molecular Weight: 268.39501
ALogP: 2.685
Rotatable Bonds: 7
Acceptors: 2
Donors: 2

Model Prediction

Prediction: Non-Irritant
Probability: 0.965
Enrichment: 1.048
Bayesian Score: -1.312
Mahalanobis Distance: 12.242
Mahalanobis Distance p-value: 7.39e-006

Prediction: Positive if the Bayesian score is above the estimated best cutoff value from minimizing the false positive and false negative rate.
Probability: The esimated probability that the sample is in the positive category. This assumes that the Bayesian score follows a normal distribution and is different from the prediction using a cutoff.
Enrichment: An estimate of enrichment, that is, the increased likelihood (versus random) of this sample being in the category.
Bayesian Score: The standard Laplacian-modified Bayesian score.
Mahalanobis Distance: The Mahalanobis distance (MD) is the distance to the center of the training data. The larger the MD, the less trustworthy the prediction.
Mahalanobis Distance p-value: The p-value gives the fraction of training data with an MD greater than or equal to the one for the given sample, assuming normally distributed data. The smaller the p-value, the less trustworthy the prediciton. For highly non-normal X properties (e.g., fingerprints), the MD p-value is wildly inaccurate.

Structural Similar Compounds

Name	Disiloxane, 1,3-bis(3-aminopropyl)-1,1,3,3-tetramethyl-	1,7-Octanediol, 3,7-dimethyl-	2,2,2-Trichloro-N-pentyl acetamide
Structure	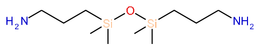	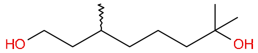	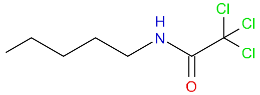
Actual Endpoint	Irritant	Irritant	Non-Irritant
Predicted Endpoint	Irritant	Irritant	Non-Irritant
Distance	0.569	0.677	0.698
Reference	NTIS** National Technical Information Service. (Springfield, VA 22161) Forme rly U.S. Clearinghouse for Scientific & Technical Information. Volume(issue)/pag e/year: OTS0535667	FCTXAV Food and Cosmetics Toxicology. (London, UK) V.1-19, 1963-81. For pu blisher information, see FCTOD7. Volume(issue)/page/year: 12,923,1974	US ARMY

Model Applicability

Unknown features are fingerprint features in the query molecule, but not found in the training set.

1. All properties and OPS components are within expected ranges.

Feature Contribution

Top features for positive contribution

Fingerprint	Bit/Smiles	Feature Structure	Score	Irritant in training set

FCFP_12	-1870530637	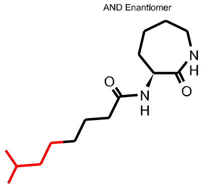 [*]C(=[*])CC(C)C	0.087	44 out of 44
FCFP_12	-154166589	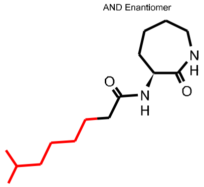 [*]CCCCC(C)C	0.078	8 out of 8
FCFP_12	-551279842	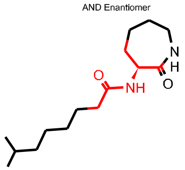 [*]C[C@H](NC(=O)C[*])C(=[*])[*]	0.066	3 out of 3

Top Features for negative contribution

Fingerprint	Bit/Smiles	Feature Structure	Score	Irritant in training set
FCFP_12	566058135	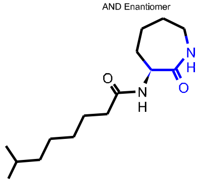 [*]NC(=O)C([*])[*]	-0.367	13 out of 21
FCFP_12	901946715	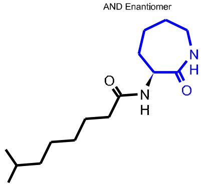 [*][C@H]1CCCCNC1=O	-0.347	1 out of 2

FCFP_12	1395495016	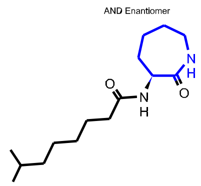 [*][C@H]1CCCCNC1=[*]	-	2 out of 3

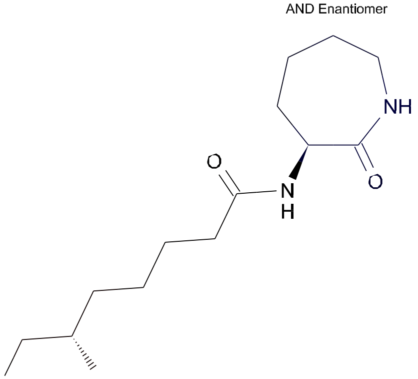

$C_{15}H_{28}N_2O_2$
Molecular Weight: 268.39501
ALogP: 2.685
Rotatable Bonds: 7
Acceptors: 2
Donors: 2

Model Prediction

Prediction: Non-Irritant
Probability: 0.967
Enrichment: 1.050
Bayesian Score: -1.225
Mahalanobis Distance: 11.180
Mahalanobis Distance p-value: 0.0012

Prediction: Positive if the Bayesian score is above the estimated best cutoff value from minimizing the false positive and false negative rate.
Probability: The esimated probability that the sample is in the positive category. This assumes that the Bayesian score follows a normal distribution and is different from the prediction using a cutoff.
Enrichment: An estimate of enrichment, that is, the increased likelihood (versus random) of this sample being in the category.
Bayesian Score: The standard Laplacian-modified Bayesian score.
Mahalanobis Distance: The Mahalanobis distance (MD) is the distance to the center of the training data. The larger the MD, the less trustworthy the prediction.
Mahalanobis Distance p-value: The p-value gives the fraction of training data with an MD greater than or equal to the one for the given sample, assuming normally distributed data. The smaller the p-value, the less trustworthy the prediciton. For highly non-normal X properties (e.g., fingerprints), the MD p-value is wildly inaccurate.

Structural Similar Compounds

Name	Disiloxane, 1,3-bis(3-aminopropyl)-1,1,3,3-tetramethyl-	1,7-Octanediol, 3,7-dimethyl-	2,2,2-Trichloro-N-pentyl acetamide
Structure	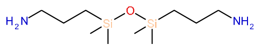	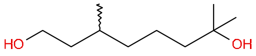	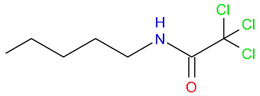
Actual Endpoint	Irritant	Irritant	Non-Irritant
Predicted Endpoint	Irritant	Irritant	Non-Irritant
Distance	0.570	0.662	0.700
Reference	NTIS** National Technical Information Service. (Springfield, VA 22161) Forme rly U.S. Clearinghouse for Scientific & Technical Information. Volume(issue)/pag e/year: OTS0535667	FCTXAV Food and Cosmetics Toxicology. (London, UK) V.1-19, 1963-81. For pu blisher information, see FCTOD7. Volume(issue)/page/year: 12,923,1974	US ARMY

Model Applicability

Unknown features are fingerprint features in the query molecule, but not found in the training set.

1. OPS PC25 out of range. Value: -3.7613. Training min, max, SD, explained variance: -3.2549, 4.4957, 0.9952, 0.0125.

Feature Contribution

Top features for positive contribution

Fingerprint	Bit/Smiles	Feature Structure	Score	Irritant in training set

FCFP_12	882707499	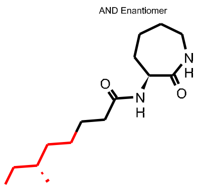 [*]CC[C@H](C)CC	0.086	37 out of 37
FCFP_12	-1580903393	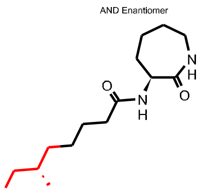 [*]C[C@H](C)CC	0.084	20 out of 20
FCFP_12	-150334802	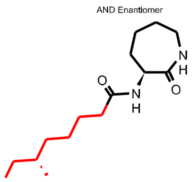 [*]CCCC[C@H](C)CC	0.081	11 out of 11

Top Features for negative contribution

**Fingerprint**	**Bit/Smiles**	**Feature Structure**	**Score**	**Irritant in training set**
FCFP_12	566058135	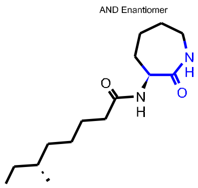 [*]NC(=O)C([*])[*]	-0.367	13 out of 21
FCFP_12	901946715	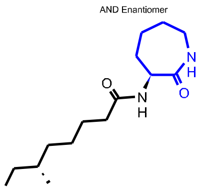 [*][C@H]1CCCCNC1=O	-0.347	1 out of 2

FCFP_12	-547731249	AND Enantiomer<br>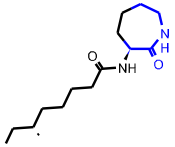<br>[*][C@H]1[*][*]CCNC1=<br>O	-	2 out of 3

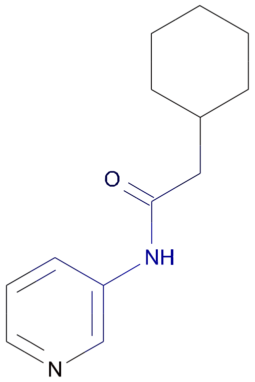

$C_{13}H_{18}N_2O$
Molecular Weight: 218.29481
ALogP: 2.171
Rotatable Bonds: 3
Acceptors: 2
Donors: 1

Model Prediction

Prediction: Non-Irritant
Probability: 0.918
Enrichment: 0.997
Bayesian Score: -2.525
Mahalanobis Distance: 8.218
Mahalanobis Distance p-value: 0.812

Prediction: Positive if the Bayesian score is above the estimated best cutoff value from minimizing the false positive and false negative rate.
Probability: The esimated probability that the sample is in the positive category. This assumes that the Bayesian score follows a normal distribution and is different from the prediction using a cutoff.
Enrichment: An estimate of enrichment, that is, the increased likelihood (versus random) of this sample being in the category.
Bayesian Score: The standard Laplacian-modified Bayesian score.
Mahalanobis Distance: The Mahalanobis distance (MD) is the distance to the center of the training data. The larger the MD, the less trustworthy the prediction.
Mahalanobis Distance p-value: The p-value gives the fraction of training data with an MD greater than or equal to the one for the given sample, assuming normally distributed data. The smaller the p-value, the less trustworthy the prediciton. For highly non-normal X properties (e.g., fingerprints), the MD p-value is wildly inaccurate.

Structural Similar Compounds

Name	Carbamic acid, methyl-, 1-naphthyl ester	1-Penten-3-one, 1-(p-methoxyphenyl)-	Aniline, p-phenoxy-
Structure	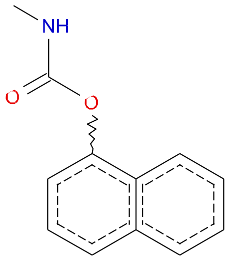	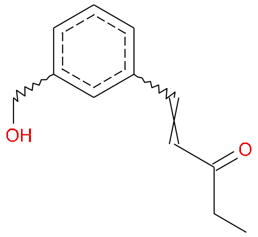	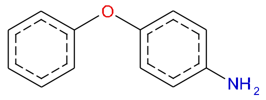
Actual Endpoint	Irritant	Irritant	Irritant
Predicted Endpoint	Non-Irritant	Irritant	Non-Irritant
Distance	0.527	0.532	0.554
Reference	JAFCAU Journal of Agricultural and Food Chemistry. (American Chemical Soc., Distribution Office Dept. 223, POB 57136, West End Stn., Washington, DC 20037) V.1- 1953- Volume(issue)/page/year: 9,30,1961	FCTXAV Food and Cosmetics Toxicology. (London, UK) V.1-19, 1963-81. For pu blisher information, see FCTOD7. Volume(issue)/page/year: 17,863,1979	85JCAE "Prehled Prumyslove Toxikologie; Organicke Latky," Marhold, J., Prague , Czechoslovakia, Avicenum, 1986 Volume(issue)/page/year: -,723,1986

Model Applicability

Unknown features are fingerprint features in the query molecule, but not found in the training set.

1. All properties and OPS components are within expected ranges.

Feature Contribution

Top features for positive contribution

Fingerprint	Bit/Smiles	Feature Structure	Score	Irritant in training set

FCFP_12	-124655670	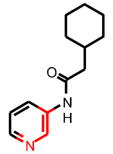 [*][c](:[*]):[cH]:n:[*]	0.082	13 out of 13
FCFP_12	-1695756380	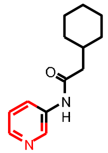 [*]1:[cH]:[cH]:[cH]:n :[cH]:1	0.077	7 out of 7
FCFP_12	1940464803	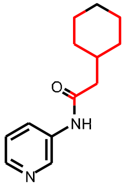 [*]C(=[*])CC1CC[*]CC1	0.077	7 out of 7

Top Features for negative contribution

Fingerprint	Bit/Smiles	Feature Structure	Score	Irritant in training set
FCFP_12	1175665944	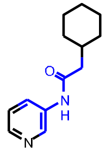 [*]CC(=O)N[c](:[cH]:[*]):[cH]:[*]	-1.020	2 out of 8
FCFP_12	1294255210	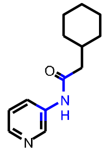 [*]C(=[*])N[c](:[*]): [*]	-0.486	12 out of 22

FCFP_12	-773983804	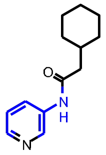<br>[*]N[c]1:[cH]:[*]:[cH]:[cH]:[cH]:1	-	46 out of 79

Indinavir

TOPKAT_Skin_Irritancy_None_vs_Irritant

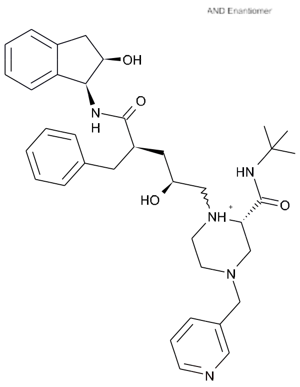

$C_{36}H_{48}N_5O_4$

Molecular Weight: 614.79741

ALogP: 1.521

Rotatable Bonds: 12

Acceptors: 6

Donors: 5

Model Prediction

Prediction: Irritant

Probability: 0.975

Enrichment: 1.058

Bayesian Score: -0.618

Mahalanobis Distance: 14.010

Mahalanobis Distance p-value: 8.25e-011

Prediction: Positive if the Bayesian score is above the estimated best cutoff value from minimizing the false positive and false negative rate.
Probability: The esimated probability that the sample is in the positive category. This assumes that the Bayesian score follows a normal distribution and is different from the prediction using a cutoff.
Enrichment: An estimate of enrichment, that is, the increased likelihood (versus random) of this sample being in the category.
Bayesian Score: The standard Laplacian-modified Bayesian score.
Mahalanobis Distance: The Mahalanobis distance (MD) is the distance to the center of the training data. The larger the MD, the less trustworthy the prediction.
Mahalanobis Distance p-value: The p-value gives the fraction of training data with an MD greater than or equal to the one for the given sample, assuming normally distributed data. The smaller the p-value, the less trustworthy the prediciton. For highly non-normal X properties (e.g., fingerprints), the MD p-value is wildly inaccurate.

Structural Similar Compounds

Name	Anthraquinone, 3-methoxy-5,4'-iminobis(1-benzamido-	Benzenesulfonic acid, 2,2'-(4,4'-biphenylylenedivinylene)di-, disod ium salt	2-Anthracenesulfonic acid, 1-amino-9,10-dihydro-9,10-dioxo-4-(2,4,6 -trimethylanilino)-, monosodium salt
Structure	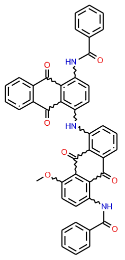	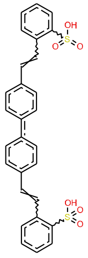	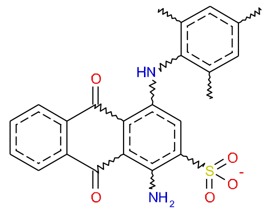
Actual Endpoint	Irritant	Irritant	Irritant
Predicted Endpoint	Non-Irritant	Non-Irritant	Non-Irritant
Distance	1.222	1.269	1.328
Reference	28ZPAK "Sbornik Vysledku Toxixologickeho Vysetreni Latek A Pripravku," Marhol d, J.V., Institut Pro Vychovu Vedoucicn Pracovniku Chemickeho Prumyclu Praha, Cz echoslovakia, 1972 Volume(issue)/page/year: -,114,1	MVCRB3 MVC-Report. (Stockholm, Sweden) No.1-2, 1972-73. Discontinued. Volu me(issue)/page/year: 2,193,1973	85JCAE "Prehled Prumyslove Toxikologie; Organicke Latky," Marhold, J., Prague , Czechoslovakia, Avicenum, 1986 Volume(issue)/page/year: -,1327,1986

Model Applicability

Unknown features are fingerprint features in the query molecule, but not found in the training set.

1. All properties and OPS components are within expected ranges.
2. Unknown FCFP_2 feature: -1853714334: [*]C[NH+](C[*])C([*])[*]
3. Unknown FCFP_2 feature: -1817836174: [*]C[C@H]([NH+]([*])[*])C(=[*])[*]
4. Unknown FCFP_2 feature: 1155241219: [*]CC[NH+]([*])[*]

Feature Contribution

Top features for positive contribution				
Fingerprint	Bit/Smiles	Feature Structure	Score	Irritant in training set

FCFP_12	-415156552	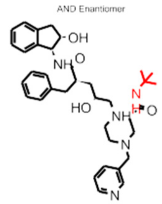 [*]NC(C)(C)C	0.085	27 out of 27
FCFP_12	-124655670	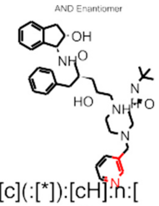 [*][c](:[*]):[cH]:n:[*]	0.082	13 out of 13
FCFP_12	-1695756380	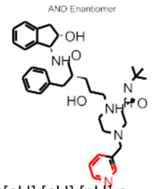 [*]1:[cH]:[cH]:[cH]:n:[cH]:1	0.077	7 out of 7

Top Features for negative contribution

Fingerprint	Bit/Smiles	Feature Structure	Score	Irritant in training set
FCFP_12	566058135	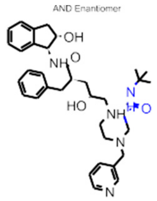 [*]NC(=O)C([*])[*]	-0.367	13 out of 21
FCFP_12	-1947828591	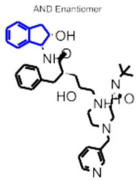 [*][C@@H]1C[c]2:[cH]:[cH]:[cH]:[cH]:[c]:2[C@@H]1[*]	-0.210	5 out of 7

FCFP_12	-1943140669	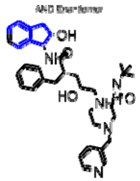<br>[*][C@@H]1C[c]2:[cH]: [cH]:[*]:[cH]:[c]:2[C@@H]1[*]	-	7 out of 9

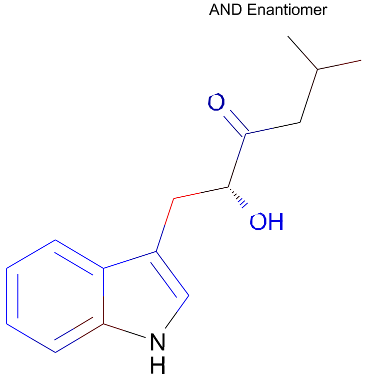

$C_{15}H_{19}NO_2$
Molecular Weight: 245.31685
ALogP: 2.932
Rotatable Bonds: 5
Acceptors: 2
Donors: 2

Model Prediction

Prediction: 136.851
Unit: mg/kg_body_weight/day
Mahalanobis Distance: 10.202
Mahalanobis Distance p-value: 0.0204

Mahalanobis Distance: The Mahalanobis distance (MD) is a generalization of the Euclidean distance that accounts for correlations among the X properties. It is calculated as the distance to the center of the training data. The larger the MD, the less trustworthy the prediction.
Mahalanobis Distance p-value: The p-value gives the fraction of training data with an MD greater than or equal to the one for the given sample, assuming normally distributed data. The smaller the p-value, the less trustworthy the prediciton. For highly non-normal X properties (e.g., fingerprints), the MD p-value is wildly inaccurate.

Structural Similar Compounds

Name	171	Cinnamyl anthranilate	5
Structure	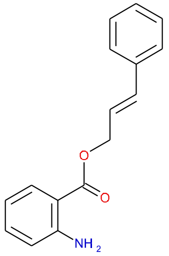	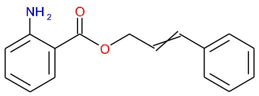	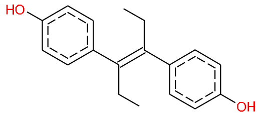
Actual Endpoint (-log C)	1.99201	1.99201	6.85816
Predicted Endpoint (-log C)	3.01089	3.01089	3.82521
Distance	0.588	0.588	0.618
Reference	CPDB	CPDB	CPDB

Model Applicability

Unknown features are fingerprint features in the query molecule, but not found in the training set.

1. All properties and OPS components are within expected ranges.
2. Unknown ECFP_2 feature: 80433051: [*]C[C@@H](O)C(=[*])[*]
3. Unknown ECFP_2 feature: 1732075620: [*]CC(=O)C([*])[*]
4. Unknown ECFP_2 feature: -1020449580: [*][c]1:[*]:[*]:[nH]:c:1

Feature Contribution

Top features for positive contribution

Fingerprint	Bit/Smiles	Feature Structure	Score
ECFP_6	1559650422	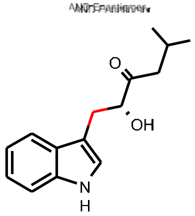 [*]C[*]	0.203

ECFP_6	1333660716	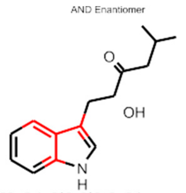<br>[*][c]1:[*]:[*]:[c](:<br>[*]):[c]:1:[cH]:[*]	0.075
ECFP_6	734603939	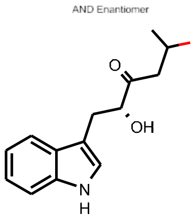<br>[*]C	0.042

Top Features for negative contribution

Fingerprint	Bit/Smiles	Feature Structure	Score
ECFP_6	1996767644	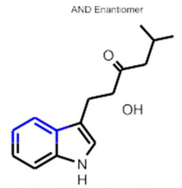<br>[*]:[cH]:[cH]:[c](:[*<br>]):[*]	-0.251
ECFP_6	642810091	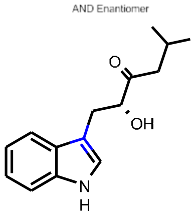<br>[*][c](:[*]):[*]	-0.247
ECFP_6	-182236392	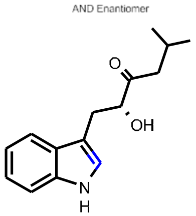<br>[*]:[cH]:[*]	-0.232

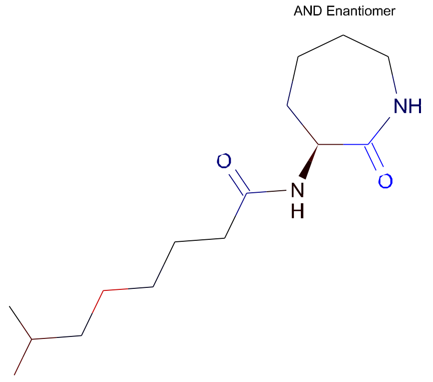

$C_{15}H_{28}N_2O_2$
Molecular Weight: 268.39501
ALogP: 2.685
Rotatable Bonds: 7
Acceptors: 2
Donors: 2

Model Prediction

Prediction: 104.247
Unit: mg/kg_body_weight/day
Mahalanobis Distance: 12.506
Mahalanobis Distance p-value: 1.72e-006

Mahalanobis Distance: The Mahalanobis distance (MD) is a generalization of the Euclidean distance that accounts for correlations among the X properties. It is calculated as the distance to the center of the training data. The larger the MD, the less trustworthy the prediction.
Mahalanobis Distance p-value: The p-value gives the fraction of training data with an MD greater than or equal to the one for the given sample, assuming normally distributed data. The smaller the p-value, the less trustworthy the prediciton. For highly non-normal X properties (e.g., fingerprints), the MD p-value is wildly inaccurate.

Structural Similar Compounds

Name	Capsaicin	816	3-Hydroxy-p-butyrophenetidide
Structure	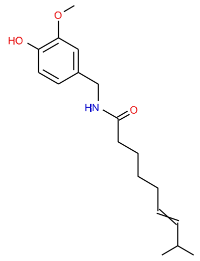	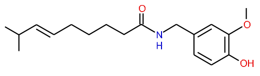	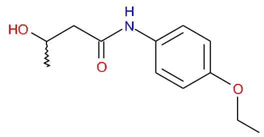
Actual Endpoint (-log C)	3.26217	3.26217	1.6061
Predicted Endpoint (-log C)	2.84959	2.84959	2.97088
Distance	0.649	0.649	0.668
Reference	CPDB	CPDB	CPDB

Model Applicability

Unknown features are fingerprint features in the query molecule, but not found in the training set.

1. All properties and OPS components are within expected ranges.
2. Unknown ECFP_2 feature: -2097159651: [*]C[C@H](N[*])C(=[*])[*]

Feature Contribution

Top features for positive contribution

Fingerprint	Bit/Smiles	Feature Structure	Score
ECFP_6	1559650422	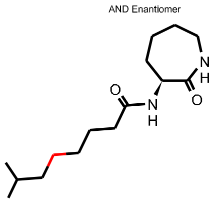 [*]C[*]	0.203

ECFP_6	-167460056	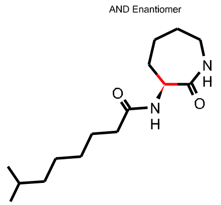 [*]C([*])[*]	0.060
ECFP_6	734603939	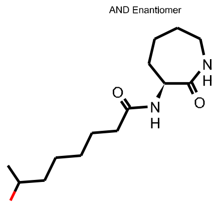 [*]C	0.042

Top Features for negative contribution

Fingerprint	Bit/Smiles	Feature Structure	Score
ECFP_6	2106656448	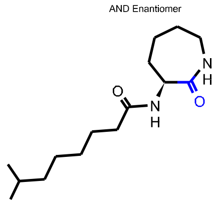 [*]C(=O)[*]	-0.275
ECFP_6	642810091	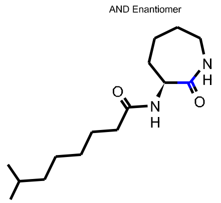 [*][c](:[*]):[*]	-0.247
ECFP_6	2099970318	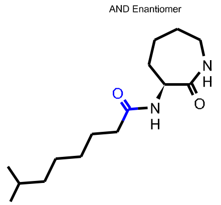 [*]C(=O)[*]	-0.118

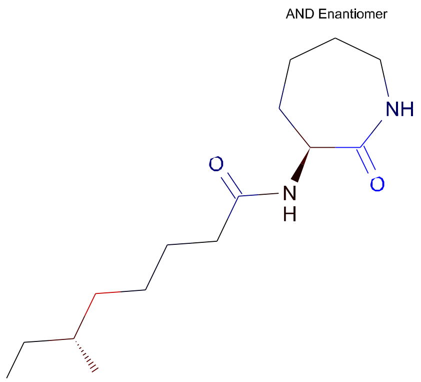

$C_{15}H_{28}N_2O_2$
Molecular Weight: 268.39501
ALogP: 2.685
Rotatable Bonds: 7
Acceptors: 2
Donors: 2

Model Prediction

Prediction: 108.043
Unit: mg/kg_body_weight/day
Mahalanobis Distance: 12.239
Mahalanobis Distance p-value: 6.59e-006

Mahalanobis Distance: The Mahalanobis distance (MD) is a generalization of the Euclidean distance that accounts for correlations among the X properties. It is calculated as the distance to the center of the training data. The larger the MD, the less trustworthy the prediction.
Mahalanobis Distance p-value: The p-value gives the fraction of training data with an MD greater than or equal to the one for the given sample, assuming normally distributed data. The smaller the p-value, the less trustworthy the prediciton. For highly non-normal X properties (e.g., fingerprints), the MD p-value is wildly inaccurate.

Structural Similar Compounds

Name	Capsaicin	816	3-Hydroxy-p-butyrophenetidide
Structure	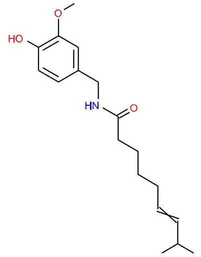	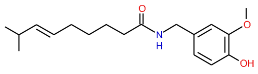	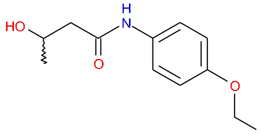
Actual Endpoint (-log C)	3.26217	3.26217	1.6061
Predicted Endpoint (-log C)	2.84959	2.84959	2.97088
Distance	0.651	0.651	0.663
Reference	CPDB	CPDB	CPDB

Model Applicability

Unknown features are fingerprint features in the query molecule, but not found in the training set.

1. All properties and OPS components are within expected ranges.
2. Unknown ECFP_2 feature: -2097159651: [*]C[C@H](N[*])C(=[*])[*]

Feature Contribution

Top features for positive contribution

Fingerprint	Bit/Smiles	Feature Structure	Score
ECFP_6	1559650422	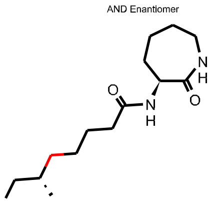 [*]C[*]	0.203

ECFP_6	-167460056	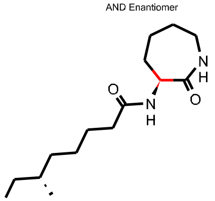 [*]C([*])[*]	0.060
ECFP_6	734603939	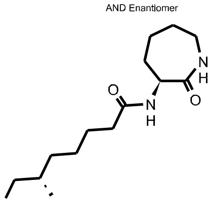 [*]C	0.042

Top Features for negative contribution

Fingerprint	Bit/Smiles	Feature Structure	Score
ECFP_6	2106656448	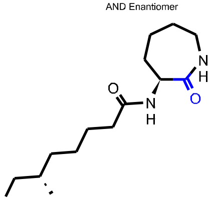 [*]C(=O)[*]	-0.275
ECFP_6	642810091	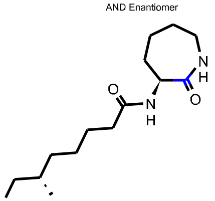 [*][c](:[*]):[*]	-0.247
ECFP_6	2099970318	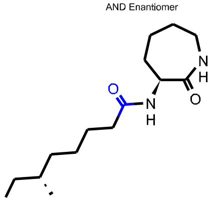 [*]C(=O)[*]	-0.118

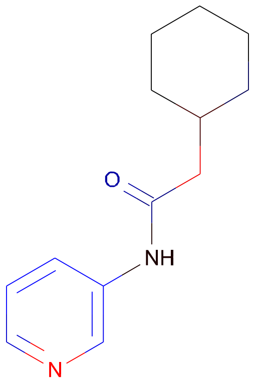

$C_{13}H_{18}N_2O$
Molecular Weight: 218.29481
ALogP: 2.171
Rotatable Bonds: 3
Acceptors: 2
Donors: 1

Model Prediction

Prediction: 61.546
Unit: mg/kg_body_weight/day
Mahalanobis Distance: 10.287
Mahalanobis Distance p-value: 0.016

Mahalanobis Distance: The Mahalanobis distance (MD) is a generalization of the Euclidean distance that accounts for correlations among the X properties. It is calculated as the distance to the center of the training data. The larger the MD, the less trustworthy the prediction.
Mahalanobis Distance p-value: The p-value gives the fraction of training data with an MD greater than or equal to the one for the given sample, assuming normally distributed data. The smaller the p-value, the less trustworthy the prediciton. For highly non-normal X properties (e.g., fingerprints), the MD p-value is wildly inaccurate.

Structural Similar Compounds

Name	666	Phenacetin	1´-Hydroxysafrole
Structure	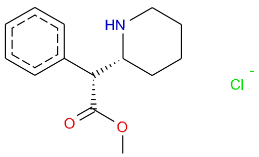	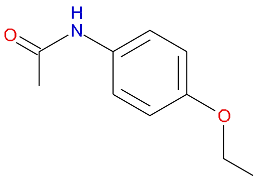	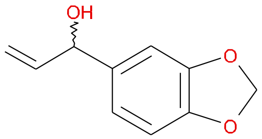
Actual Endpoint (-log C)	3.36793	1.92296	3.39839
Predicted Endpoint (-log C)	2.71924	2.82896	3.24585
Distance	0.511	0.564	0.579
Reference	CPDB	CPDB	CPDB

Model Applicability

Unknown features are fingerprint features in the query molecule, but not found in the training set.

1. All properties and OPS components are within expected ranges.
2. Unknown ECFP_2 feature: -82840383: [*]C([*])CC(=[*])[*]
3. Unknown ECFP_2 feature: -1795620553: [*]CC(C[*])C[*]

Feature Contribution

Top features for positive contribution

Fingerprint	Bit/Smiles	Feature Structure	Score
ECFP_6	655739385	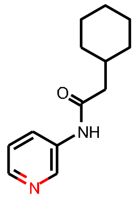 [*]:n:[*]	0.229

ECFP_6	1559650422	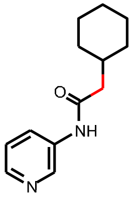 [*]C[*]	0.203
ECFP_6	-167460056	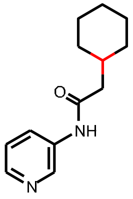 [*]C([*])[*]	0.060
**Top Features for negative contribution**			
**Fingerprint**	**Bit/Smiles**	**Feature Structure**	**Score**
ECFP_6	1996767644	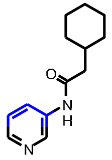 [*]:[cH]:[cH]:[c](:[*]):[*]	-0.251
ECFP_6	642810091	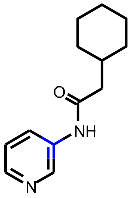 [*][c](:[*]):[*]	-0.247
ECFP_6	-182236392	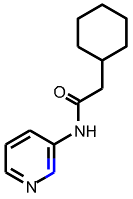 [*]:[cH]:[*]	-0.232

Indinavir

TOPKAT_Carcinogenic_Potency_TD50_Mouse

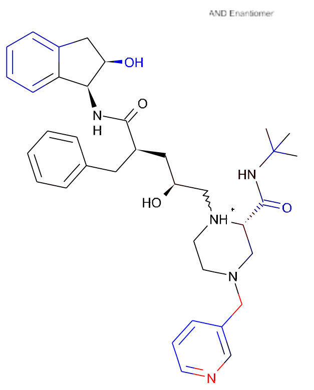

$C_{36}H_{48}N_5O_4$
Molecular Weight: 614.79741
ALogP: 1.521
Rotatable Bonds: 12
Acceptors: 6
Donors: 5

Model Prediction

Prediction: 6.954
Unit: mg/kg_body_weight/day
Mahalanobis Distance: 15.343
Mahalanobis Distance p-value: 4.06e-014

Mahalanobis Distance: The Mahalanobis distance (MD) is a generalization of the Euclidean distance that accounts for correlations among the X properties. It is calculated as the distance to the center of the training data. The larger the MD, the less trustworthy the prediction.
Mahalanobis Distance p-value: The p-value gives the fraction of training data with an MD greater than or equal to the one for the given sample, assuming normally distributed data. The smaller the p-value, the less trustworthy the prediciton. For highly non-normal X properties (e.g., fingerprints), the MD p-value is wildly inaccurate.

Structural Similar Compounds

Name	Ochratoxin A	542	Tamoxifen citrate
Structure	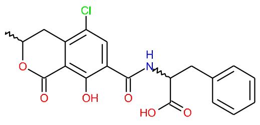	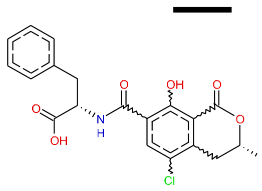	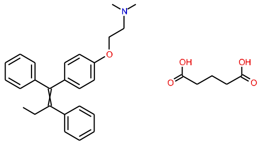
Actual Endpoint (-log C)	4.79932	4.79932	5.05965
Predicted Endpoint (-log C)	3.6353	3.6353	4.24168
Distance	0.994	0.994	1.008
Reference	CPDB	CPDB	CPDB

Model Applicability

Unknown features are fingerprint features in the query molecule, but not found in the training set.

1. All properties and OPS components are within expected ranges.
2. Unknown ECFP_2 feature: 1976330679: [*][NH+]([*])[*]
3. Unknown ECFP_2 feature: 1134829831: [*]C[NH+](C[*])C([*])[*]
4. Unknown ECFP_2 feature: -1924540582: [*]C[C@H]([NH+]([*])[*])C(=[*])[*]
5. Unknown ECFP_2 feature: -244159614: [*]CC[NH+]([*])[*]
6. Unknown ECFP_2 feature: -44121127: [*]N([*])C[c](:[*]):[*]
7. Unknown ECFP_2 feature: 474121058: [*]C([*])C[NH+]([*])[*]
8. Unknown ECFP_2 feature: -1567199489: [*]N[C@@H]1[C@H]([*])[*][*]:[c]1:[*]

Feature Contribution

Top features for positive contribution			
**Fingerprint**	**Bit/Smiles**	**Feature Structure**	**Score**

ECFP_6	655739385	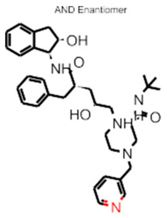 [*]:n:[*]	0.229
ECFP_6	1559650422	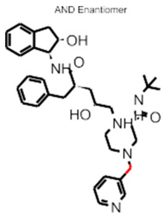 [*]C[*]	0.203
ECFP_6	-2024255407	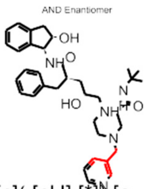 [*]C[c](:[cH]:[*]):[cH]:[*]	0.172

Top Features for negative contribution

**Fingerprint**	**Bit/Smiles**	**Feature Structure**	**Score**
ECFP_6	1996767644	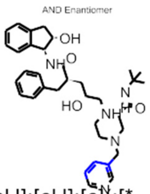 [*]:[cH]:[cH]:[c](:[*]):[*]	-0.251
ECFP_6	642810091	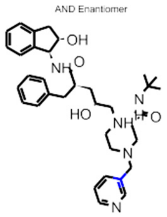 [*][c](:[*]):[*]	-0.247

ECFP_6	-182236392	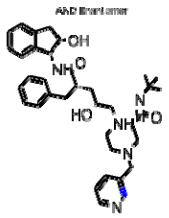 [*]:[cH]:[*]	-

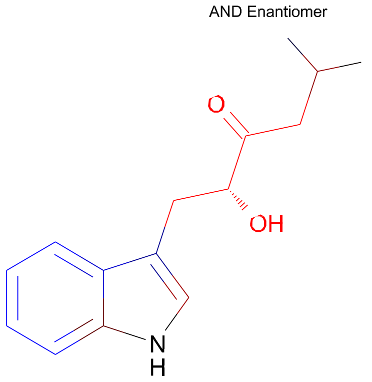

$C_{15}H_{19}NO_2$
Molecular Weight: 245.31685
ALogP: 2.932
Rotatable Bonds: 5
Acceptors: 2
Donors: 2

Structural Similar Compounds

Name	Cinnamyl anthranilate	171	2,5-Dimethoxy-4´-aminostilbene
Structure	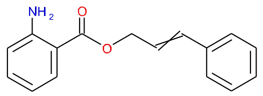	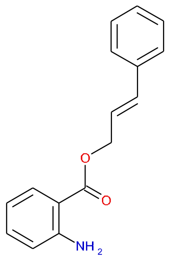	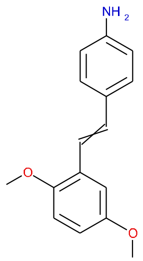
Actual Endpoint (-log C)	1.32084	1.32084	5.54914
Predicted Endpoint (-log C)	2.6022	2.6022	4.63197
Distance	0.577	0.577	0.602
Reference	CPDB	CPDB	CPDB

Model Applicability

Unknown features are fingerprint features in the query molecule, but not found in the training set.

1. All properties and OPS components are within expected ranges.

Model Prediction

Prediction: 0.910
Unit: mg/kg_body_weight/day
Mahalanobis Distance: 13.799
Mahalanobis Distance p-value: 6.78e-008

Mahalanobis Distance: The Mahalanobis distance (MD) is a generalization of the Euclidean distance that accounts for correlations among the X properties. It is calculated as the distance to the center of the training data. The larger the MD, the less trustworthy the prediction.
Mahalanobis Distance p-value: The p-value gives the fraction of training data with an MD greater than or equal to the one for the given sample, assuming normally distributed data. The smaller the p-value, the less trustworthy the prediciton. For highly non-normal X properties (e.g., fingerprints), the MD p-value is wildly inaccurate.

Feature Contribution

Top features for positive contribution

Fingerprint	Bit/Smiles	Feature Structure	Score
FCFP_6	-1043250487	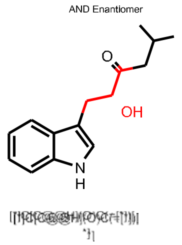	1.153

FCFP_6	565968762	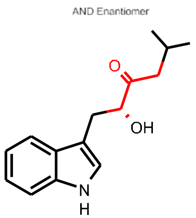 [*]CC(=O)C([*])[*]	0.266
FCFP_6	1	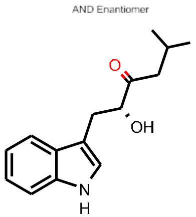 [*]=O	0.234
**Top Features for negative contribution**			
**Fingerprint**	**Bit/Smiles**	**Feature Structure**	**Score**
FCFP_6	991735244	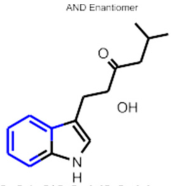 [*]:[c]1:[*]:[cH]:[cH]:[cH]:[cH]:1	-0.422
FCFP_6	16	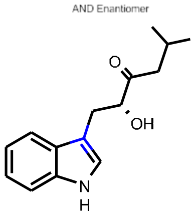 [*][c](:[*]):[*]	-0.354
FCFP_6	0	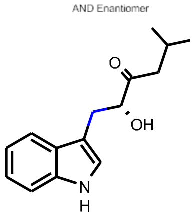 [*]C[*]	-0.115

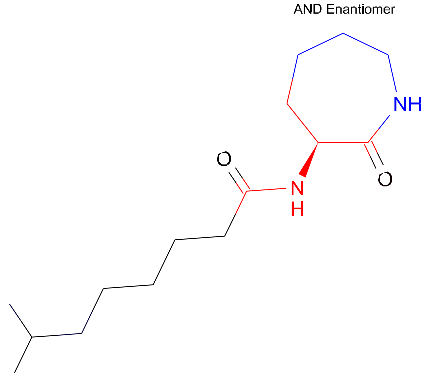

$C_{15}H_{28}N_2O_2$
Molecular Weight: 268.39501
ALogP: 2.685
Rotatable Bonds: 7
Acceptors: 2
Donors: 2

Model Prediction

Prediction: 16.647
Unit: mg/kg_body_weight/day
Mahalanobis Distance: 11.911
Mahalanobis Distance p-value: 0.000769

Mahalanobis Distance: The Mahalanobis distance (MD) is a generalization of the Euclidean distance that accounts for correlations among the X properties. It is calculated as the distance to the center of the training data. The larger the MD, the less trustworthy the prediction.
Mahalanobis Distance p-value: The p-value gives the fraction of training data with an MD greater than or equal to the one for the given sample, assuming normally distributed data. The smaller the p-value, the less trustworthy the prediciton. For highly non-normal X properties (e.g., fingerprints), the MD p-value is wildly inaccurate.

Structural Similar Compounds

Name	1,3-Dibutyl-1-nitrosourea	381	11-Aminoundecanoic acid
Structure	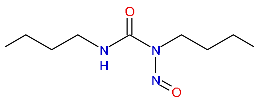	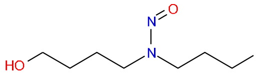	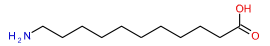
Actual Endpoint (-log C)	4.67233	5.58123	2.26246
Predicted Endpoint (-log C)	4.84898	4.70679	2.18375
Distance	0.610	0.671	0.676
Reference	CPDB	CPDB	CPDB

Model Applicability

Unknown features are fingerprint features in the query molecule, but not found in the training set.

1. All properties and OPS components are within expected ranges.

Feature Contribution

Top features for positive contribution

Fingerprint	Bit/Smiles	Feature Structure	Score
FCFP_6	-1043250487	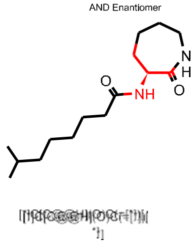	1.153

FCFP_6	1	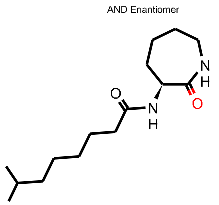 [*]=O	0.234
FCFP_6	-885550502	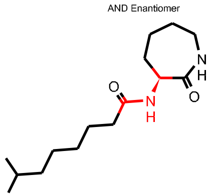 [*]C([*])NC(=[*])[*]	0.229

Top Features for negative contribution

Fingerprint	Bit/Smiles	Feature Structure	Score
FCFP_6	-1272709286	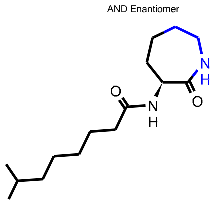 [*]CCN[*]	-0.526
FCFP_6	1175638033	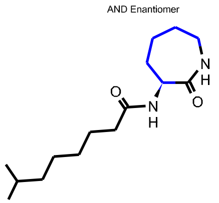 [*][C@@H]1[*][*]CCCC1	-0.512
FCFP_6	566058135	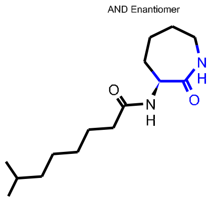 [*]NC(=O)C([*])[*]	-0.182

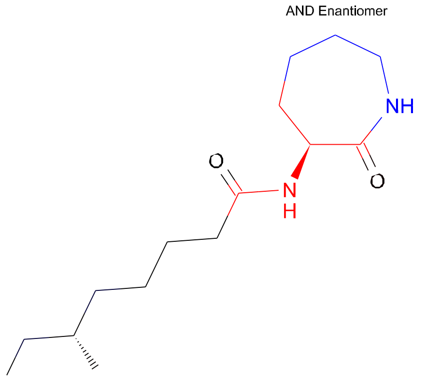

$C_{15}H_{28}N_2O_2$
Molecular Weight: 268.39501
ALogP: 2.685
Rotatable Bonds: 7
Acceptors: 2
Donors: 2

Model Prediction

Prediction: 16.647
Unit: mg/kg_body_weight/day
Mahalanobis Distance: 11.911
Mahalanobis Distance p-value: 0.000769

Mahalanobis Distance: The Mahalanobis distance (MD) is a generalization of the Euclidean distance that accounts for correlations among the X properties. It is calculated as the distance to the center of the training data. The larger the MD, the less trustworthy the prediction.
Mahalanobis Distance p-value: The p-value gives the fraction of training data with an MD greater than or equal to the one for the given sample, assuming normally distributed data. The smaller the p-value, the less trustworthy the prediciton. For highly non-normal X properties (e.g., fingerprints), the MD p-value is wildly inaccurate.

Structural Similar Compounds

Name	1,3-Dibutyl-1-nitrosourea	381	N-Hexylnitrosourea
Structure	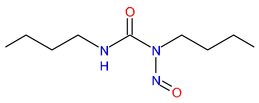	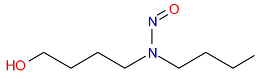	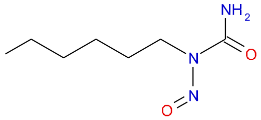
Actual Endpoint (-log C)	4.67233	5.58123	5.52846
Predicted Endpoint (-log C)	4.84898	4.70679	4.45937
Distance	0.611	0.672	0.677
Reference	CPDB	CPDB	CPDB

Model Applicability

Unknown features are fingerprint features in the query molecule, but not found in the training set.

1. All properties and OPS components are within expected ranges.

Feature Contribution

Top features for positive contribution

Fingerprint	Bit/Smiles	Feature Structure	Score
FCFP_6	-1043250487	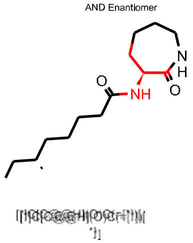 AND Enantiomer	1.153

FCFP_6	1	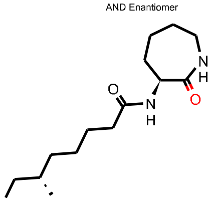<br>[*]=O	0.234
FCFP_6	-885550502	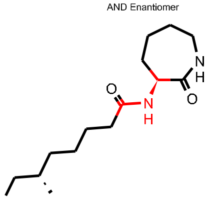<br>[*]C([*])NC(=[*])[*]	0.229

Top Features for negative contribution

Fingerprint	Bit/Smiles	Feature Structure	Score
FCFP_6	-1272709286	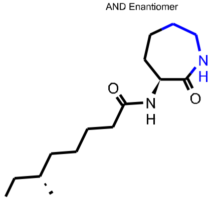<br>[*]CCN[*]	-0.526
FCFP_6	1175638033	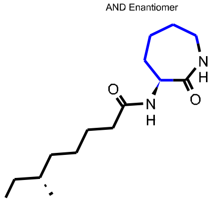<br>[*][C@@H]1[*][*]CCCC1	-0.512
FCFP_6	566058135	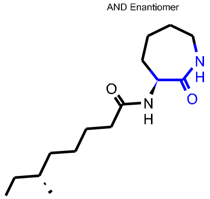<br>[*]NC(=O)C([*])[*]	-0.182

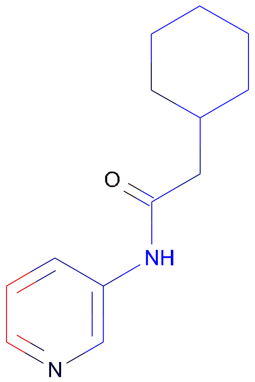

$C_{13}H_{18}N_2O$
Molecular Weight: 218.29481
ALogP: 2.171
Rotatable Bonds: 3
Acceptors: 2
Donors: 1

Model Prediction

Prediction: 200.907
Unit: mg/kg_body_weight/day
Mahalanobis Distance: 12.724
Mahalanobis Distance p-value: 2.11e-005

Mahalanobis Distance: The Mahalanobis distance (MD) is a generalization of the Euclidean distance that accounts for correlations among the X properties. It is calculated as the distance to the center of the training data. The larger the MD, the less trustworthy the prediction.
Mahalanobis Distance p-value: The p-value gives the fraction of training data with an MD greater than or equal to the one for the given sample, assuming normally distributed data. The smaller the p-value, the less trustworthy the prediciton. For highly non-normal X properties (e.g., fingerprints), the MD p-value is wildly inaccurate.

Structural Similar Compounds

Name	Phenacetin	Nitrosoanabasine	526
Structure	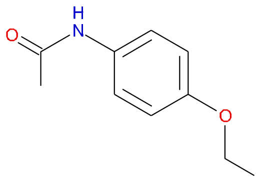	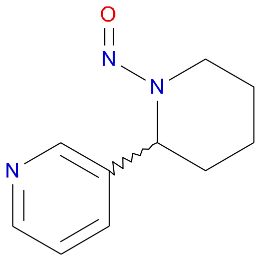	AND Enantiomer 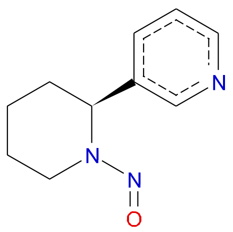
Actual Endpoint (-log C)	2.15647	4.20601	4.20601
Predicted Endpoint (-log C)	2.91803	4.92308	4.92308
Distance	0.528	0.550	0.550
Reference	CPDB	CPDB	CPDB

Model Applicability

Unknown features are fingerprint features in the query molecule, but not found in the training set.

1. All properties and OPS components are within expected ranges.

Feature Contribution

Top features for positive contribution

Fingerprint	Bit/Smiles	Feature Structure	Score
FCFP_6	1	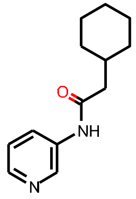 [*]=O	0.234

FCFP_6	730557100	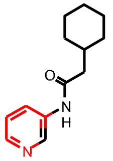 [*][c]1:[*]:n:[cH]:[cH]:[cH]:1	0.141
FCFP_6	-1272798659	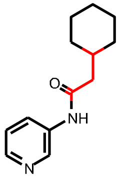 [*]C([*])CC(=[*])[*]	0.110
**Top Features for negative contribution**			
**Fingerprint**	**Bit/Smiles**	**Feature Structure**	**Score**
FCFP_6	1175638033	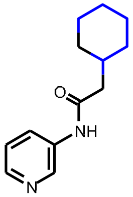 [*][C@@H]1[*][*]CCCC1	-0.512
FCFP_6	16	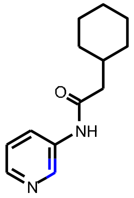 [*][c](:[*]):[*]	-0.354
FCFP_6	590925877	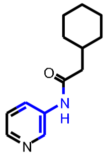 [*]N[c](:[cH]:[*]):[cH]:[*]	-0.323

Indinavir

TOPKAT_Carcinogenic_Potency_TD50_Rat

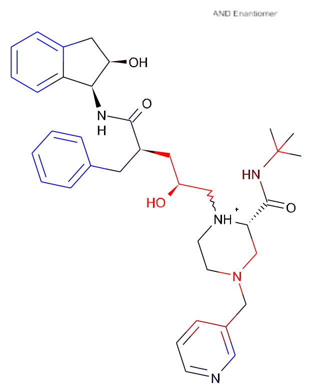

$C_{36}H_{48}N_5O_4$

Molecular Weight: 614.79741

ALogP: 1.521

Rotatable Bonds: 12

Acceptors: 6

Donors: 5

Model Prediction

Prediction: 0.142

Unit: mg/kg_body_weight/day

Mahalanobis Distance: 17.025

Mahalanobis Distance p-value: 9.66e-018

Mahalanobis Distance: The Mahalanobis distance (MD) is a generalization of the Euclidean distance that accounts for correlations among the X properties. It is calculated as the distance to the center of the training data. The larger the MD, the less trustworthy the prediction.
Mahalanobis Distance p-value: The p-value gives the fraction of training data with an MD greater than or equal to the one for the given sample, assuming normally distributed data. The smaller the p-value, the less trustworthy the prediciton. For highly non-normal X properties (e.g., fingerprints), the MD p-value is wildly inaccurate.

Structural Similar Compounds

Name	551	Tamoxifen citrate	Fluvastatin
Structure	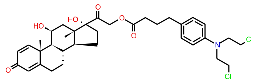	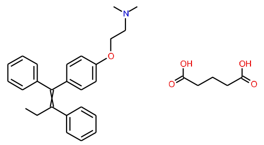	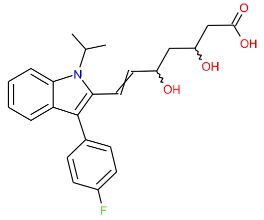
Actual Endpoint (-log C)	4.52736	5.10442	3.51742
Predicted Endpoint (-log C)	6.81053	3.58059	5.41573
Distance	0.981	0.989	1.009
Reference	CPDB	CPDB	CPDB

Model Applicability

Unknown features are fingerprint features in the query molecule, but not found in the training set.

1. OPS PC6 out of range. Value: 7.7579. Training min, max, SD, explained variance: -5.5832, 6.4847, 1.973, 0.0374.

Feature Contribution

Top features for positive contribution

Fingerprint	Bit/Smiles	Feature Structure	Score
FCFP_6	-1043250487	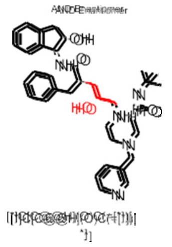	1.153

FCFP_6	9	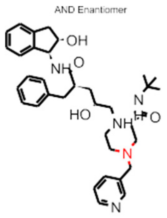 [*]N([*])[*]	0.385
FCFP_6	-587569116	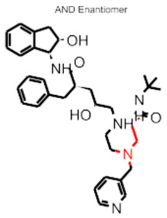 [*]C([*])CN([*])[*]	0.319

Top Features for negative contribution

**Fingerprint**	**Bit/Smiles**	**Feature Structure**	**Score**
FCFP_6	991735244	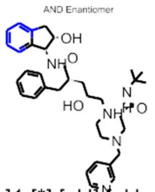 [*]:[c]1:[*]:[cH]:[cH]:[cH]:[cH]:1	-0.422
FCFP_6	-2093839777	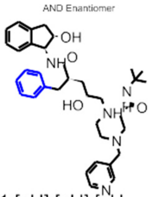 [*][c]1:[cH]:[cH]:[cH]:[cH]:[cH]:1	-0.378
FCFP_6	16	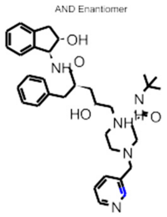 [*][c](:[*]):[*]	-0.354

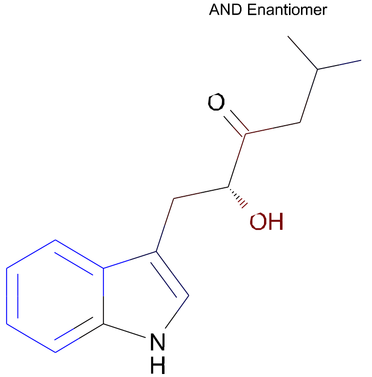

$C_{15}H_{19}NO_2$
Molecular Weight: 245.31685
ALogP: 2.932
Rotatable Bonds: 5
Acceptors: 2
Donors: 2

Structural Similar Compounds

Name	ETODOLAC	PINDOLOL	DICHLOFENAC.NA
Structure	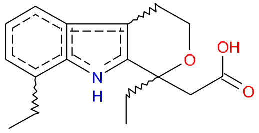	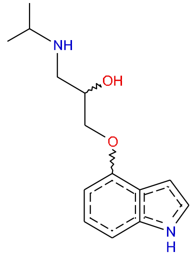	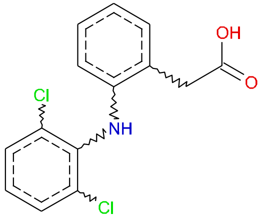
Actual Endpoint (-log C)	4.9813	3.4038	5.47151
Predicted Endpoint (-log C)	4.39289	4.03315	3.9421
Distance	0.483	0.501	0.549
Reference	NDA-18922	NDA-18285	NDA-19201

Model Prediction

Prediction: 0.081
Unit: g/kg_body_weight
Mahalanobis Distance: 28.400
Mahalanobis Distance p-value: 9.65e-022

Mahalanobis Distance: The Mahalanobis distance (MD) is a generalization of the Euclidean distance that accounts for correlations among the X properties. It is calculated as the distance to the center of the training data. The larger the MD, the less trustworthy the prediction.
Mahalanobis Distance p-value: The p-value gives the fraction of training data with an MD greater than or equal to the one for the given sample, assuming normally distributed data. The smaller the p-value, the less trustworthy the prediciton. For highly non-normal X properties (e.g., fingerprints), the MD p-value is wildly inaccurate.

Model Applicability

Unknown features are fingerprint features in the query molecule, but not found in the training set.

1. All properties and OPS components are within expected ranges.
2. Unknown ECFP_6 feature: -152683720: [*]:[nH]:[*]
3. Unknown ECFP_6 feature: 2023785560: [*]C([*])O
4. Unknown ECFP_6 feature: -953984246: [*]:[c]1:[*]:[*]:[cH]:[nH]:1
5. Unknown ECFP_6 feature: 770157610: [*]C([*])C[c](:[*]):[*]
6. Unknown ECFP_6 feature: 80433051: [*]C[C@@H](O)C(=[*])[*]
7. Unknown ECFP_6 feature: 1035165602: [*]CC(C)C
8. Unknown ECFP_6 feature: -1310859884: [*]C([*])CC(=[*])[*]
9. Unknown ECFP_6 feature: -2024509555: [*]C[c]1:[cH]:[*]:[*]:[c]:1:[*]
10. Unknown ECFP_6 feature: 1732075620: [*]CC(=O)C([*])[*]
11. Unknown ECFP_6 feature: 1333660716: [*][c]1:[*]:[*]:[c](:[*]):[c]:1:[cH]:[*]
12. Unknown ECFP_6 feature: 1099224616: [*]:[cH]:[c]1:[nH]:[*]:[*]:[c]:1:[*]
13. Unknown ECFP_6 feature: -1020449580: [*][c]1:[*]:[*]:[nH]:[cH]:1
14. Unknown ECFP_6 feature: 1997021792: [*]:[cH]:[cH]:[cH]:[*]

Feature Contribution

Top features for positive contribution			
Fingerprint	Bit/Smiles	Feature Structure	Score

ECFP_6	1559650422	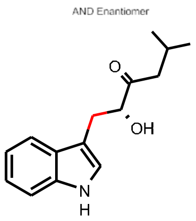<br>[*]C[*]	0.129
FCFP_6	3	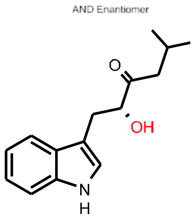<br>[*]O	0.092
ECFP_6	2099970318	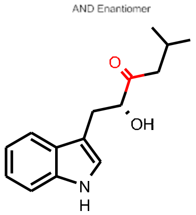<br>[*]C(=O)[*]	0.077

Top Features for negative contribution

Fingerprint	Bit/Smiles	Feature Structure	Score
FCFP_6	991735244	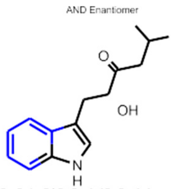<br>[*]:[c]1:[*]:[cH]:[cH]:[cH]:[cH]:1	-0.134
ECFP_6	1564392544	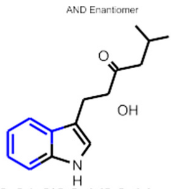<br>[*]:[c]1:[*]:[cH]:[cH]:[cH]:[cH]:1	-0.133

FCFP_6	1	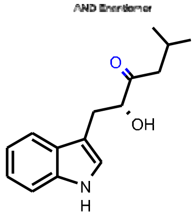<br>[*]=O	-

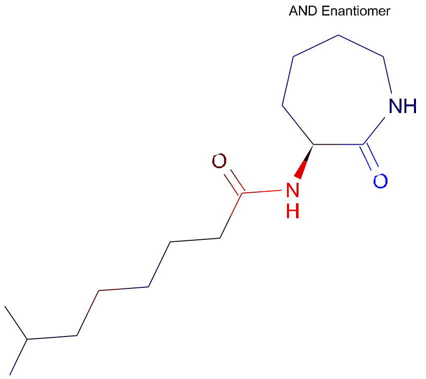

$C_{15}H_{28}N_2O_2$
Molecular Weight: 268.39501
ALogP: 2.685
Rotatable Bonds: 7
Acceptors: 2
Donors: 2

Structural Similar Compounds

Name	N;N'-DICYCLOHEXYLTHIOUREA	METOCLOPRAMIDE	11-AMINOUNDECANOIC ACID
Structure	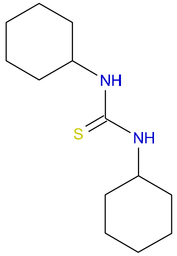	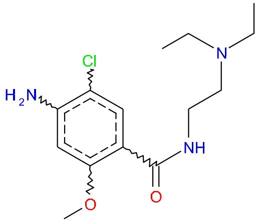	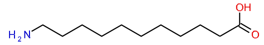
Actual Endpoint (-log C)	1.98301	4.47683	2.72983
Predicted Endpoint (-log C)	3.65588	3.8785	2.871
Distance	0.618	0.634	0.686
Reference	NTP 56 41	NDA-17854	FUND.APPL.TOXICOL.3.6 14.1983

Model Prediction

Prediction: 0.541
Unit: g/kg_body_weight
Mahalanobis Distance: 27.037
Mahalanobis Distance p-value: 2.98e-019

Mahalanobis Distance: The Mahalanobis distance (MD) is a generalization of the Euclidean distance that accounts for correlations among the X properties. It is calculated as the distance to the center of the training data. The larger the MD, the less trustworthy the prediction.
Mahalanobis Distance p-value: The p-value gives the fraction of training data with an MD greater than or equal to the one for the given sample, assuming normally distributed data. The smaller the p-value, the less trustworthy the prediciton. For highly non-normal X properties (e.g., fingerprints), the MD p-value is wildly inaccurate.

Model Applicability

Unknown features are fingerprint features in the query molecule, but not found in the training set.

1. All properties and OPS components are within expected ranges.
2. Unknown ECFP_6 feature: -154530762: [*]N[*]
3. Unknown ECFP_6 feature: -2091181441: [*]C([*])NC(=[*])[*]
4. Unknown ECFP_6 feature: -1694930393: [*]CNC(=[*])[*]
5. Unknown ECFP_6 feature: -2097159651: [*]C[C@H](N[*])C(=[*])[*]
6. Unknown ECFP_6 feature: -1332781180: [*]CCC[*]
7. Unknown ECFP_6 feature: -1790802833: [*]CCC([*])[*]
8. Unknown ECFP_6 feature: -1331920947: [*]CCN[*]
9. Unknown ECFP_6 feature: 1035165602: [*]CC(C)C
10. Unknown ECFP_6 feature: -867777309: [*]NC(=O)C([*])[*]
11. Unknown ECFP_6 feature: -1789102870: [*]CCC(=[*])[*]
12. Unknown ECFP_6 feature: 1731843802: [*]CC(=O)N[*]

Feature Contribution

Top features for positive contribution			
Fingerprint	Bit/Smiles	Feature Structure	Score

ECFP_6	-167460056	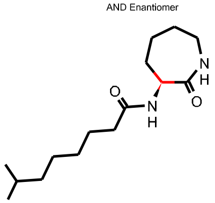 [*]C([*])[*]	0.136
ECFP_6	1559650422	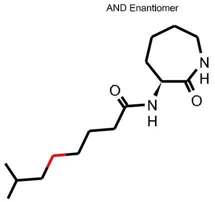 [*]C[*]	0.129
FCFP_6	3	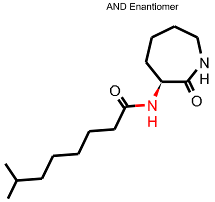 [*]O	0.092

Top Features for negative contribution

Fingerprint	Bit/Smiles	Feature Structure	Score
ECFP_6	2106656448	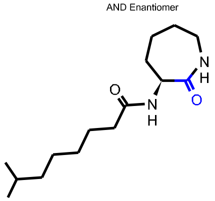 [*]C(=O)[*]	-0.110
FCFP_6	1	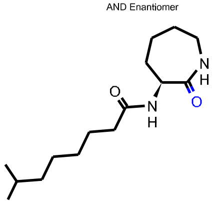 [*]=O	-0.102

FCFP_6	13659732	AND Enantiomer 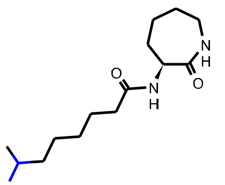 [*]C([*])C	-

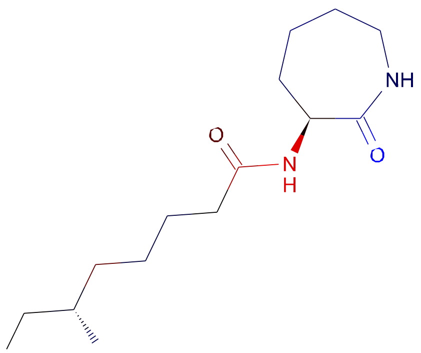

$C_{15}H_{28}N_2O_2$
Molecular Weight: 268.39501
ALogP: 2.685
Rotatable Bonds: 7
Acceptors: 2
Donors: 2

Structural Similar Compounds

Name	N;N'-DICYCLOHEXYLTHIOUREA	METOCLOPRAMIDE	CARBROMAL
Structure	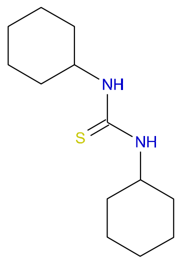	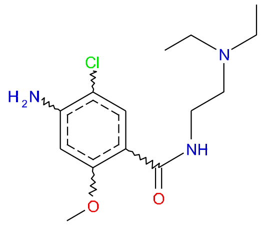	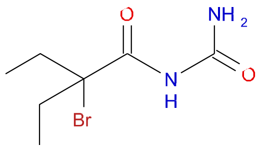
Actual Endpoint (-log C)	1.98301	4.47683	3.27801
Predicted Endpoint (-log C)	3.65588	3.8785	3.98319
Distance	0.624	0.629	0.687
Reference	NTP 56 41	NDA-17854	NTP 173 C-3

Model Prediction

Prediction: 0.456
Unit: g/kg_body_weight
Mahalanobis Distance: 28.352
Mahalanobis Distance p-value: 1.18e-021

Mahalanobis Distance: The Mahalanobis distance (MD) is a generalization of the Euclidean distance that accounts for correlations among the X properties. It is calculated as the distance to the center of the training data. The larger the MD, the less trustworthy the prediction.
Mahalanobis Distance p-value: The p-value gives the fraction of training data with an MD greater than or equal to the one for the given sample, assuming normally distributed data. The smaller the p-value, the less trustworthy the prediciton. For highly non-normal X properties (e.g., fingerprints), the MD p-value is wildly inaccurate.

Model Applicability

Unknown features are fingerprint features in the query molecule, but not found in the training set.

1. All properties and OPS components are within expected ranges.
2. Unknown ECFP_6 feature: -154530762: [*]N[*]
3. Unknown ECFP_6 feature: -2091181441: [*]C([*])NC(=[*])[*]
4. Unknown ECFP_6 feature: -1694930393: [*]CNC(=[*])[*]
5. Unknown ECFP_6 feature: -2097159651: [*]C[C@H](N[*])C(=[*])[*]
6. Unknown ECFP_6 feature: 194354829: [*]CC(C)C[*]
7. Unknown ECFP_6 feature: -1790802833: [*]CCC([*])[*]
8. Unknown ECFP_6 feature: -1332781180: [*]CCC[*]
9. Unknown ECFP_6 feature: -949992060: [*]C([*])CC
10. Unknown ECFP_6 feature: -1331920947: [*]CCN[*]
11. Unknown ECFP_6 feature: -1789102870: [*]CCC(=[*])[*]
12. Unknown ECFP_6 feature: -867777309: [*]NC(=O)C([*])[*]
13. Unknown ECFP_6 feature: 1731843802: [*]CC(=O)N[*]

Feature Contribution

Top features for positive contribution			
**Fingerprint**	**Bit/Smiles**	**Feature Structure**	**Score**

ECFP_6	-167460056	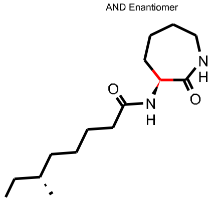 [*]C([*])[*]	0.136
ECFP_6	1559650422	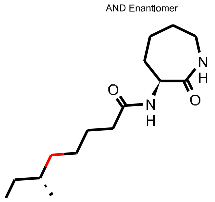 [*]C[*]	0.129
FCFP_6	3	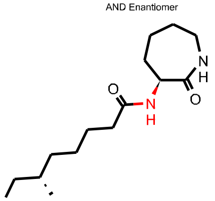 [*]O	0.092

Top Features for negative contribution

**Fingerprint**	**Bit/Smiles**	**Feature Structure**	**Score**
ECFP_6	2106656448	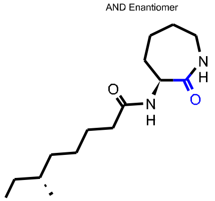 [*]C(=O)[*]	-0.110
FCFP_6	1	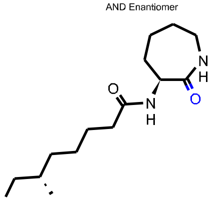 [*]=O	-0.102

FCFP_6	13659732	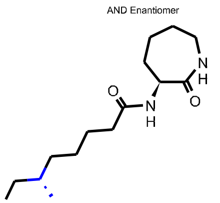 [*]C([*])C	-

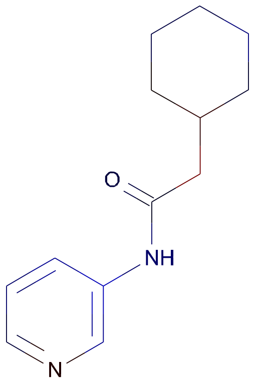

$C_{13}H_{18}N_2O$
Molecular Weight: 218.29481
ALogP: 2.171
Rotatable Bonds: 3
Acceptors: 2
Donors: 1

Structural Similar Compounds

Name	CHLORPROPHAM	LINURON	CARBOXIN
Structure	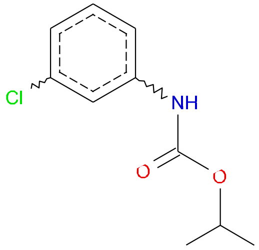	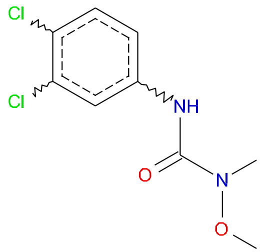	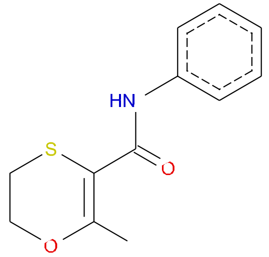
Actual Endpoint (-log C)	3.15363	3.90151	3.8945
Predicted Endpoint (-log C)	3.82083	3.96609	3.7395
Distance	0.479	0.484	0.504
Reference	COVER SHEET 0283;891101;(1)	HEEP ECAO CIN P015;8401;(2)	EPA COVER SHEET 0022;890701;(1)

Model Prediction

Prediction: 0.054
Unit: g/kg_body_weight
Mahalanobis Distance: 24.905
Mahalanobis Distance p-value: 2.15e-015

Mahalanobis Distance: The Mahalanobis distance (MD) is a generalization of the Euclidean distance that accounts for correlations among the X properties. It is calculated as the distance to the center of the training data. The larger the MD, the less trustworthy the prediction.
Mahalanobis Distance p-value: The p-value gives the fraction of training data with an MD greater than or equal to the one for the given sample, assuming normally distributed data. The smaller the p-value, the less trustworthy the prediciton. For highly non-normal X properties (e.g., fingerprints), the MD p-value is wildly inaccurate.

Model Applicability

Unknown features are fingerprint features in the query molecule, but not found in the training set.

1. All properties and OPS components are within expected ranges.
2. Unknown ECFP_6 feature: 1731843802: [*]CC(=O)N[*]
3. Unknown ECFP_6 feature: -82840383: [*]C([*])CC(=[*])[*]
4. Unknown ECFP_6 feature: -709633021: [*][c](:[*]):[cH]:n:[*]
5. Unknown ECFP_6 feature: -177077903: [*]N[c](:[cH]:[*]):[cH]:[*]
6. Unknown ECFP_6 feature: 1997021792: [*]:[cH]:[cH]:[cH]:[*]
7. Unknown ECFP_6 feature: 1996163143: [*]:[cH]:[cH]:n:[*]
8. Unknown ECFP_6 feature: -677055651: [*]:[cH]:n:[cH]:[*]
9. Unknown ECFP_6 feature: -1795620553: [*]CC(C[*])C[*]
10. Unknown ECFP_6 feature: -1332781180: [*]CCC[*]

Feature Contribution

Top features for positive contribution			
Fingerprint	Bit/Smiles	Feature Structure	Score

ECFP_6	-167460056	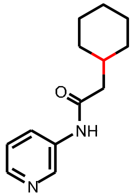 [*]C([*])[*]	0.136
ECFP_6	1559650422	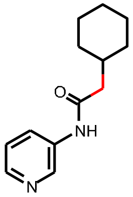 [*]C[*]	0.129
FCFP_6	3	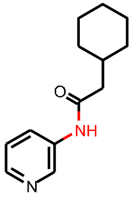 [*]O	0.092
**Top Features for negative contribution**			
**Fingerprint**	**Bit/Smiles**	**Feature Structure**	**Score**
FCFP_6	1	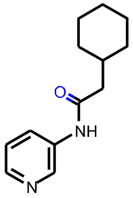 [*]=O	-0.102
ECFP_6	-1236483485	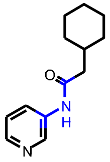 [*]C(=[*])N[c](:[*]):[*]	-0.075

FCFP_6	117563803	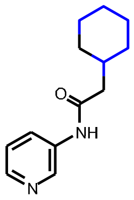 [*][C@@H]1[*][*]CCCC1	-

Indinavir

TOPKAT_Chronic_LOAEL

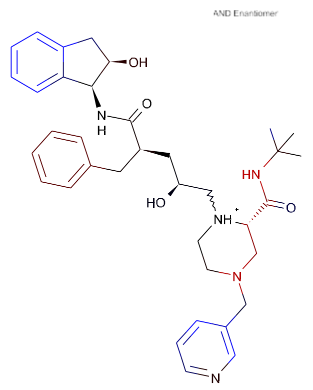

$C_{36}H_{48}N_5O_4$
Molecular Weight: 614.79741
ALogP: 1.521
Rotatable Bonds: 12
Acceptors: 6
Donors: 5

Structural Similar Compounds

Name	GLYBURIDE	GLIPIZIDE	RESERPINE
Structure	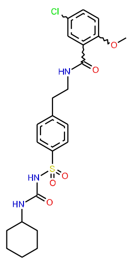	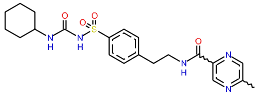	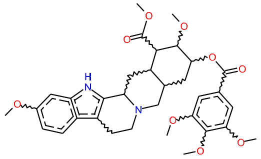
Actual Endpoint (-log C)	4.21661	3.94991	6.38645
Predicted Endpoint (-log C)	4.21035	3.95594	5.548
Distance	0.914	0.922	1.006
Reference	UPJ-26452	NDA-17583	NTP 193 22

Model Prediction

Prediction: 0.008
Unit: g/kg_body_weight
Mahalanobis Distance: 38.381
Mahalanobis Distance p-value: 1.86e-039

Mahalanobis Distance: The Mahalanobis distance (MD) is a generalization of the Euclidean distance that accounts for correlations among the X properties. It is calculated as the distance to the center of the training data. The larger the MD, the less trustworthy the prediction.
Mahalanobis Distance p-value: The p-value gives the fraction of training data with an MD greater than or equal to the one for the given sample, assuming normally distributed data. The smaller the p-value, the less trustworthy the prediciton. For highly non-normal X properties (e.g., fingerprints), the MD p-value is wildly inaccurate.

Model Applicability

Unknown features are fingerprint features in the query molecule, but not found in the training set.

1. All properties and OPS components are within expected ranges.
2. Unknown FCFP_2 feature: 10: [*][NH+]([*])[*]
3. Unknown FCFP_2 feature: -1853714334: [*]C[NH+](C[*])C([*])[*]
4. Unknown FCFP_2 feature: -1817836174: [*]C[C@H]([NH+]([*])[*])C(=[*])[*]
5. Unknown FCFP_2 feature: 1155241219: [*]CC[NH+]([*])[*]
6. Unknown ECFP_6 feature: 1976330679: [*][NH+]([*])[*]
7. Unknown ECFP_6 feature: 1134829831: [*]C[NH+](C[*])C([*])[*]
8. Unknown ECFP_6 feature: -1924540582: [*]C[C@H]([NH+]([*])[*])C(=[*])[*]
9. Unknown ECFP_6 feature: -756348342: [*]C([*])CN([*])[*]
10. Unknown ECFP_6 feature: -2041399277: [*]CN(C[*])C[*]
11. Unknown ECFP_6 feature: -757679000: [*]CCN([*])[*]
12. Unknown ECFP_6 feature: -244159614: [*]CC[NH+]([*])[*]
13. Unknown ECFP_6 feature: -81134287: [*]NC(=O)C([*])[*]
14. Unknown ECFP_6 feature: 16744893: [*]C(=[*])NC([*])([*])[*]
15. Unknown ECFP_6 feature: -44121127: [*]N([*])C[c](:[*]):[*]
16. Unknown ECFP_6 feature: -709633021: [*][c](:[*]):[cH]:n:[*]
17. Unknown ECFP_6 feature: 1997021792: [*]:[cH]:[cH]:[cH]:[*]
18. Unknown ECFP_6 feature: 1996163143: [*]:[cH]:[cH]:n:[*]
19. Unknown ECFP_6 feature: -677055651: [*]:[cH]:n:[cH]:[*]
20. Unknown ECFP_6 feature: -1623733623: [*]NC(C)(C)C
21. Unknown ECFP_6 feature: 865857320: [*]C([*])([*])C

22. Unknown ECFP_6 feature: 474121058: [*]C([*])C[NH+]([*])[*]
23. Unknown ECFP_6 feature: 196083830: [*]CC(O)C[*]
24. Unknown ECFP_6 feature: -1312559847: [*]C([*])CC([*])[*]
25. Unknown ECFP_6 feature: 198723869: [*]CC(C[*])C(=[*])[*]
26. Unknown ECFP_6 feature: -649348348: [*]NC(=O)C([*])[*]
27. Unknown ECFP_6 feature: -2091181441: [*]C([*])NC(=[*])[*]
28. Unknown ECFP_6 feature: -1567199489: [*]N[C@@H]1[C@H]([*])[*][*]:[c]1:[*]
29. Unknown ECFP_6 feature: -329826665: [*][C@H]1[*]:[*]C[C@H]1O
30. Unknown ECFP_6 feature: 53207596: [*][C@H]1[*][*]:[c](:[*])C1
31. Unknown ECFP_6 feature: 1334973442: [*]:[cH]:[c]1C[*][*][c]:1:[*]
32. Unknown ECFP_6 feature: 1336304100: [*][C@@H]1[*][*][c](:[*]):[c]1:[cH]:[*]
33. Unknown ECFP_6 feature: 2024749573: [*]C([*])O
34. Unknown ECFP_6 feature: 2023785560: [*]C([*])O
35. Unknown ECFP_6 feature: 770157610: [*]C([*])C[c](:[*]):[*]

Feature Contribution

Top features for positive contribution

Fingerprint	Bit/Smiles	Feature Structure	Score
ECFP_6	-167460056	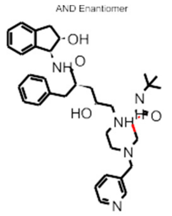 [*]C([*])[*]	0.136
ECFP_6	1559650422	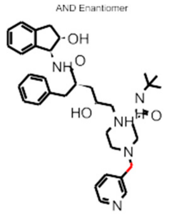 [*]C[*]	0.129

FCFP_6	3	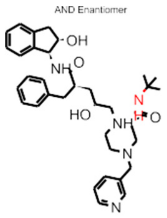 [*]O	0.092
**Top Features for negative contribution**			
**Fingerprint**	**Bit/Smiles**	**Feature Structure**	**Score**
FCFP_6	991735244	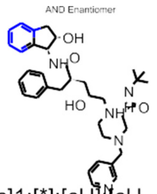 [*]:[c]1:[*]:[cH]:[cH]:[cH]:[cH]:1	-0.134
ECFP_6	1564392544	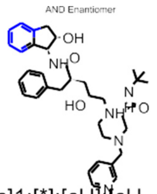 [*]:[c]1:[*]:[cH]:[cH]:[cH]:[cH]:1	-0.133
FCFP_6	1	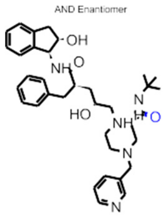 [*]=O	-0.102

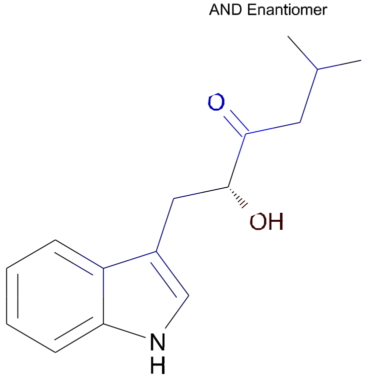

$C_{15}H_{19}NO_2$
Molecular Weight: 245.31685
ALogP: 2.932
Rotatable Bonds: 5
Acceptors: 2
Donors: 2

Structural Similar Compounds

Name	BENZOIN	5,5-DIPHENYLHYDANTOIN	CINNAMYL ANTHRANILATE
Structure	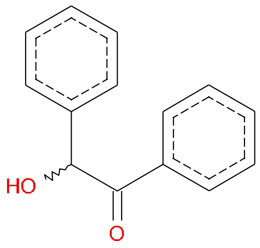	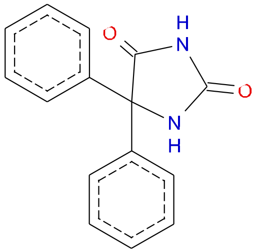	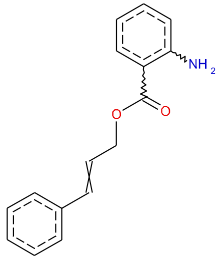
Actual Endpoint (-log C)	4.27569	3.36845	2.2733
Predicted Endpoint (-log C)	3.12678	3.3497	3.25473
Distance	0.523	0.531	0.533
Reference	NCI/NTP TR-204	NCI/NTP TR-404	NCI/NTP TR-196

Model Prediction

Prediction: 0.346
Unit: g/kg_body_weight
Mahalanobis Distance: 7.619
Mahalanobis Distance p-value: 0.0596

Mahalanobis Distance: The Mahalanobis distance (MD) is a generalization of the Euclidean distance that accounts for correlations among the X properties. It is calculated as the distance to the center of the training data. The larger the MD, the less trustworthy the prediction.
Mahalanobis Distance p-value: The p-value gives the fraction of training data with an MD greater than or equal to the one for the given sample, assuming normally distributed data. The smaller the p-value, the less trustworthy the prediciton. For highly non-normal X properties (e.g., fingerprints), the MD p-value is wildly inaccurate.

Model Applicability

Unknown features are fingerprint features in the query molecule, but not found in the training set.

1. All properties and OPS components are within expected ranges.

Feature Contribution

Top features for positive contribution

Fingerprint	Bit/Smiles	Feature Structure	Score
FCFP_2	3	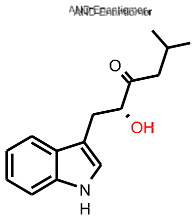 [*]O	0.074

Top Features for negative contribution

Fingerprint	Bit/Smiles	Feature Structure	Score

FCFP_2	-1272798659	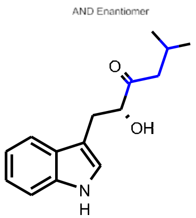<br>[*]C([*])CC(=[*])[*]	-0.111
FCFP_2	1872154524	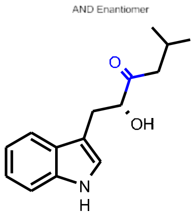<br>[*]C(=O)[*]	-0.105
FCFP_2	203677720	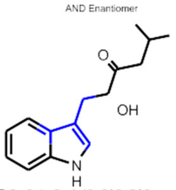<br>[*]C[c]1:[cH]:[*]:[*]<br>:[c]:1:[*]	-0.083

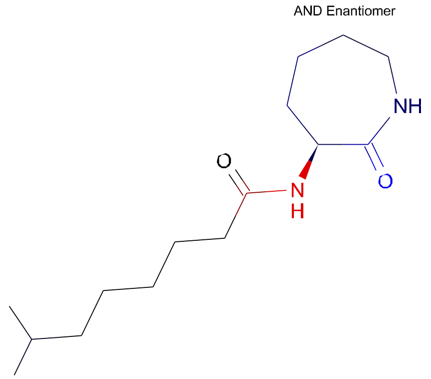

$C_{15}H_{28}N_2O_2$
Molecular Weight: 268.39501
ALogP: 2.685
Rotatable Bonds: 7
Acceptors: 2
Donors: 2

Model Prediction

Prediction: 0.126
Unit: g/kg_body_weight
Mahalanobis Distance: 7.845
Mahalanobis Distance p-value: 0.0356

Mahalanobis Distance: The Mahalanobis distance (MD) is a generalization of the Euclidean distance that accounts for correlations among the X properties. It is calculated as the distance to the center of the training data. The larger the MD, the less trustworthy the prediction.
Mahalanobis Distance p-value: The p-value gives the fraction of training data with an MD greater than or equal to the one for the given sample, assuming normally distributed data. The smaller the p-value, the less trustworthy the prediciton. For highly non-normal X properties (e.g., fingerprints), the MD p-value is wildly inaccurate.

Structural Similar Compounds

Name	N,N'-DIETHYLTHIOUREA	CARBROMAL	TOLBUTAMIDE
Structure	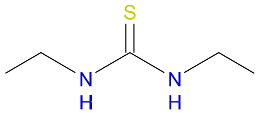	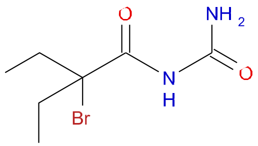	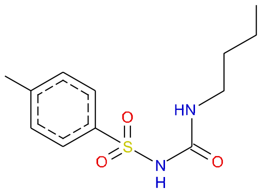
Actual Endpoint (-log C)	4.07016	3.32377	2.3985
Predicted Endpoint (-log C)	3.12792	3.55919	3.32272
Distance	0.525	0.529	0.560
Reference	NCI/NTP TR-149	NCI/NTP TR-173	NCI/NTP TR-031

Model Applicability

Unknown features are fingerprint features in the query molecule, but not found in the training set.

1. OPS PC12 out of range. Value: -2.8089. Training min, max, SD, explained variance: -2.364, 2.9228, 1.079, 0.0263.

Feature Contribution

Top features for positive contribution

Fingerprint	Bit/Smiles	Feature Structure	Score
FCFP_2	-885550502	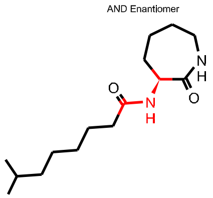 [*]C([*])NC(=[*])[*]	0.115

FCFP_2	3	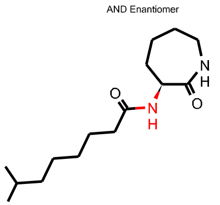 [*]O	0.074
**Top Features for negative contribution**			
**Fingerprint**	**Bit/Smiles**	**Feature Structure**	**Score**
FCFP_2	-1272798659	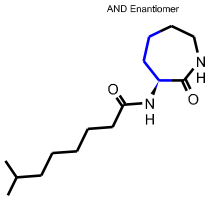 [*]C([*])CC(=[*])[*]	-0.111
FCFP_2	1872154524	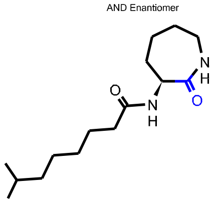 [*]C(=O)[*]	-0.105
FCFP_2	1	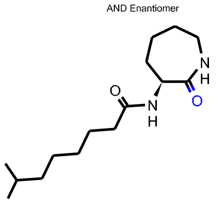 [*]=O	-0.080

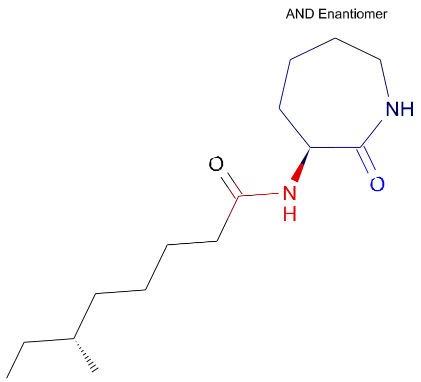

$C_{15}H_{28}N_2O_2$
Molecular Weight: 268.39501
ALogP: 2.685
Rotatable Bonds: 7
Acceptors: 2
Donors: 2

Model Prediction

Prediction: 0.126
Unit: g/kg_body_weight
Mahalanobis Distance: 7.845
Mahalanobis Distance p-value: 0.0356

Mahalanobis Distance: The Mahalanobis distance (MD) is a generalization of the Euclidean distance that accounts for correlations among the X properties. It is calculated as the distance to the center of the training data. The larger the MD, the less trustworthy the prediction.
Mahalanobis Distance p-value: The p-value gives the fraction of training data with an MD greater than or equal to the one for the given sample, assuming normally distributed data. The smaller the p-value, the less trustworthy the prediciton. For highly non-normal X properties (e.g., fingerprints), the MD p-value is wildly inaccurate.

Structural Similar Compounds

Name	N,N'-DIETHYLTHIOUREA	CARBROMAL	TOLBUTAMIDE
Structure	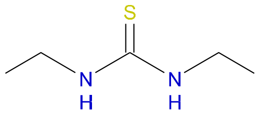	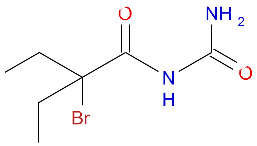	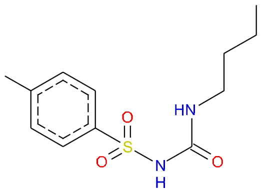
Actual Endpoint (-log C)	4.07016	3.32377	2.3985
Predicted Endpoint (-log C)	3.12792	3.55919	3.32272
Distance	0.525	0.529	0.560
Reference	NCI/NTP TR-149	NCI/NTP TR-173	NCI/NTP TR-031

Model Applicability

Unknown features are fingerprint features in the query molecule, but not found in the training set.

1. OPS PC12 out of range. Value: -2.8089. Training min, max, SD, explained variance: -2.364, 2.9228, 1.079, 0.0263.

Feature Contribution

Top features for positive contribution

Fingerprint	Bit/Smiles	Feature Structure	Score
FCFP_2	-885550502	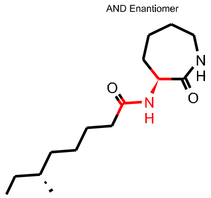 [*]C([*])NC(=[*])[*]	0.115

FCFP_2	3	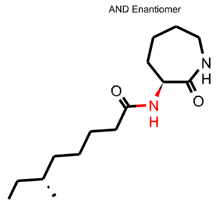<br>[*]O	0.074
**Top Features for negative contribution**			
**Fingerprint**	**Bit/Smiles**	**Feature Structure**	**Score**
FCFP_2	-1272798659	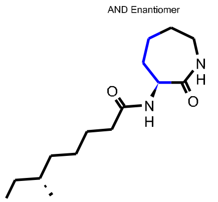<br>[*]C([*])CC(=[*])[*]	-0.111
FCFP_2	1872154524	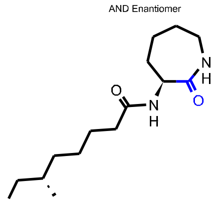<br>[*]C(=O)[*]	-0.105
FCFP_2	1	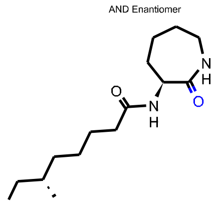<br>[*]=O	-0.080

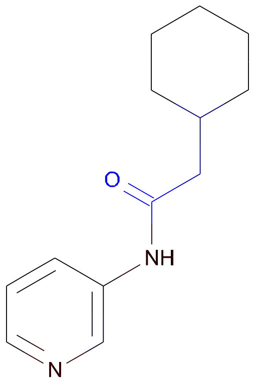

$C_{13}H_{18}N_2O$
Molecular Weight: 218.29481
ALogP: 2.171
Rotatable Bonds: 3
Acceptors: 2
Donors: 1

Model Prediction

Prediction: 0.105
Unit: g/kg_body_weight
Mahalanobis Distance: 8.547
Mahalanobis Distance p-value: 0.0056

Mahalanobis Distance: The Mahalanobis distance (MD) is a generalization of the Euclidean distance that accounts for correlations among the X properties. It is calculated as the distance to the center of the training data. The larger the MD, the less trustworthy the prediction.
Mahalanobis Distance p-value: The p-value gives the fraction of training data with an MD greater than or equal to the one for the given sample, assuming normally distributed data. The smaller the p-value, the less trustworthy the prediciton. For highly non-normal X properties (e.g., fingerprints), the MD p-value is wildly inaccurate.

Structural Similar Compounds

Name	4-(CHLOROACETYL) ACETANILIDE	1-PHENYL-2-THIOUREA	FLUOMETURON
Structure	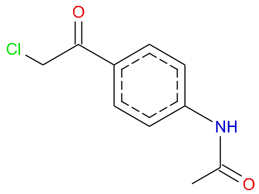	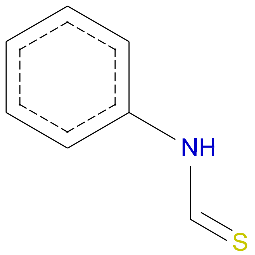	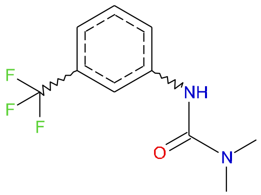
Actual Endpoint (-log C)	3.37137	4.40496	4.31473
Predicted Endpoint (-log C)	3.35582	3.16907	3.73166
Distance	0.369	0.410	0.430
Reference	NCI/NTP TR-177	NCI/NTP TR-148	NCI/NTP TR-195

Model Applicability

Unknown features are fingerprint features in the query molecule, but not found in the training set.

1. OPS PC9 out of range. Value: 3.8294. Training min, max, SD, explained variance: -2.8548, 3.3954, 1.263, 0.0360.
2. OPS PC11 out of range. Value: -3.9518. Training min, max, SD, explained variance: -3.8346, 3.8752, 1.233, 0.0343.

Feature Contribution

Top features for positive contribution

Fingerprint	Bit/Smiles	Feature Structure	Score
FCFP_2	3	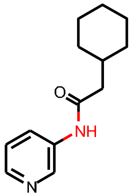 [*]O	0.074

FCFP_2	17	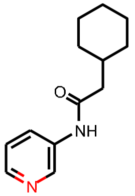 [*]:n:[*]	0.044
FCFP_2	590925877	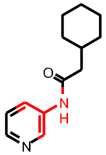 [*]N[c](:[cH]:[*]):[cH]:[*]	0.008

Top Features for negative contribution

Fingerprint	Bit/Smiles	Feature Structure	Score
FCFP_2	-1272798659	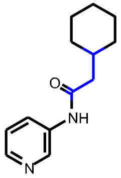 [*]C([*])CC(=[*])[*]	-0.111
FCFP_2	1872154524	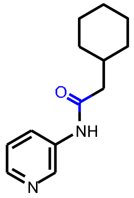 [*]C(=O)[*]	-0.105
FCFP_2	1	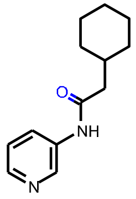 [*]=O	-0.080

Indinavir

TOPKAT_Rat_Maximum_Tolerated_Dose_Feed

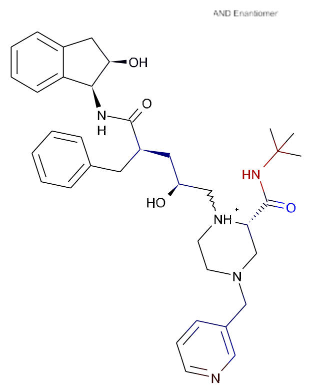

$C_{36}H_{48}N_5O_4$

Molecular Weight: 614.79741

ALogP: 1.521

Rotatable Bonds: 12

Acceptors: 6

Donors: 5

Structural Similar Compounds

Name	SALICYLAZOSULFAPYRIDINE	RESERPINE	FUROSEMIDE
Structure	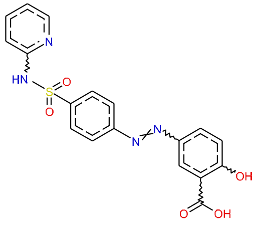	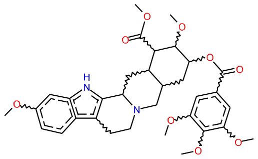	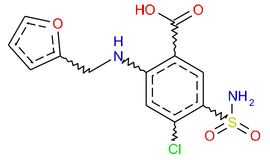
Actual Endpoint (-log C)	3.375	6.13118	4.04236
Predicted Endpoint (-log C)	2.80292	4.38304	2.8614
Distance	1.013	1.085	1.087
Reference	NCI/NTP TR-457	NCI/NTP TR-193	NCI/NTP TR-356

Model Prediction

Prediction: 0.133

Unit: g/kg_body_weight

Mahalanobis Distance: 12.374

Mahalanobis Distance p-value: 2.37e-009

Mahalanobis Distance: The Mahalanobis distance (MD) is a generalization of the Euclidean distance that accounts for correlations among the X properties. It is calculated as the distance to the center of the training data. The larger the MD, the less trustworthy the prediction.

Mahalanobis Distance p-value: The p-value gives the fraction of training data with an MD greater than or equal to the one for the given sample, assuming normally distributed data. The smaller the p-value, the less trustworthy the prediciton. For highly non-normal X properties (e.g., fingerprints), the MD p-value is wildly inaccurate.

Model Applicability

Unknown features are fingerprint features in the query molecule, but not found in the training set.

1. OPS PC8 out of range. Value: 4.2529. Training min, max, SD, explained variance: -3.8548, 3.9137, 1.331, 0.0400.
2. OPS PC9 out of range. Value: 4.5815. Training min, max, SD, explained variance: -2.8548, 3.3954, 1.263, 0.0360.
3. Unknown FCFP_2 feature: 10: [*][NH+]([*])[*]
4. Unknown FCFP_2 feature: -1853714334: [*]C[NH+](C[*])C([*])[*]
5. Unknown FCFP_2 feature: -1817836174: [*]C[C@H]([NH+]([*])[*])C(=[*])[*]
6. Unknown FCFP_2 feature: 1155241219: [*]CC[NH+]([*])[*]
7. Unknown FCFP_2 feature: 906798516: [*]N([*])C[c](:[*]):[*]

Feature Contribution

Top features for positive contribution

Fingerprint	Bit/Smiles	Feature Structure	Score

FCFP_2	-885550502	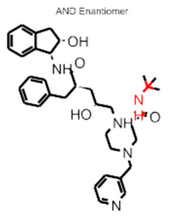 [*]C([*])NC(=[*])[*]	0.115
FCFP_2	3	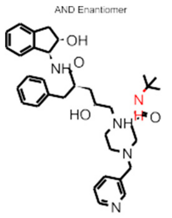 [*]O	0.074
FCFP_2	17	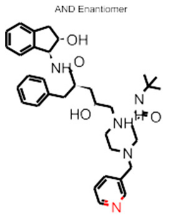 [*]:n:[*]	0.044
**Top Features for negative contribution**			
**Fingerprint**	**Bit/Smiles**	**Feature Structure**	**Score**
FCFP_2	-1272798659	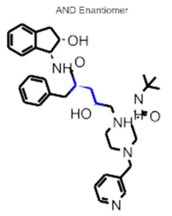 [*]C([*])CC(=[*])[*]	-0.111
FCFP_2	1872154524	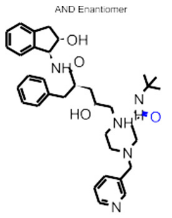 [*]C(=O)[*]	-0.105

FCFP_2	20367772	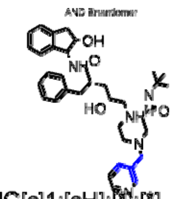<br>[*]C[c]1:[cH]:[*]:[*]:[c]:1:[*]	-

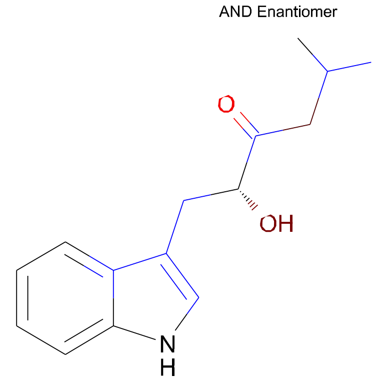

$C_{15}H_{19}NO_2$
Molecular Weight: 245.31685
ALogP: 2.932
Rotatable Bonds: 5
Acceptors: 2
Donors: 2

Model Prediction

Prediction: 1.769
Unit: g/kg_body_weight
Mahalanobis Distance: 8.323
Mahalanobis Distance p-value: 0.000857

Mahalanobis Distance: The Mahalanobis distance (MD) is a generalization of the Euclidean distance that accounts for correlations among the X properties. It is calculated as the distance to the center of the training data. The larger the MD, the less trustworthy the prediction.
Mahalanobis Distance p-value: The p-value gives the fraction of training data with an MD greater than or equal to the one for the given sample, assuming normally distributed data. The smaller the p-value, the less trustworthy the prediciton. For highly non-normal X properties (e.g., fingerprints), the MD p-value is wildly inaccurate.

Structural Similar Compounds

Name	4-HEXYLRESORCINOL	PHENYLBUTAZONE	PROBENECID
Structure	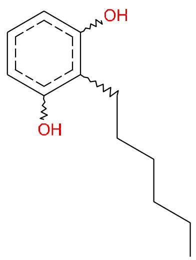	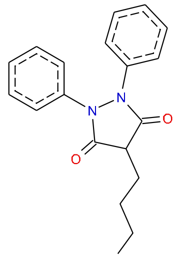	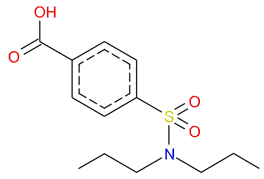
Actual Endpoint (-log C)	3.1915	3.48909	2.85333
Predicted Endpoint (-log C)	2.16134	3.17333	2.4258
Distance	0.657	0.802	0.826
Reference	NCI/NTP TR-330	NCI/NTP TR-367	NCI/NTP TR-395

Model Applicability

Unknown features are fingerprint features in the query molecule, but not found in the training set.

1. All properties and OPS components are within expected ranges.
2. Unknown FCFP_2 feature: 19: [*]:[nH]:[*]
3. Unknown FCFP_2 feature: 2005402822: [*]:[c]1:[*]:[*]:c:[nH]:1
4. Unknown FCFP_2 feature: 307448885: [*]:[c]1:[*]:[*]:[nH]:[c]:1:c:[*]
5. Unknown FCFP_2 feature: 1618184456: [*][c]1:[*]:[*]:[nH]:c:1

Feature Contribution

Top features for positive contribution

Fingerprint	Bit/Smiles	Feature Structure	Score
FCFP_2	1	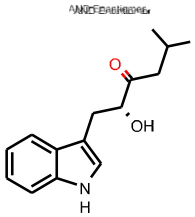 [*]=O	0.511

FCFP_2	3	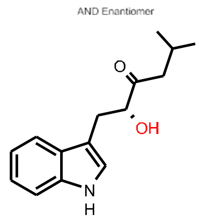<br>[*]O	0.104
FCFP_2	-1272798659	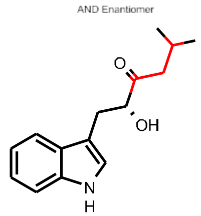<br>[*]C([*])CC(=[*])[*]	0.070
**Top Features for negative contribution**			
**Fingerprint**	**Bit/Smiles**	**Feature Structure**	**Score**
FCFP_2	136597326	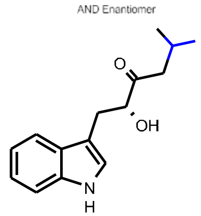<br>[*]C([*])C	-0.489
FCFP_2	203677720	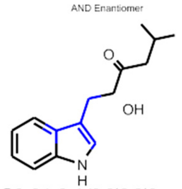<br>[*]C[c]1:[cH]:[*]:[*]<br>:[c]:1:[*]	-0.406
FCFP_2	1872154524	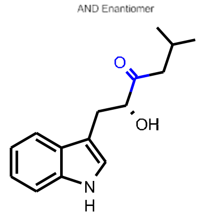<br>[*]C(=O)[*]	-0.307

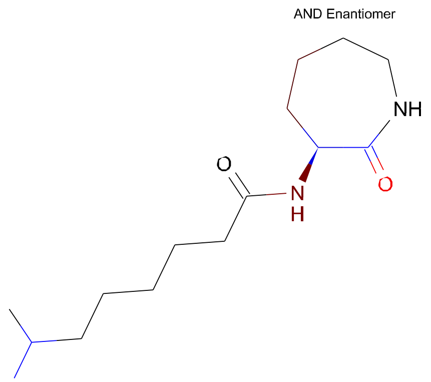

$C_{15}H_{28}N_2O_2$
Molecular Weight: 268.39501
ALogP: 2.685
Rotatable Bonds: 7
Acceptors: 2
Donors: 2

Model Prediction

Prediction: 2.202
Unit: g/kg_body_weight
Mahalanobis Distance: 6.598
Mahalanobis Distance p-value: 0.0573

Mahalanobis Distance: The Mahalanobis distance (MD) is a generalization of the Euclidean distance that accounts for correlations among the X properties. It is calculated as the distance to the center of the training data. The larger the MD, the less trustworthy the prediction.
Mahalanobis Distance p-value: The p-value gives the fraction of training data with an MD greater than or equal to the one for the given sample, assuming normally distributed data. The smaller the p-value, the less trustworthy the prediciton. For highly non-normal X properties (e.g., fingerprints), the MD p-value is wildly inaccurate.

Structural Similar Compounds

Name	LITHOCHLOLIC ACID	4-HEXYLRESORCINOL	PROBENECID
Structure	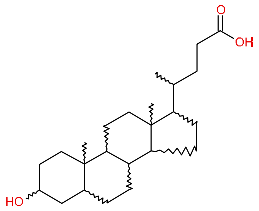	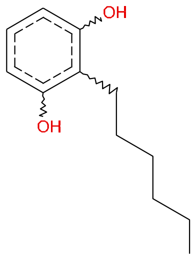	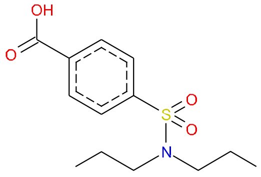
Actual Endpoint (-log C)	2.87689	3.1915	2.85333
Predicted Endpoint (-log C)	3.8262	2.16134	2.4258
Distance	0.668	0.793	0.824
Reference	NCI/NTP TR-175	NCI/NTP TR-330	NCI/NTP TR-395

Model Applicability

Unknown features are fingerprint features in the query molecule, but not found in the training set.

1. All properties and OPS components are within expected ranges.

Feature Contribution

Top features for positive contribution

Fingerprint	Bit/Smiles	Feature Structure	Score
FCFP_2	1	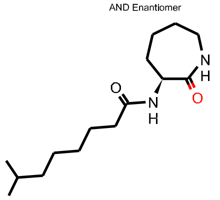 [*]=O	0.511

FCFP_2	3	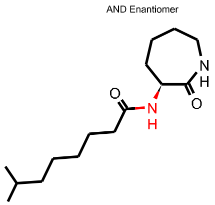 [*]O	0.104
FCFP_2	-1272798659	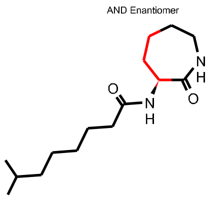 [*]C([*])CC(=[*])[*]	0.070

Top Features for negative contribution

Fingerprint	Bit/Smiles	Feature Structure	Score
FCFP_2	136597326	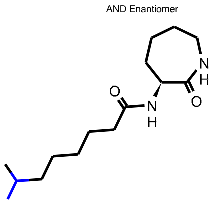 [*]C([*])C	-0.489
FCFP_2	1872154524	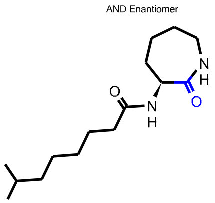 [*]C(=O)[*]	-0.307
FCFP_2	0	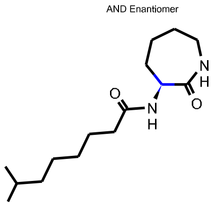 [*]C[*]	-0.290

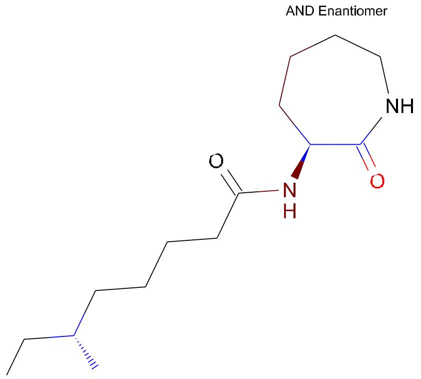

$C_{15}H_{28}N_2O_2$
Molecular Weight: 268.39501
ALogP: 2.685
Rotatable Bonds: 7
Acceptors: 2
Donors: 2

Model Prediction

Prediction: 2.202
Unit: g/kg_body_weight
Mahalanobis Distance: 6.598
Mahalanobis Distance p-value: 0.0573

Mahalanobis Distance: The Mahalanobis distance (MD) is a generalization of the Euclidean distance that accounts for correlations among the X properties. It is calculated as the distance to the center of the training data. The larger the MD, the less trustworthy the prediction.
Mahalanobis Distance p-value: The p-value gives the fraction of training data with an MD greater than or equal to the one for the given sample, assuming normally distributed data. The smaller the p-value, the less trustworthy the prediciton. For highly non-normal X properties (e.g., fingerprints), the MD p-value is wildly inaccurate.

Structural Similar Compounds

Name	LITHOCHOLIC ACID	4-HEXYLRESORCINOL	PROBENECID
Structure	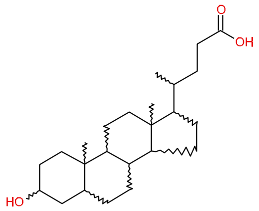	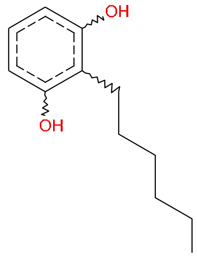	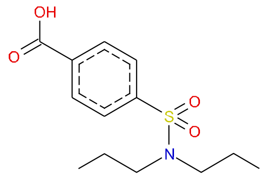
Actual Endpoint (-log C)	2.87689	3.1915	2.85333
Predicted Endpoint (-log C)	3.8262	2.16134	2.4258
Distance	0.668	0.793	0.824
Reference	NCI/NTP TR-175	NCI/NTP TR-330	NCI/NTP TR-395

Model Applicability

Unknown features are fingerprint features in the query molecule, but not found in the training set.

1. All properties and OPS components are within expected ranges.

Feature Contribution

Top features for positive contribution

Fingerprint	Bit/Smiles	Feature Structure	Score
FCFP_2	1	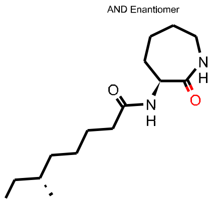 [*]=O	0.511

FCFP_2	3	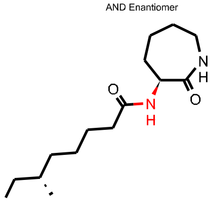 [*]O	0.104
FCFP_2	-1272798659	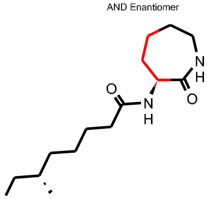 [*]C([*])CC(=[*])[*]	0.070

Top Features for negative contribution

Fingerprint	Bit/Smiles	Feature Structure	Score
FCFP_2	136597326	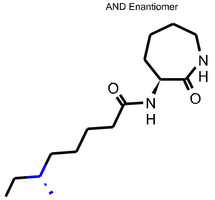 [*]C([*])C	-0.489
FCFP_2	1872154524	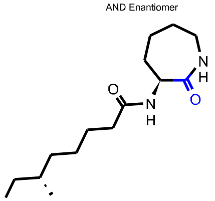 [*]C(=O)[*]	-0.307
FCFP_2	0	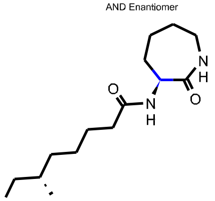 [*]C[*]	-0.290

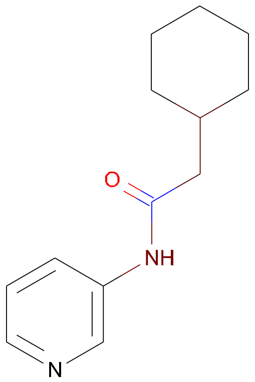

$C_{13}H_{18}N_2O$

Molecular Weight: 218.29481

ALogP: 2.171

Rotatable Bonds: 3

Acceptors: 2

Donors: 1

Model Prediction

Prediction: 0.128

Unit: g/kg_body_weight

Mahalanobis Distance: 7.052

Mahalanobis Distance p-value: 0.0218

Mahalanobis Distance: The Mahalanobis distance (MD) is a generalization of the Euclidean distance that accounts for correlations among the X properties. It is calculated as the distance to the center of the training data. The larger the MD, the less trustworthy the prediction.
Mahalanobis Distance p-value: The p-value gives the fraction of training data with an MD greater than or equal to the one for the given sample, assuming normally distributed data. The smaller the p-value, the less trustworthy the prediciton. For highly non-normal X properties (e.g., fingerprints), the MD p-value is wildly inaccurate.

Structural Similar Compounds

Name	MONURON	M-CRESIDINE	BETA-NITROSTYRENE
Structure	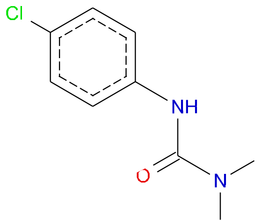	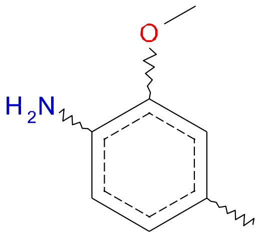	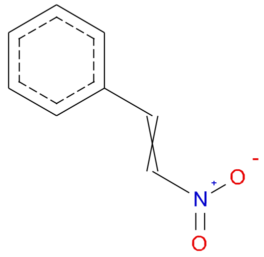
Actual Endpoint (-log C)	3.46878	5.93318	2.6965
Predicted Endpoint (-log C)	3.8732	3.87056	2.69255
Distance	0.494	0.576	0.636
Reference	NCI/NTP TR-266	NCI/NTP TR-105	NCI/NTP TR-170

Model Applicability

Unknown features are fingerprint features in the query molecule, but not found in the training set.

1. All properties and OPS components are within expected ranges.

Feature Contribution

Top features for positive contribution			
**Fingerprint**	**Bit/Smiles**	**Feature Structure**	**Score**
FCFP_2	1	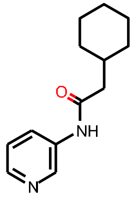 [*]=O	0.511

FCFP_2	3	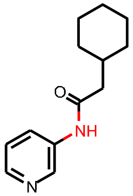 [*]O	0.104
FCFP_2	-1272798659	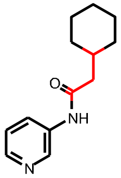 [*]C([*])CC(=[*])[*]	0.070
**Top Features for negative contribution**			
**Fingerprint**	**Bit/Smiles**	**Feature Structure**	**Score**
FCFP_2	1872154524	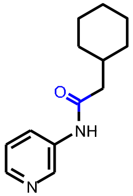 [*]C(=O)[*]	-0.307
FCFP_2	0	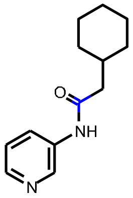 [*]C[*]	-0.290

Indinavir

TOPKAT_Rat_Maximum_Tolerated_Dose_Gavage

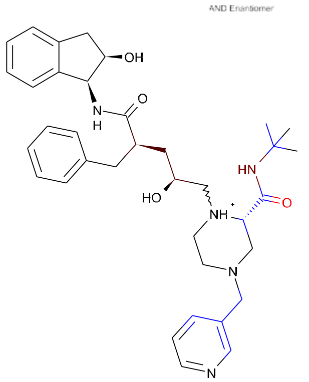

$C_{36}H_{48}N_5O_4$
Molecular Weight: 614.79741
ALogP: 1.521
Rotatable Bonds: 12
Acceptors: 6
Donors: 5

Structural Similar Compounds

Name	OCHRATOXIN	AMPICILLIN TRIHYDRATE	PENICILLIN VK
Structure	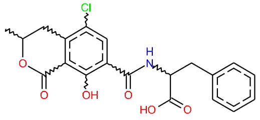	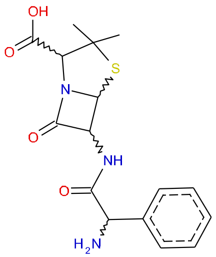	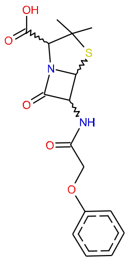
Actual Endpoint (-log C)	6.28396	2.36724	2.54455
Predicted Endpoint (-log C)	5.12358	2.27651	3.9702
Distance	1.187	1.426	1.462
Reference	NCI/NTP TR-358	NCI/NTP TR-318	NCI/NTP TR-336

Model Prediction

Prediction: 0.480
Unit: g/kg_body_weight
Mahalanobis Distance: 17.572
Mahalanobis Distance p-value: 1.84e-016

Mahalanobis Distance: The Mahalanobis distance (MD) is a generalization of the Euclidean distance that accounts for correlations among the X properties. It is calculated as the distance to the center of the training data. The larger the MD, the less trustworthy the prediction.
Mahalanobis Distance p-value: The p-value gives the fraction of training data with an MD greater than or equal to the one for the given sample, assuming normally distributed data. The smaller the p-value, the less trustworthy the prediciton. For highly non-normal X properties (e.g., fingerprints), the MD p-value is wildly inaccurate.

Model Applicability

Unknown features are fingerprint features in the query molecule, but not found in the training set.

1. Molecular_Weight out of range. Value: 614.8. Training min, max, mean, SD: 68.074, 434.63, 171.13, 85.06.
2. Num_H_Donors out of range. Value: 5. Training min, max, mean, SD: 0, 3, 0.4375, 0.8311.
3. Num_AromaticRings out of range. Value: 3. Training min, max, mean, SD: 0, 2, 0.5625, 0.693.
4. OPS PC6 out of range. Value: -2.5695. Training min, max, SD, explained variance: -2.4321, 2.9885, 1.256, 0.0488.
5. OPS PC9 out of range. Value: -3.2385. Training min, max, SD, explained variance: -2.7086, 2.9267, 1.019, 0.0321.
6. Unknown FCFP_2 feature: 10: [*][NH+]([*])[*]
7. Unknown FCFP_2 feature: -1853714334: [*]C[NH+](C[*])C([*])[*]
8. Unknown FCFP_2 feature: -1817836174: [*]C[C@H]([NH+]([*])[*])C(=[*])[*]
9. Unknown FCFP_2 feature: 1155241219: [*]CC[NH+]([*])[*]
10. Unknown FCFP_2 feature: 906798516: [*]N([*])C[c](:[*]):[*]
11. Unknown FCFP_2 feature: -415156552: [*]NC(C)(C)C

Feature Contribution

Top features for positive contribution

Fingerprint	Bit/Smiles	Feature Structure	Score

FCFP_2	1	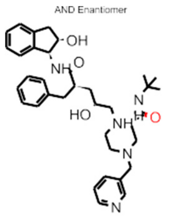 [*]=O	0.511
FCFP_2	3	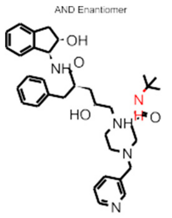 [*]O	0.104
FCFP_2	-1272798659	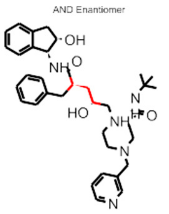 [*]C([*])CC(=[*])[*]	0.070
**Top Features for negative contribution**			
**Fingerprint**	**Bit/Smiles**	**Feature Structure**	**Score**
FCFP_2	136597326	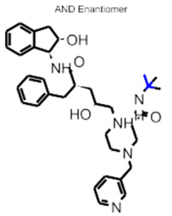 [*]C([*])C	-0.489
FCFP_2	203677720	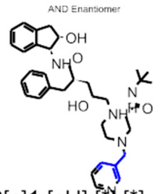 [*]C[c]1:[cH]:[*]:[*]:[c]:1:[*]	-0.406

FCFP_2	187215452	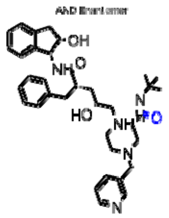<br>[*]C(=O)[*]	

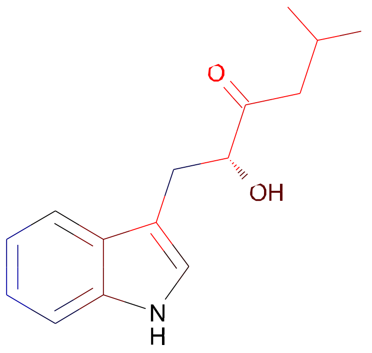

$C_{15}H_{19}NO_2$
Molecular Weight: 245.31685
ALogP: 2.932
Rotatable Bonds: 5
Acceptors: 2
Donors: 2

Model Prediction

Prediction: 0.308
Unit: g/kg_body_weight
Mahalanobis Distance: 19.423
Mahalanobis Distance p-value: 3.23e-007

Mahalanobis Distance: The Mahalanobis distance (MD) is a generalization of the Euclidean distance that accounts for correlations among the X properties. It is calculated as the distance to the center of the training data. The larger the MD, the less trustworthy the prediction.
Mahalanobis Distance p-value: The p-value gives the fraction of training data with an MD greater than or equal to the one for the given sample, assuming normally distributed data. The smaller the p-value, the less trustworthy the prediciton. For highly non-normal X properties (e.g., fingerprints), the MD p-value is wildly inaccurate.

Structural Similar Compounds

Name	BUFURALOL	INDOLE; 3-(2-AMINOPROPYL)-	PRODOLIC ACID
Structure	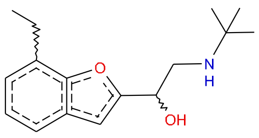	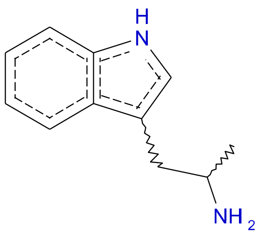	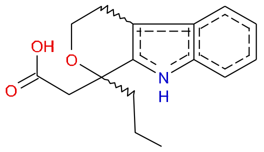
Actual Endpoint (-log C)	2.542	3.899	2.284
Predicted Endpoint (-log C)	2.50976	2.60392	3.15318
Distance	0.437	0.447	0.450
Reference	ARZNAD 27;1410;77	TXAPA9 4;547;62	AGACBH 4;370;74

Model Applicability

Unknown features are fingerprint features in the query molecule, but not found in the training set.

1. All properties and OPS components are within expected ranges.
2. Unknown ECFP_2 feature: 80433051: [*]C[C@@H](O)C(=[*])[*]
3. Unknown FCFP_6 feature: 19: [*]:[nH]:[*]
4. Unknown FCFP_6 feature: 16: [*][c](:[*]):[*]
5. Unknown FCFP_6 feature: 2005402822: [*]:[c]1:[*]:[*]:[cH]:[nH]:1
6. Unknown FCFP_6 feature: 307448885: [*]:[cH]:[c]1:[nH]:[*]:[*]:[c]:1:[*]
7. Unknown FCFP_6 feature: 1618184456: [*][c]1:[*]:[*]:[nH]:[cH]:1
8. Unknown FCFP_6 feature: 1618154665: [*]:[cH]:[cH]:[c](:[*]):[*]

Feature Contribution

Top features for positive contribution

Fingerprint	Bit/Smiles	Feature Structure	Score

ECFP_6	642810091	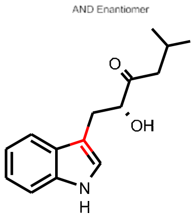<br>[*][c](:[*]):[*]	0.281
ECFP_6	1035165602	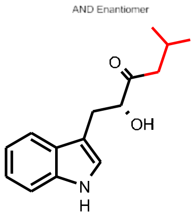<br>[*]CC(C)C	0.172
ECFP_6	-1074141656	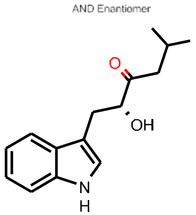<br>[*]=O	0.142

Top Features for negative contribution

Fingerprint	Bit/Smiles	Feature Structure	Score
ECFP_6	734603939	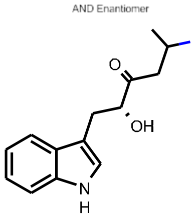<br>[*]C	-0.201
FCFP_6	3	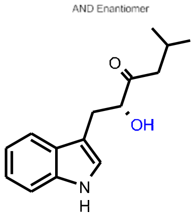<br>[*]O	-0.107

ECFP_6	199702179	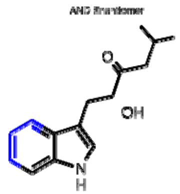[*]:[cH]:[cH]:[cH]:[*]	-

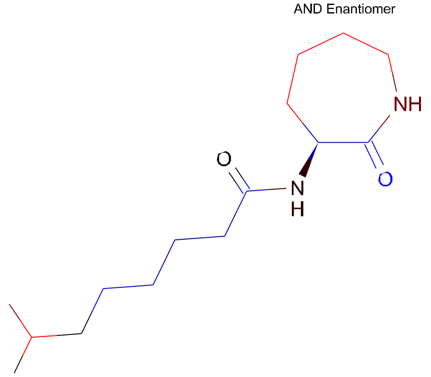

$C_{15}H_{28}N_2O_2$
Molecular Weight: 268.39501
ALogP: 2.685
Rotatable Bonds: 7
Acceptors: 2
Donors: 2

Structural Similar Compounds

Name	THIOUREA; N;N'-DIBUTYL	5-ETHYL-5-ISOAMYLBARBITURIC ACID	CARMOFUR
Structure	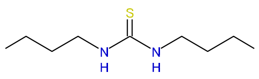	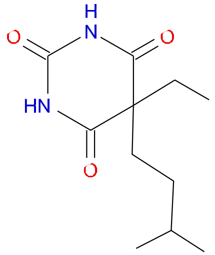	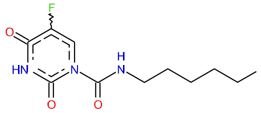
Actual Endpoint (-log C)	2.731	2.957	2.982
Predicted Endpoint (-log C)	2.5023	2.59837	2.84661
Distance	0.572	0.596	0.600
Reference	JPETAB 90;260;47	ARZNAD 21;719;71	NIIRDN 6;191;82

Model Prediction

Prediction: 2.732
Unit: g/kg_body_weight
Mahalanobis Distance: 27.707
Mahalanobis Distance p-value: 7.31e-048

Mahalanobis Distance: The Mahalanobis distance (MD) is a generalization of the Euclidean distance that accounts for correlations among the X properties. It is calculated as the distance to the center of the training data. The larger the MD, the less trustworthy the prediction.
Mahalanobis Distance p-value: The p-value gives the fraction of training data with an MD greater than or equal to the one for the given sample, assuming normally distributed data. The smaller the p-value, the less trustworthy the prediciton. For highly non-normal X properties (e.g., fingerprints), the MD p-value is wildly inaccurate.

Model Applicability

Unknown features are fingerprint features in the query molecule, but not found in the training set.

1. All properties and OPS components are within expected ranges.
2. Unknown ECFP_2 feature: -2097159651: [*]C[C@H](N[*])C(=[*])[*]

Feature Contribution

Top features for positive contribution

Fingerprint	Bit/Smiles	Feature Structure	Score
ECFP_6	642810091	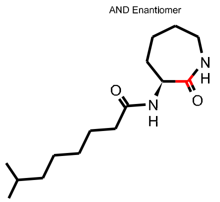 [*][c](:[*]):[*]	0.281

ECFP_6	-1897341097	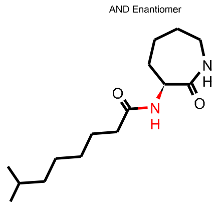 [*]N[*]	0.216
ECFP_6	1035165602	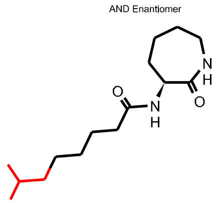 [*]CC(C)C	0.172

Top Features for negative contribution

Fingerprint	Bit/Smiles	Feature Structure	Score
ECFP_6	2106656448	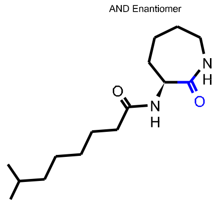 [*]C(=O)[*]	-0.352
FCFP_6	566058135	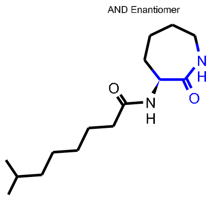 [*]NC(=O)C([*])[*]	-0.216
ECFP_6	734603939	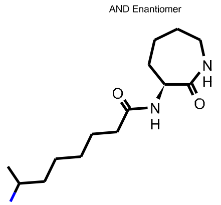 [*]C	-0.201

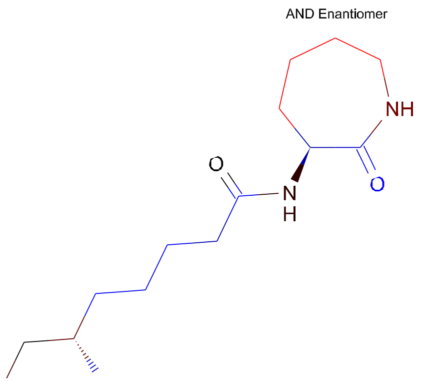

$C_{15}H_{28}N_2O_2$
Molecular Weight: 268.39501
ALogP: 2.685
Rotatable Bonds: 7
Acceptors: 2
Donors: 2

Model Prediction

Prediction: 3.110
Unit: g/kg_body_weight
Mahalanobis Distance: 26.971
Mahalanobis Distance p-value: 5.1e-043

Mahalanobis Distance: The Mahalanobis distance (MD) is a generalization of the Euclidean distance that accounts for correlations among the X properties. It is calculated as the distance to the center of the training data. The larger the MD, the less trustworthy the prediction.
Mahalanobis Distance p-value: The p-value gives the fraction of training data with an MD greater than or equal to the one for the given sample, assuming normally distributed data. The smaller the p-value, the less trustworthy the prediciton. For highly non-normal X properties (e.g., fingerprints), the MD p-value is wildly inaccurate.

Structural Similar Compounds

Name	THIOUREA; N;N'-DIBUTYL	2-HYDROXY-3-ETHYLHEPTANOIC ACID	CARMOFUR
Structure	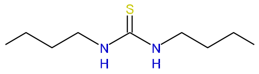	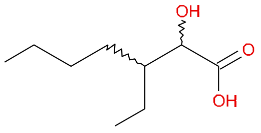	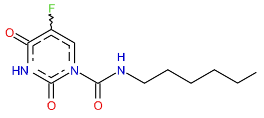
Actual Endpoint (-log C)	2.731	1.71	2.982
Predicted Endpoint (-log C)	2.5023	1.54092	2.84661
Distance	0.566	0.589	0.597
Reference	JPETAB 90;260;47	AIHAAP 23;95;62	NIIRDN 6;191;82

Model Applicability

Unknown features are fingerprint features in the query molecule, but not found in the training set.

1. All properties and OPS components are within expected ranges.
2. Unknown ECFP_2 feature: -2097159651: [*]C[C@H](N[*])C(=[*])[*]

Feature Contribution

Top features for positive contribution

Fingerprint	Bit/Smiles	Feature Structure	Score
ECFP_6	642810091	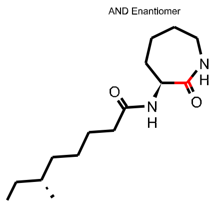 [*][c](:[*]):[*]	0.281

ECFP_6	-1897341097	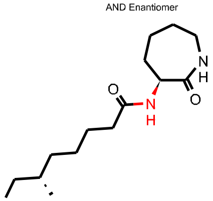 [*]N[*]	0.216
ECFP_6	-1074141656	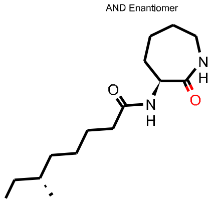 [*]=O	0.142

Top Features for negative contribution

**Fingerprint**	**Bit/Smiles**	**Feature Structure**	**Score**
ECFP_6	2106656448	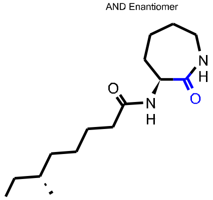 [*]C(=O)[*]	-0.352
FCFP_6	566058135	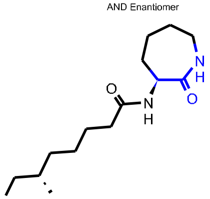 [*]NC(=O)C([*])[*]	-0.216
ECFP_6	734603939	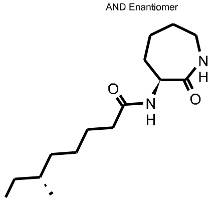 [*]C	-0.201

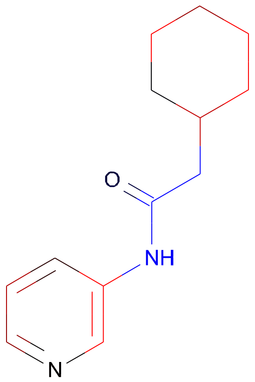

$C_{13}H_{18}N_2O$
Molecular Weight: 218.29481
ALogP: 2.171
Rotatable Bonds: 3
Acceptors: 2
Donors: 1

Structural Similar Compounds

Name	PYRACARBOLID	GLUTETHIMIDE	CHLORTHENOXAZINE
Structure	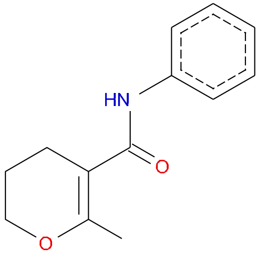	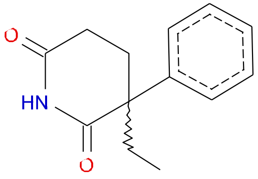	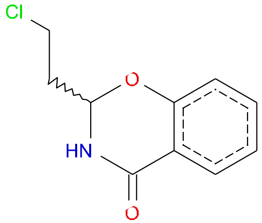
Actual Endpoint (-log C)	1.161	2.559	1.326
Predicted Endpoint (-log C)	2.21704	2.36261	2.7131
Distance	0.418	0.442	0.455
Reference	85JFAN A354;83	27ZQAG -;233;72	ARZNAD 7;651;57

Model Applicability

Unknown features are fingerprint features in the query molecule, but not found in the training set.

1. All properties and OPS components are within expected ranges.
2. Unknown FCFP_6 feature: 16: [*][c](:[*]):[*]
3. Unknown FCFP_6 feature: 1618154665: [*]:[cH]:[cH]:[c](:[*]):[*]
4. Unknown FCFP_6 feature: 1747237384: [*]:[cH]:n:[cH]:[*]

Model Prediction

Prediction: 1.787
Unit: g/kg_body_weight
Mahalanobis Distance: 18.058
Mahalanobis Distance p-value: 0.000573

Mahalanobis Distance: The Mahalanobis distance (MD) is a generalization of the Euclidean distance that accounts for correlations among the X properties. It is calculated as the distance to the center of the training data. The larger the MD, the less trustworthy the prediction.
Mahalanobis Distance p-value: The p-value gives the fraction of training data with an MD greater than or equal to the one for the given sample, assuming normally distributed data. The smaller the p-value, the less trustworthy the prediciton. For highly non-normal X properties (e.g., fingerprints), the MD p-value is wildly inaccurate.

Feature Contribution

Top features for positive contribution

Fingerprint	Bit/Smiles	Feature Structure	Score
ECFP_6	642810091	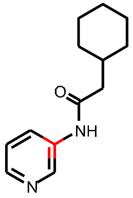 [*][c](:[*]):[*]	0.281

ECFP_6	-1897341097	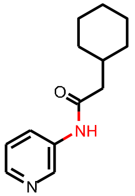 [*]N[*]	0.216
ECFP_6	-1074141656	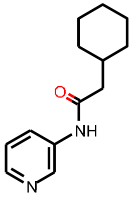 [*]=O	0.142
**Top Features for negative contribution**			
**Fingerprint**	**Bit/Smiles**	**Feature Structure**	**Score**
ECFP_6	655739385	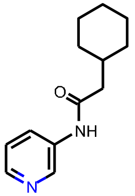 [*]:n:[*]	-0.239
FCFP_6	566058135	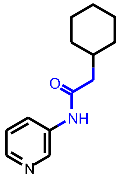 [*]NC(=O)C([*])[*]	-0.216
FCFP_6	3	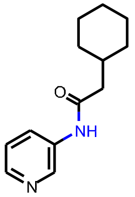 [*]O	-0.107

Indinavir

TOPKAT_Rat_Oral_LD50

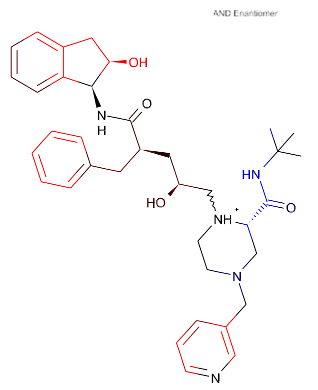

$C_{36}H_{48}N_5O_4$
Molecular Weight: 614.79741
ALogP: 1.521
Rotatable Bonds: 12
Acceptors: 6
Donors: 5

Model Prediction

Prediction: 3.862
Unit: g/kg_body_weight
Mahalanobis Distance: 25.828
Mahalanobis Distance p-value: 6.43e-036

Mahalanobis Distance: The Mahalanobis distance (MD) is a generalization of the Euclidean distance that accounts for correlations among the X properties. It is calculated as the distance to the center of the training data. The larger the MD, the less trustworthy the prediction.
Mahalanobis Distance p-value: The p-value gives the fraction of training data with an MD greater than or equal to the one for the given sample, assuming normally distributed data. The smaller the p-value, the less trustworthy the prediciton. For highly non-normal X properties (e.g., fingerprints), the MD p-value is wildly inaccurate.

Structural Similar Compounds

Name	FEBANTEL	LABETALOL .HCI (HCI STRIPPED)	MOPIDAMOL
Structure	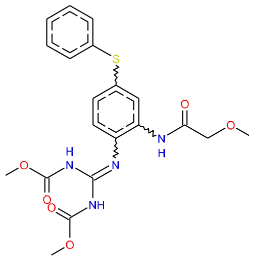	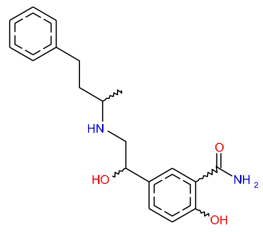	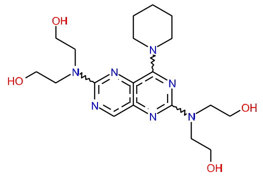
Actual Endpoint (-log C)	1.624	2.191	2.148
Predicted Endpoint (-log C)	2.37098	2.18158	2.45739
Distance	0.995	1.021	1.028
Reference	ARZNAD 28;2193;78	KSRNAM 21;6307;87	MDACAP 17;89;81

Model Applicability

Unknown features are fingerprint features in the query molecule, but not found in the training set.

1. All properties and OPS components are within expected ranges.
2. Unknown ECFP_2 feature: 1976330679: [*][NH+]([*])[*]
3. Unknown ECFP_2 feature: 1134829831: [*]C[NH+](C[*])C([*])[*]
4. Unknown ECFP_2 feature: -1924540582: [*]C[C@H]([NH+]([*])[*])C(=[*])[*]
5. Unknown ECFP_2 feature: -244159614: [*]CC[NH+]([*])[*]
6. Unknown ECFP_2 feature: 474121058: [*]C([*])C[NH+]([*])[*]
7. Unknown FCFP_6 feature: 10: [*][NH+]([*])[*]
8. Unknown FCFP_6 feature: 16: [*][c](:[*]):[*]
9. Unknown FCFP_6 feature: -1853714334: [*]C[NH+](C[*])C([*])[*]
10. Unknown FCFP_6 feature: -1817836174: [*]C[C@H]([NH+]([*])[*])C(=[*])[*]
11. Unknown FCFP_6 feature: 1155241219: [*]CC[NH+]([*])[*]
12. Unknown FCFP_6 feature: 906798516: [*]N([*])C[c](:[*]):[*]
13. Unknown FCFP_6 feature: 1618154665: [*]:[cH]:[cH]:[c](:[*]):[*]
14. Unknown FCFP_6 feature: 1747237384: [*]:[cH]:n:[cH]:[*]
15. Unknown FCFP_6 feature: 1186393305: [*]N[C@@H]1[C@H]([*])[*][*]:[c]1:[*]

Feature Contribution

Top features for positive contribution

Fingerprint	Bit/Smiles	Feature Structure	Score

ECFP_6	642810091	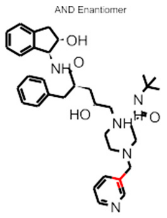 [*][c](:[*]):[*]	0.281
ECFP_6	-1897341097	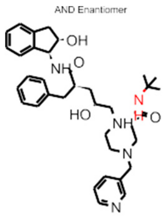 [*]N[*]	0.216
ECFP_6	1571214559	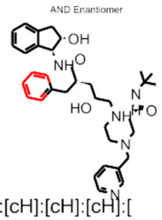 [*]1:[cH]:[cH]:[cH]:[cH]:[cH]:1	0.190
**Top Features for negative contribution**			
**Fingerprint**	**Bit/Smiles**	**Feature Structure**	**Score**
ECFP_6	655739385	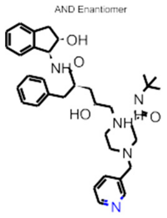 [*]:n:[*]	-0.239
FCFP_6	566058135	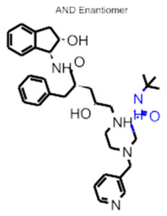 [*]NC(=O)C([*])[*]	-0.216

ECFP_6	73460393	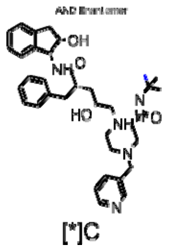	-
